# Supplementary figures and images for: High-resolution imaging of the osteogenic and angiogenic interface at the site of murine cranial bone defect repair via multiphoton microscopy (part 1 of 2)
Source: eLife. 2022 Nov 3;11:e83146. doi: 10.7554/eLife.83146 (PMC9678361; doi:10.7554/eLife.83146)

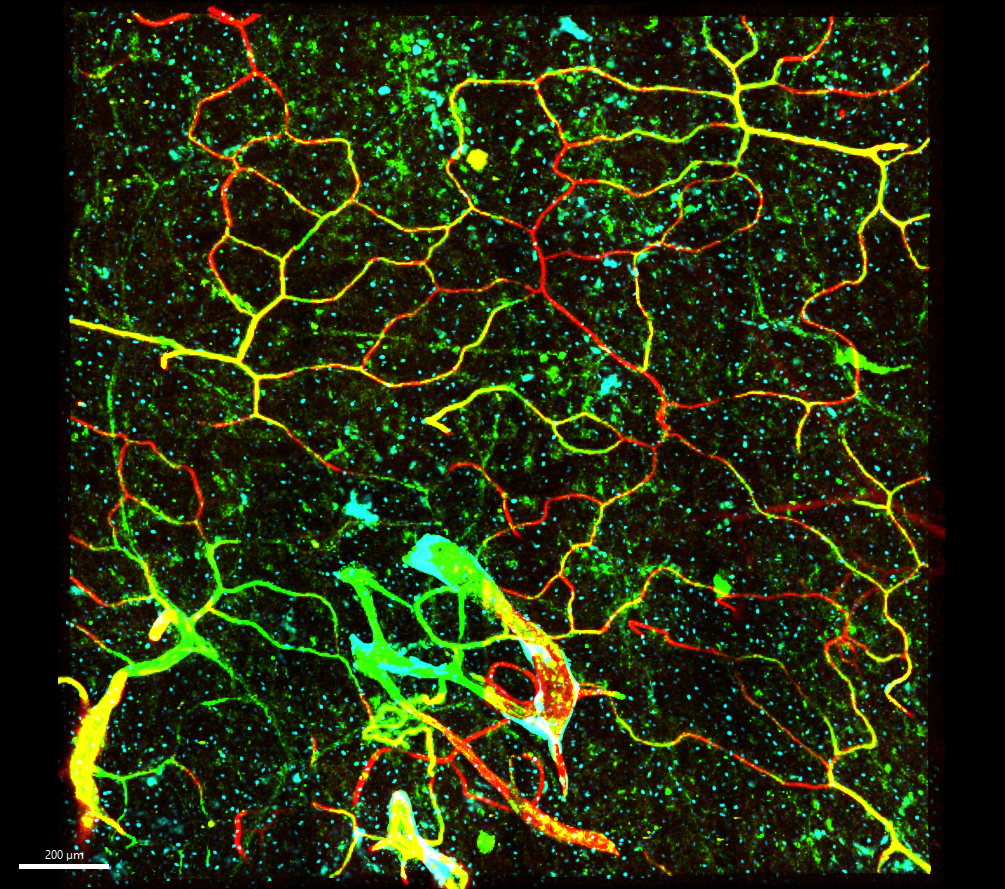

Supplement: Figure 1—source data 1. [file elife-83146-fig1-data1.zip › Figure 1/000 non injury normal bone top side scan top 23 slices cd31 endo gfp.tif]

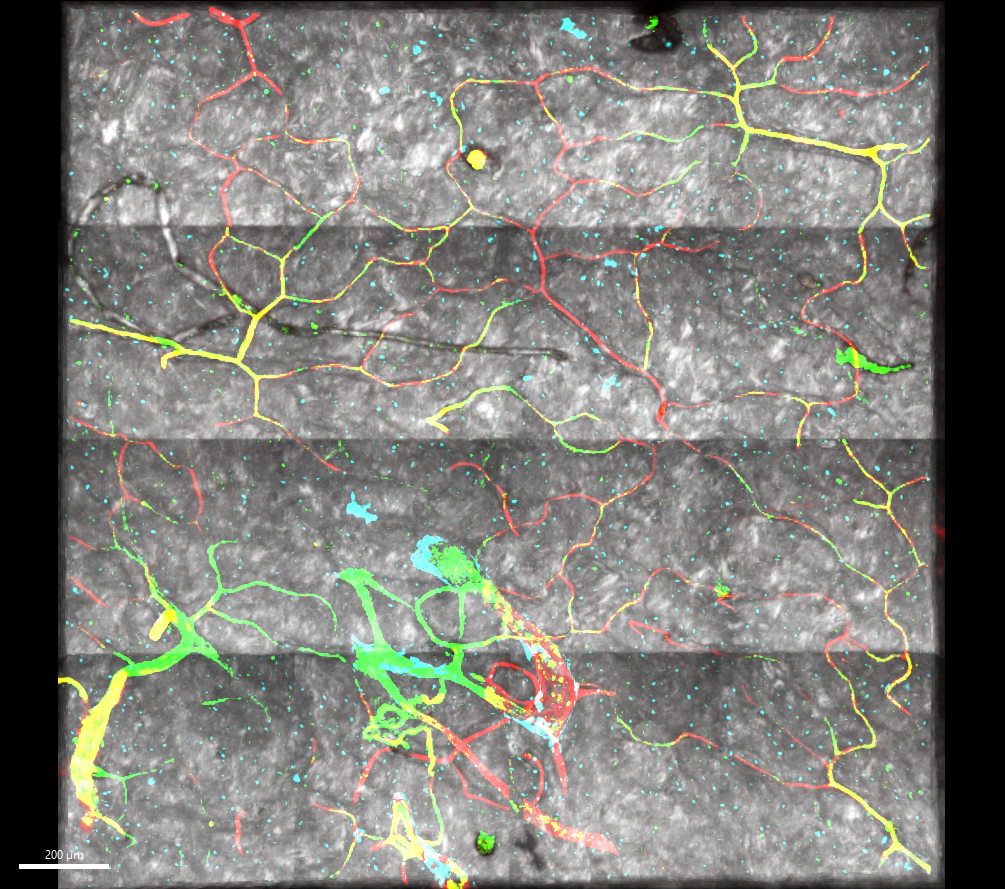

Supplement: Figure 1—source data 1. [file elife-83146-fig1-data1.zip › Figure 1/000 non injury normal bone top side scan top 23 slices cd31 endo gfp-shg.tif]

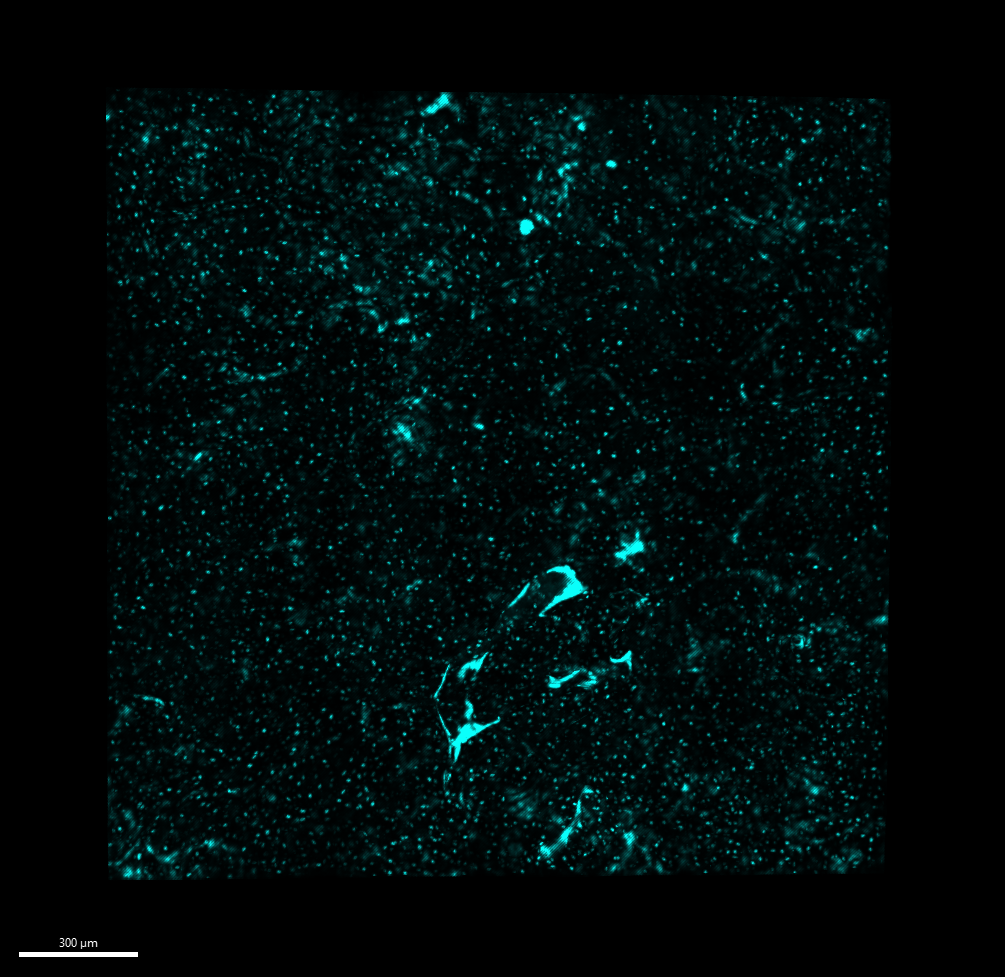

Supplement: Figure 1—source data 1. [file elife-83146-fig1-data1.zip › Figure 1/000 non injury normal bone top side scan top 23 slices gfp.tif]

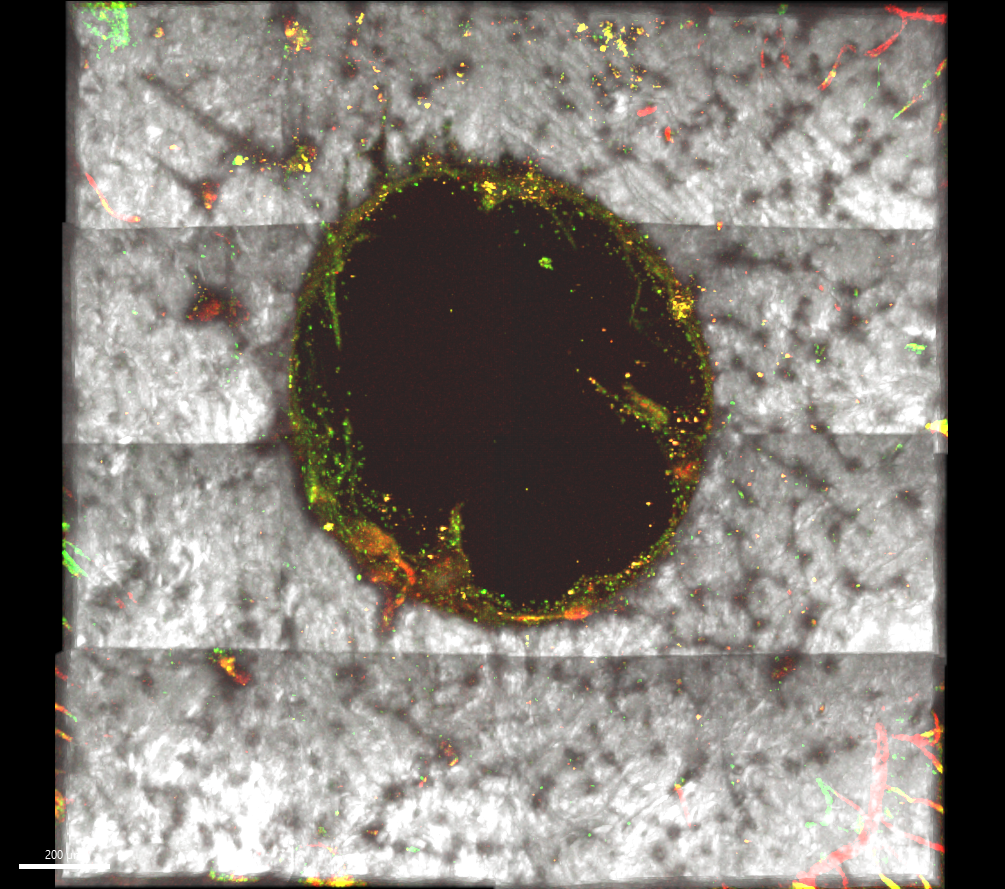

Supplement: Figure 1—source data 1. [file elife-83146-fig1-data1.zip › Figure 1/002 1mm day 1 top 20 slices endo cd31 gfp shg.tif]

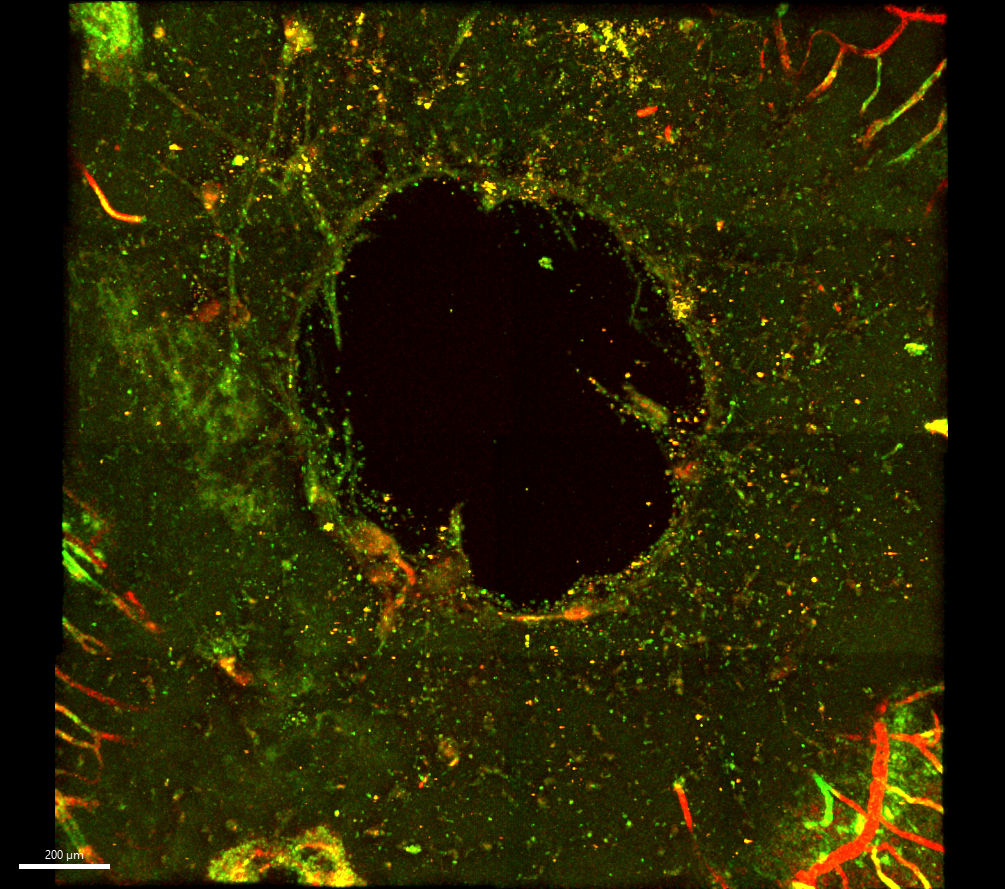

Supplement: Figure 1—source data 1. [file elife-83146-fig1-data1.zip › Figure 1/002 1mm day 1 top 20 slices endo cd31 gfp.tif]

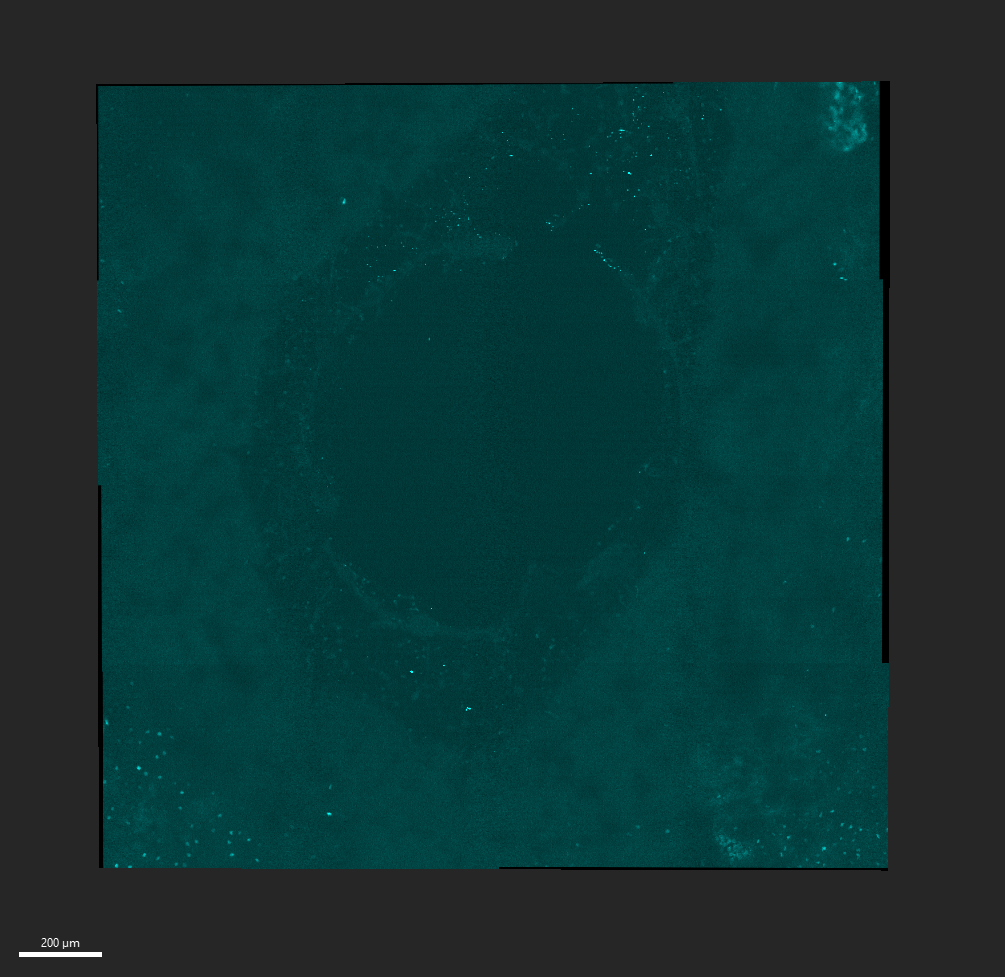

Supplement: Figure 1—source data 1. [file elife-83146-fig1-data1.zip › Figure 1/002 1mm day 1 top slices gfp.tif]

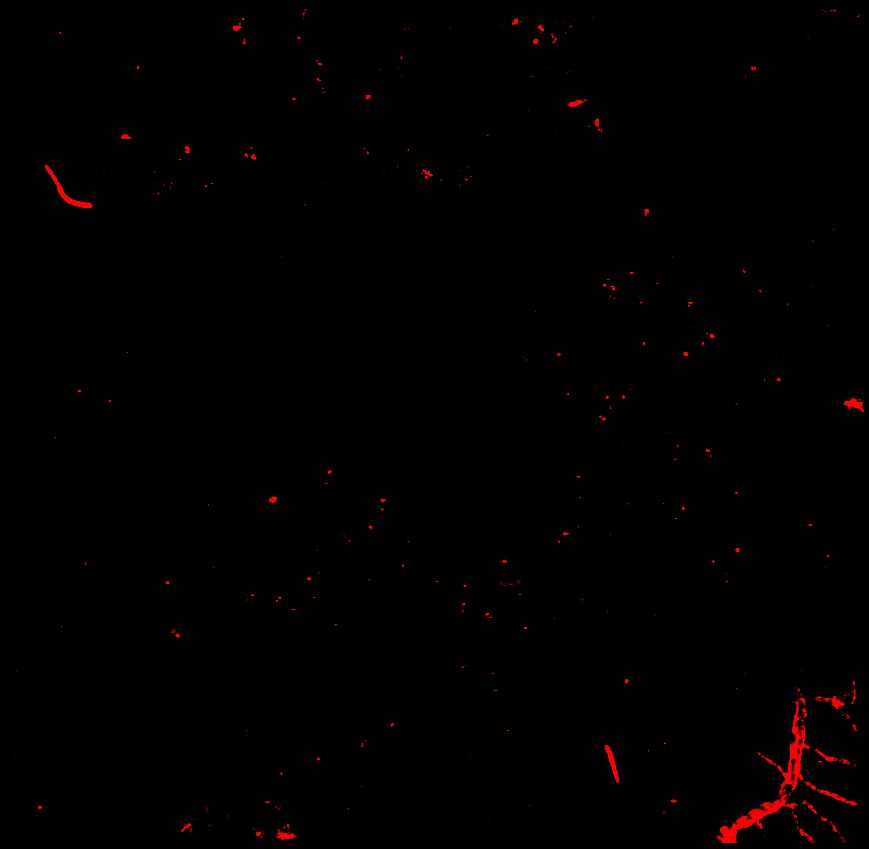

Supplement: Figure 1—source data 1. [file elife-83146-fig1-data1.zip › Figure 1/002 day 1 top cd31only.tif]

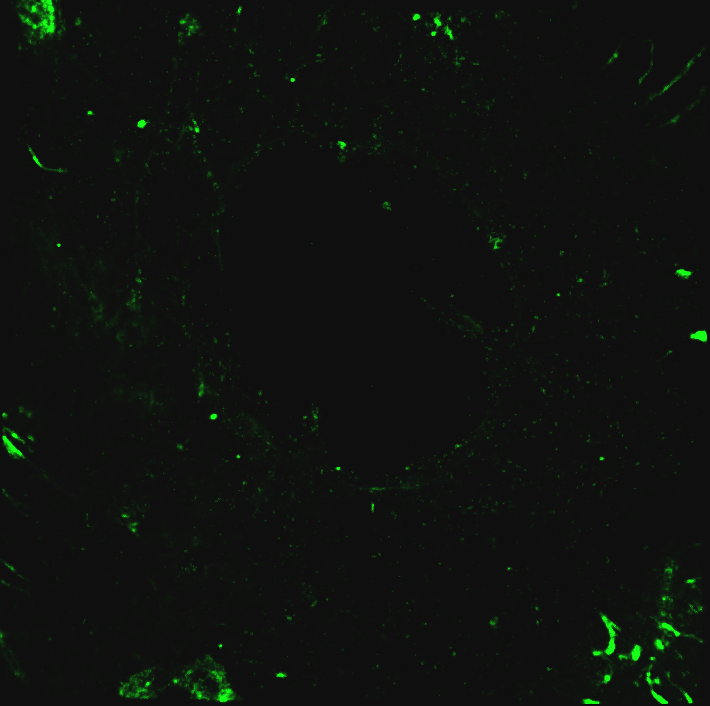

Supplement: Figure 1—source data 1. [file elife-83146-fig1-data1.zip › Figure 1/002 day 1 top endo.tif]

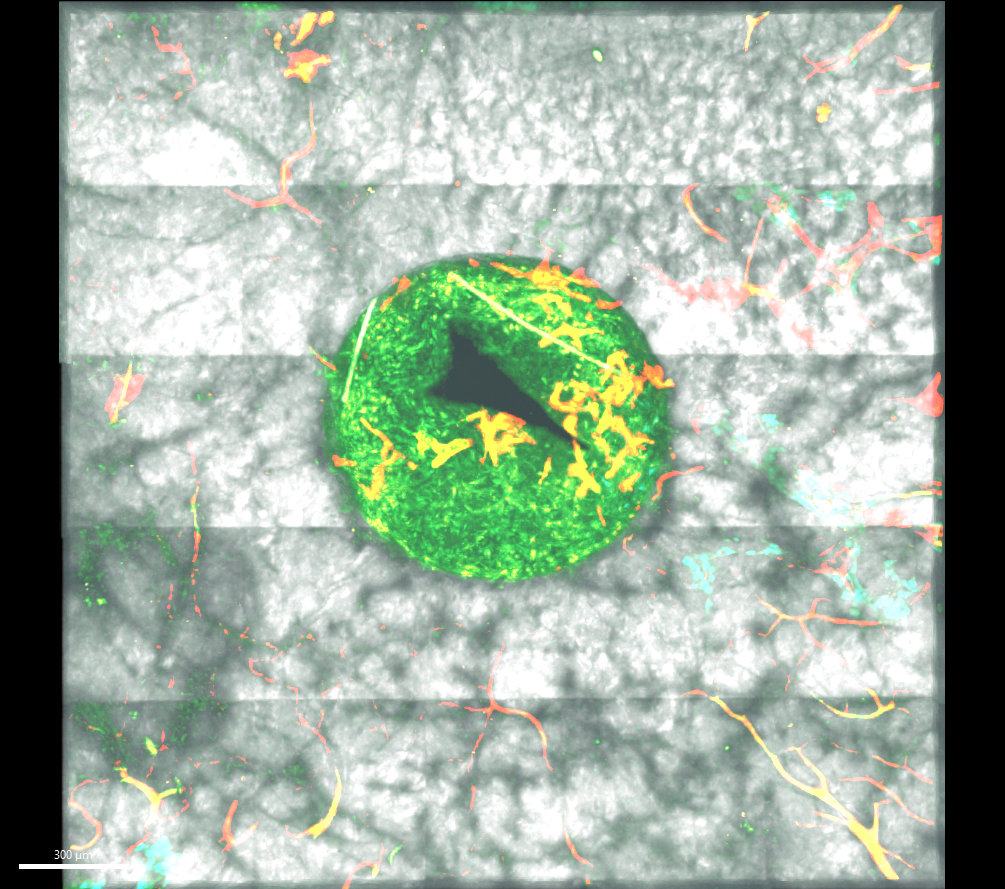

Supplement: Figure 1—source data 1. [file elife-83146-fig1-data1.zip › Figure 1/004 1mm day 3 top 23 slices endo cd31 gfp shg.tif]

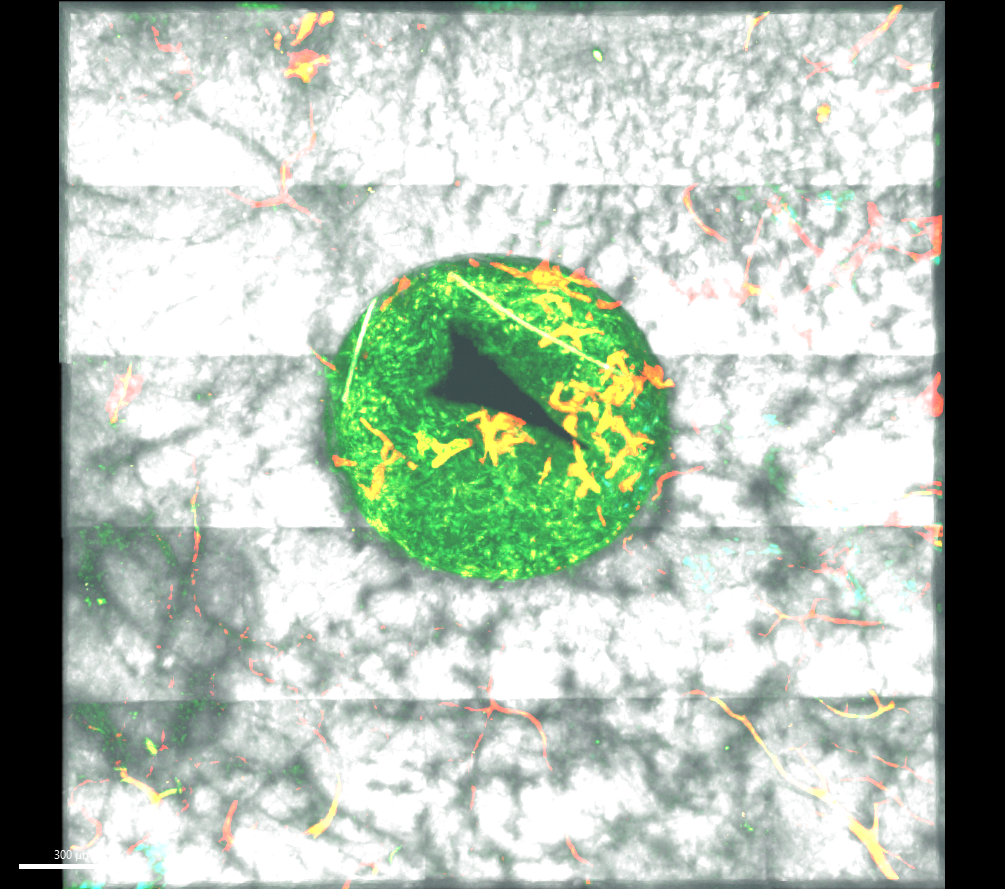

Supplement: Figure 1—source data 1. [file elife-83146-fig1-data1.zip › Figure 1/004 1mm day 3 top 23 slices endo cd31 gfp shg-2.tif]

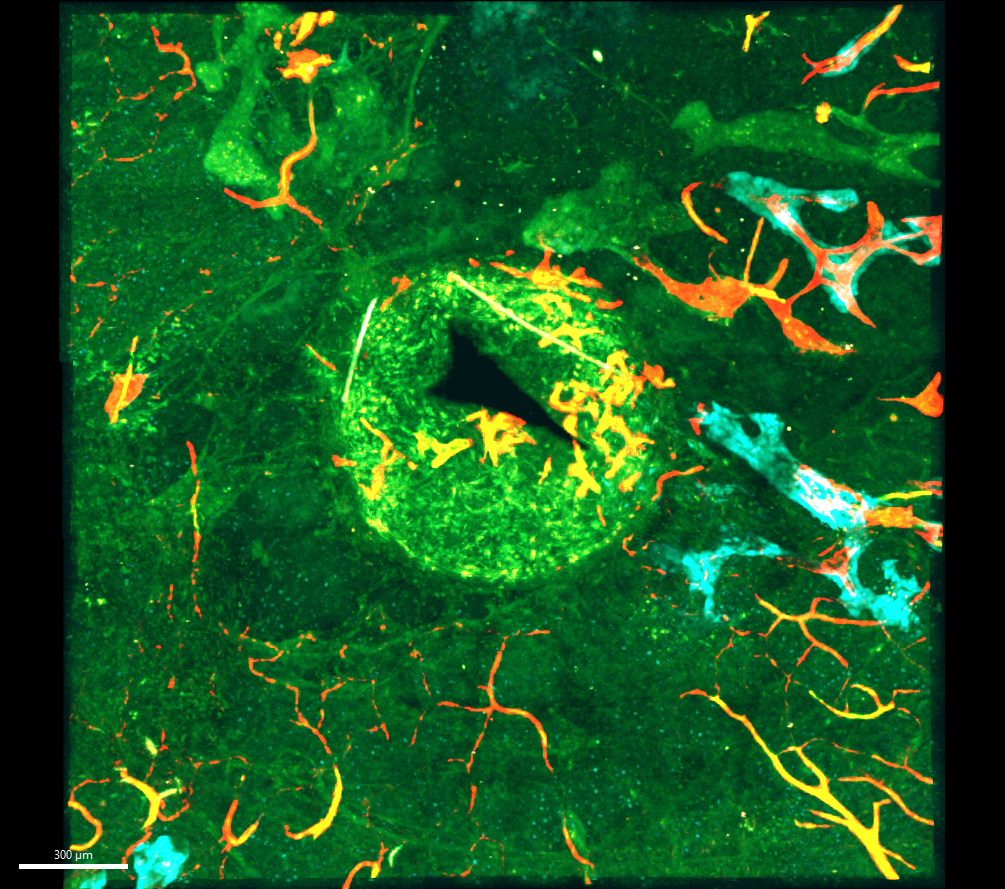

Supplement: Figure 1—source data 1. [file elife-83146-fig1-data1.zip › Figure 1/004 1mm day 3 top 23 slices endo cd31 gfp.tif]

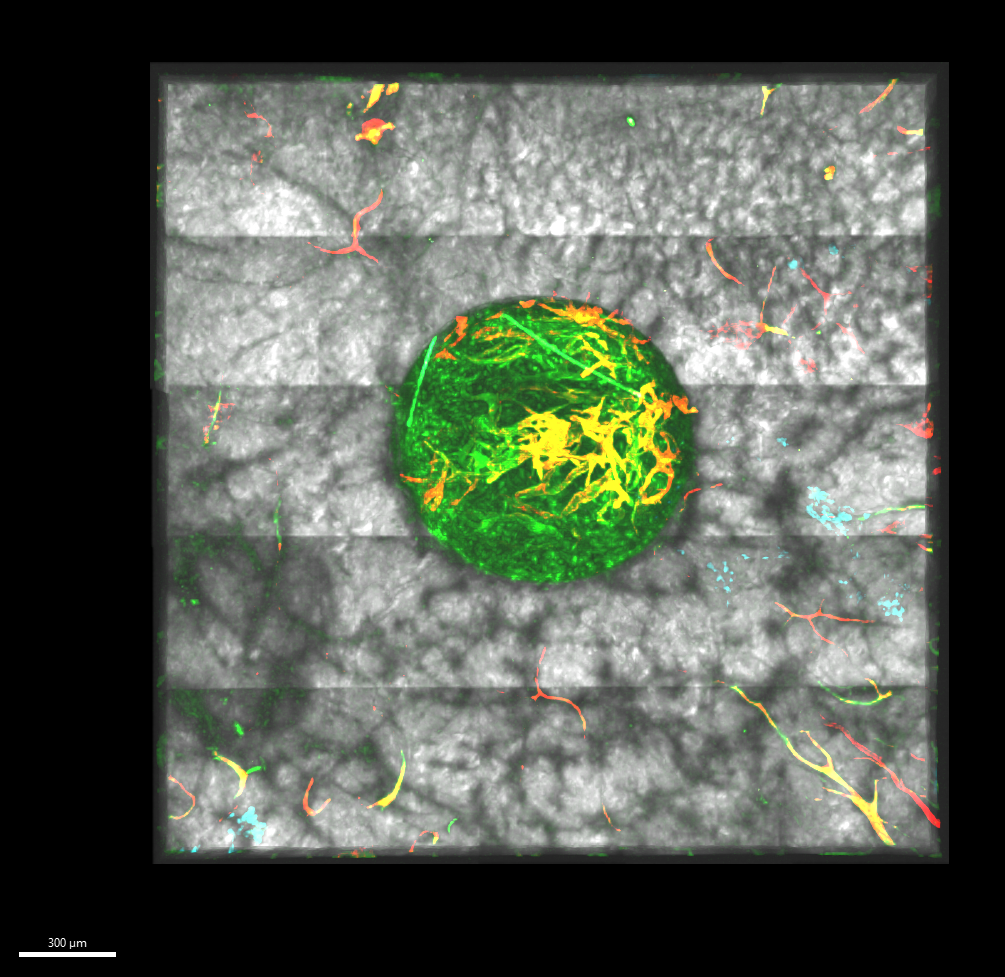

Supplement: Figure 1—source data 1. [file elife-83146-fig1-data1.zip › Figure 1/004 1mm day 3 top all slice endo cd31 gfp-shg good-2.tif]

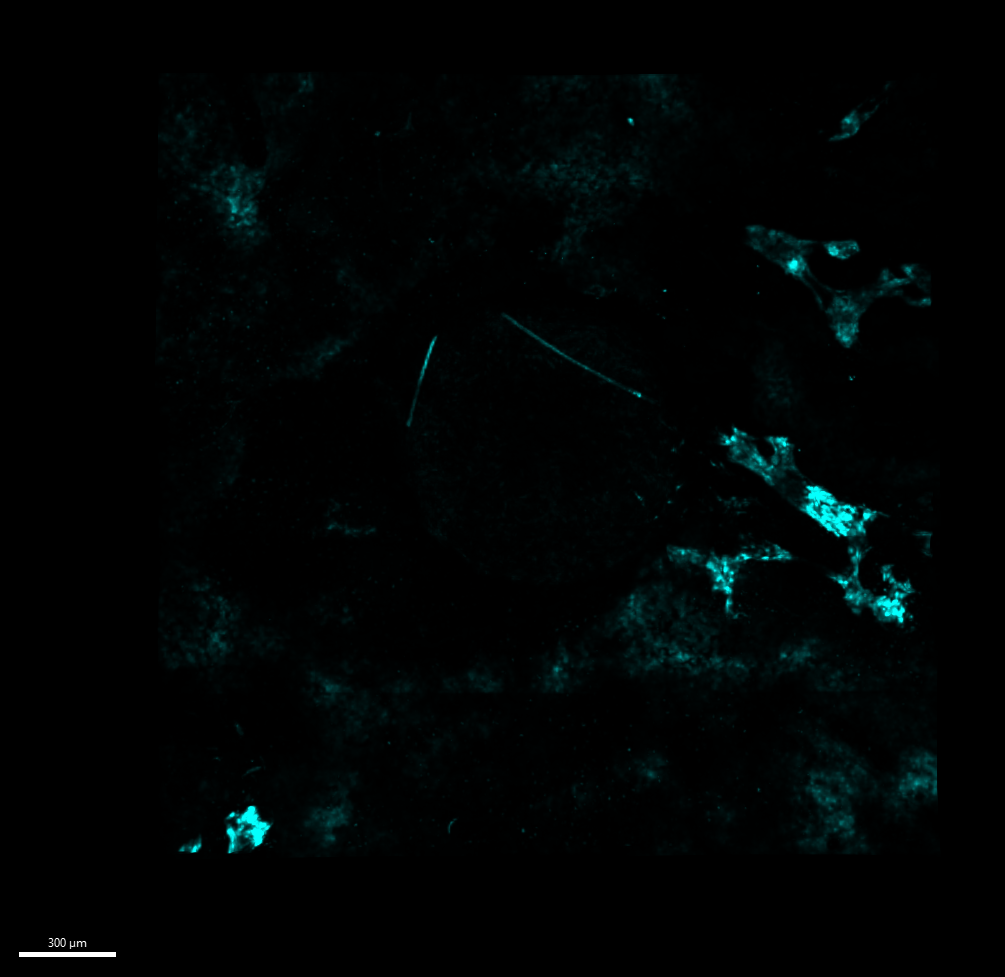

Supplement: Figure 1—source data 1. [file elife-83146-fig1-data1.zip › Figure 1/004 1mm day 3 top all slice gfp-good-2.tif]

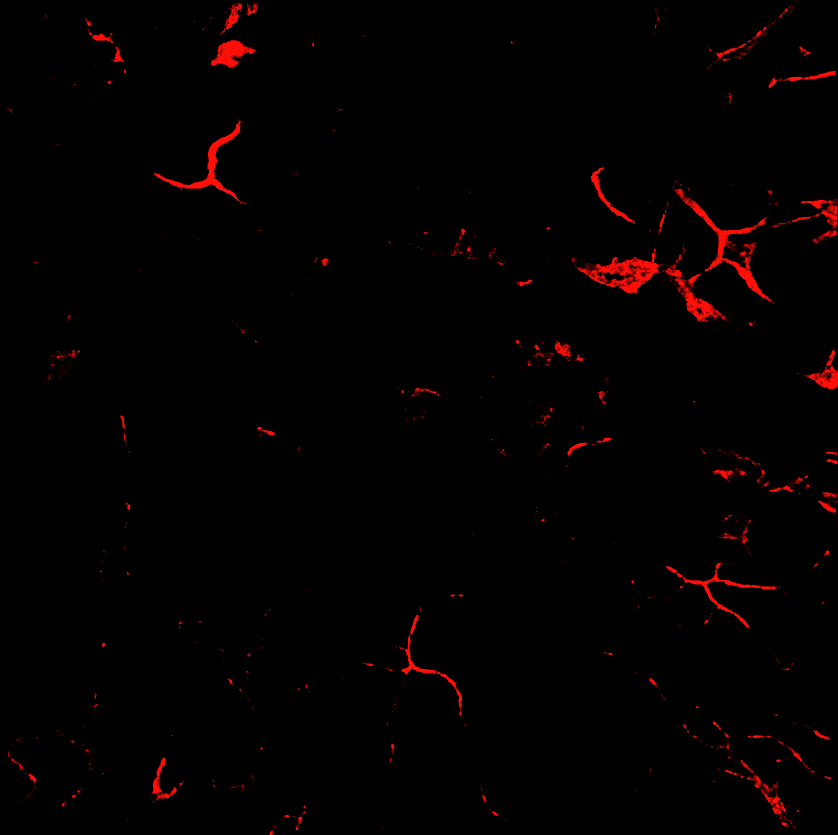

Supplement: Figure 1—source data 1. [file elife-83146-fig1-data1.zip › Figure 1/004 day 3 top cd31only-crop.tif]

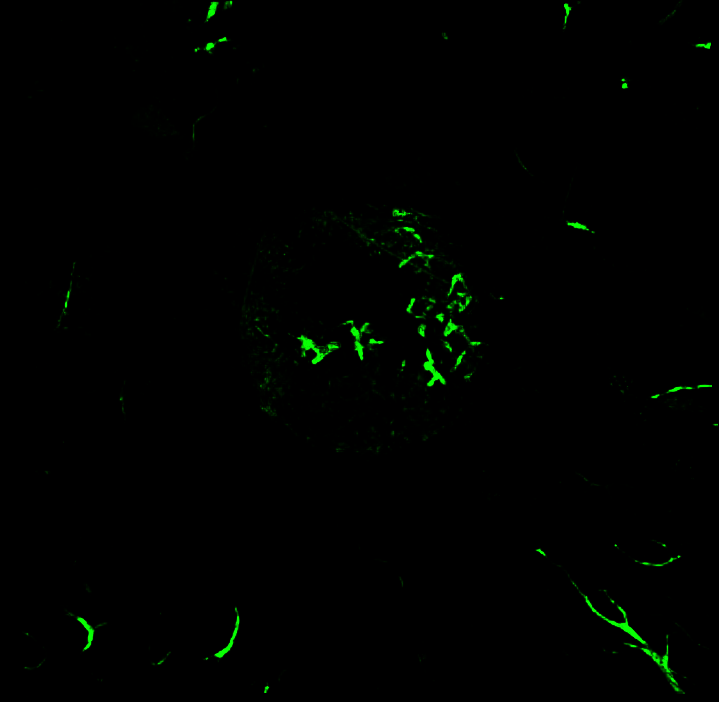

Supplement: Figure 1—source data 1. [file elife-83146-fig1-data1.zip › Figure 1/004 day 3 top endo.tif]

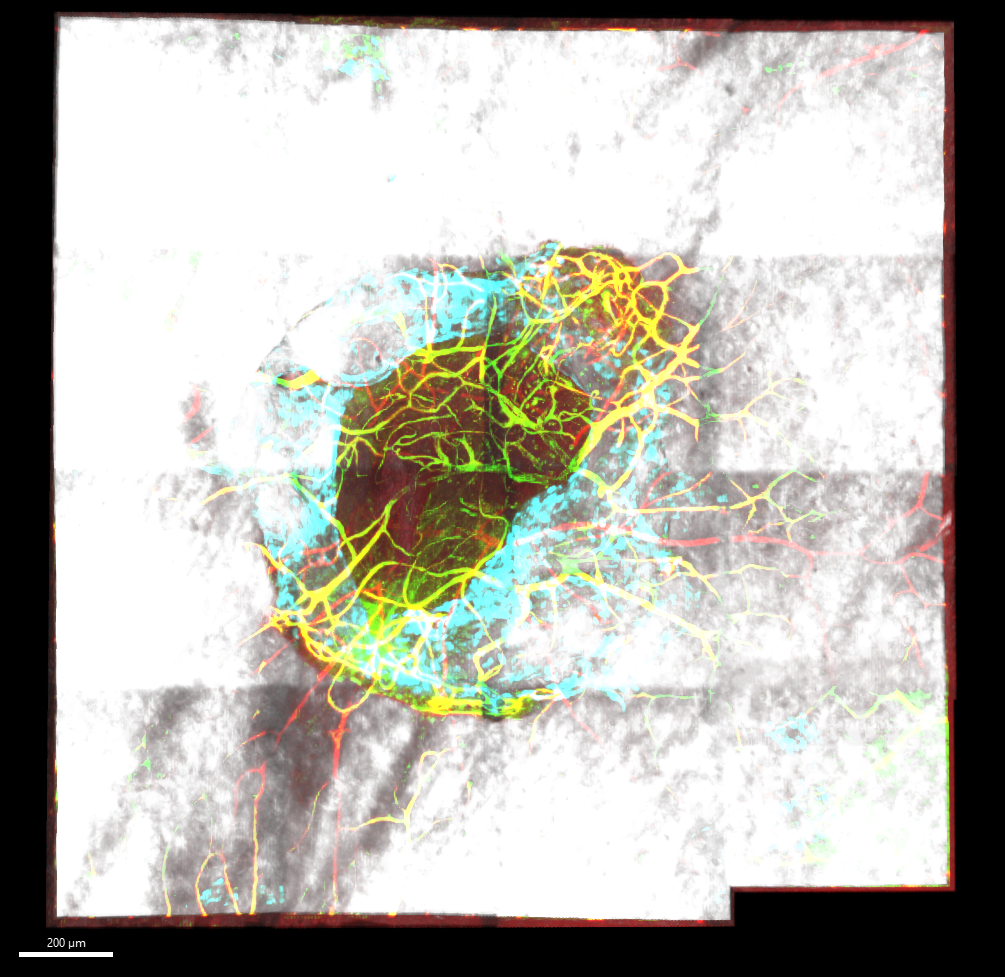

Supplement: Figure 1—source data 1. [file elife-83146-fig1-data1.zip › Figure 1/165 1mm day 21 side 2 flip cd31 endo gfp shg good.tif]

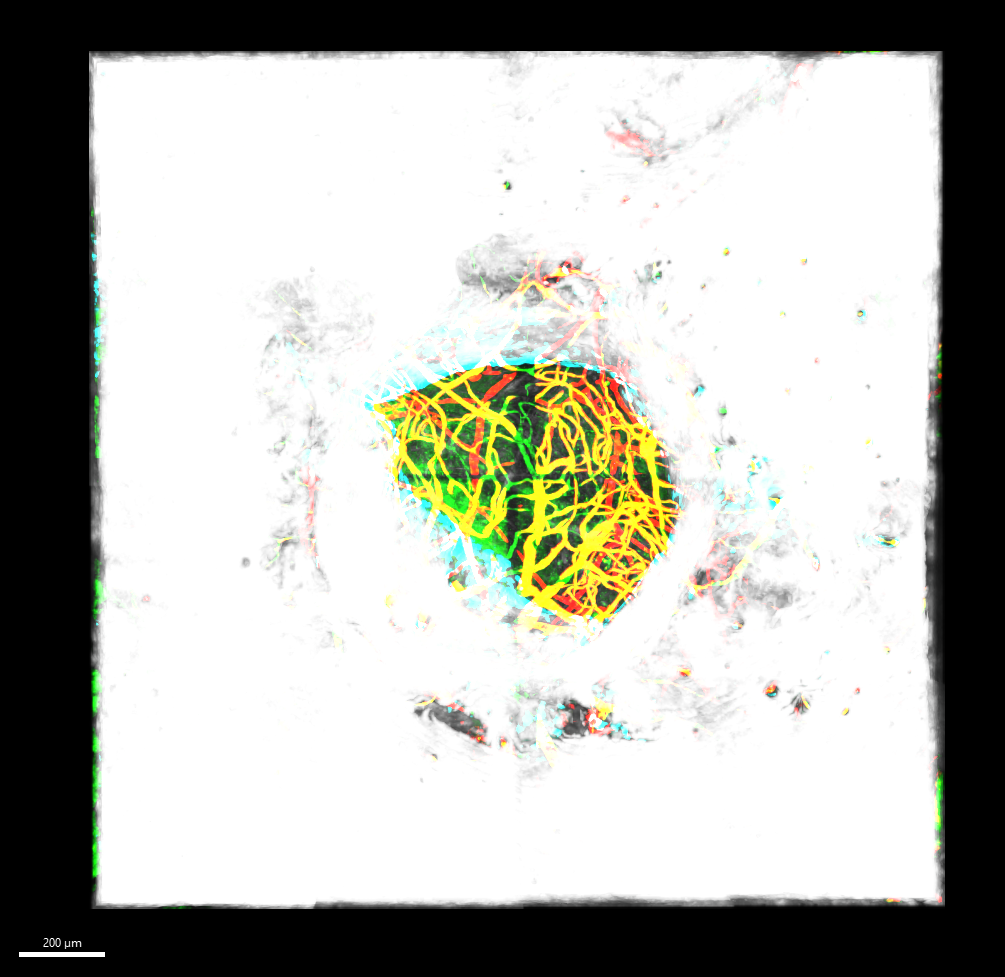

Supplement: Figure 1—source data 1. [file elife-83146-fig1-data1.zip › Figure 1/165 1mm day 21 side 2 flip cd31 endo gfp shg.tif]

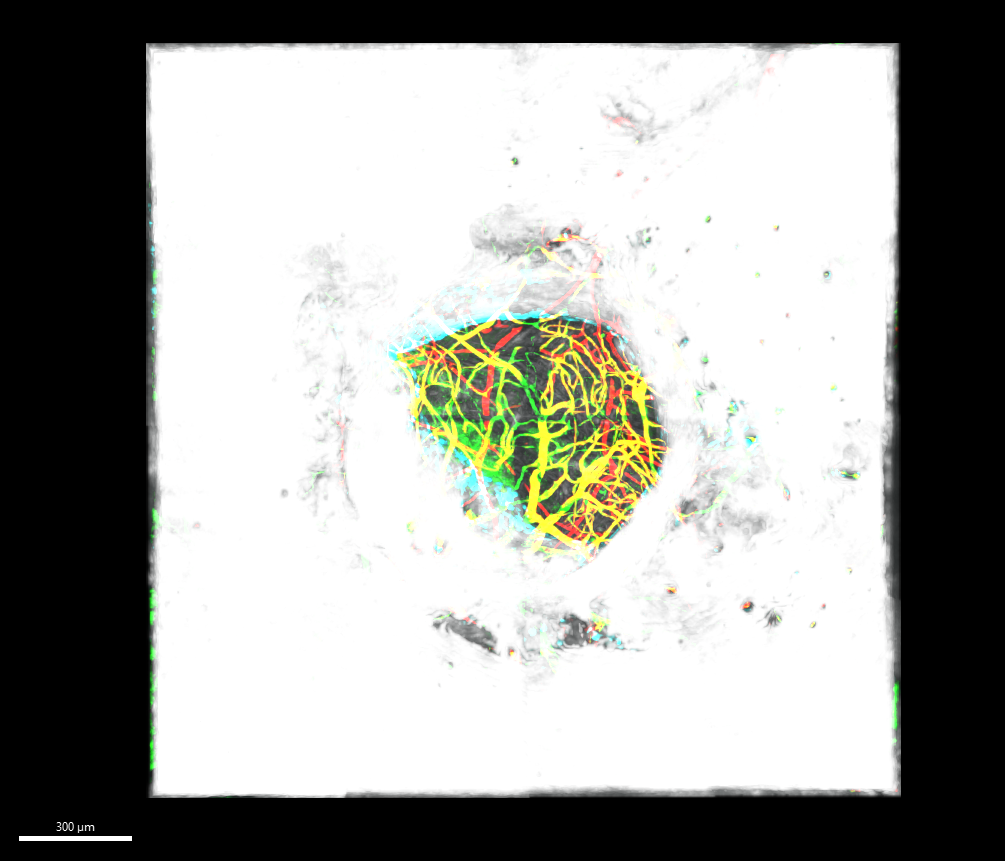

Supplement: Figure 1—source data 1. [file elife-83146-fig1-data1.zip › Figure 1/165 1mm day 21 side 2 flip cd31 endo gfp shg-2.tif]

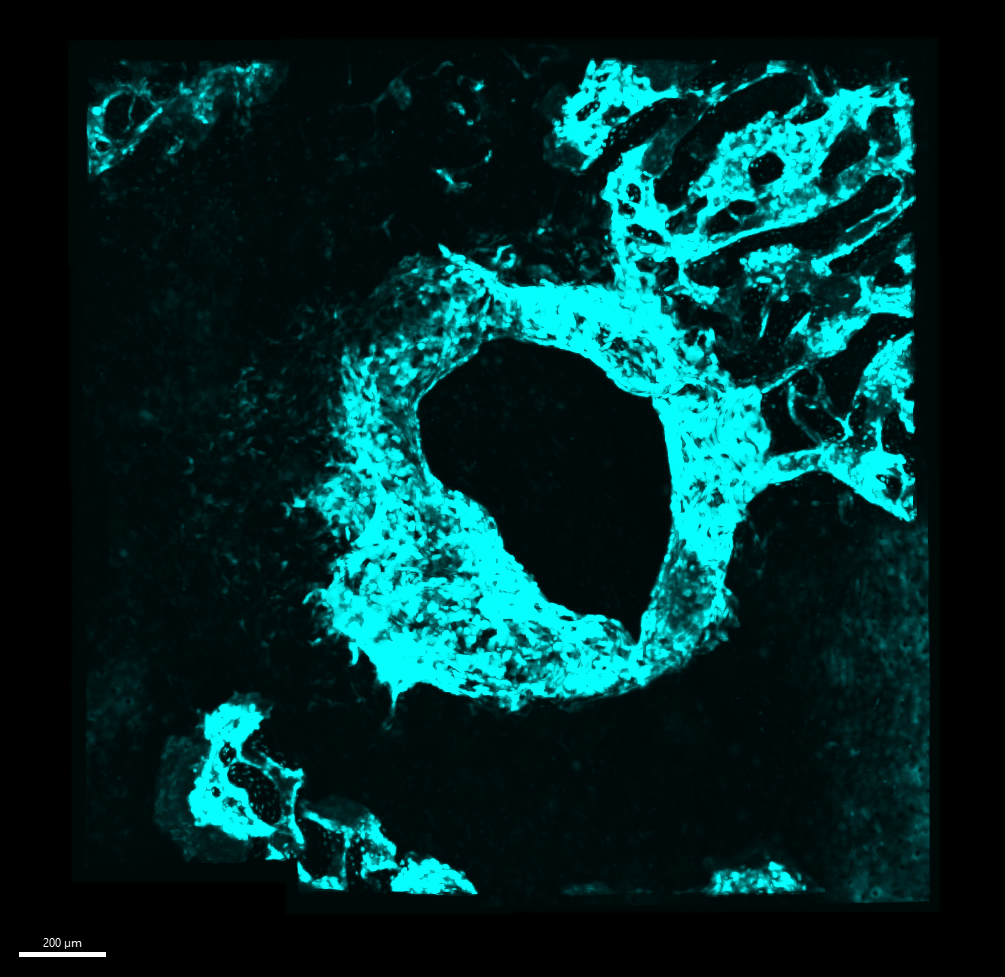

Supplement: Figure 1—source data 1. [file elife-83146-fig1-data1.zip › Figure 1/165 1mm day 21 side 2 flip gfp.tif]

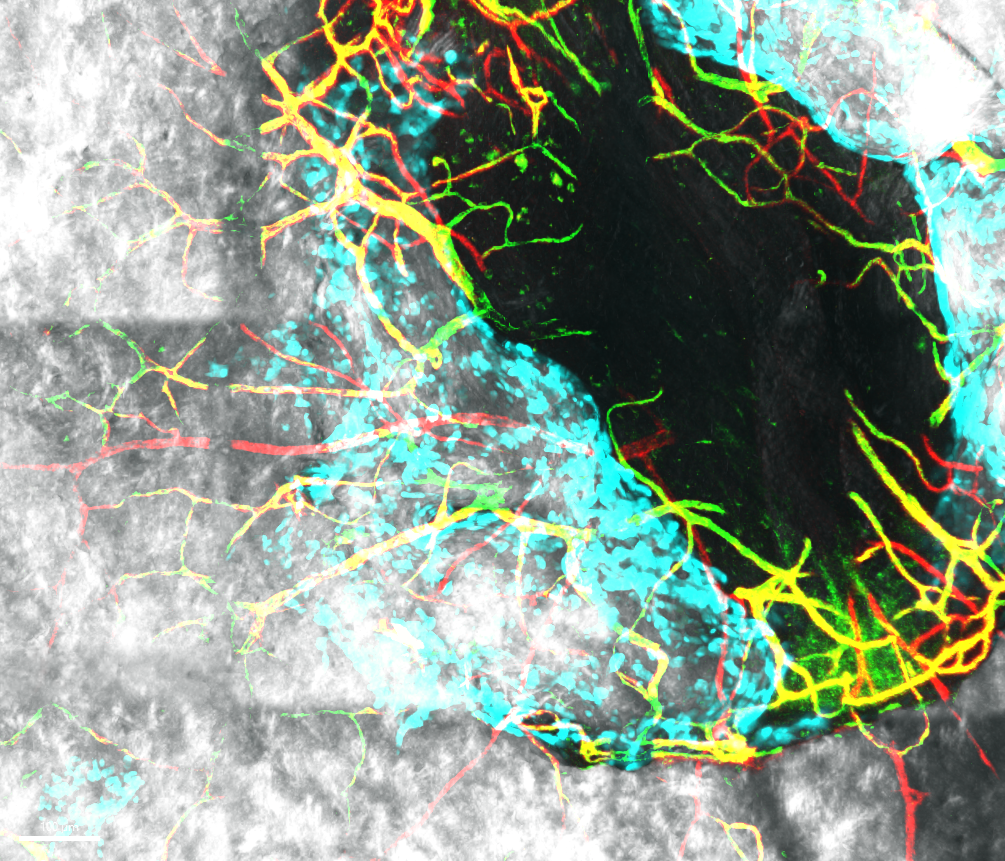

Supplement: Figure 1—source data 1. [file elife-83146-fig1-data1.zip › Figure 1/165 1mm day 21 side 2 top cd31 endo gfp shg zoom-3 - Copy.tif]

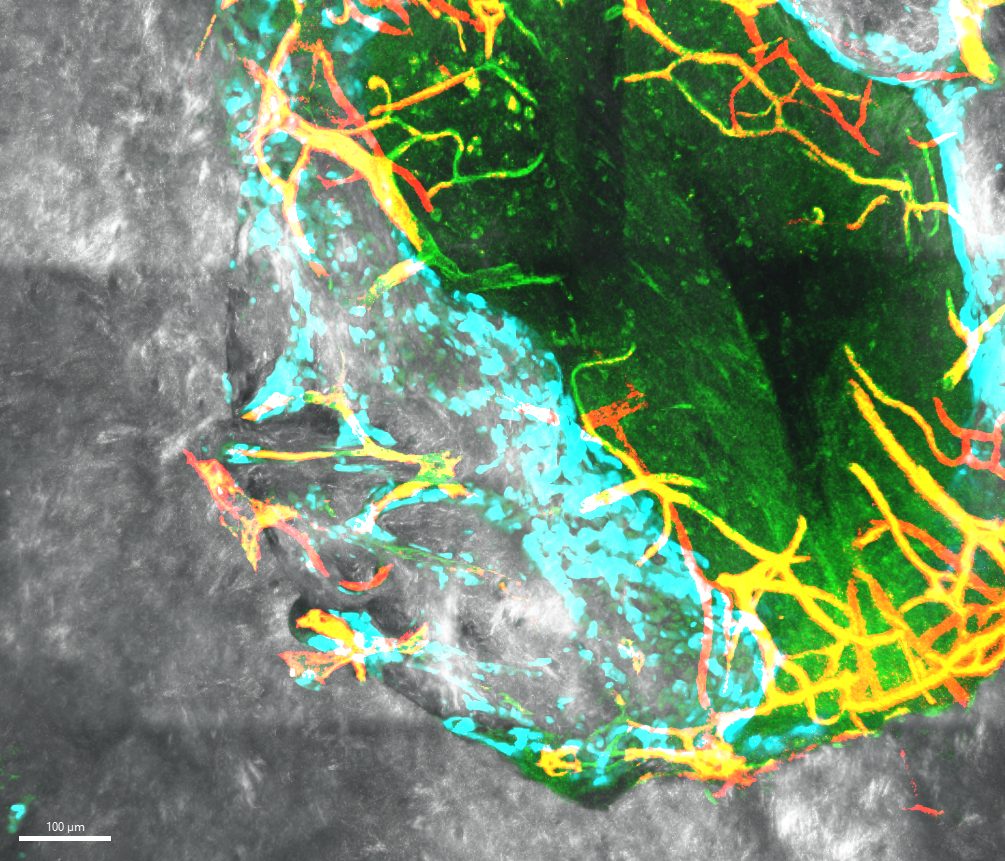

Supplement: Figure 1—source data 1. [file elife-83146-fig1-data1.zip › Figure 1/165 1mm day 21 side 2 top cd31 endo gfp shg zoom-2 - Copy.tif]

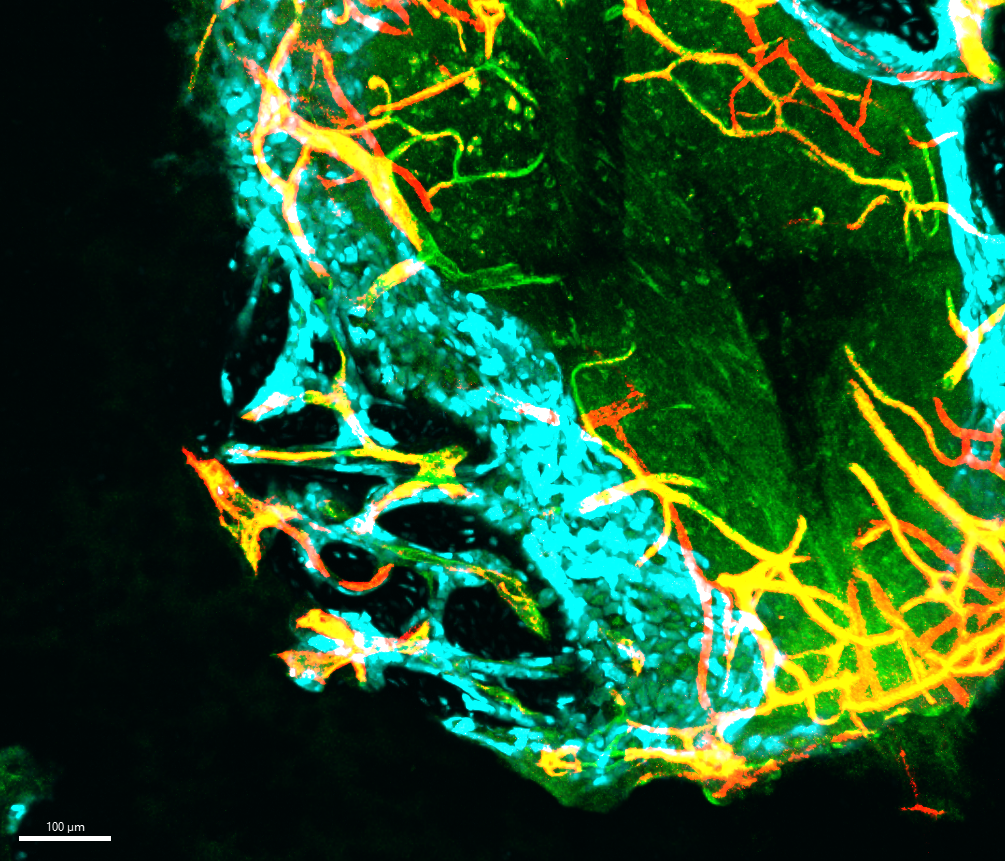

Supplement: Figure 1—source data 1. [file elife-83146-fig1-data1.zip › Figure 1/165 1mm day 21 side 2 top cd31 endo gfp zoom-2 - Copy.tif]

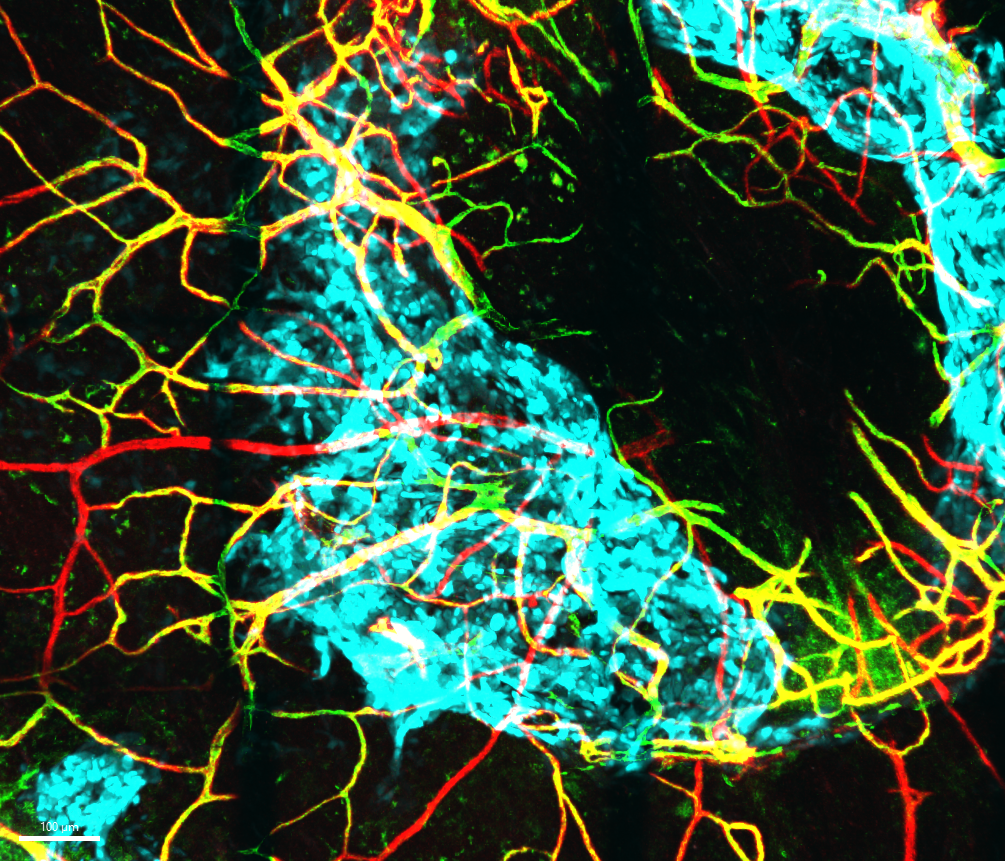

Supplement: Figure 1—source data 1. [file elife-83146-fig1-data1.zip › Figure 1/165 1mm day 21 side 2 top cd31 endo gfp zoom-3 - Copy.tif]

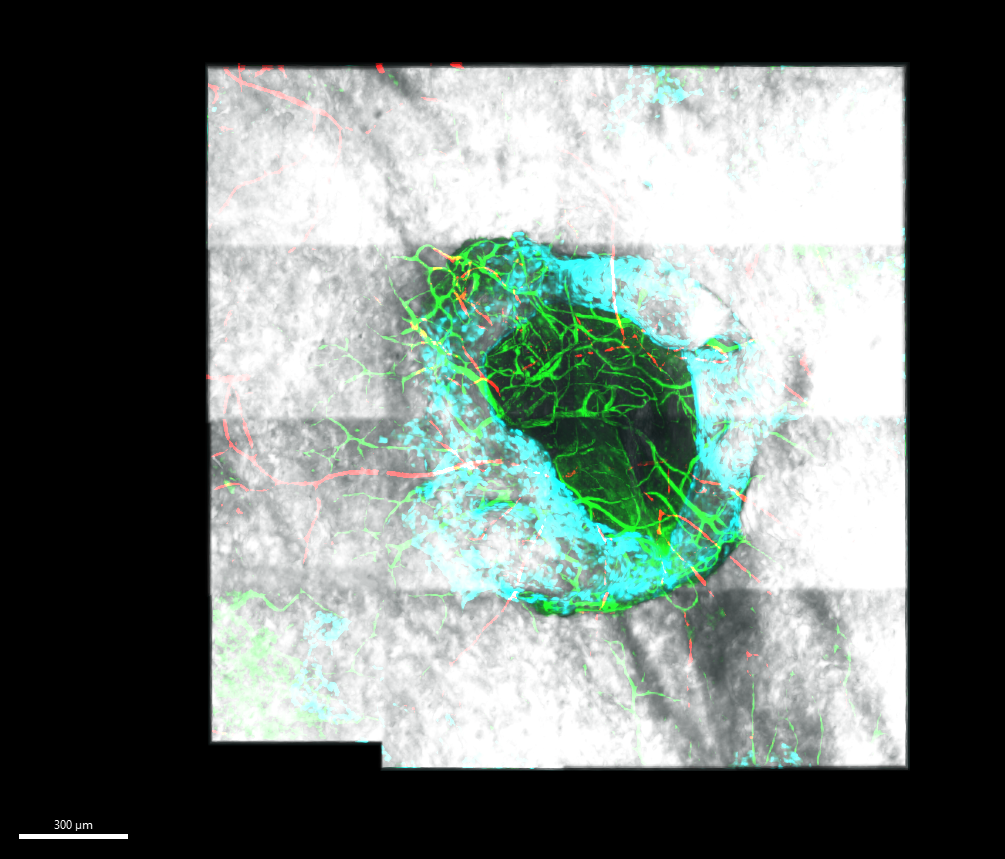

Supplement: Figure 1—source data 1. [file elife-83146-fig1-data1.zip › Figure 1/165 1mm day 21 side 2 top cd31 only endo gfp shg.tif]

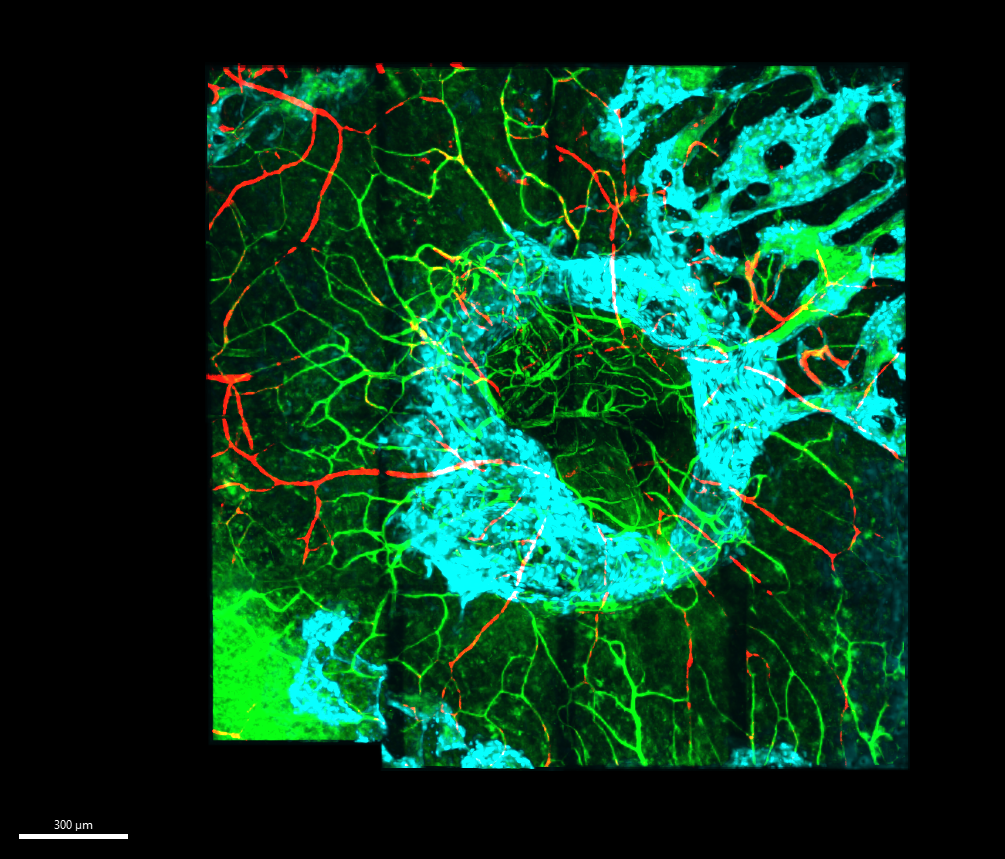

Supplement: Figure 1—source data 1. [file elife-83146-fig1-data1.zip › Figure 1/165 1mm day 21 side 2 top cd31 only endo gfp.tif]

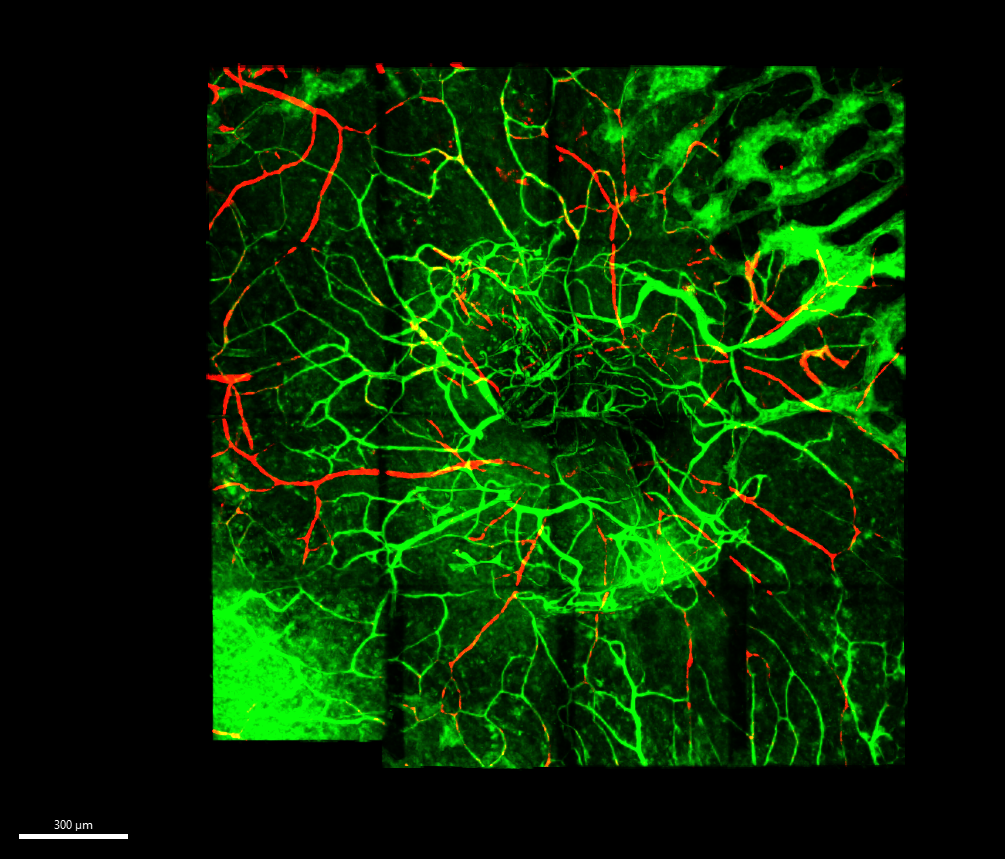

Supplement: Figure 1—source data 1. [file elife-83146-fig1-data1.zip › Figure 1/165 1mm day 21 side 2 top cd31 only endo.tif]

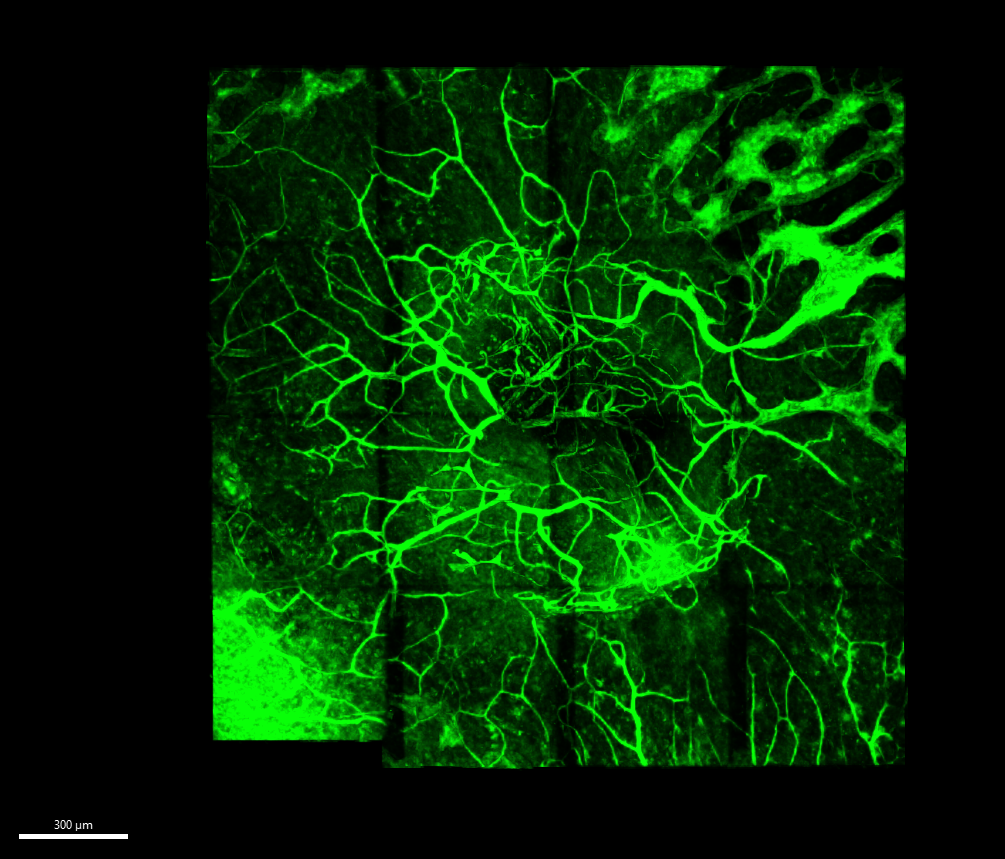

Supplement: Figure 1—source data 1. [file elife-83146-fig1-data1.zip › Figure 1/165 1mm day 21 side 2 top endo.tif]

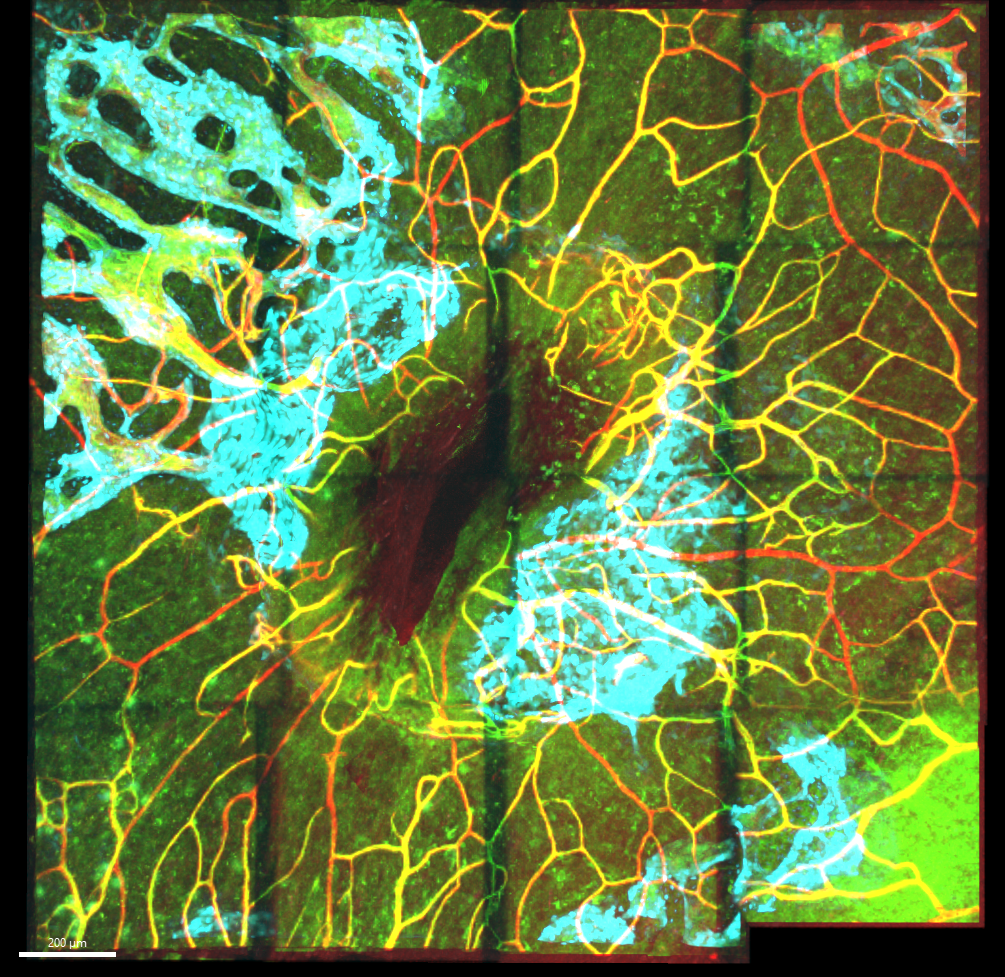

Supplement: Figure 1—source data 1. [file elife-83146-fig1-data1.zip › Figure 1/165 1mm day 21 side 2 top slice 28 cd31 endo gfp.tif]

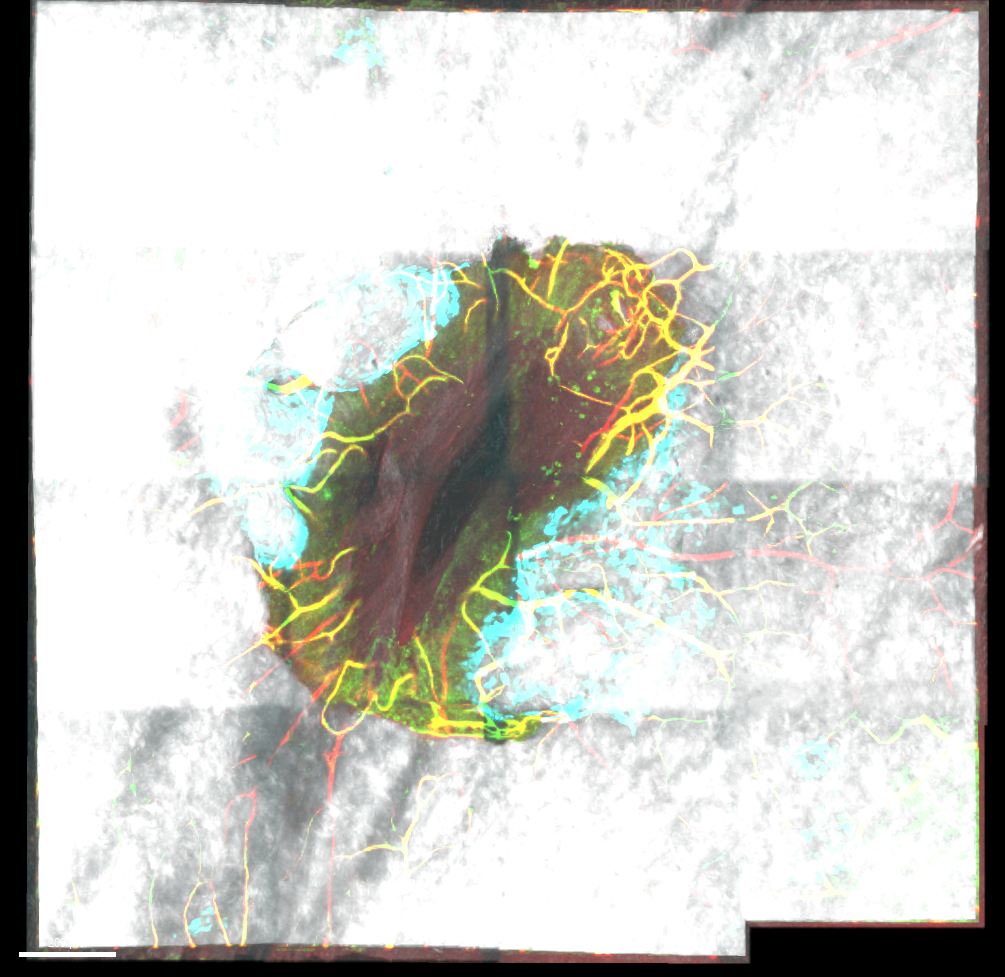

Supplement: Figure 1—source data 1. [file elife-83146-fig1-data1.zip › Figure 1/165 1mm day 21 side 2 top slice 28 cd31 endo gfp-2 shg.tif]

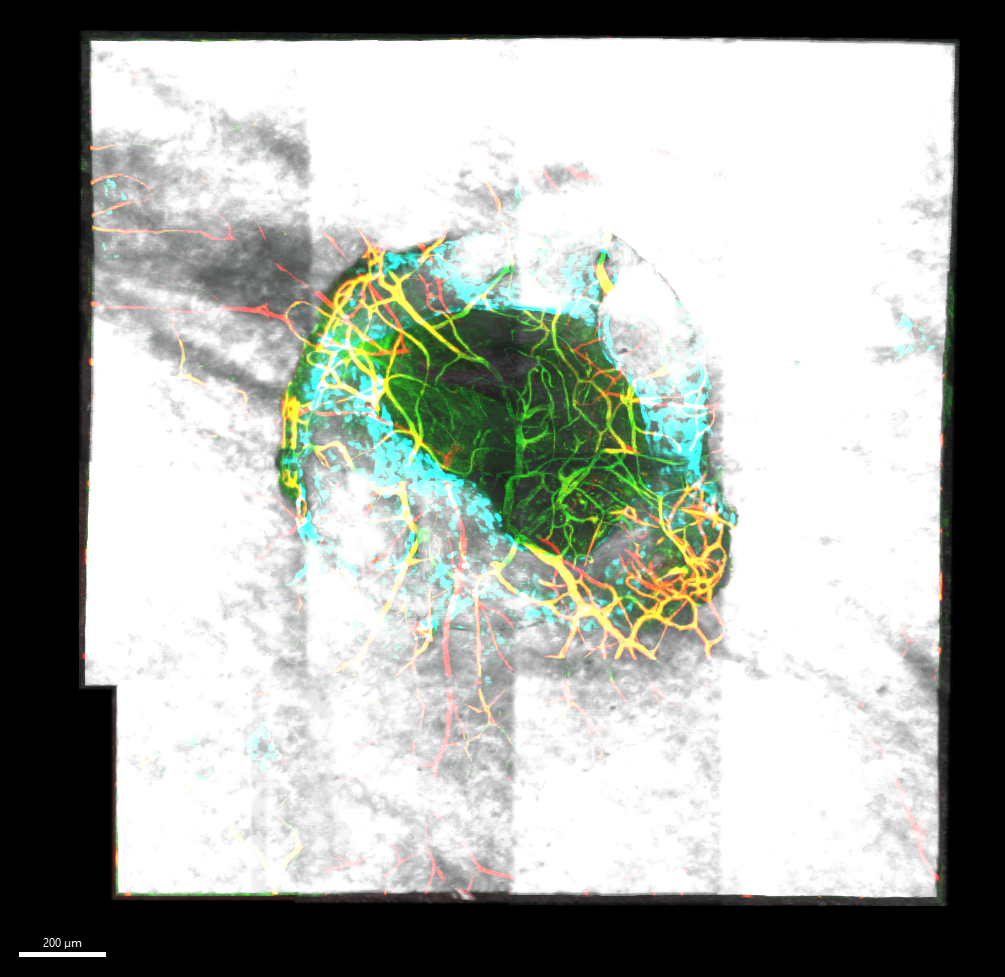

Supplement: Figure 1—source data 1. [file elife-83146-fig1-data1.zip › Figure 1/165 1mm day 21 side 2 top cd31 endo gfp shg.tif]

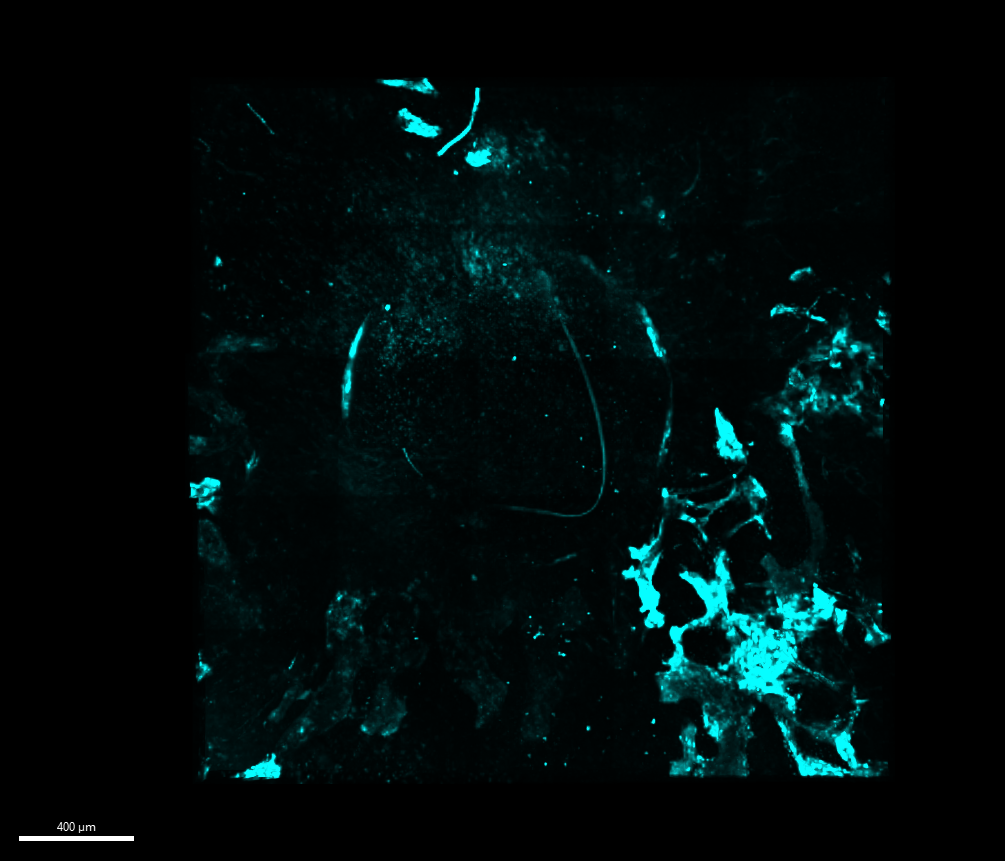

Supplement: Figure 1—source data 1. [file elife-83146-fig1-data1.zip › Figure 1/909 day 10 1mm top side 34 sliced gfp new.tif]

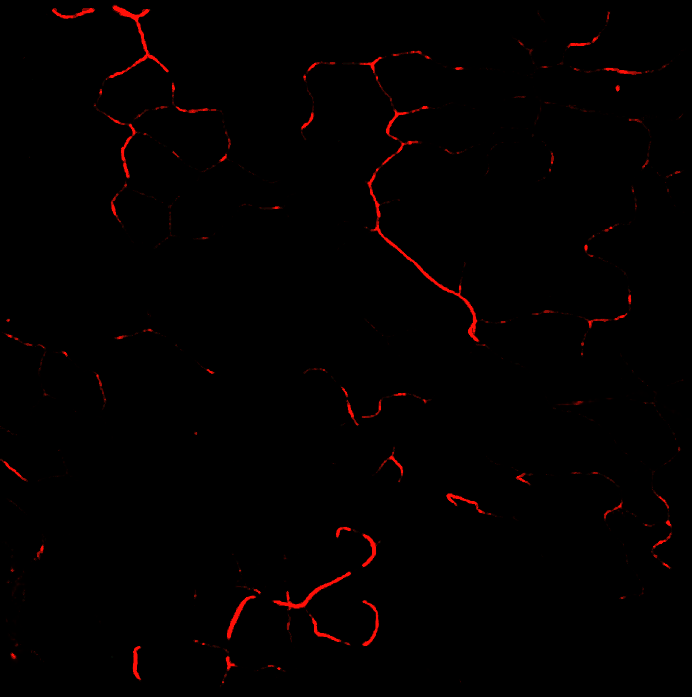

Supplement: Figure 1—source data 1. [file elife-83146-fig1-data1.zip › Figure 1/no injury top cd31only crop final.tif]

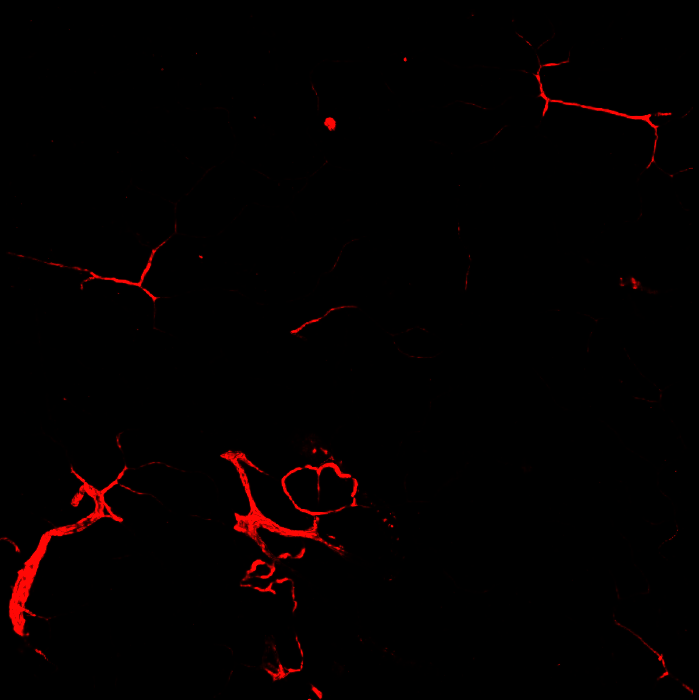

Supplement: Figure 1—source data 1. [file elife-83146-fig1-data1.zip › Figure 1/no injury top cd31only crop.tif]

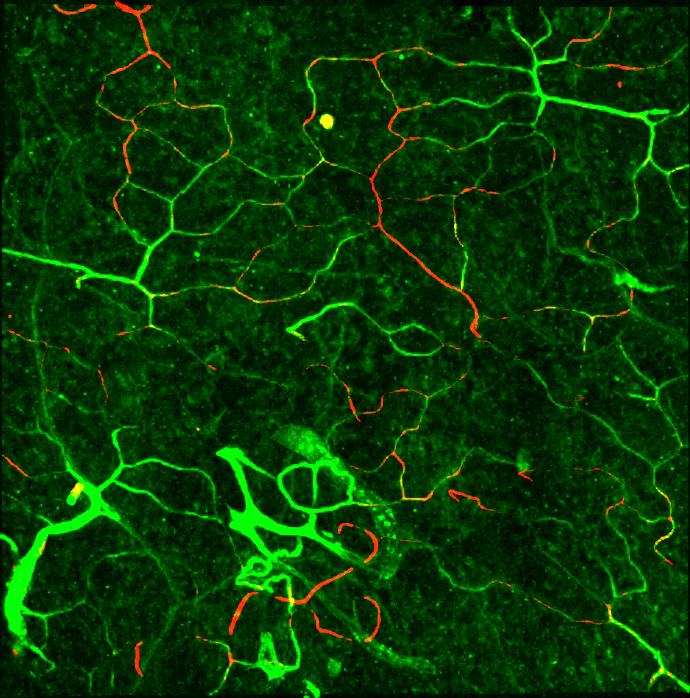

Supplement: Figure 1—source data 1. [file elife-83146-fig1-data1.zip › Figure 1/no injury top cd31only endo crop final.tif]

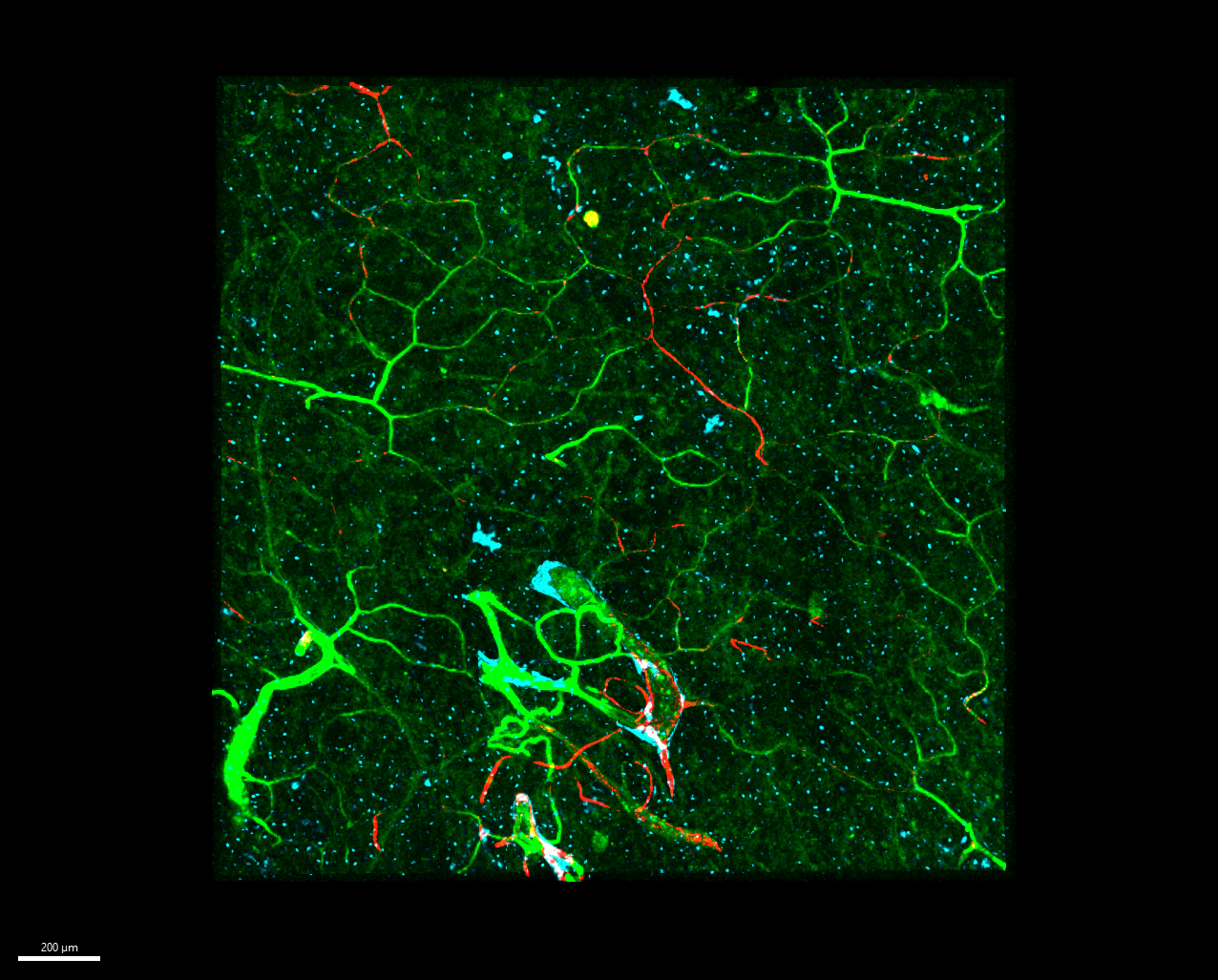

Supplement: Figure 1—source data 1. [file elife-83146-fig1-data1.zip › Figure 1/no injury top endo cd31only gfp no shg-2.tif]

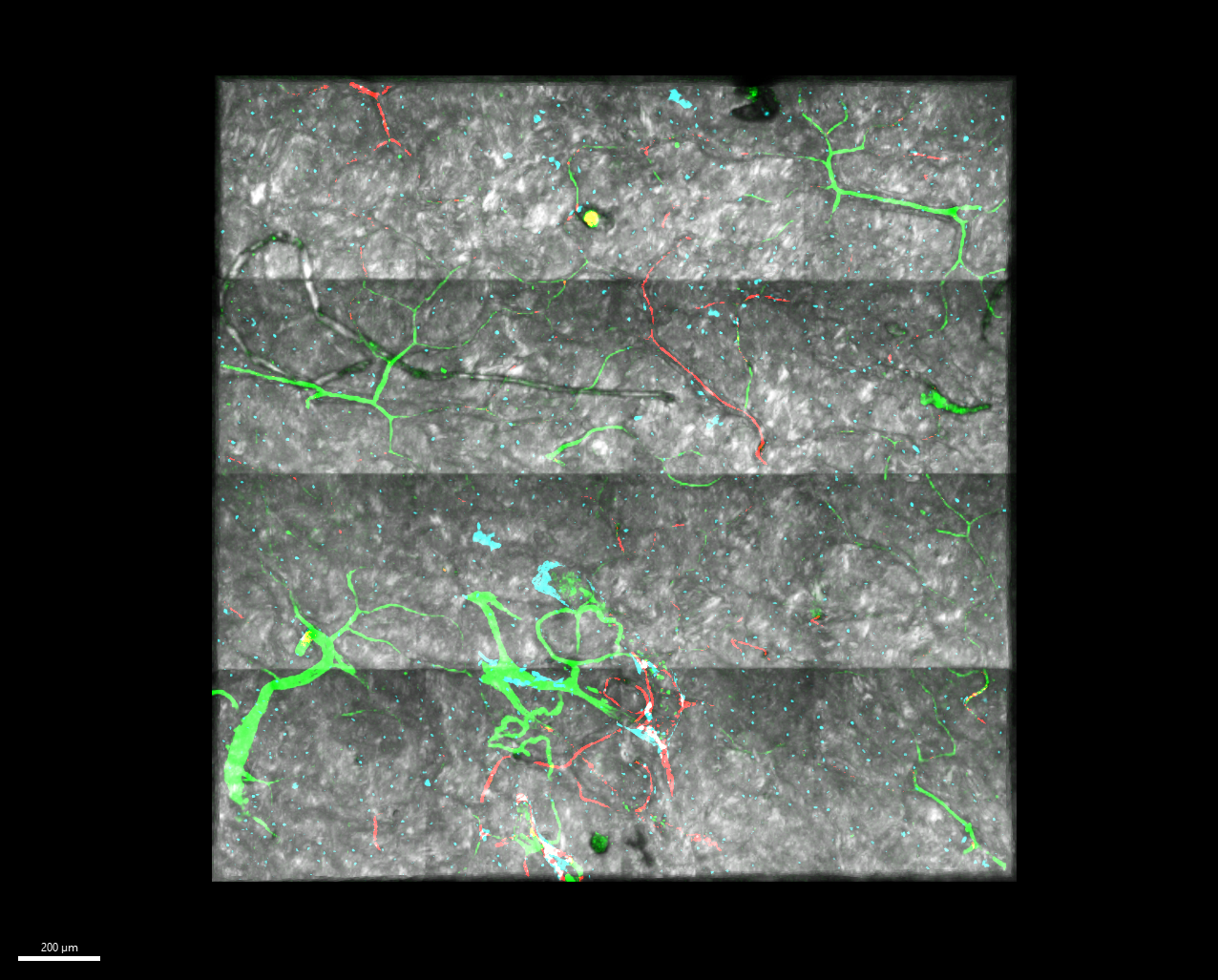

Supplement: Figure 1—source data 1. [file elife-83146-fig1-data1.zip › Figure 1/no injury top endo cd31only gfp.tif]

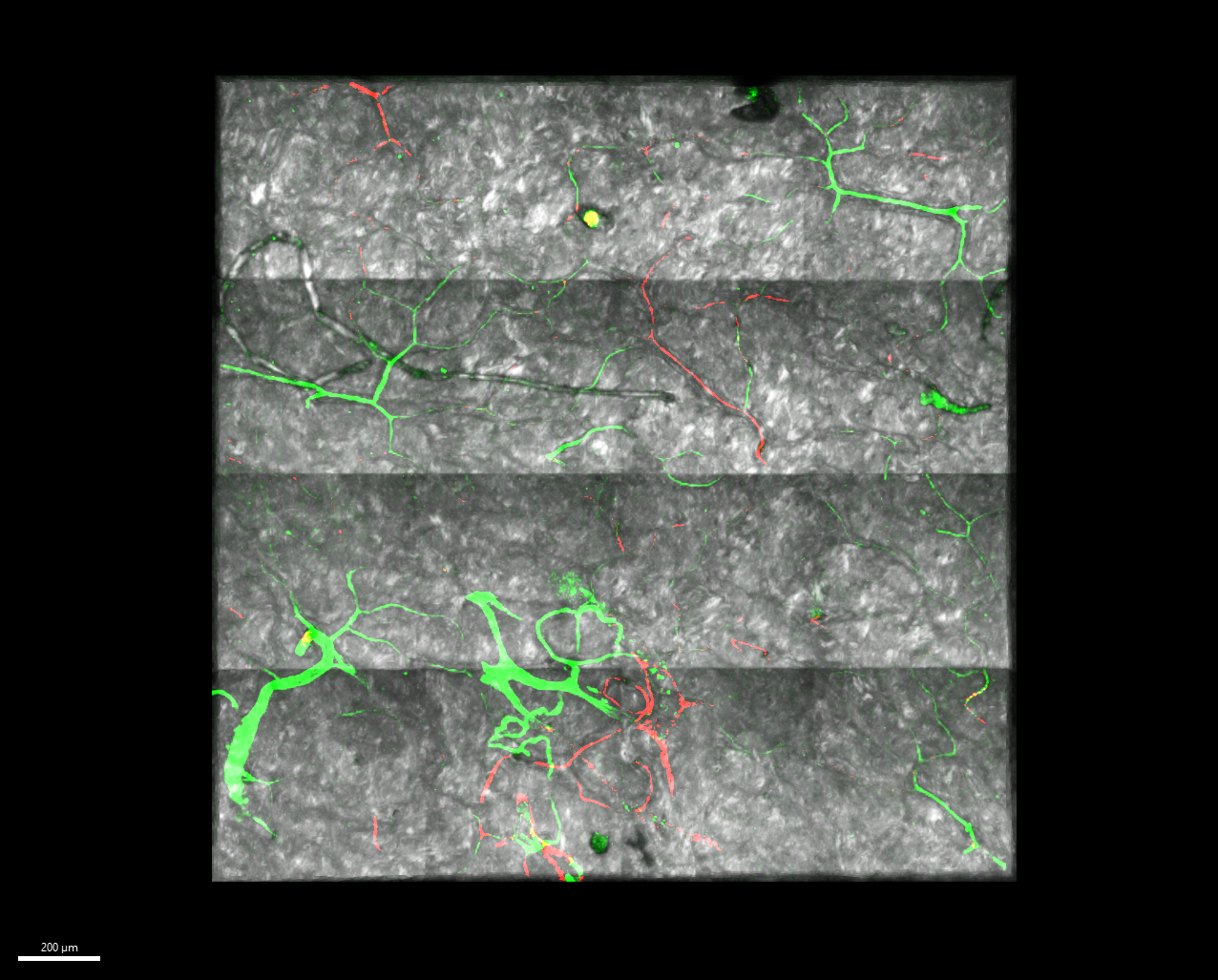

Supplement: Figure 1—source data 1. [file elife-83146-fig1-data1.zip › Figure 1/no injury top endo cd31only.tif]

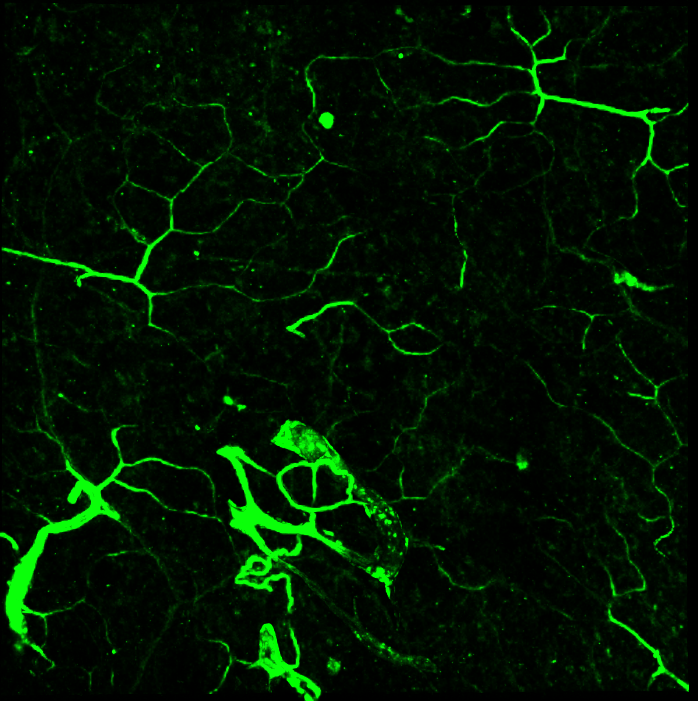

Supplement: Figure 1—source data 1. [file elife-83146-fig1-data1.zip › Figure 1/no injury top endo.tif]

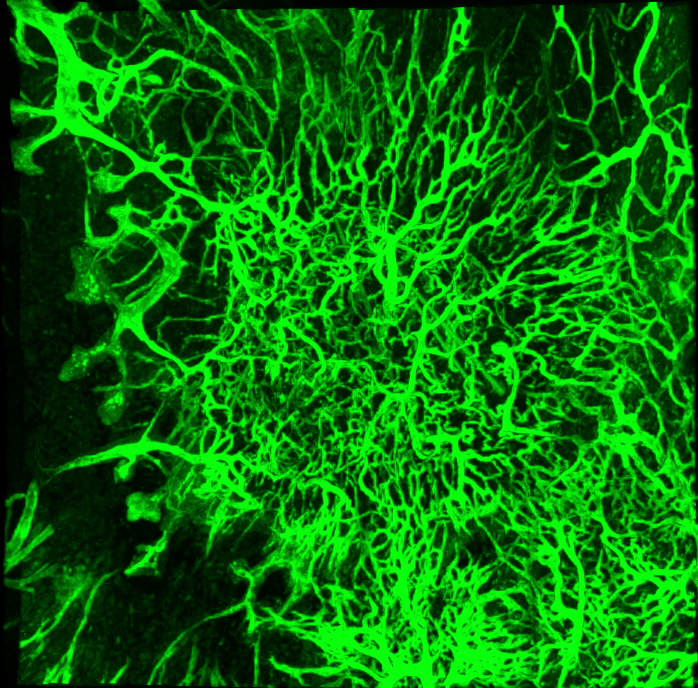

Supplement: Figure 1—source data 1. [file elife-83146-fig1-data1.zip › Figure 1/RE013 day 14 side 1 top side endo.tif]

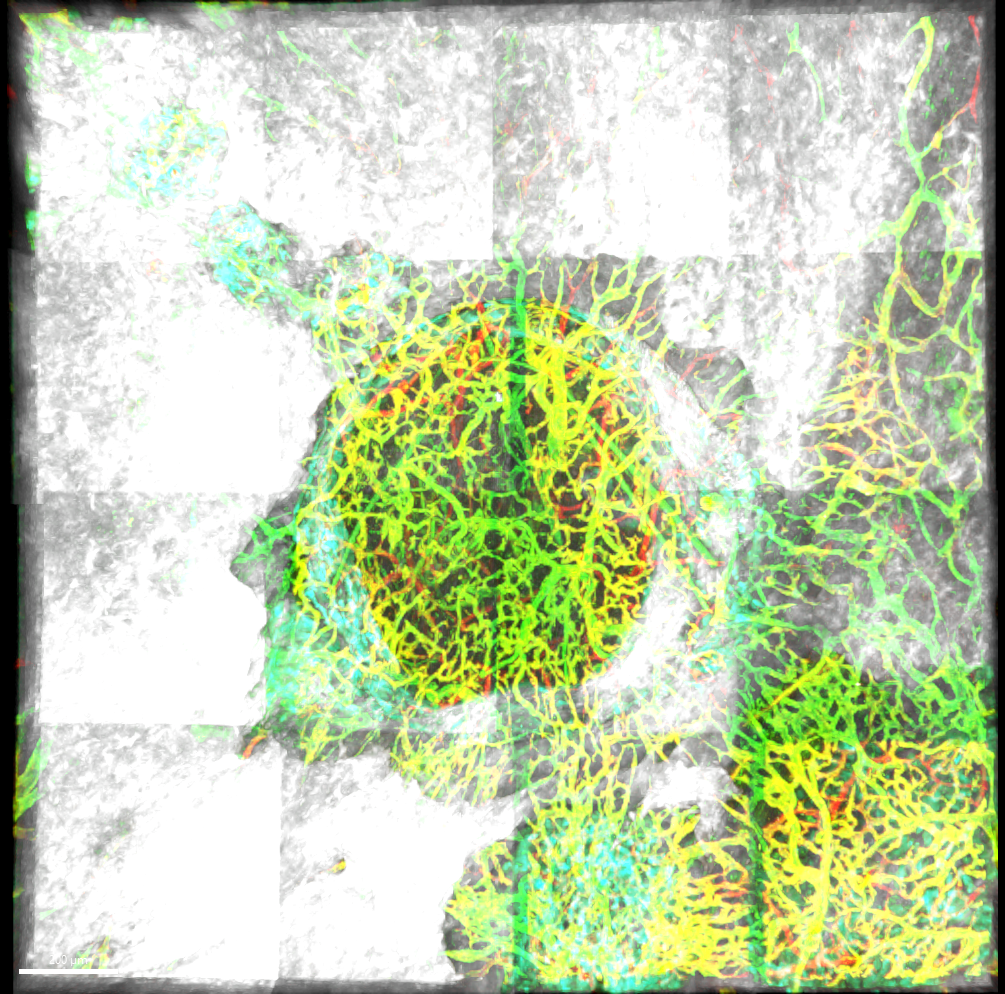

Supplement: Figure 1—source data 1. [file elife-83146-fig1-data1.zip › Figure 1/RE013 day 14 side 1 top 39 slices endo cd31 gfp shg.tif]

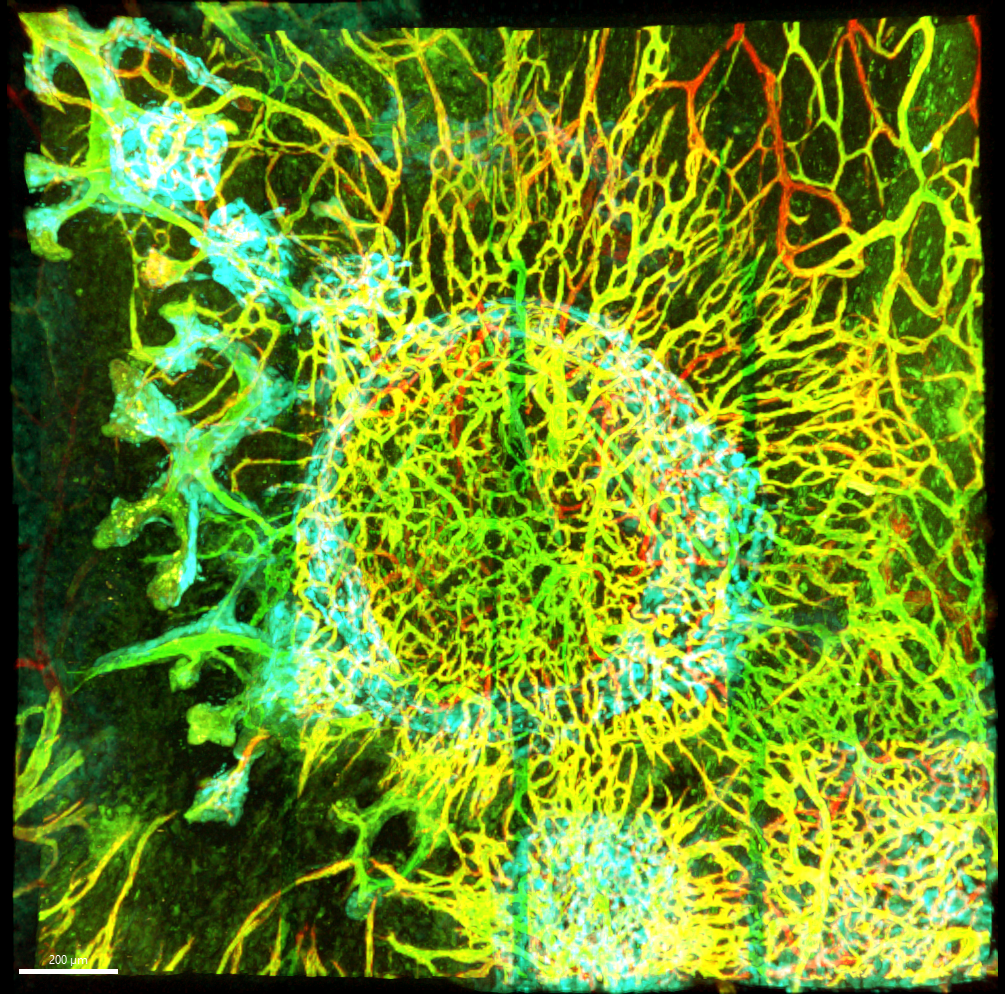

Supplement: Figure 1—source data 1. [file elife-83146-fig1-data1.zip › Figure 1/RE013 day 14 side 1 top 39 slices endo cd31 gfp.tif]

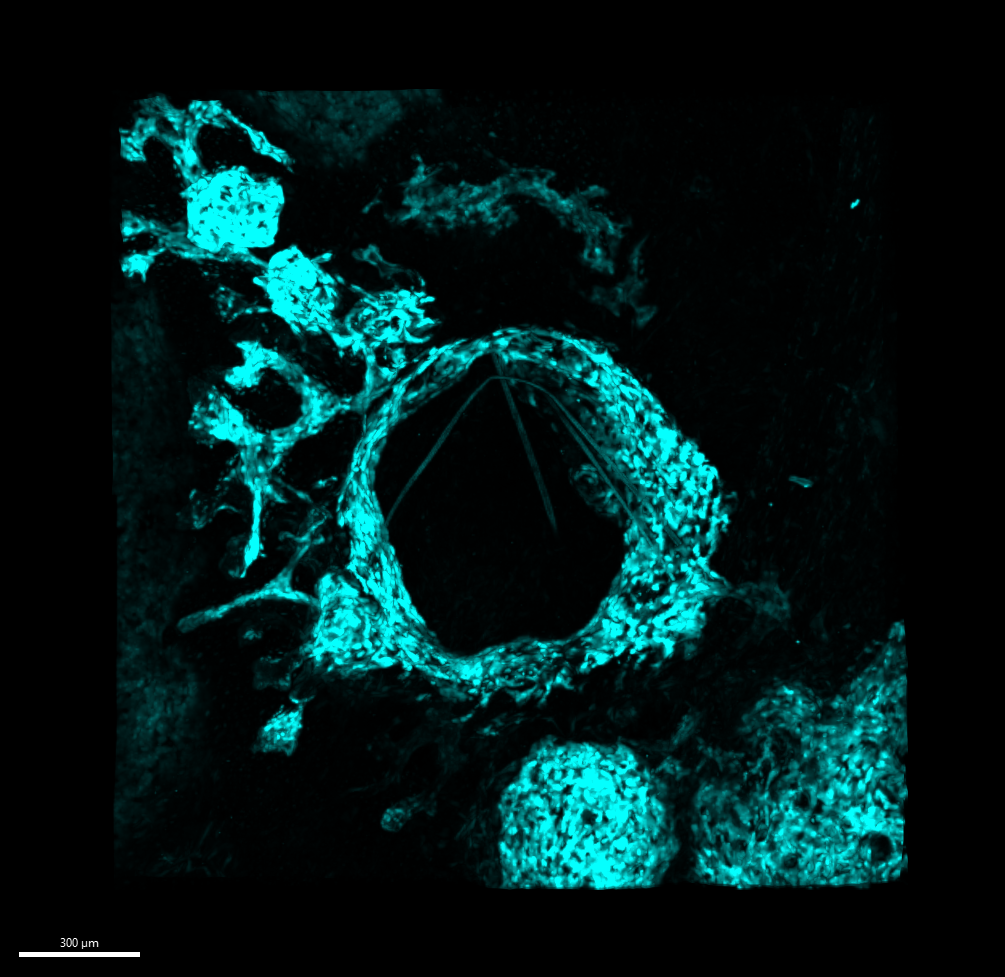

Supplement: Figure 1—source data 1. [file elife-83146-fig1-data1.zip › Figure 1/RE013 day 14 side 1 top 39 slices gfp.tif]

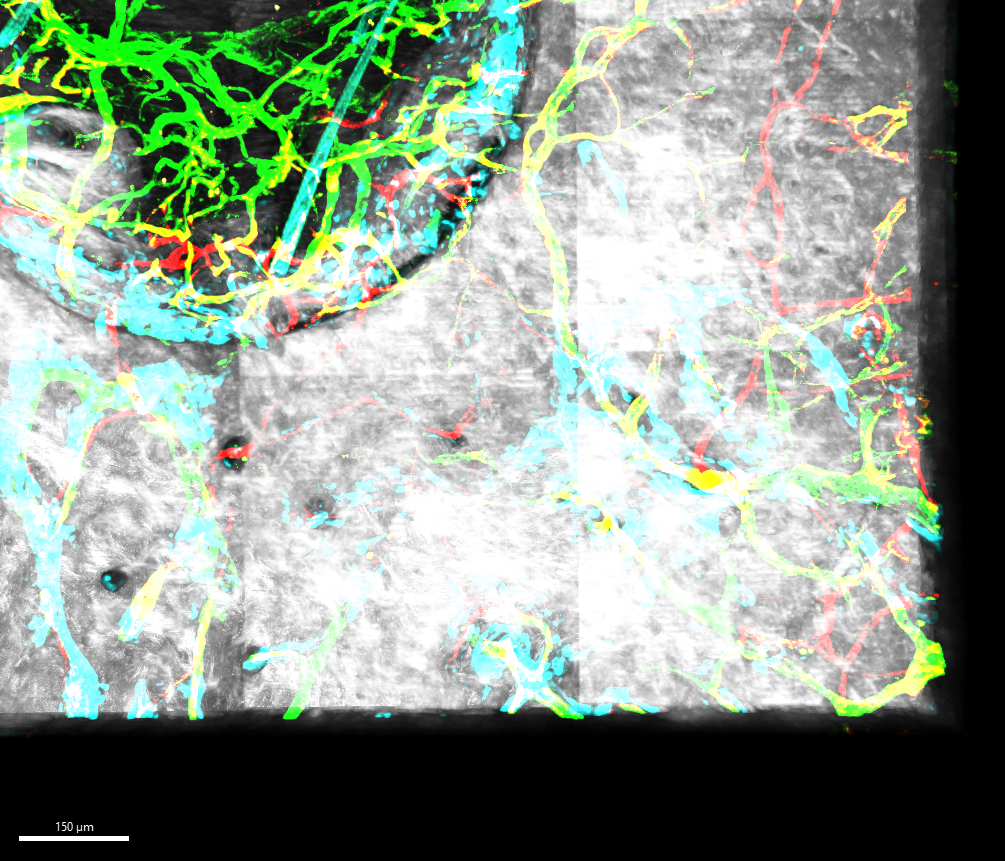

Supplement: Figure 1—source data 1. [file elife-83146-fig1-data1.zip › Figure 1/RE013 day 14 top side 2 gfp cd31 endo shg zoom 1 - Copy.tif]

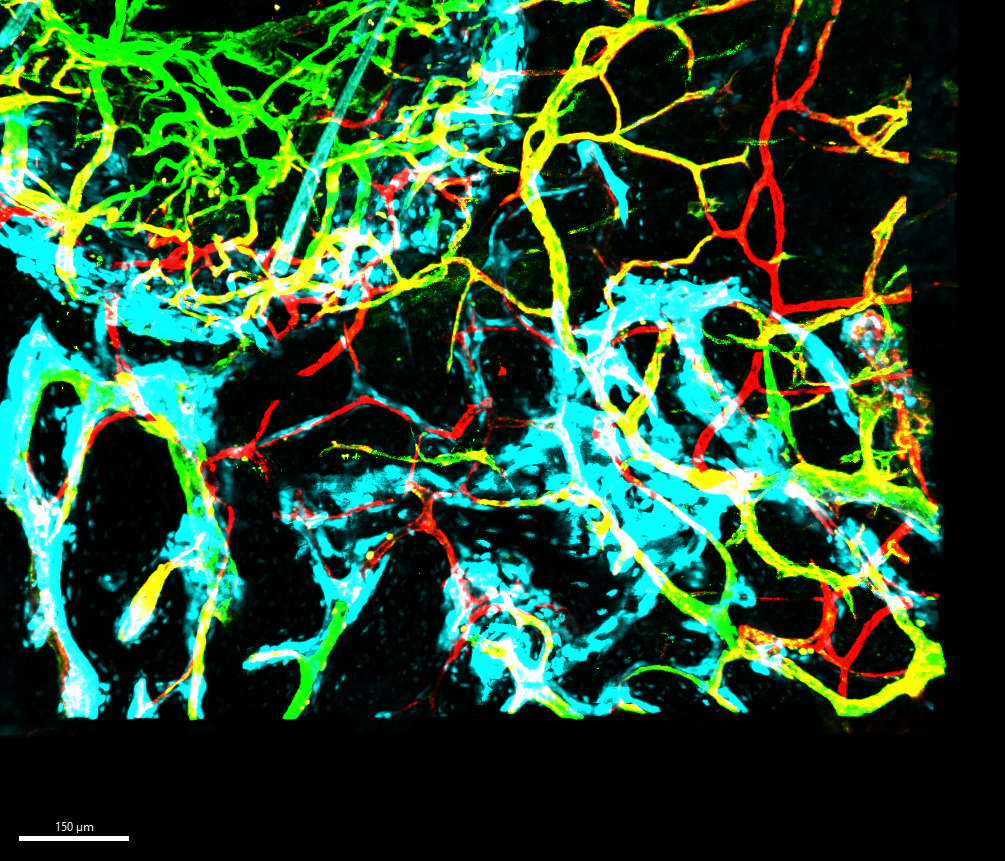

Supplement: Figure 1—source data 1. [file elife-83146-fig1-data1.zip › Figure 1/RE013 day 14 top side 2 gfp cd31 endo zoom 1 - Copy.tif]

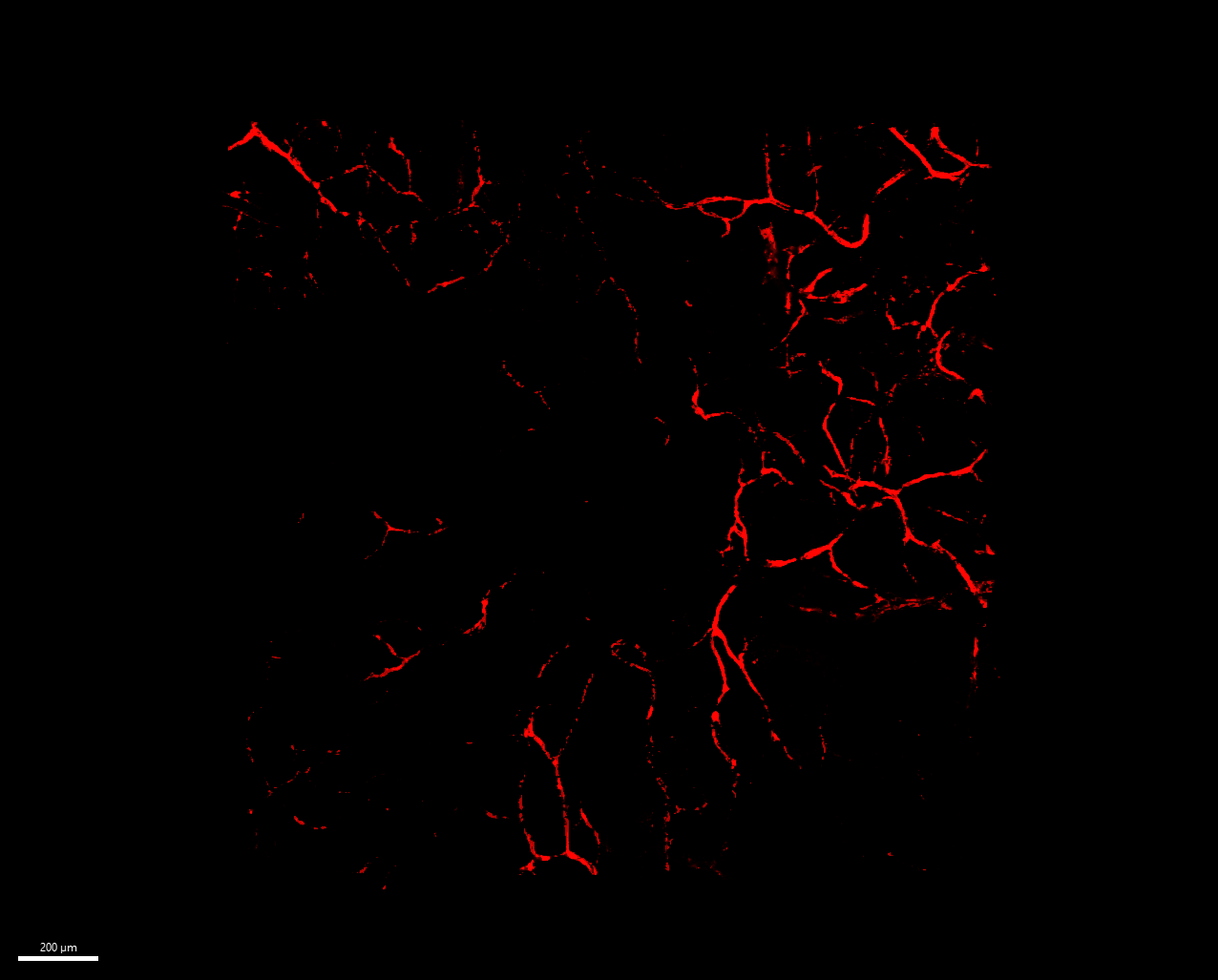

Supplement: Figure 1—source data 1. [file elife-83146-fig1-data1.zip › Figure 1/RE013 day 14 top side cd31 only - Copy.tif]

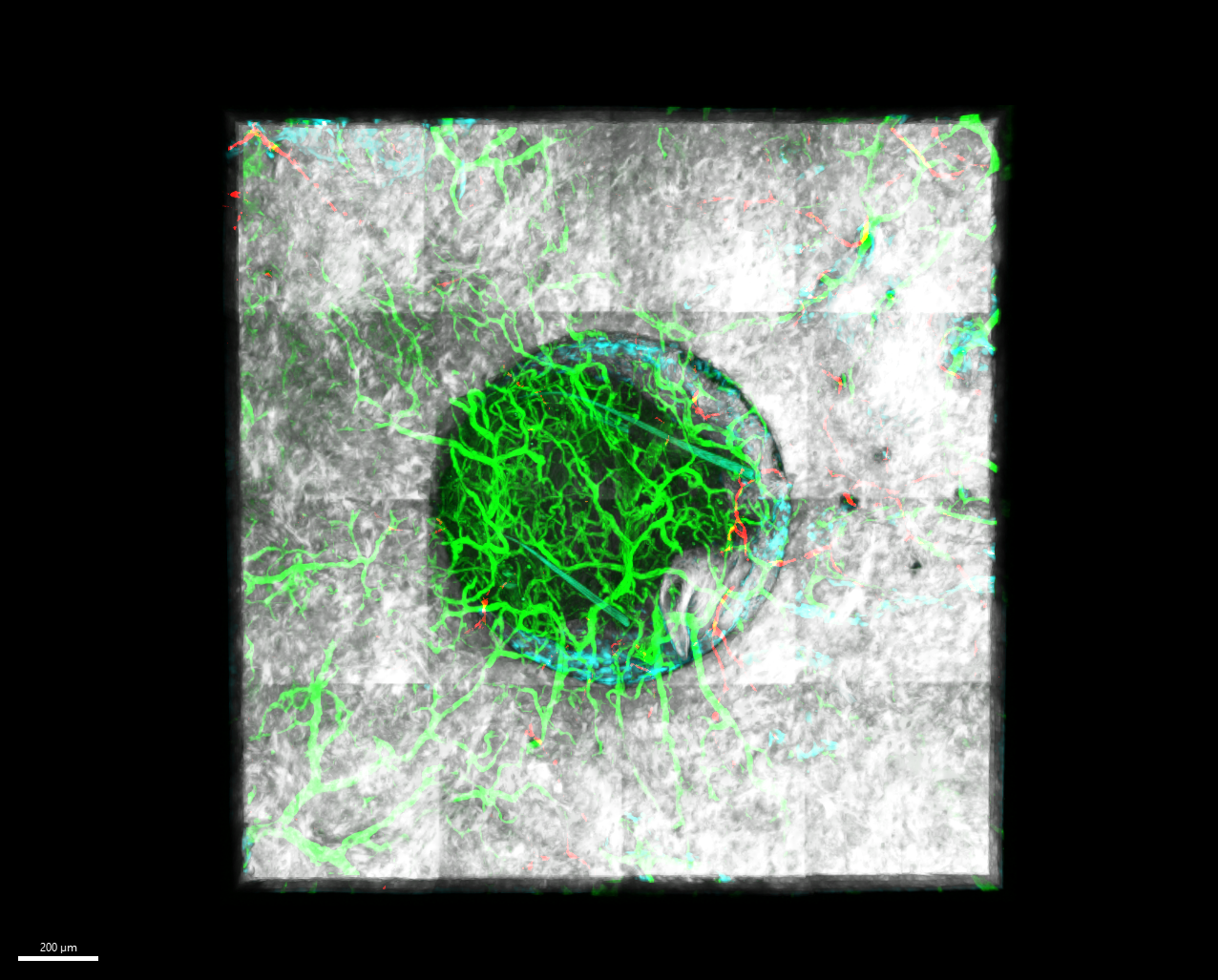

Supplement: Figure 1—source data 1. [file elife-83146-fig1-data1.zip › Figure 1/RE013 day 14 top side cd31 only endo gfp shg.tif]

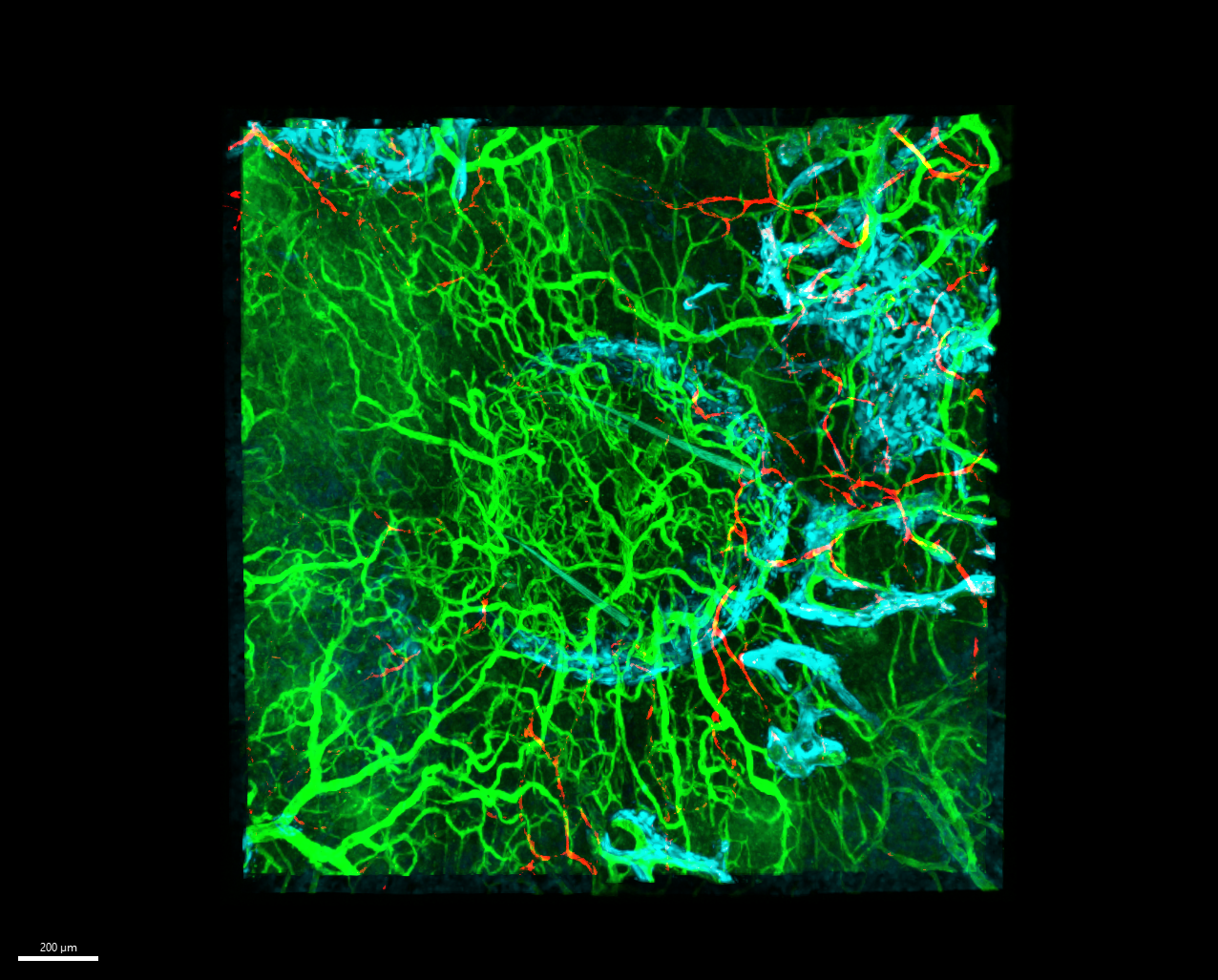

Supplement: Figure 1—source data 1. [file elife-83146-fig1-data1.zip › Figure 1/RE013 day 14 top side cd31 only endo gfp.tif]

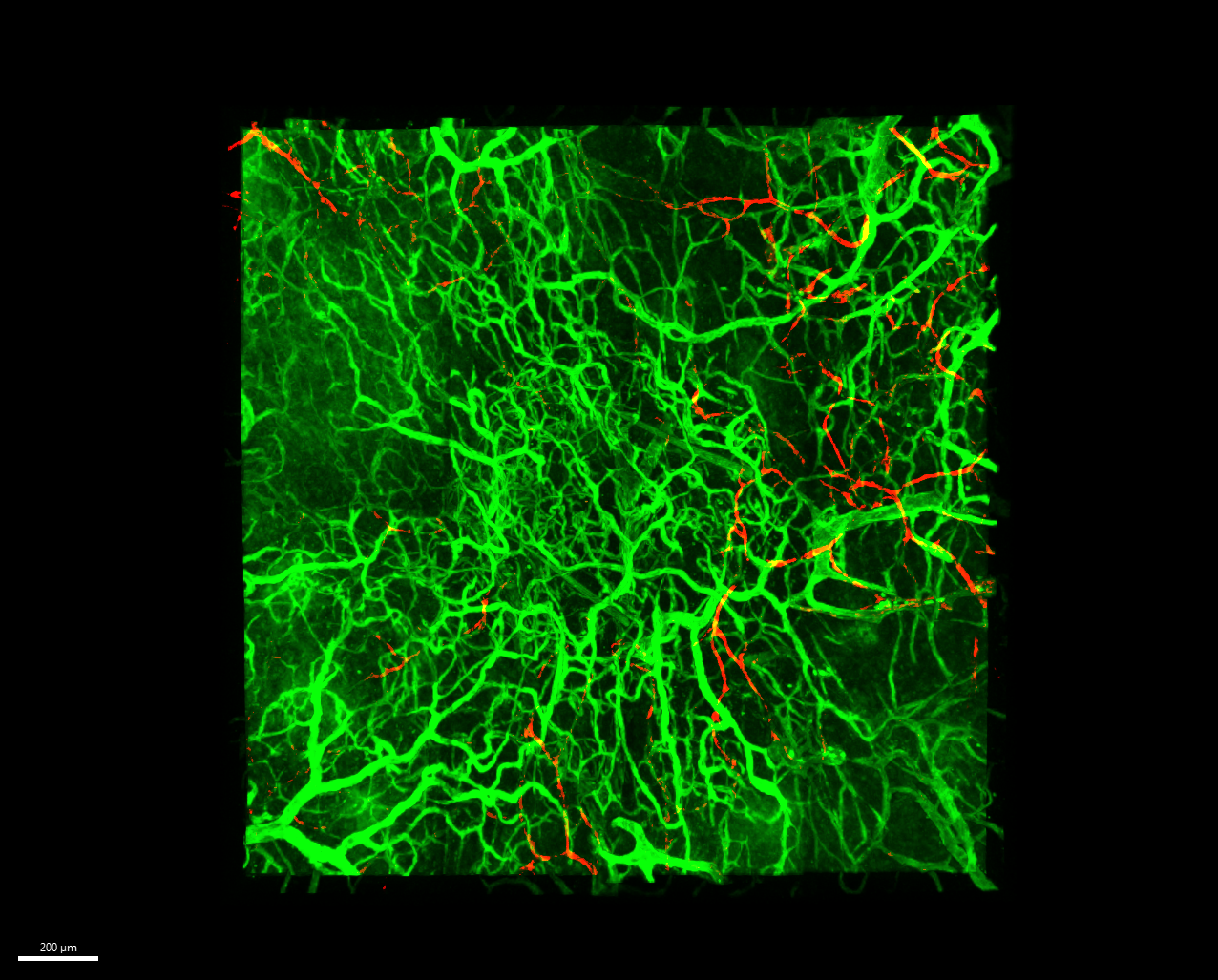

Supplement: Figure 1—source data 1. [file elife-83146-fig1-data1.zip › Figure 1/RE013 day 14 top side cd31 only endo.tif]

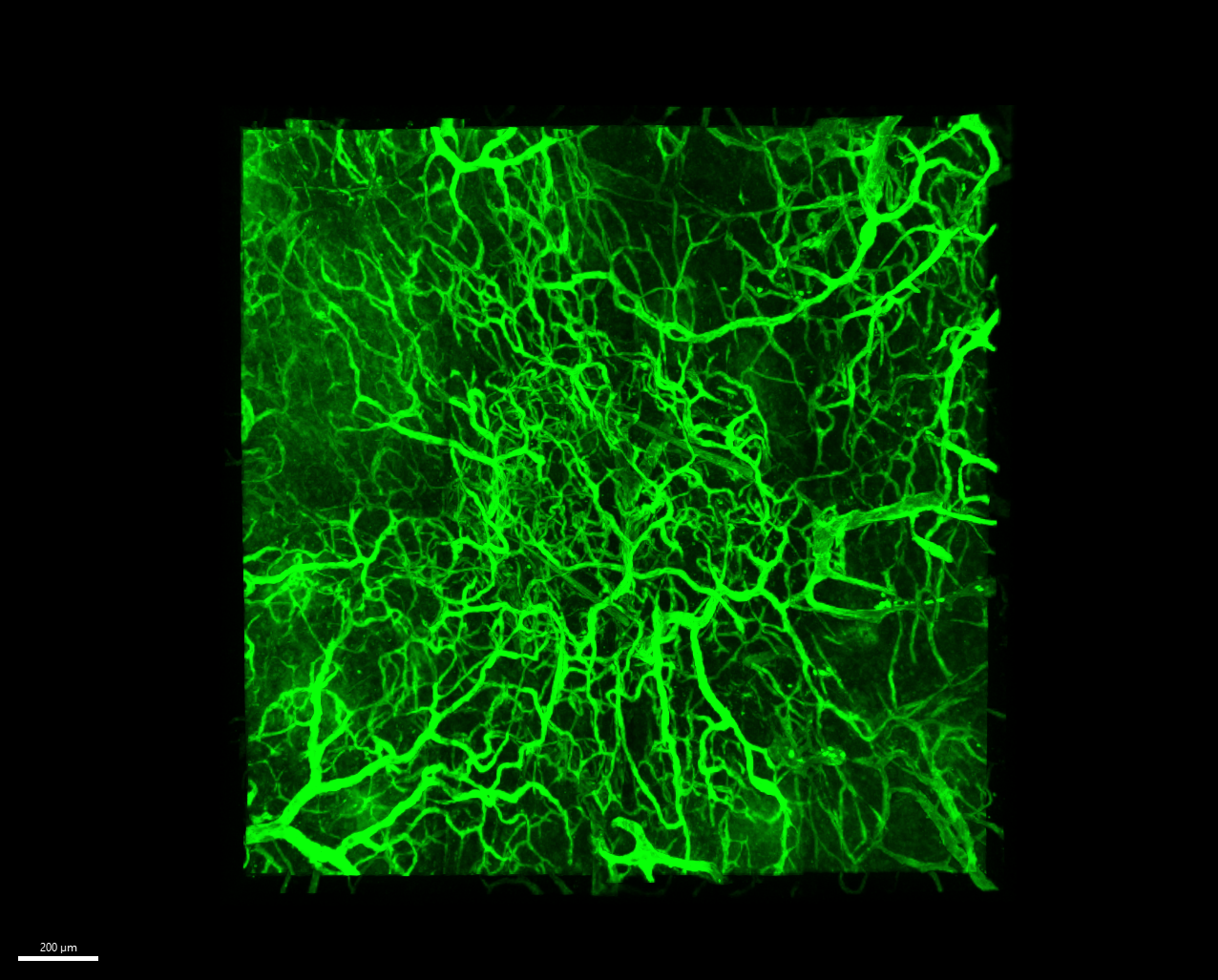

Supplement: Figure 1—source data 1. [file elife-83146-fig1-data1.zip › Figure 1/RE013 day 14 top side endo.tif]

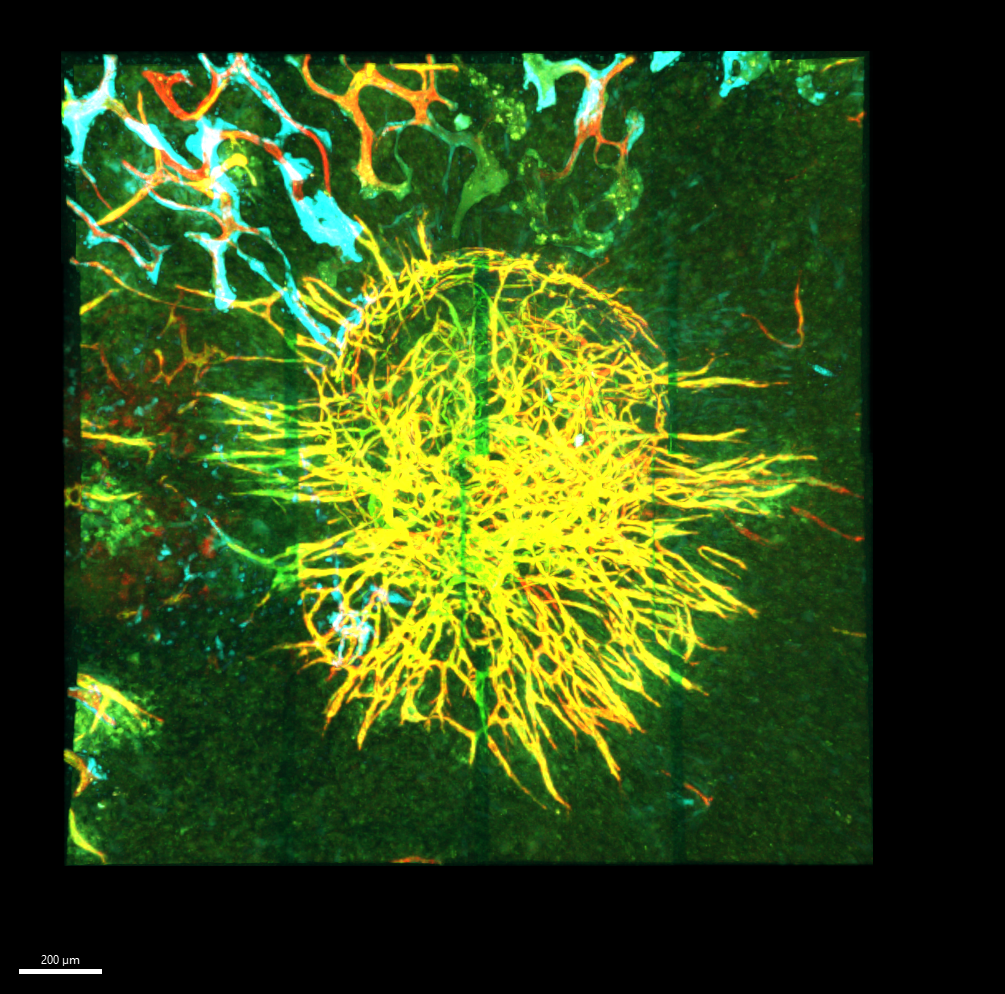

Supplement: Figure 1—source data 1. [file elife-83146-fig1-data1.zip › Figure 1/RE016 day 10 top side 1 33 slices for quan gfp cd31 endo good.tif]

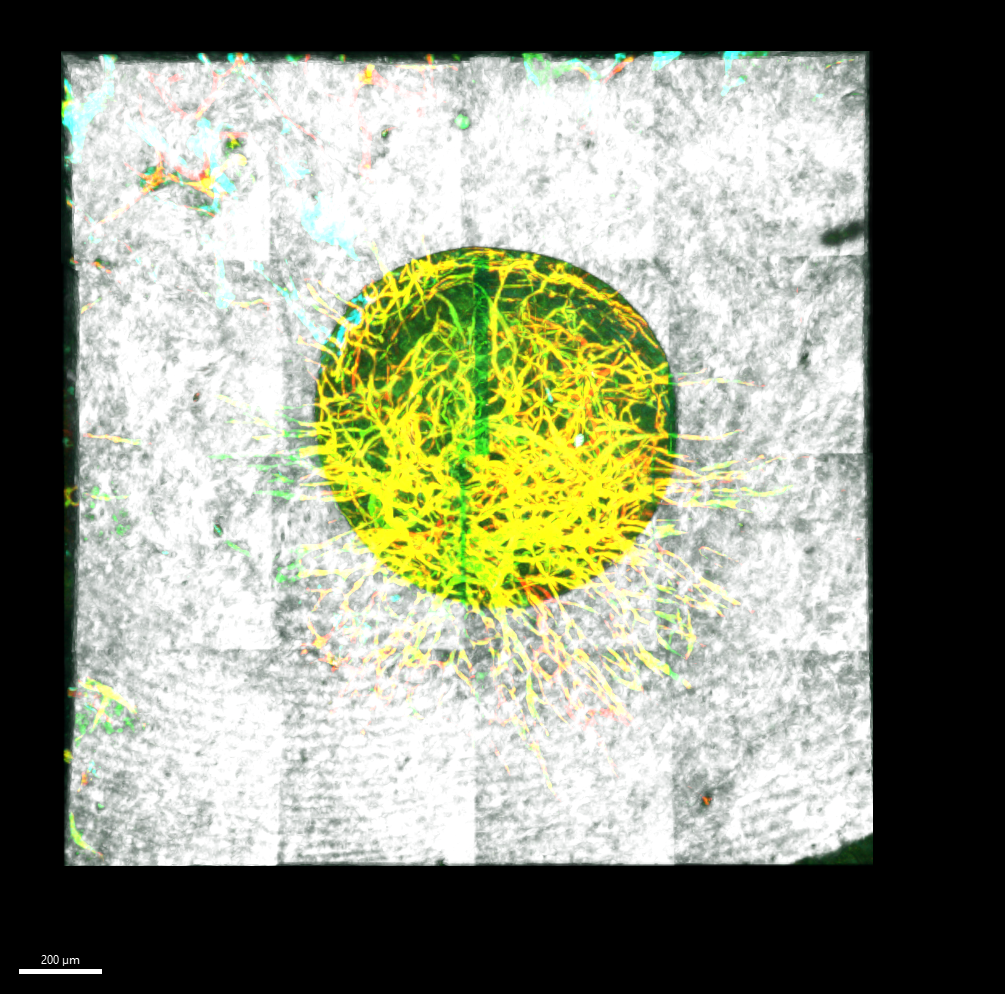

Supplement: Figure 1—source data 1. [file elife-83146-fig1-data1.zip › Figure 1/RE016 day 10 top side 1 33 slices for quan gfp cd31 endo shg good.tif]

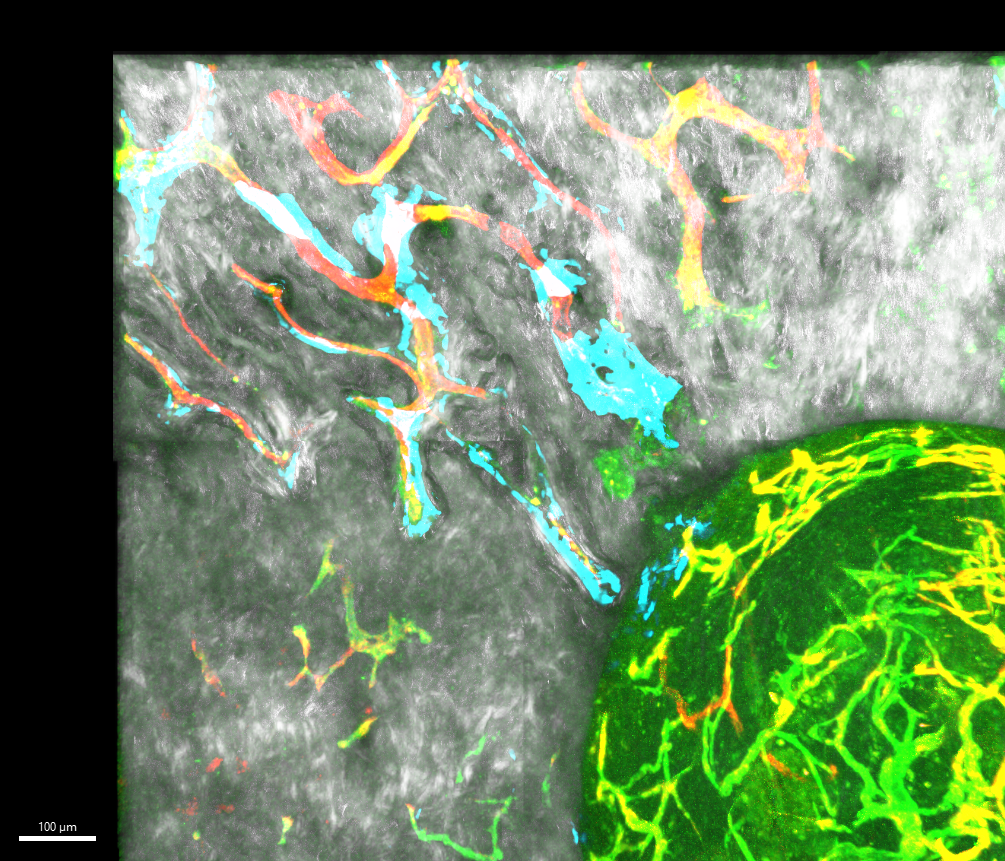

Supplement: Figure 1—source data 1. [file elife-83146-fig1-data1.zip › Figure 1/RE016 day 10 top side 1 33 slices for quan gfp cd31 endo shg zoom 1 - Copy.tif]

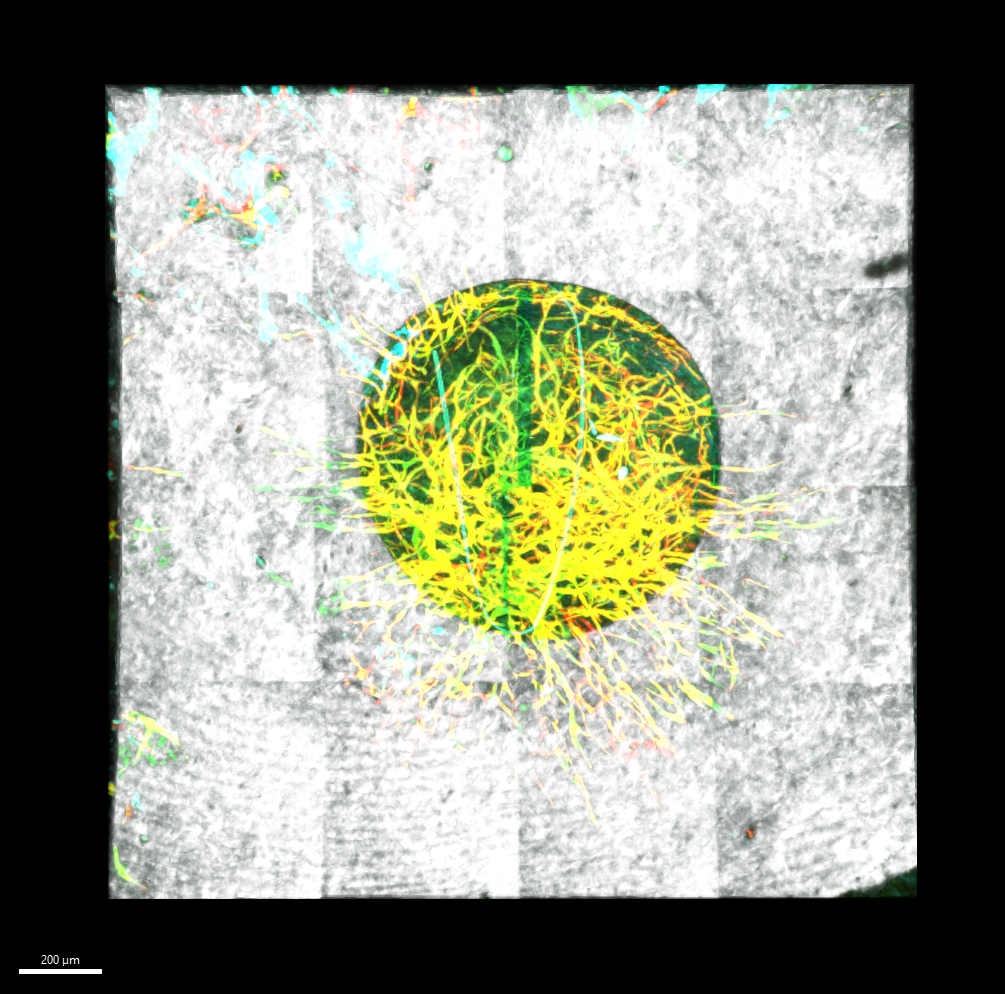

Supplement: Figure 1—source data 1. [file elife-83146-fig1-data1.zip › Figure 1/RE016 day 10 top side 1 33 slices for quan gfp cd31 endo shg.tif]

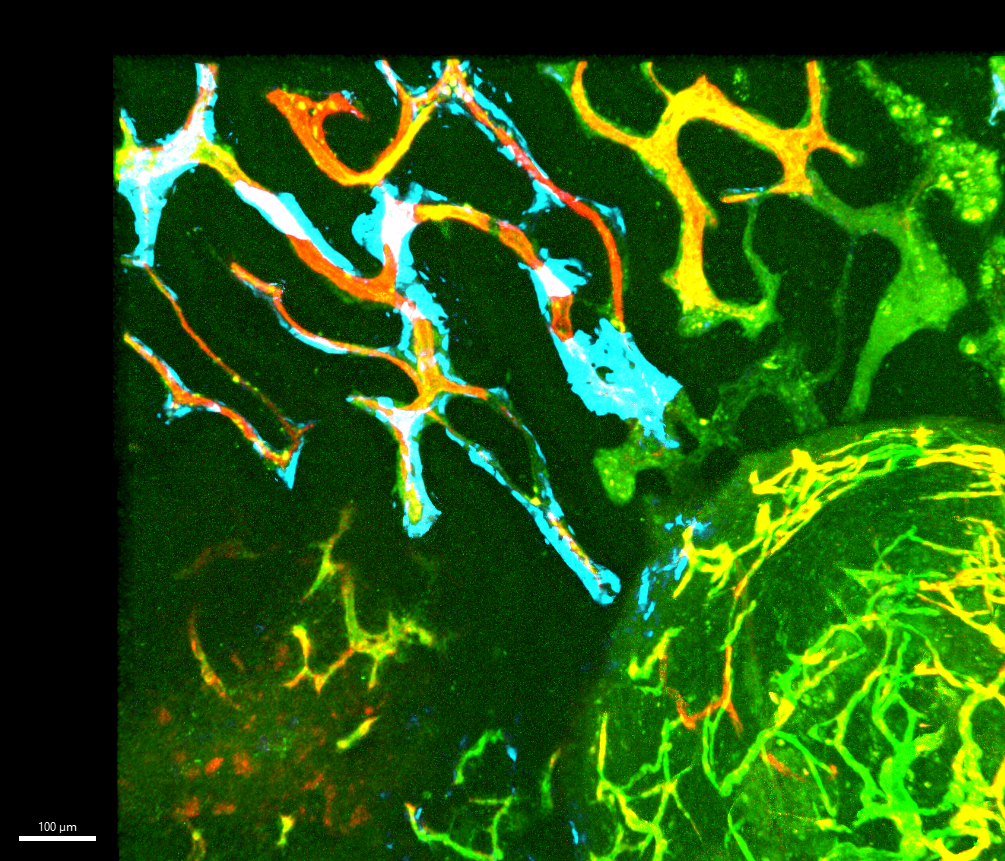

Supplement: Figure 1—source data 1. [file elife-83146-fig1-data1.zip › Figure 1/RE016 day 10 top side 1 33 slices for quan gfp cd31 endo zoom 1.tif]

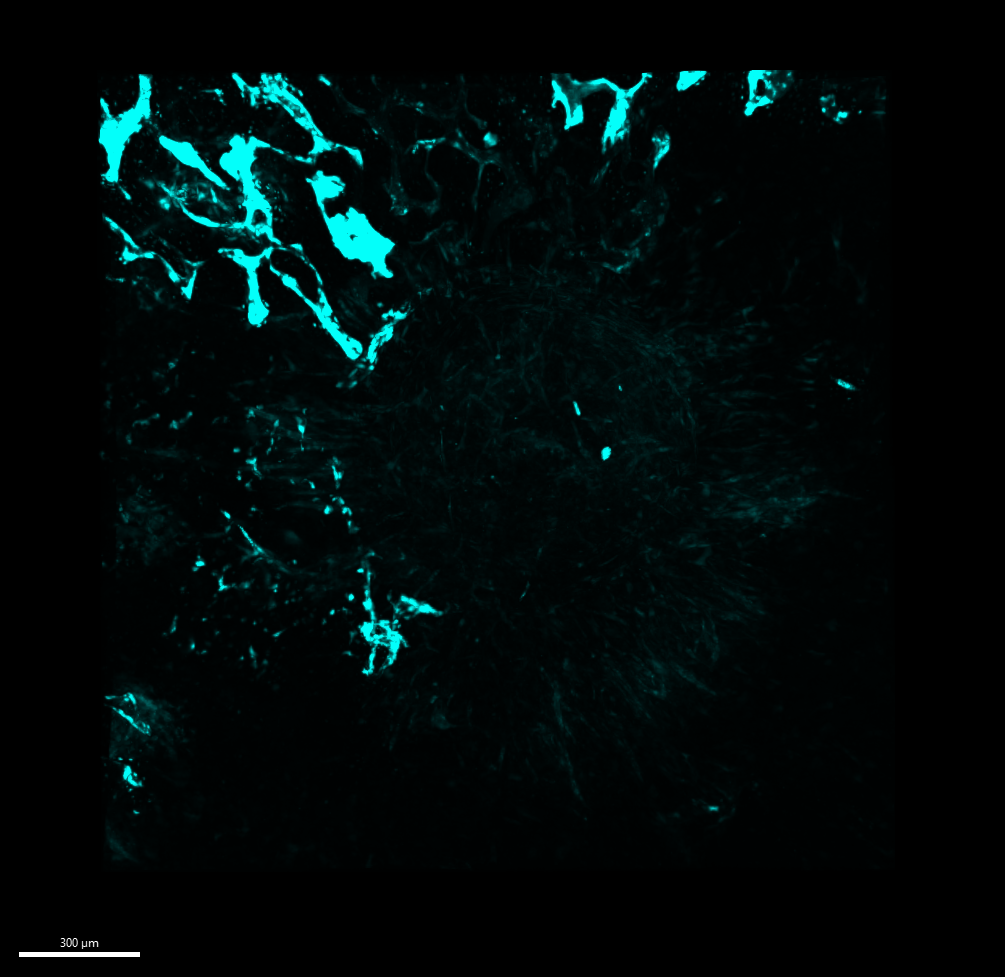

Supplement: Figure 1—source data 1. [file elife-83146-fig1-data1.zip › Figure 1/RE016 day 10 top side 1 33 slices for quan gfp.tif]

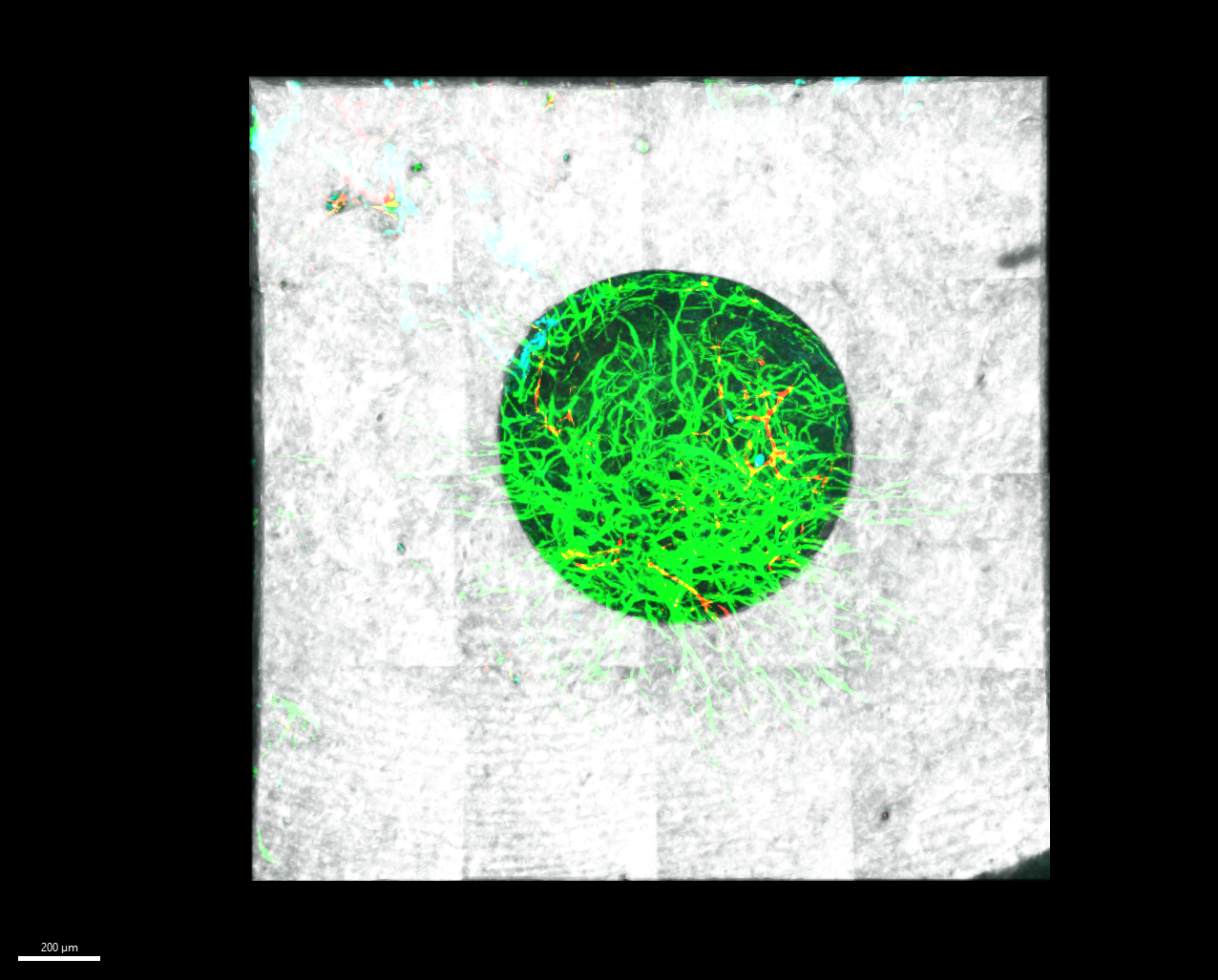

Supplement: Figure 1—source data 1. [file elife-83146-fig1-data1.zip › Figure 1/RE016 day 10 top side cd31 only endo gfp shg.tif]

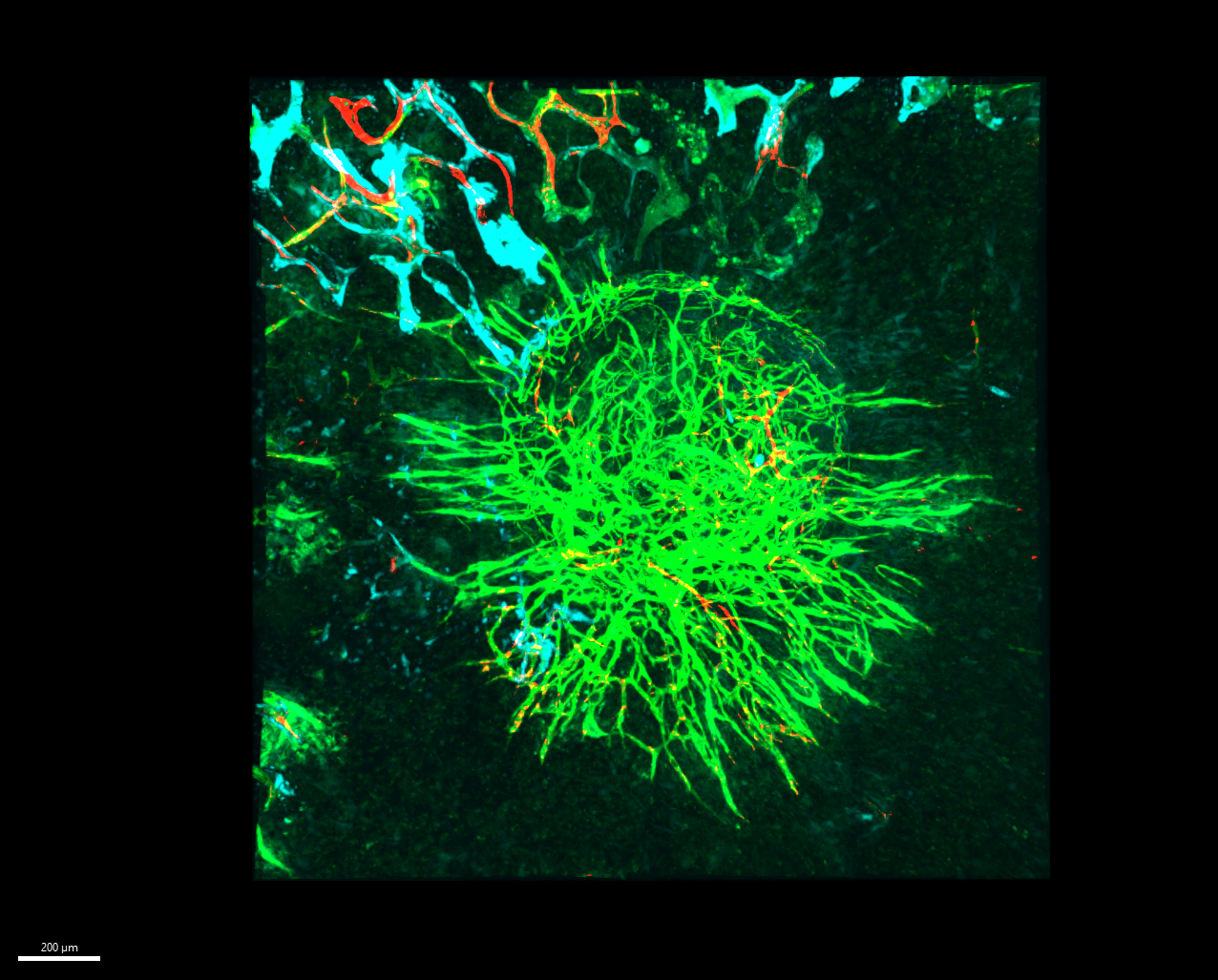

Supplement: Figure 1—source data 1. [file elife-83146-fig1-data1.zip › Figure 1/RE016 day 10 top side cd31 only endo gfp.tif]

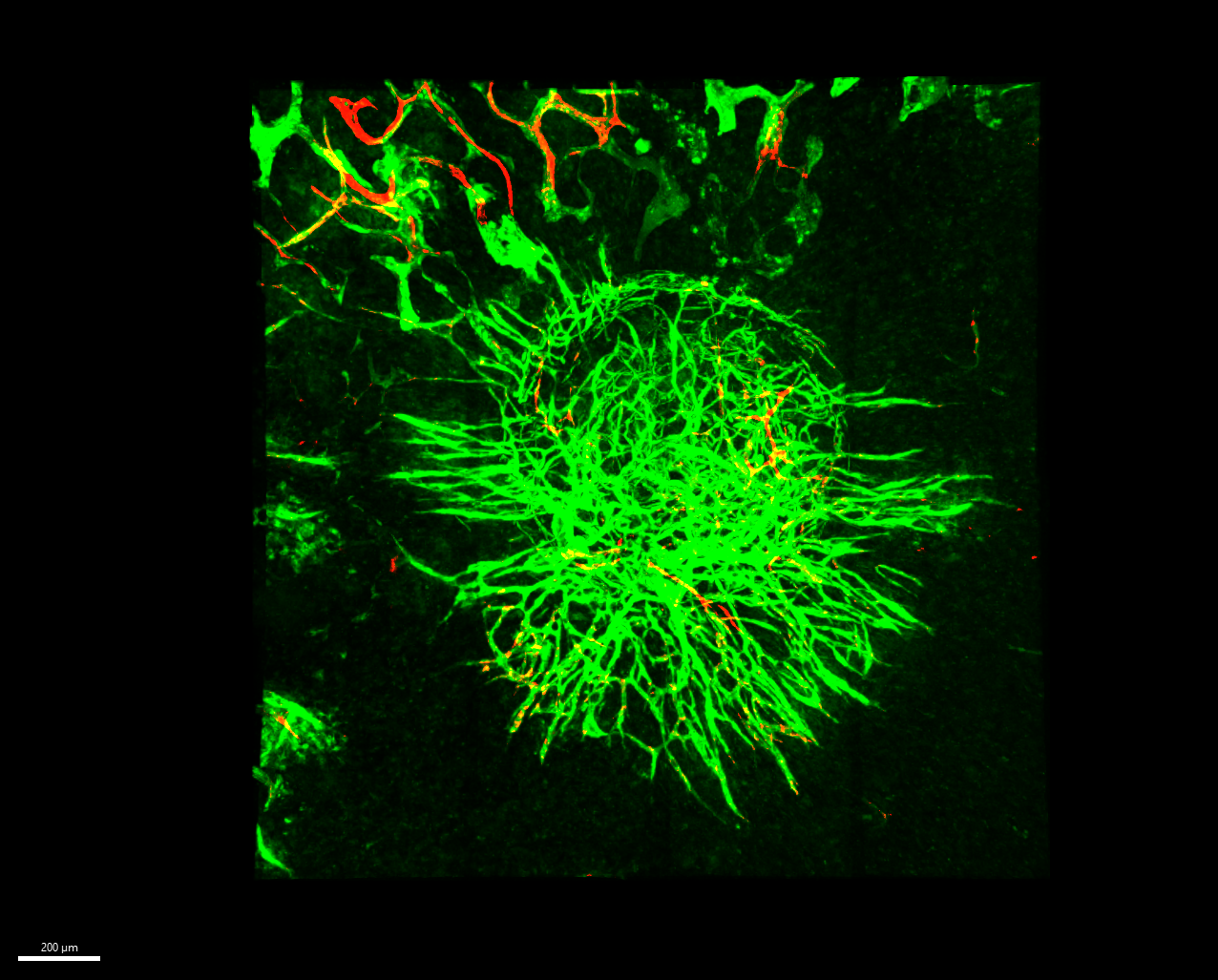

Supplement: Figure 1—source data 1. [file elife-83146-fig1-data1.zip › Figure 1/RE016 day 10 top side cd31 only endo.tif]

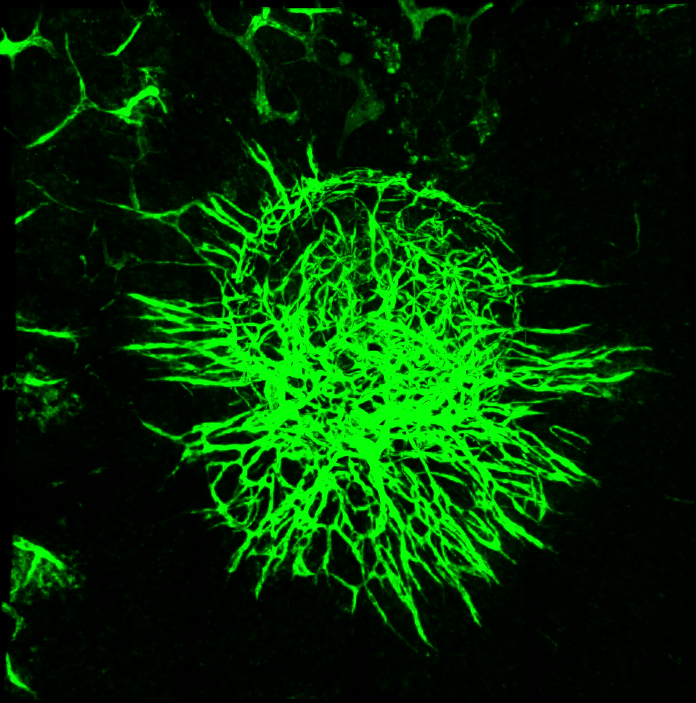

Supplement: Figure 1—source data 1. [file elife-83146-fig1-data1.zip › Figure 1/RE016 day10 topsideendo.tif]

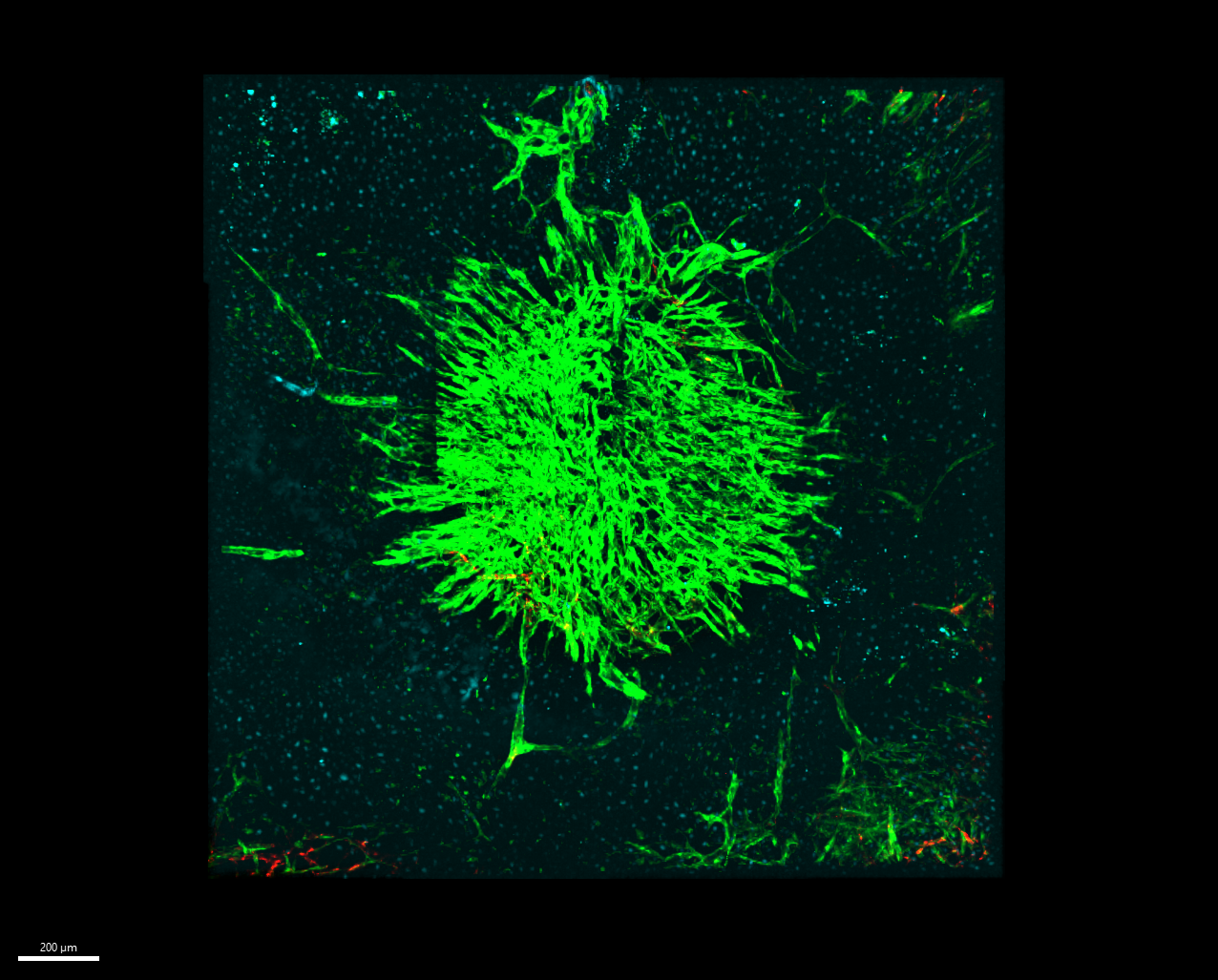

Supplement: Figure 1—source data 1. [file elife-83146-fig1-data1.zip › Figure 1/RE085 day 5 1mm top side 1 cd31 only and endo 2.tif]

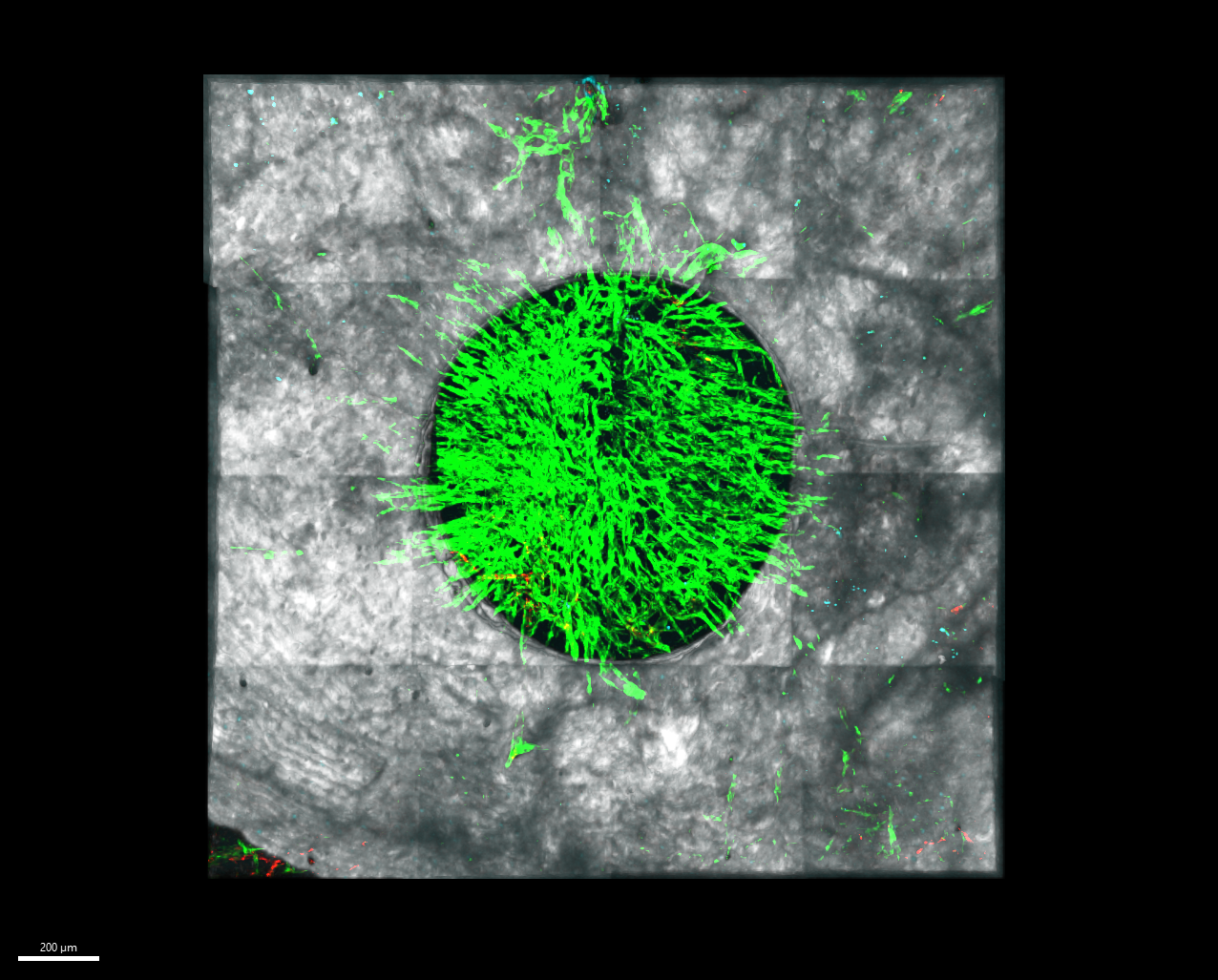

Supplement: Figure 1—source data 1. [file elife-83146-fig1-data1.zip › Figure 1/RE085 day 5 1mm top side 1 cd31 only and endo.tif]

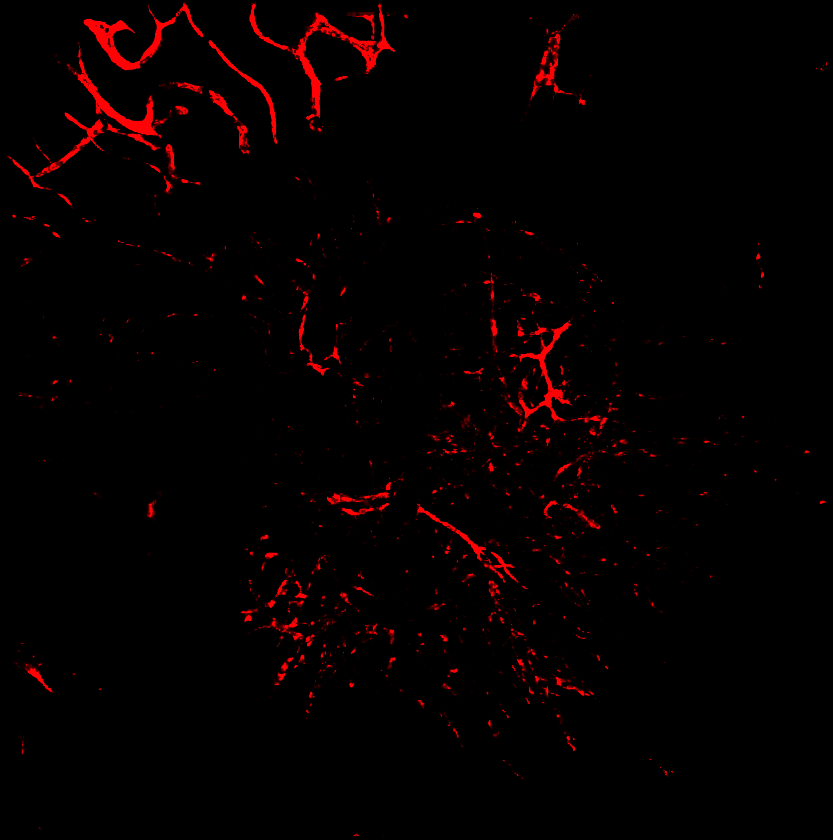

Supplement: Figure 1—source data 1. [file elife-83146-fig1-data1.zip › Figure 1/RE085 day 5 1mm top side 1 cd31 only crop.tif]

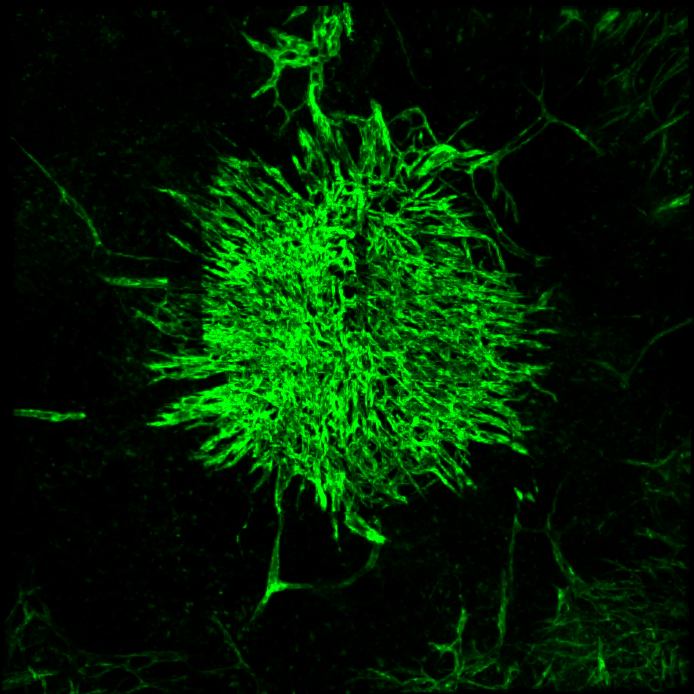

Supplement: Figure 1—source data 1. [file elife-83146-fig1-data1.zip › Figure 1/RE085 day 5 1mm top side 1 endo.tif]

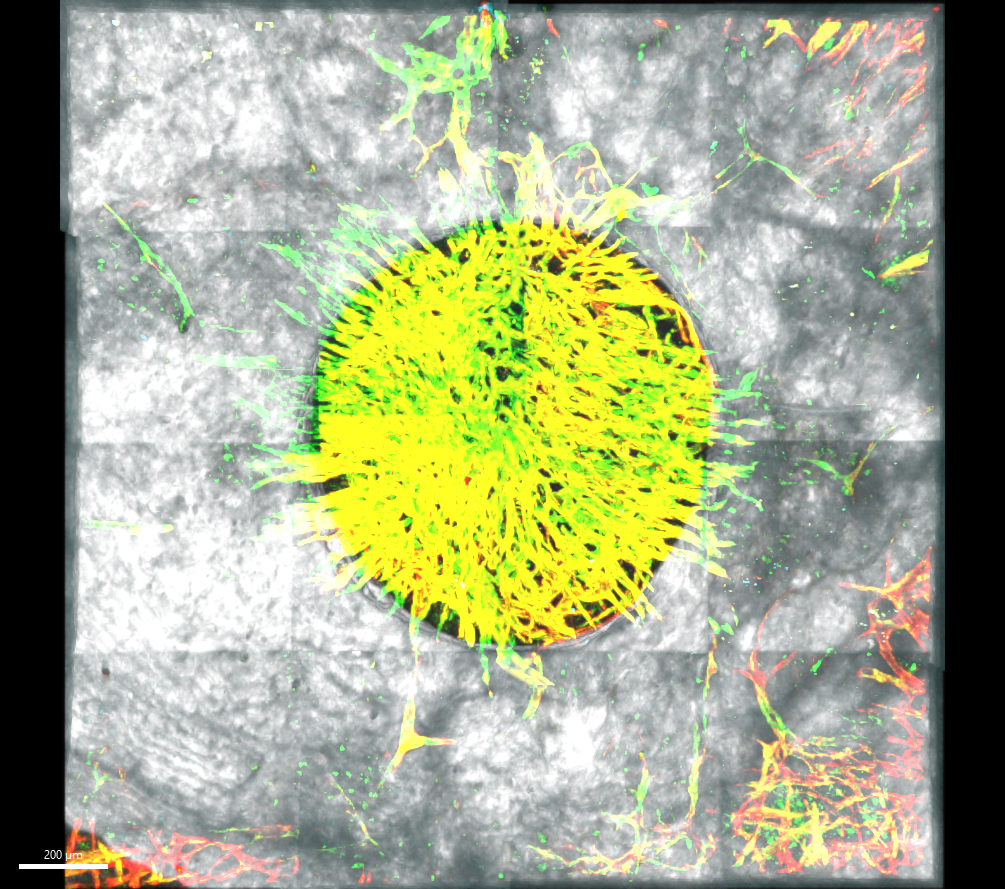

Supplement: Figure 1—source data 1. [file elife-83146-fig1-data1.zip › Figure 1/RE085 day 5 1mm top side 2 gfp endo cd31- shg.tif]

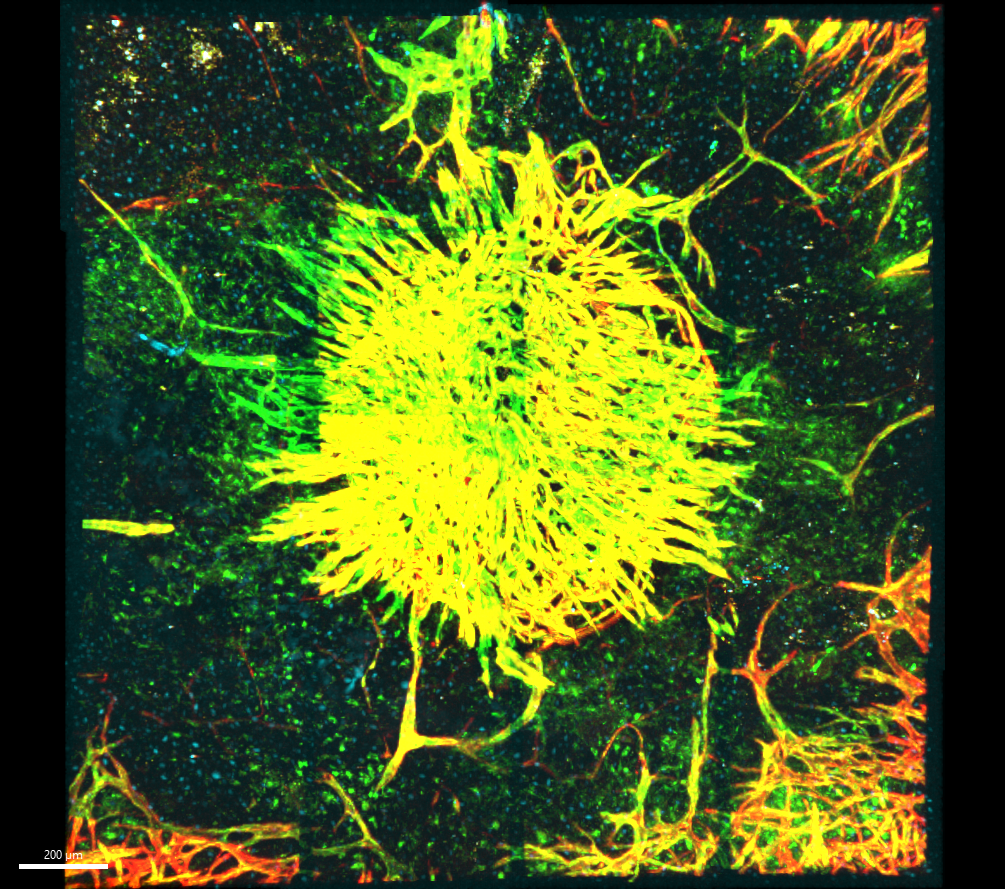

Supplement: Figure 1—source data 1. [file elife-83146-fig1-data1.zip › Figure 1/RE085 day 5 1mm top side 2 gfp endo cd31.tif]

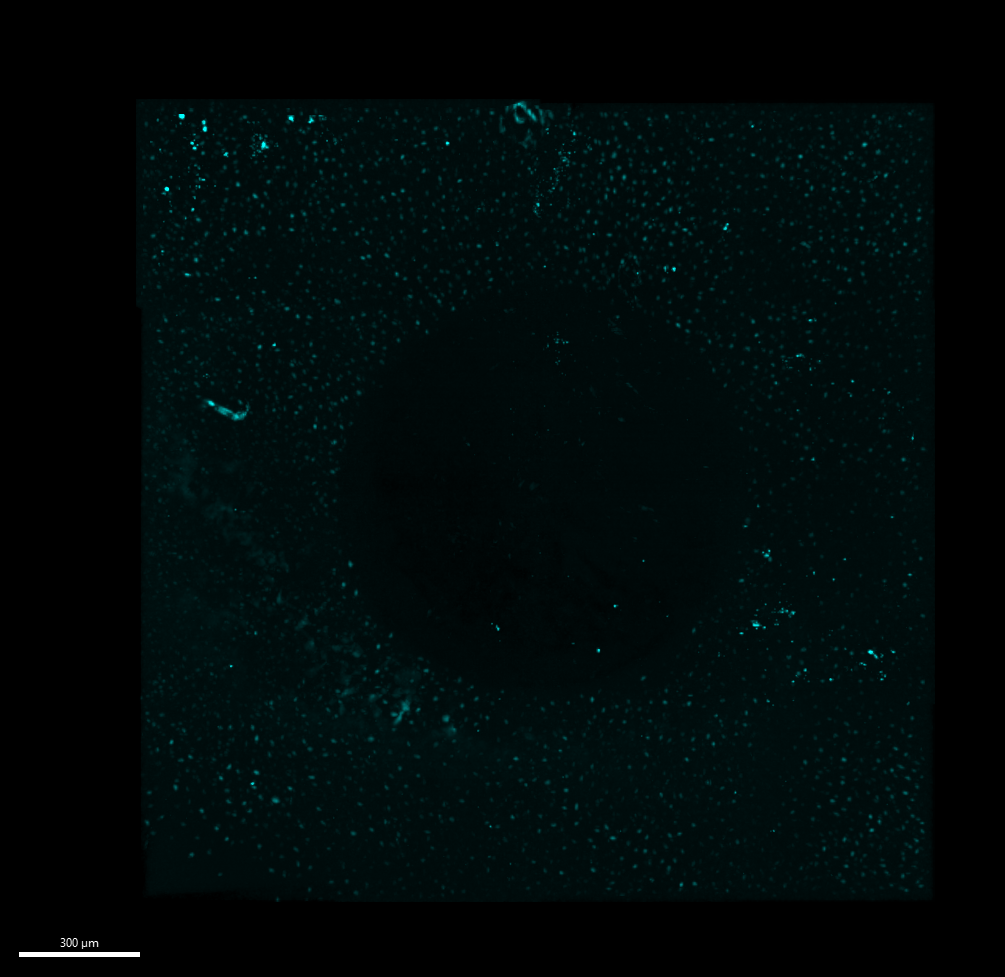

Supplement: Figure 1—source data 1. [file elife-83146-fig1-data1.zip › Figure 1/RE085 day 5 1mm top side 2 gfp.tif]

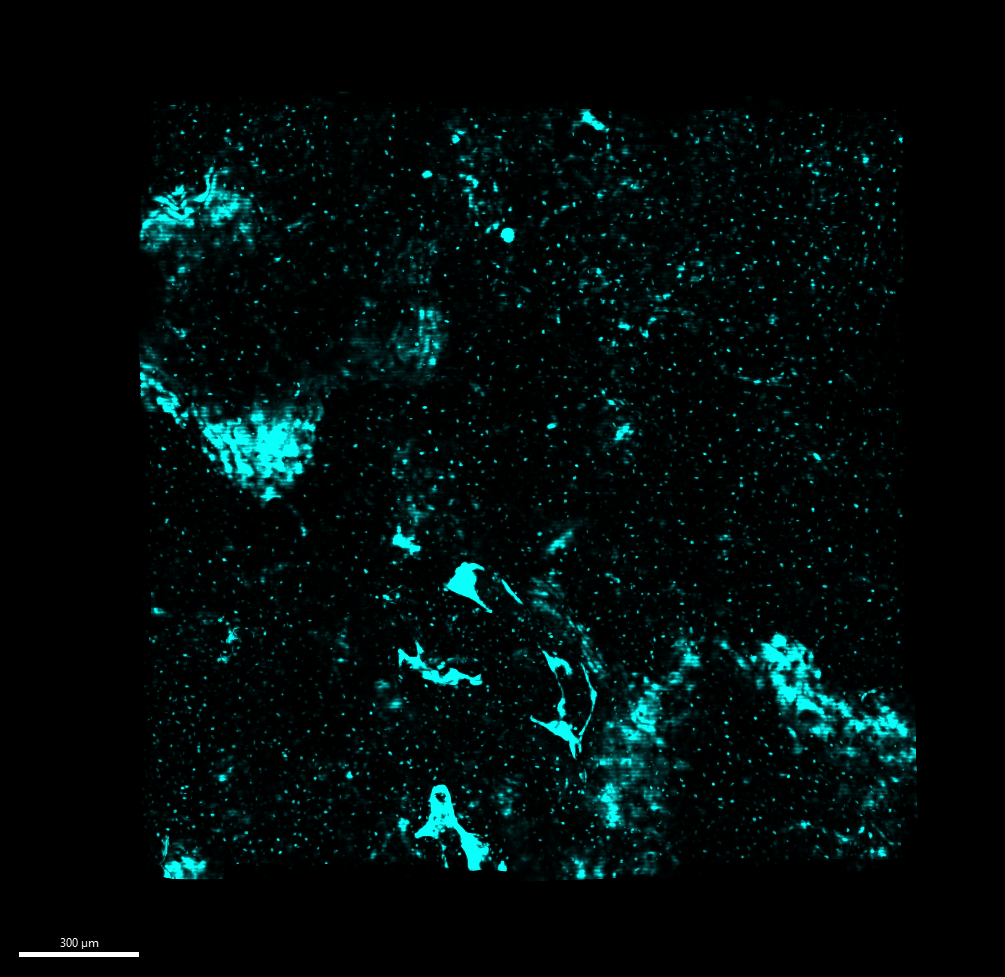

Supplement: Figure 2—source data 1. [file elife-83146-fig2-data1.zip › Figure 2/000 non injury normal bone flip side scan gfp.tif]

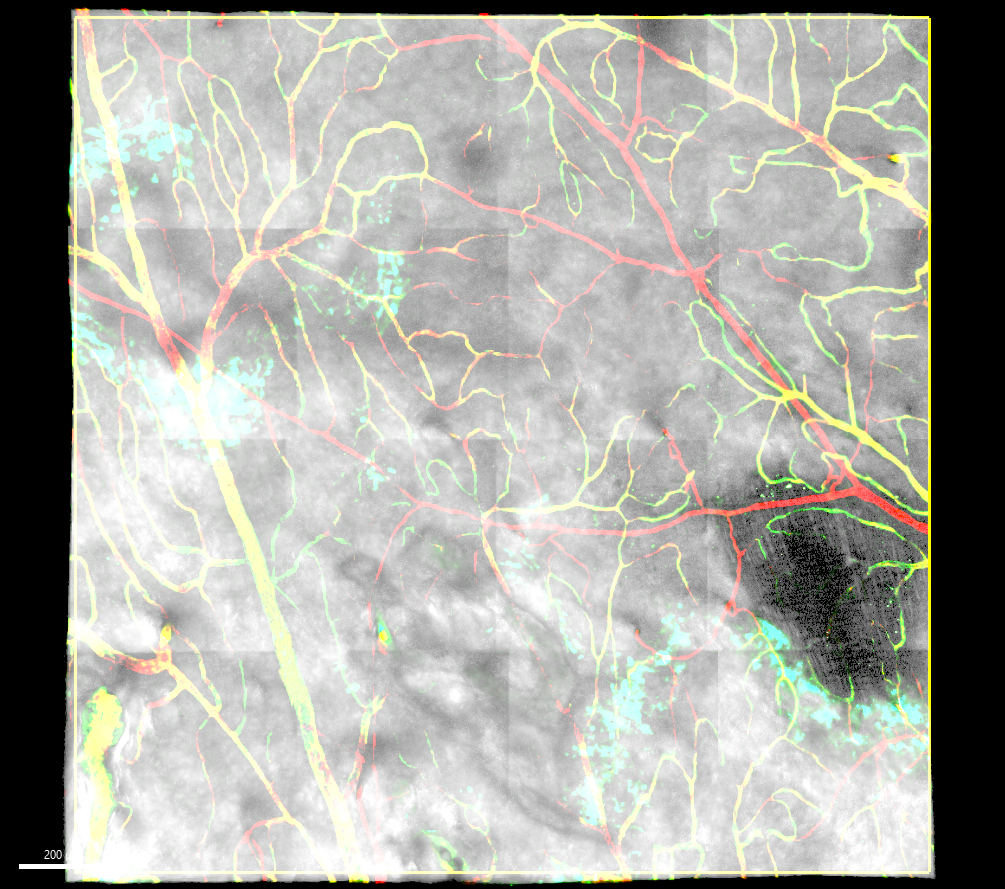

Supplement: Figure 2—source data 1. [file elife-83146-fig2-data1.zip › Figure 2/000 non injury normal bone top side scan bottom slices cd31 endo gfp shg.tif]

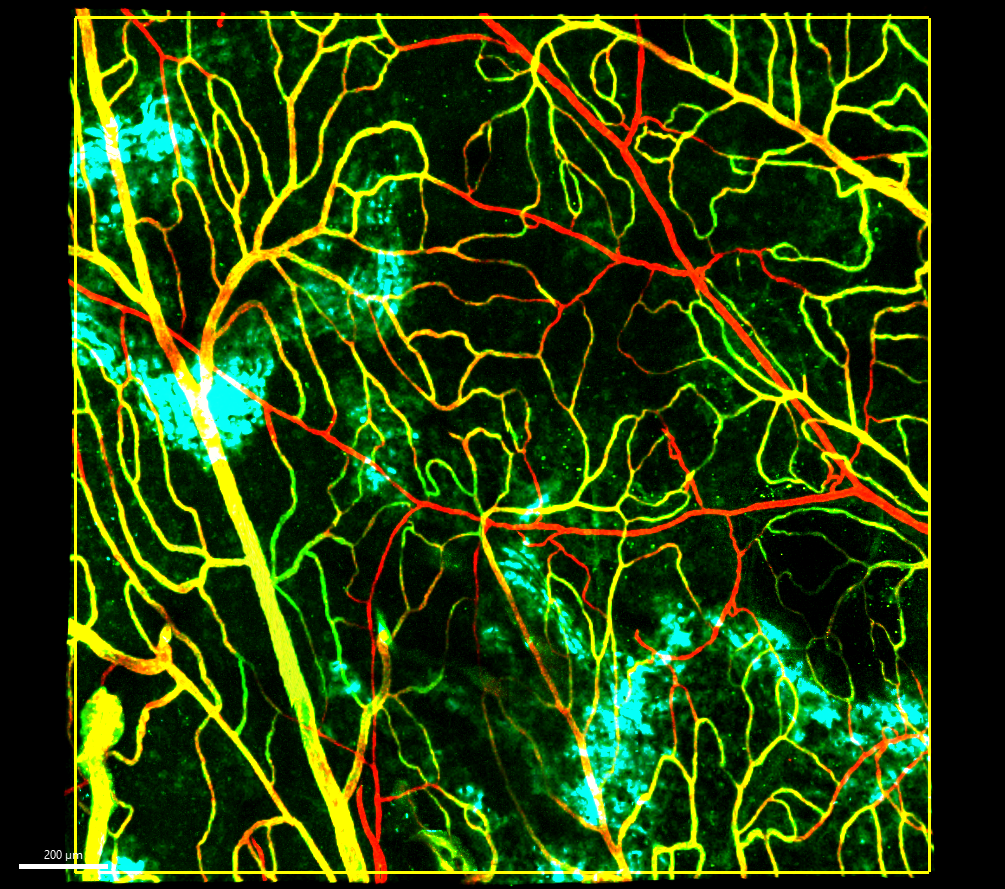

Supplement: Figure 2—source data 1. [file elife-83146-fig2-data1.zip › Figure 2/000 non injury normal bone top side scan bottom slices cd31 endo gfp.tif]

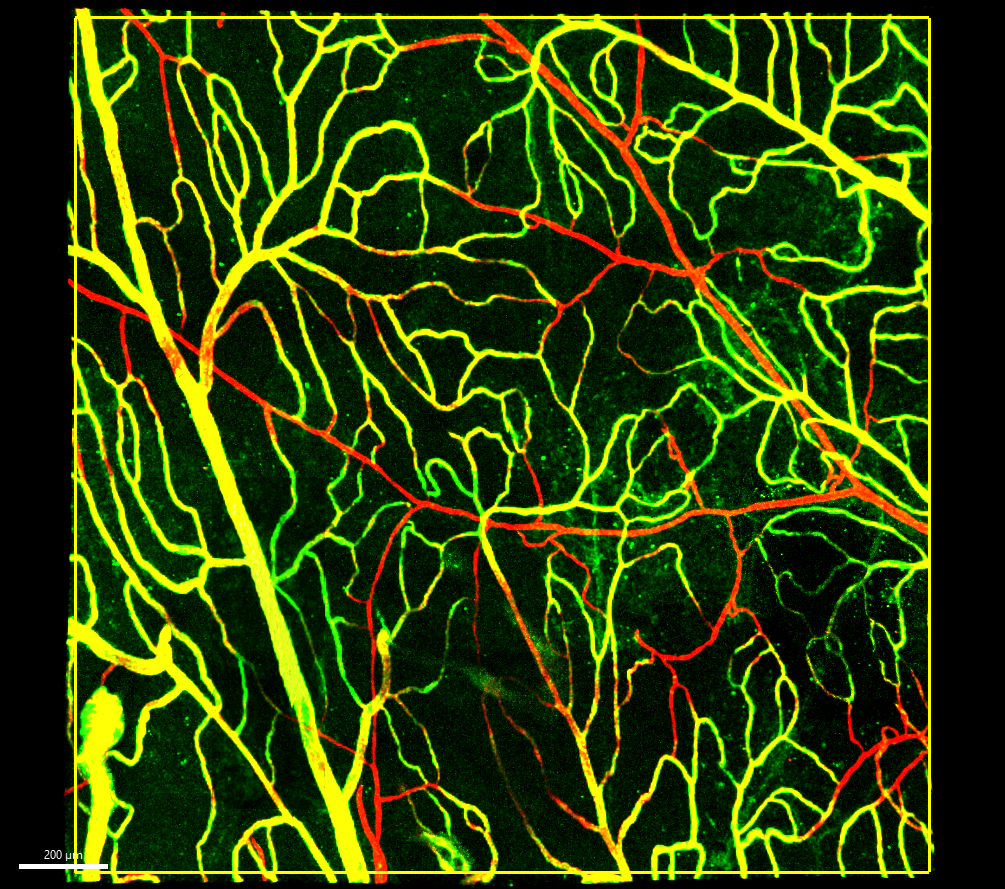

Supplement: Figure 2—source data 1. [file elife-83146-fig2-data1.zip › Figure 2/000 non injury normal bone top side scan bottom slices cd31 endo good.tif]

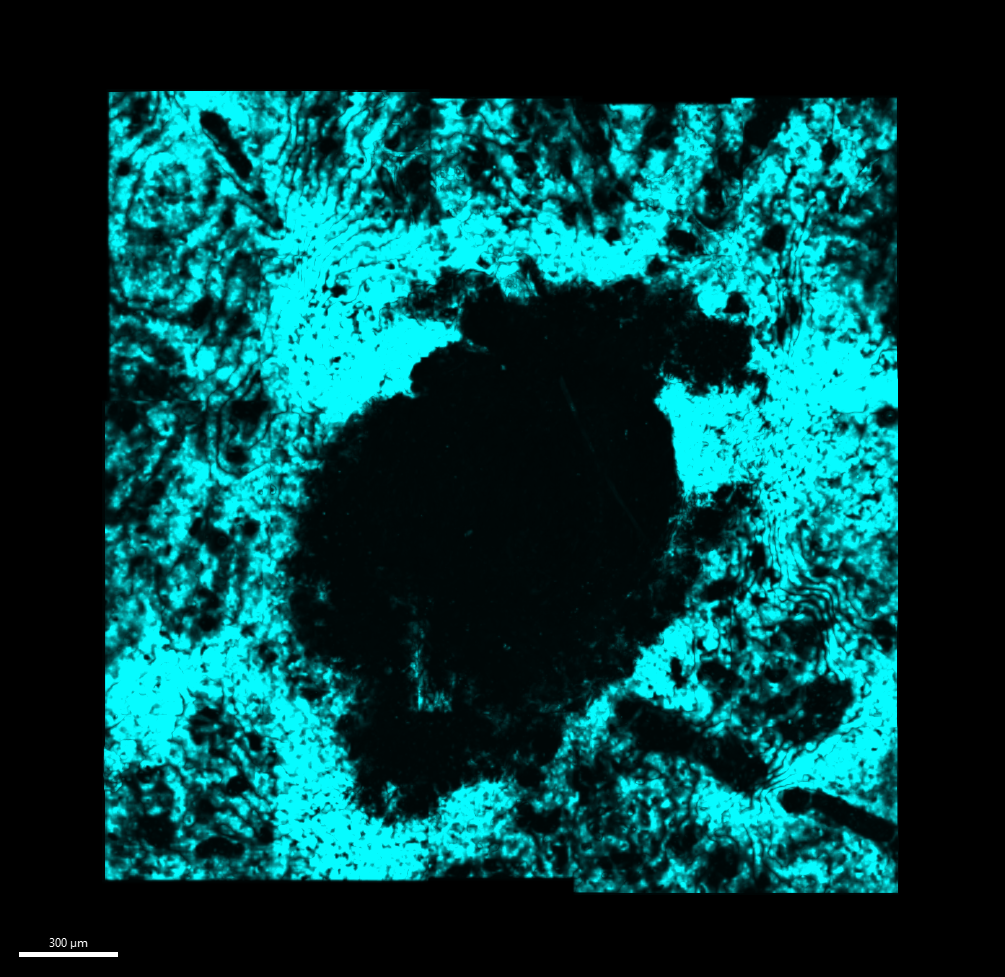

Supplement: Figure 2—source data 1. [file elife-83146-fig2-data1.zip › Figure 2/001 1mm day 1 flip for quan_ gfp-2.tif]

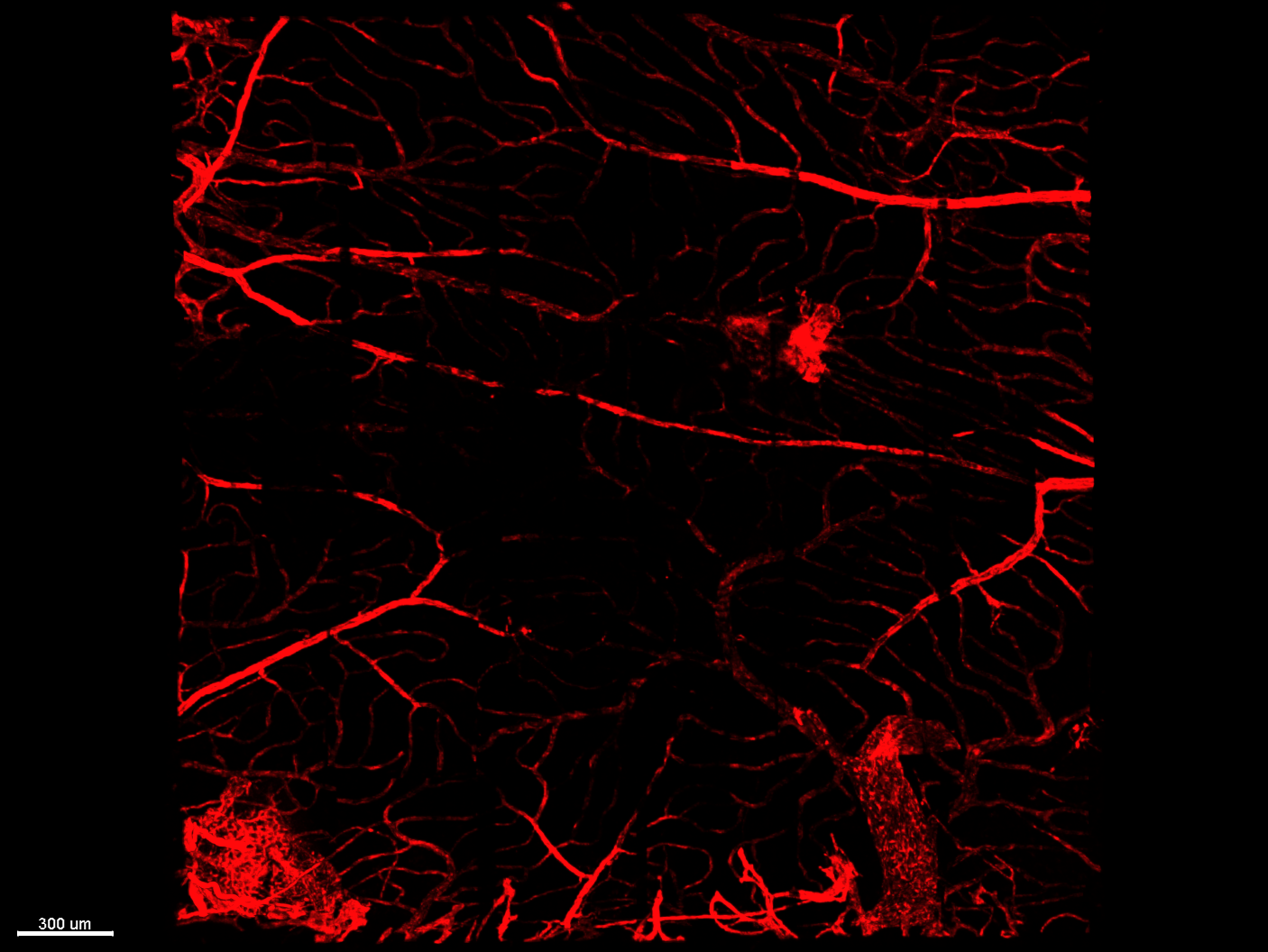

Supplement: Figure 2—source data 1. [file elife-83146-fig2-data1.zip › Figure 2/002 1mm day 1 flip cd31only.tif]

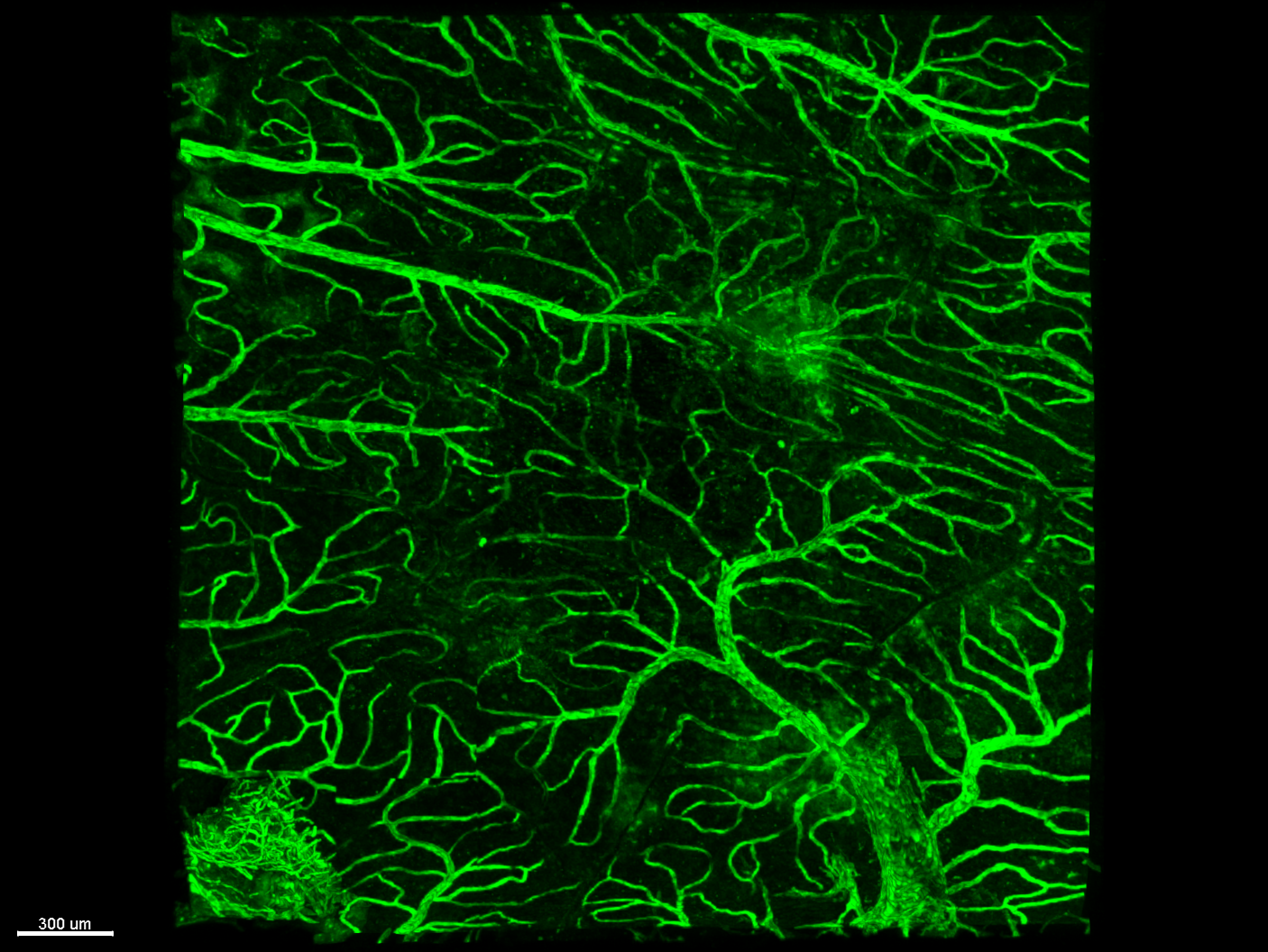

Supplement: Figure 2—source data 1. [file elife-83146-fig2-data1.zip › Figure 2/002 1mm day 1 flip endo only.tif]

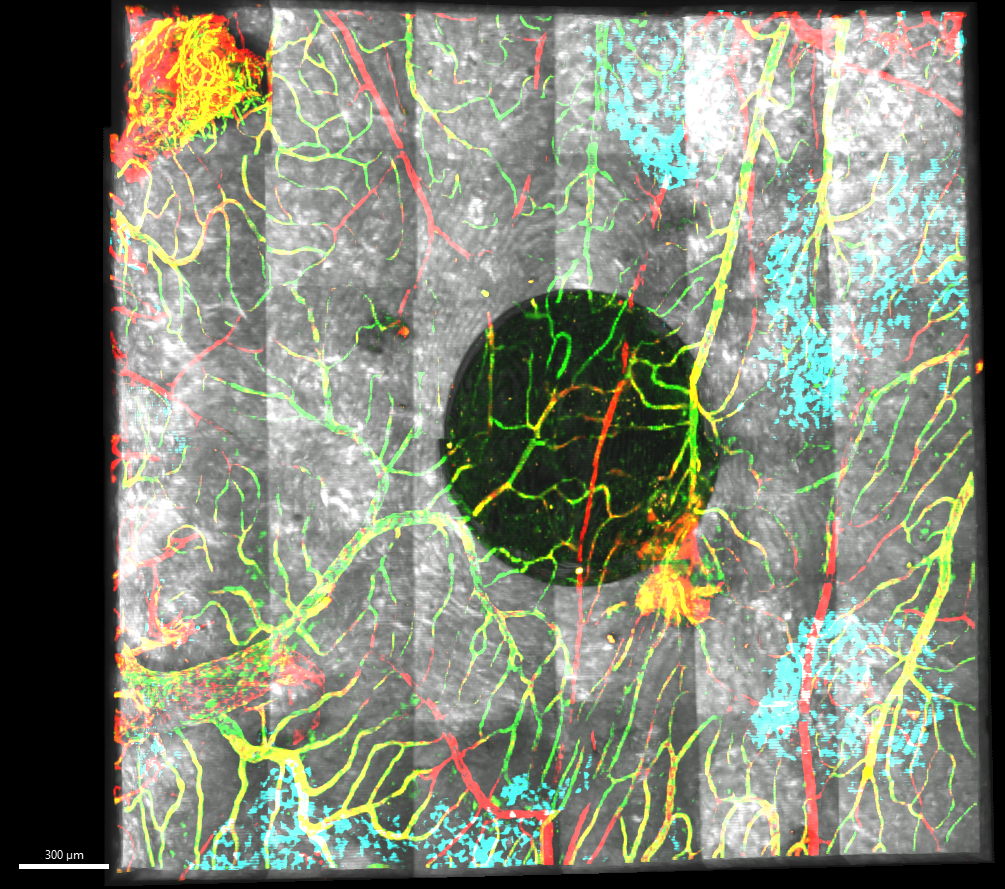

Supplement: Figure 2—source data 1. [file elife-83146-fig2-data1.zip › Figure 2/002 1mm day 1 flip for quan_cd31 endo gfp shg.tif]

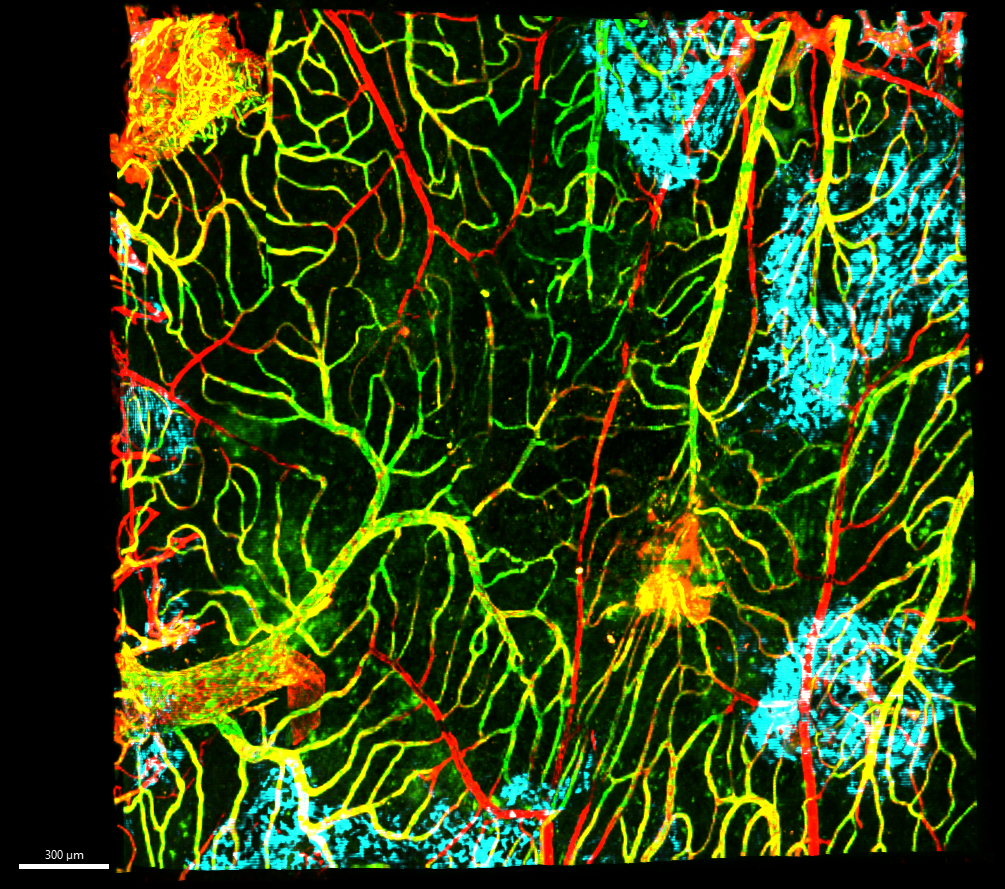

Supplement: Figure 2—source data 1. [file elife-83146-fig2-data1.zip › Figure 2/002 1mm day 1 flip for quan_cd31 endo gfp.tif]

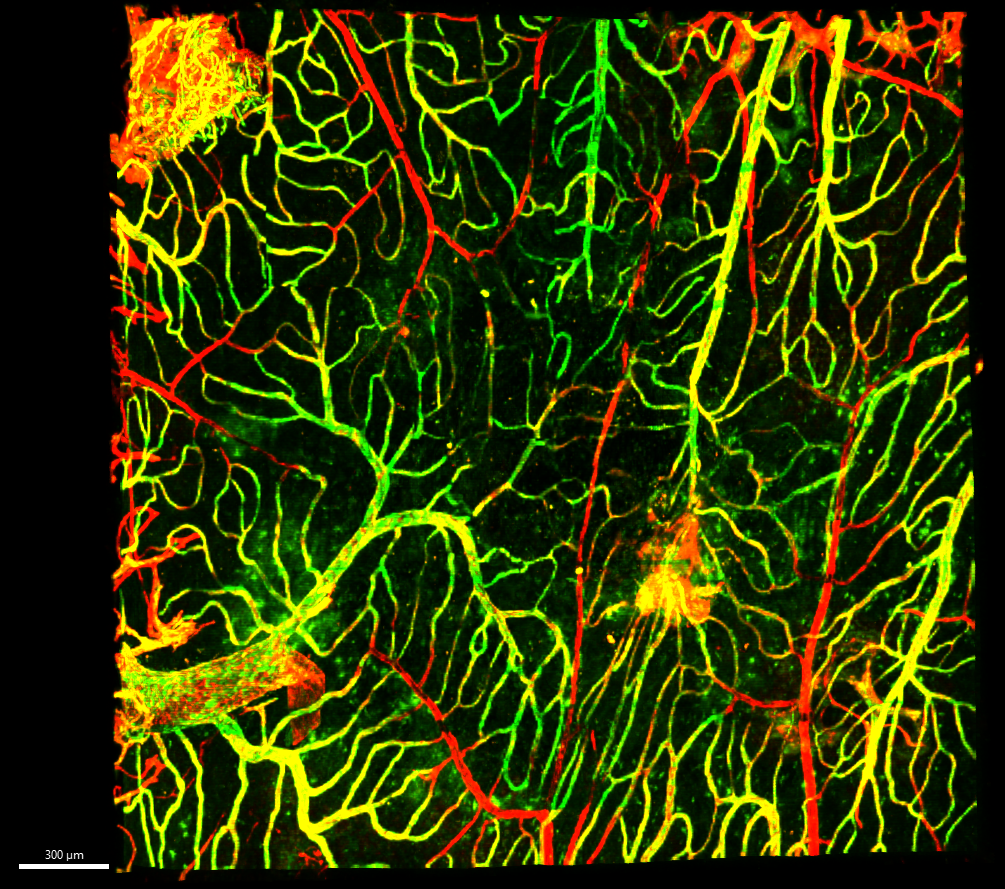

Supplement: Figure 2—source data 1. [file elife-83146-fig2-data1.zip › Figure 2/002 1mm day 1 flip for quan_cd31 endo.tif]

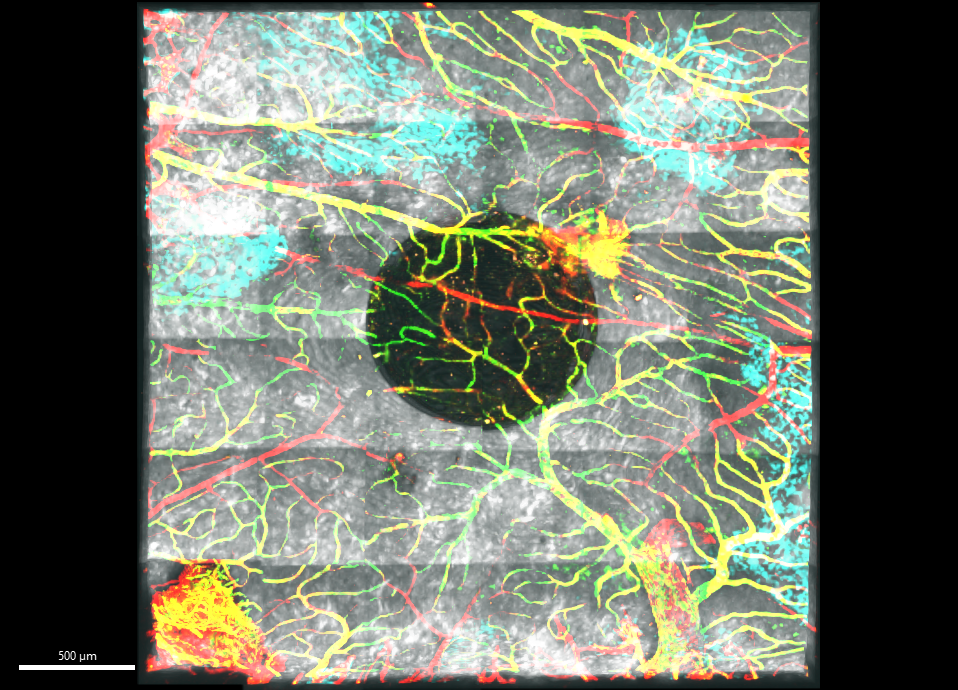

Supplement: Figure 2—source data 1. [file elife-83146-fig2-data1.zip › Figure 2/002 1mm day 1 flip for quan_endo cd31 gfp shg.tif]

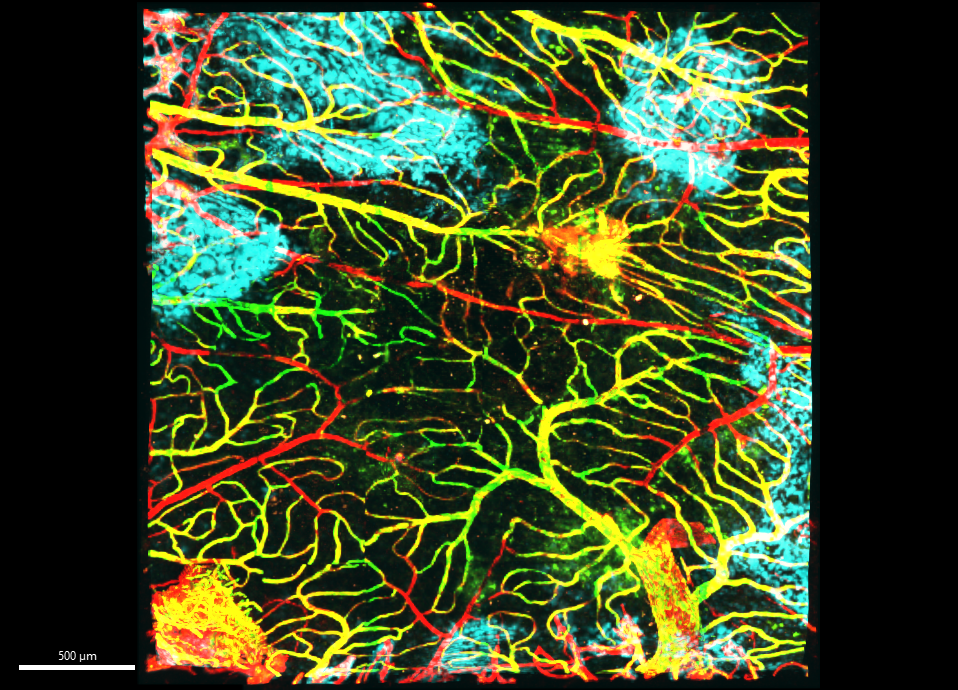

Supplement: Figure 2—source data 1. [file elife-83146-fig2-data1.zip › Figure 2/002 1mm day 1 flip for quan_endo cd31 gfp.tif]

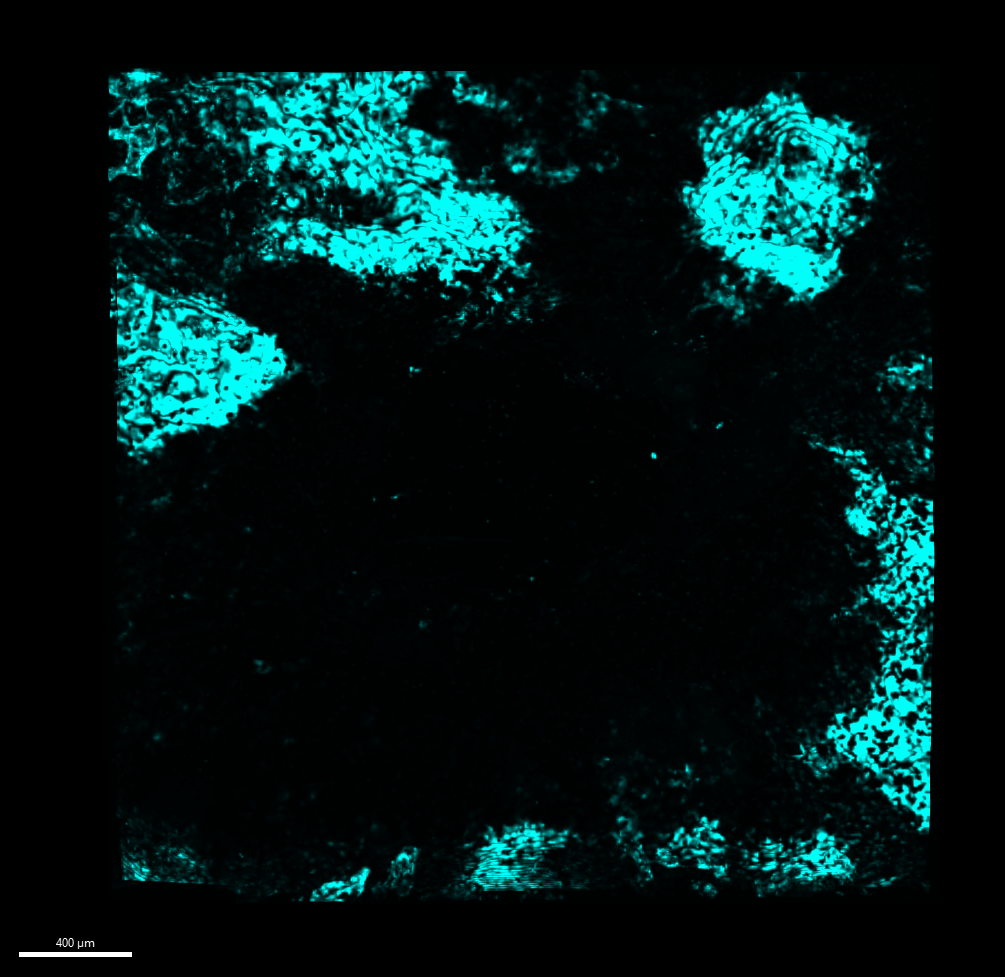

Supplement: Figure 2—source data 1. [file elife-83146-fig2-data1.zip › Figure 2/002 1mm day 1 flip for quan_gfp.tif]

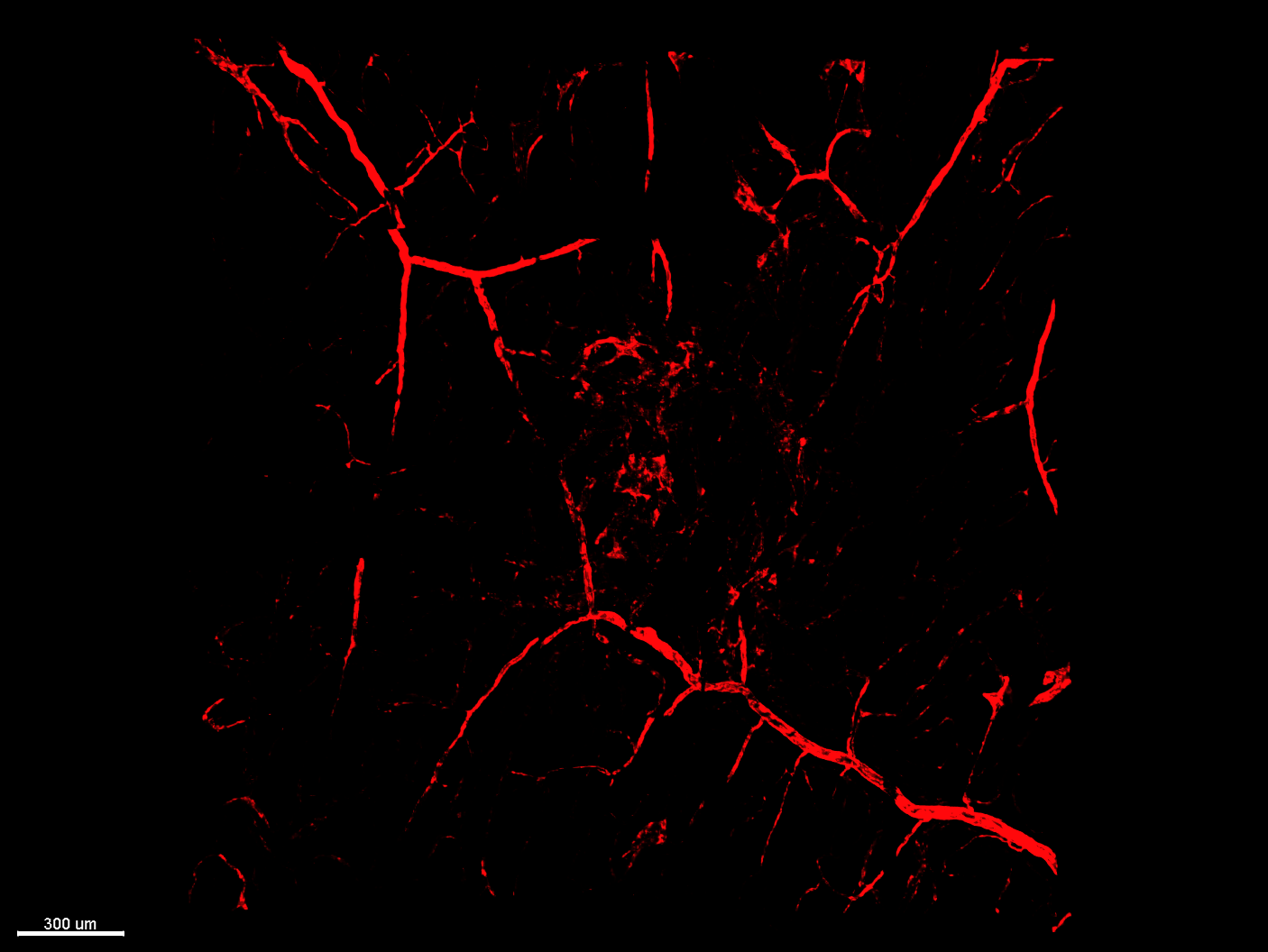

Supplement: Figure 2—source data 1. [file elife-83146-fig2-data1.zip › Figure 2/004 1mm day 3 cd31 only.tif]

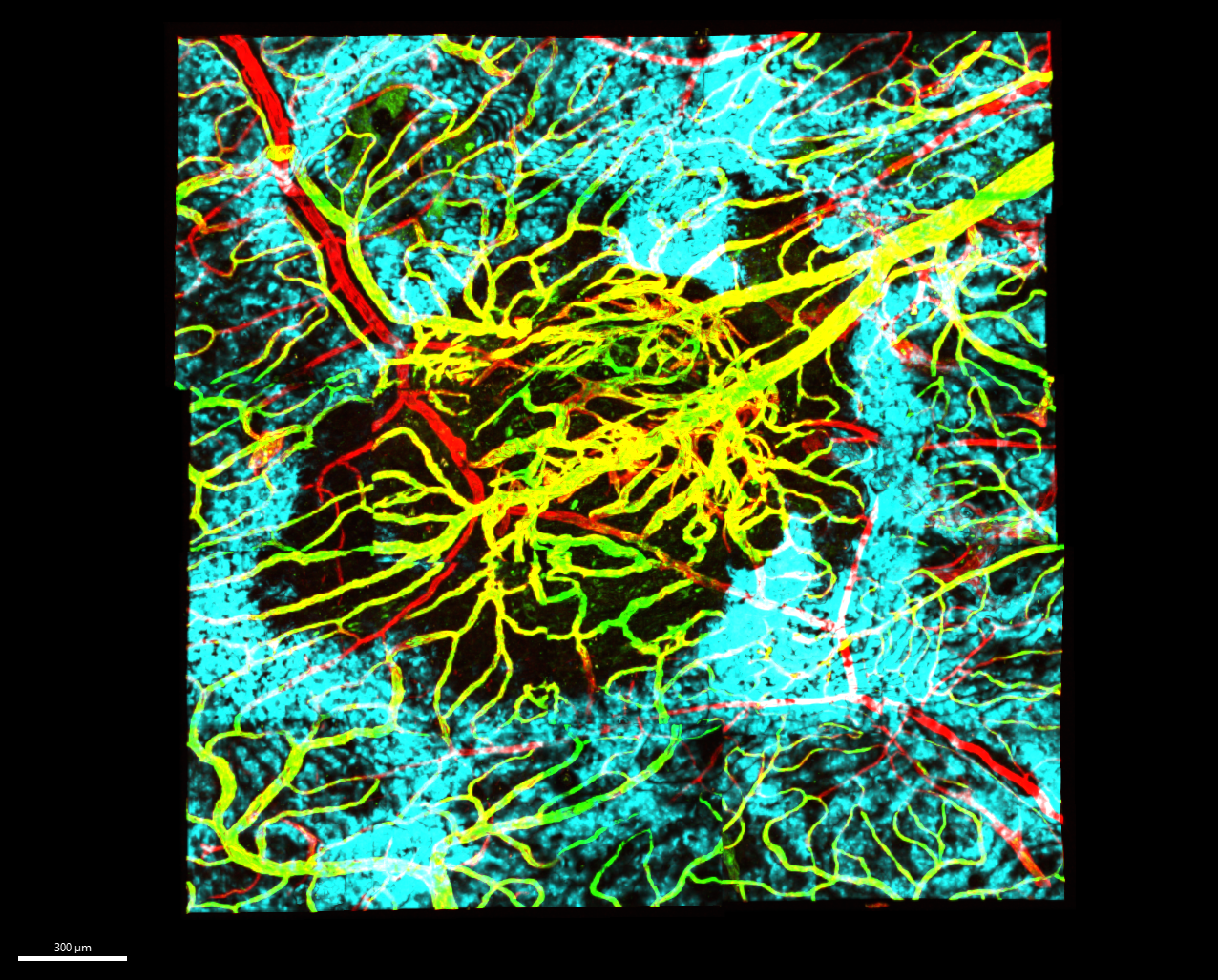

Supplement: Figure 2—source data 1. [file elife-83146-fig2-data1.zip › Figure 2/004 1mm day 3 flip for quan-40 slices 4x4_ endo cd31 gfp good.tif]

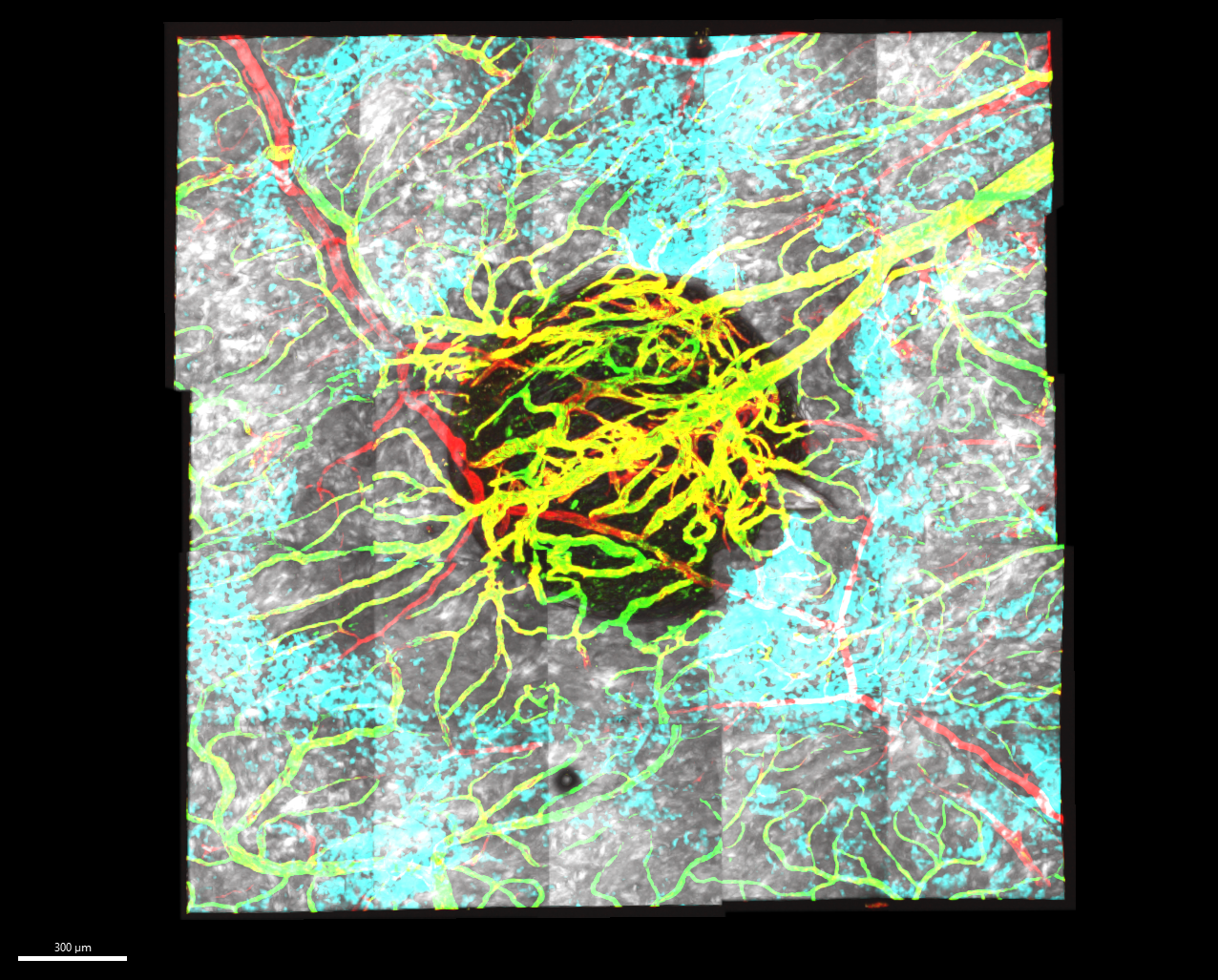

Supplement: Figure 2—source data 1. [file elife-83146-fig2-data1.zip › Figure 2/004 1mm day 3 flip for quan-40 slices 4x4_ endo cd31 gfp shg good.tif]

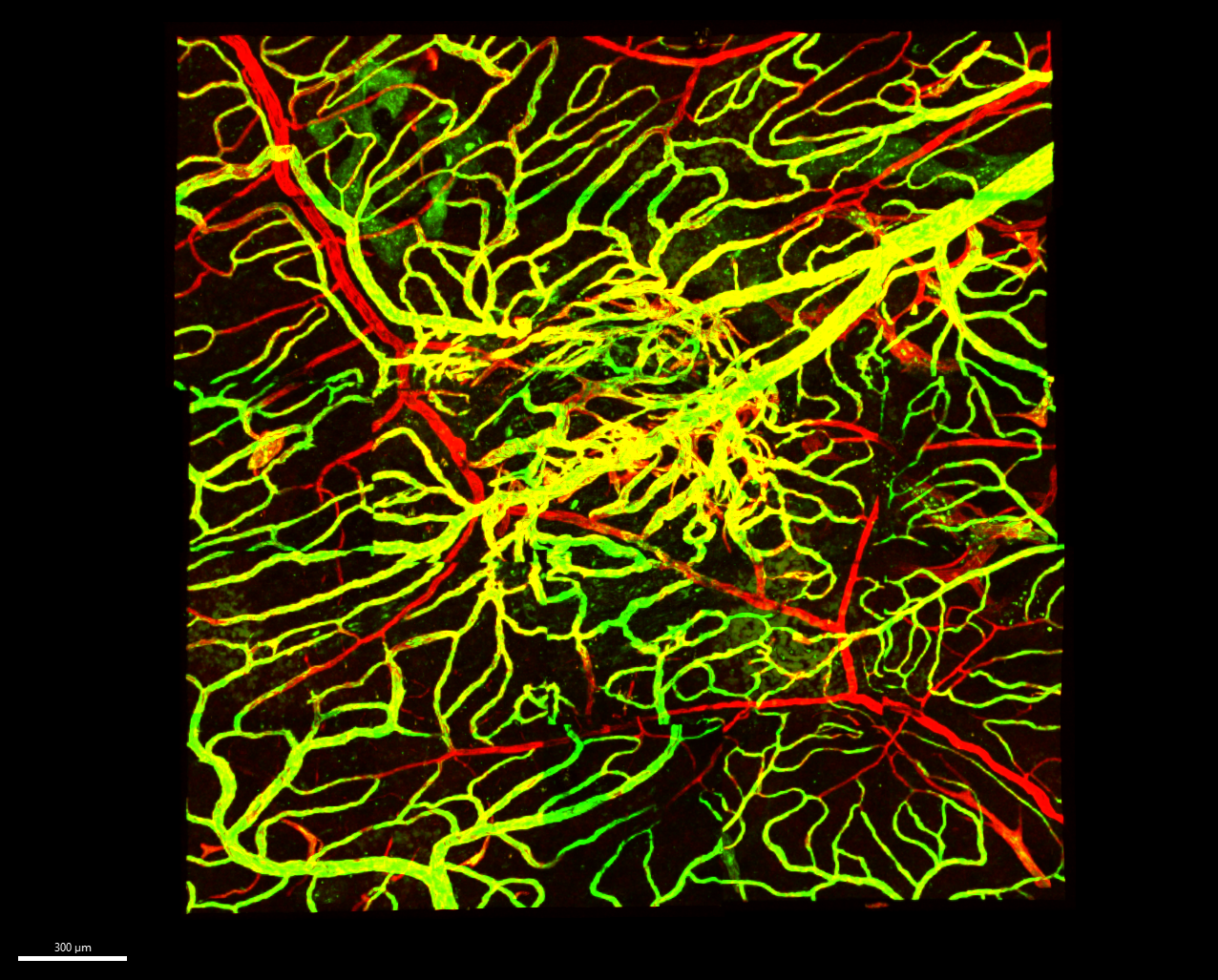

Supplement: Figure 2—source data 1. [file elife-83146-fig2-data1.zip › Figure 2/004 1mm day 3 flip for quan-40 slices 4x4_ endo cd31 shg good.tif]

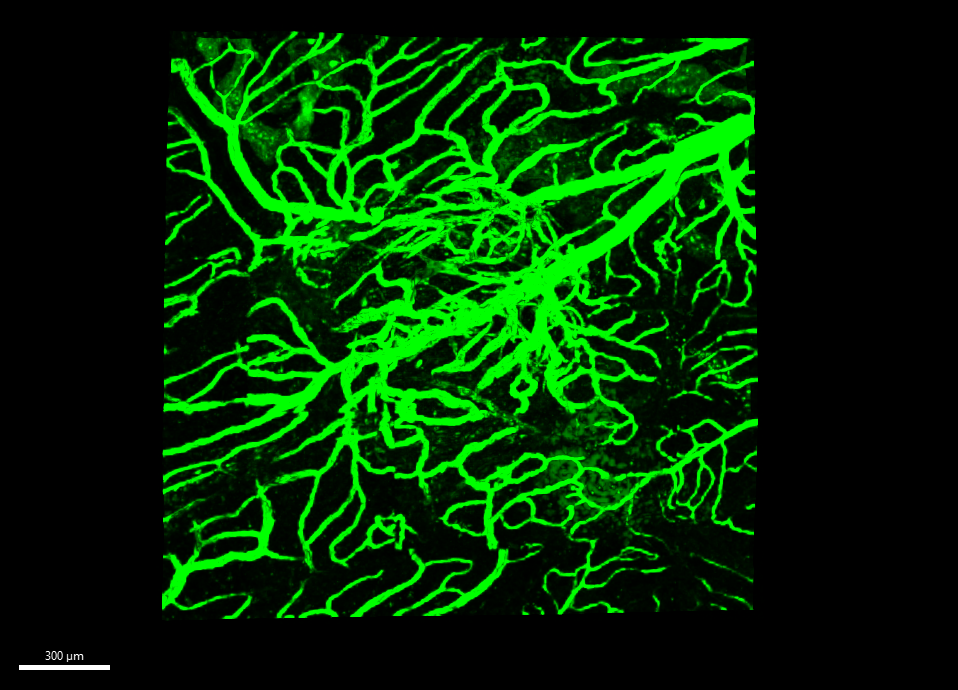

Supplement: Figure 2—source data 1. [file elife-83146-fig2-data1.zip › Figure 2/004 1mm day 3 flip for quan-40 slices 4x4_ endo.tif]

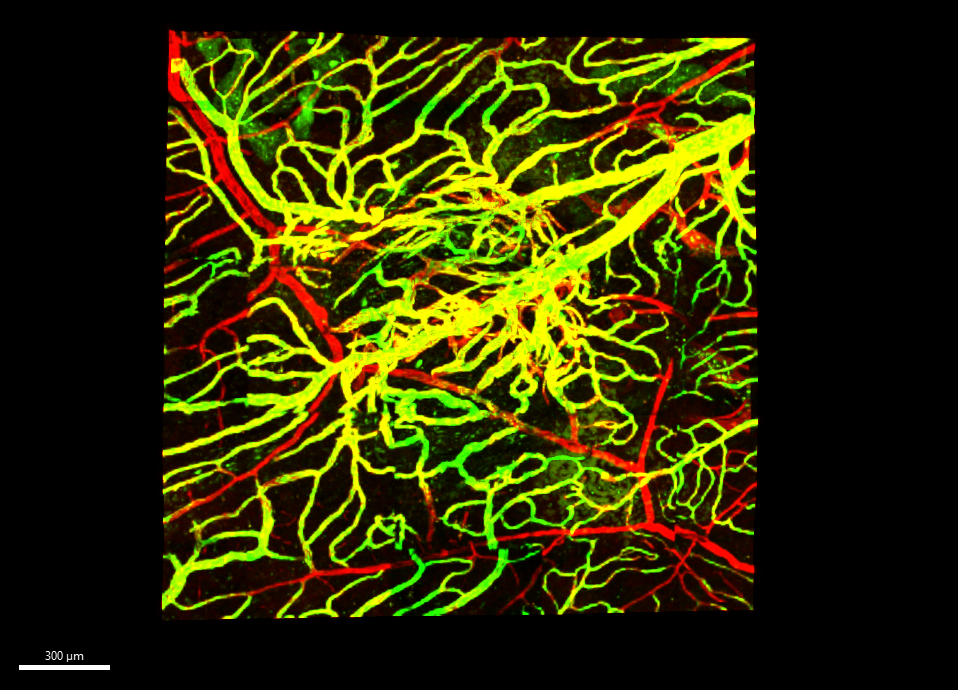

Supplement: Figure 2—source data 1. [file elife-83146-fig2-data1.zip › Figure 2/004 1mm day 3 flip for quan-40 slices 4x4_cd31 endo.tif]

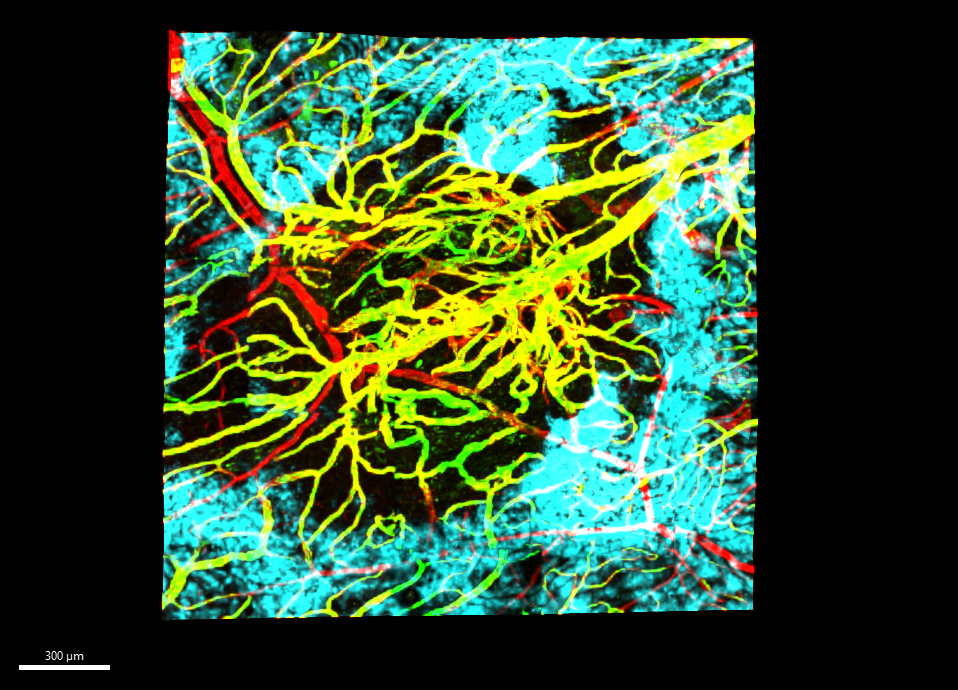

Supplement: Figure 2—source data 1. [file elife-83146-fig2-data1.zip › Figure 2/004 1mm day 3 flip for quan-40 slices 4x4_gfp cd31 endo.tif]

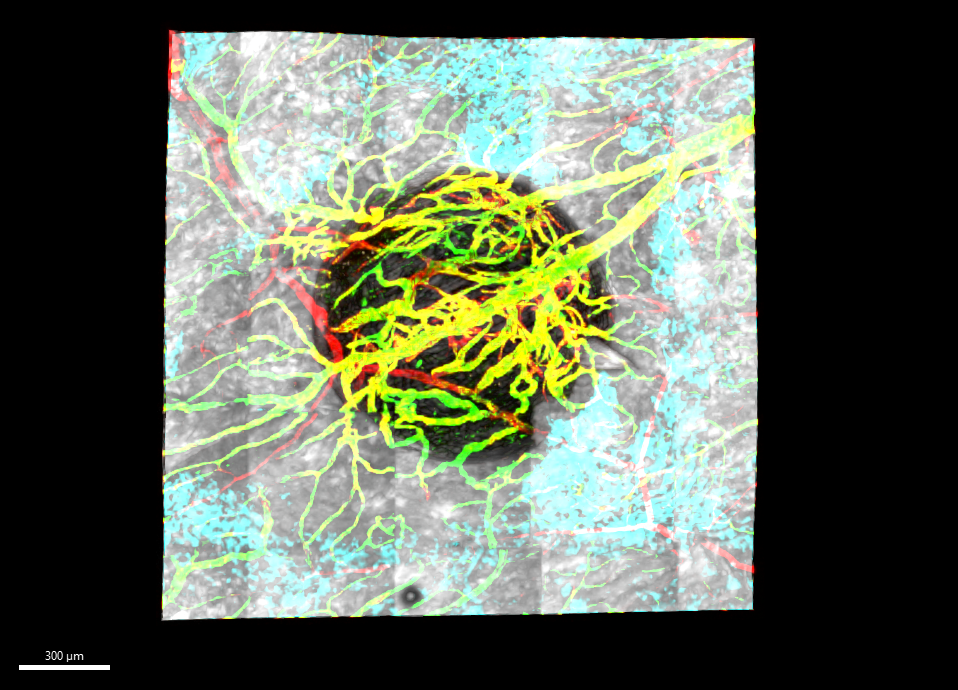

Supplement: Figure 2—source data 1. [file elife-83146-fig2-data1.zip › Figure 2/004 1mm day 3 flip for quan-40 slices 4x4_shg cd31 endo gfp.tif]

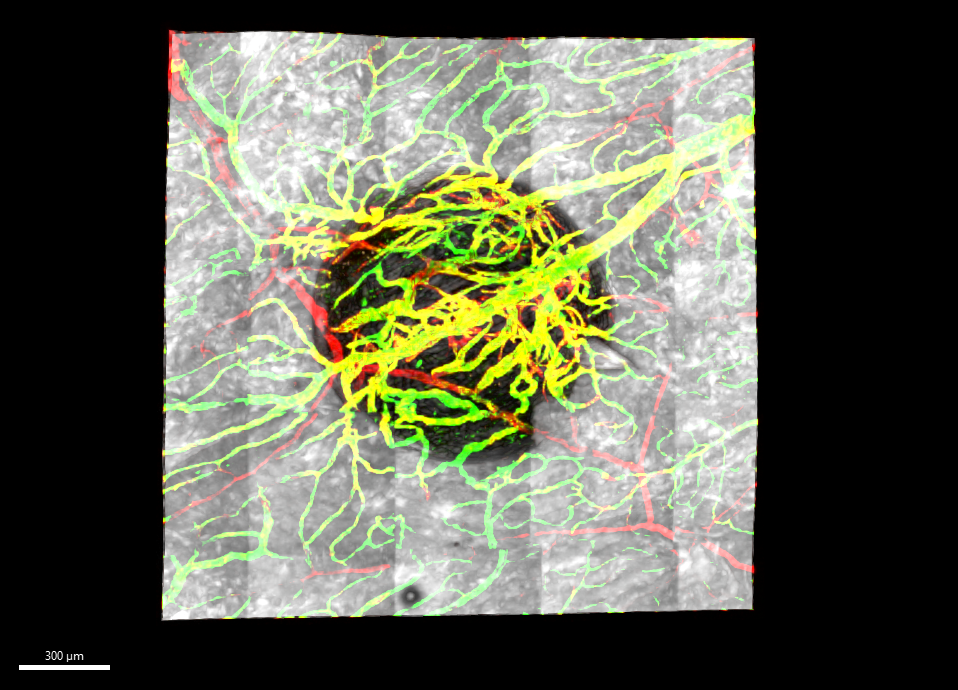

Supplement: Figure 2—source data 1. [file elife-83146-fig2-data1.zip › Figure 2/004 1mm day 3 flip for quan-40 slices 4x4_shg cd31 endo.png]

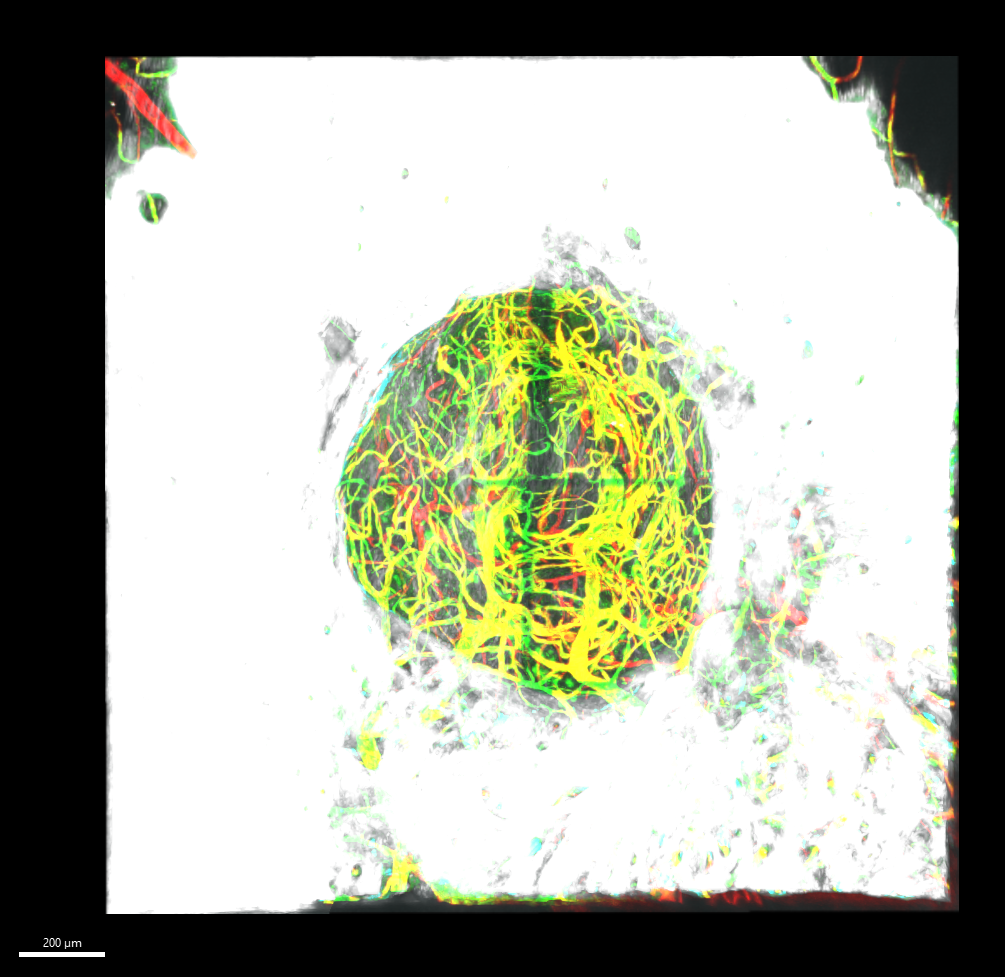

Supplement: Figure 2—source data 1. [file elife-83146-fig2-data1.zip › Figure 2/165 1mm day 21 side 1 flip cd31 endo gfp-shg.tif]

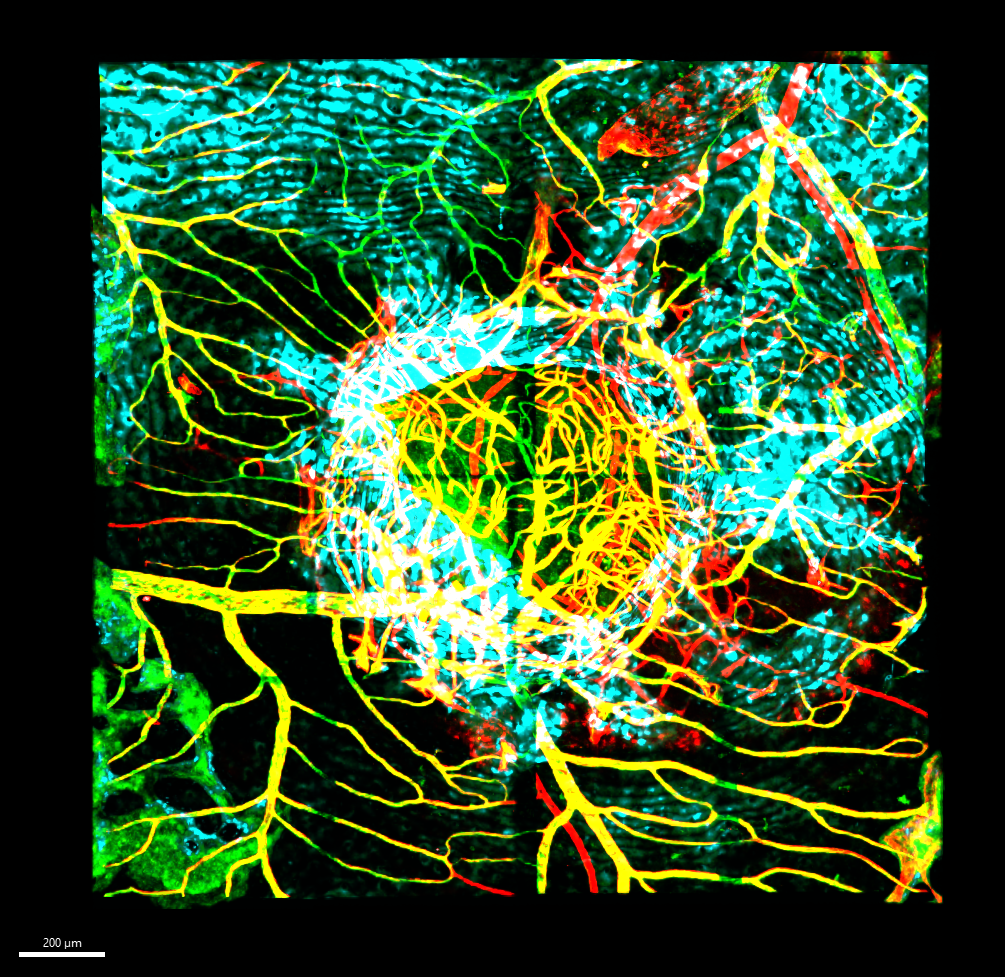

Supplement: Figure 2—source data 1. [file elife-83146-fig2-data1.zip › Figure 2/165 1mm day 21 side 2 flip cd31 endo gfp.tif]

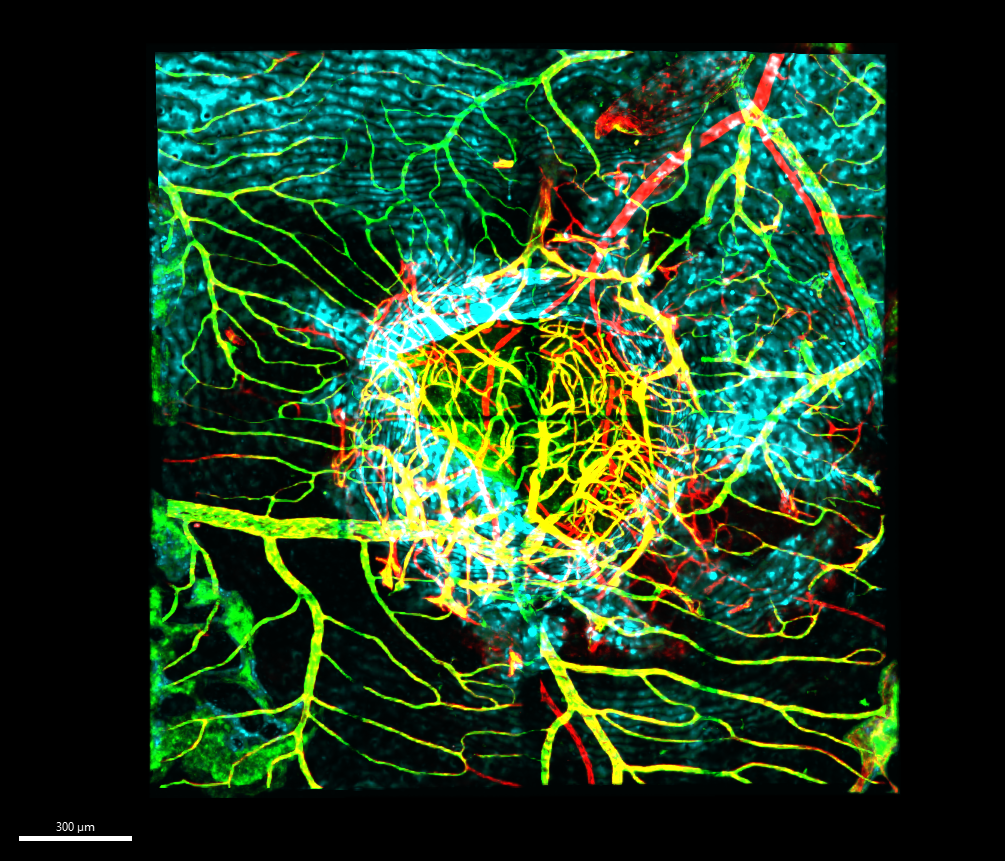

Supplement: Figure 2—source data 1. [file elife-83146-fig2-data1.zip › Figure 2/165 1mm day 21 side 2 flip cd31 endo gfp-2.tif]

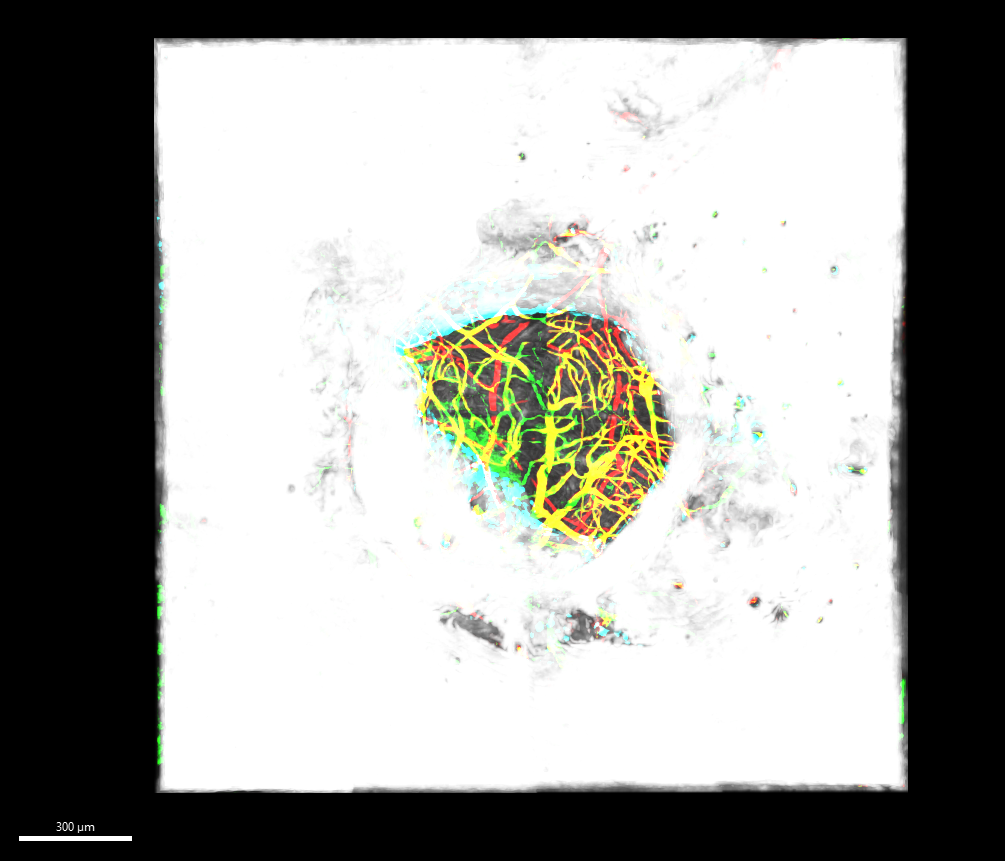

Supplement: Figure 2—source data 1. [file elife-83146-fig2-data1.zip › Figure 2/165 day 21 side 1 flip for quan-40 slices 4x4_cd31 endo gfp shg-2.tif]

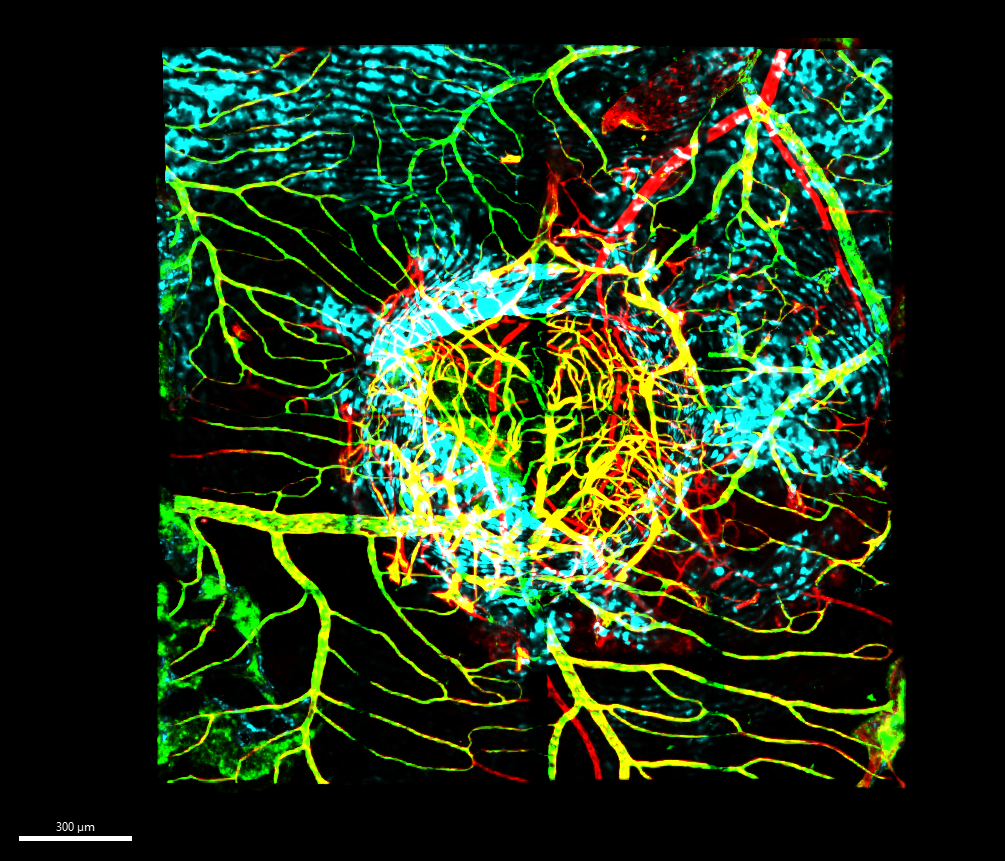

Supplement: Figure 2—source data 1. [file elife-83146-fig2-data1.zip › Figure 2/165 day 21 side 1 flip for quan-40 slices 4x4_cd31 endo gfp-2.tif]

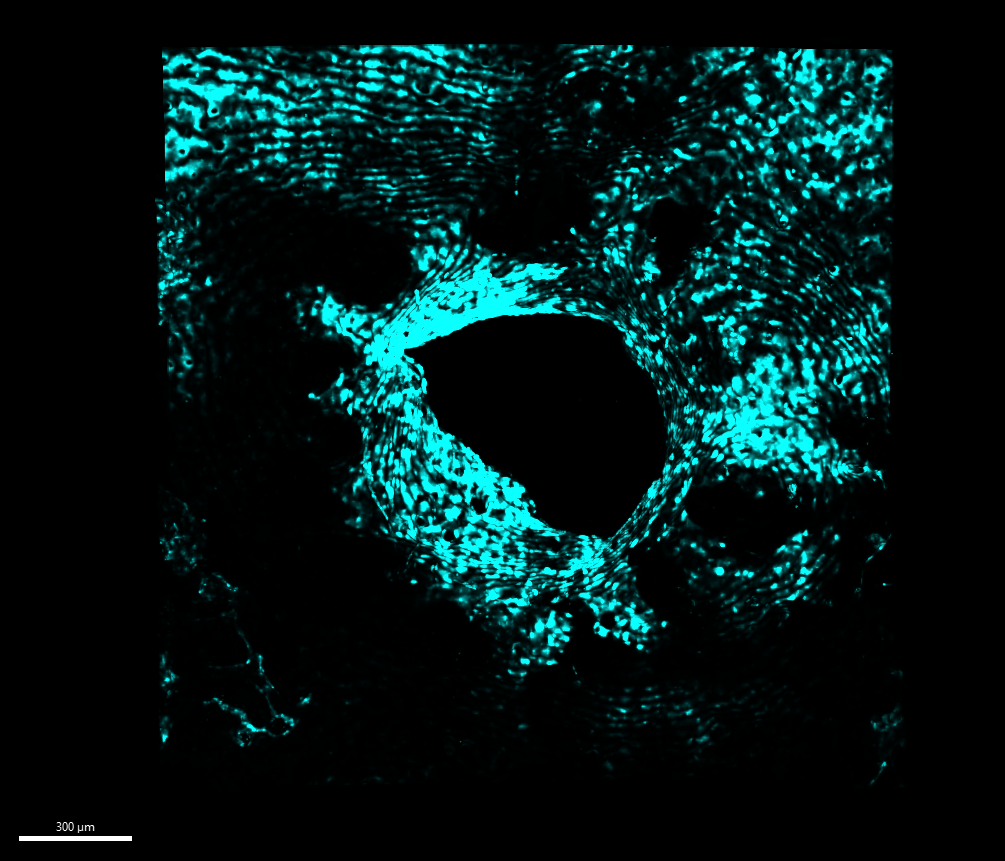

Supplement: Figure 2—source data 1. [file elife-83146-fig2-data1.zip › Figure 2/165 day 21 side 1 flip for quan-40 slices 4x4_gfp-2.tif]

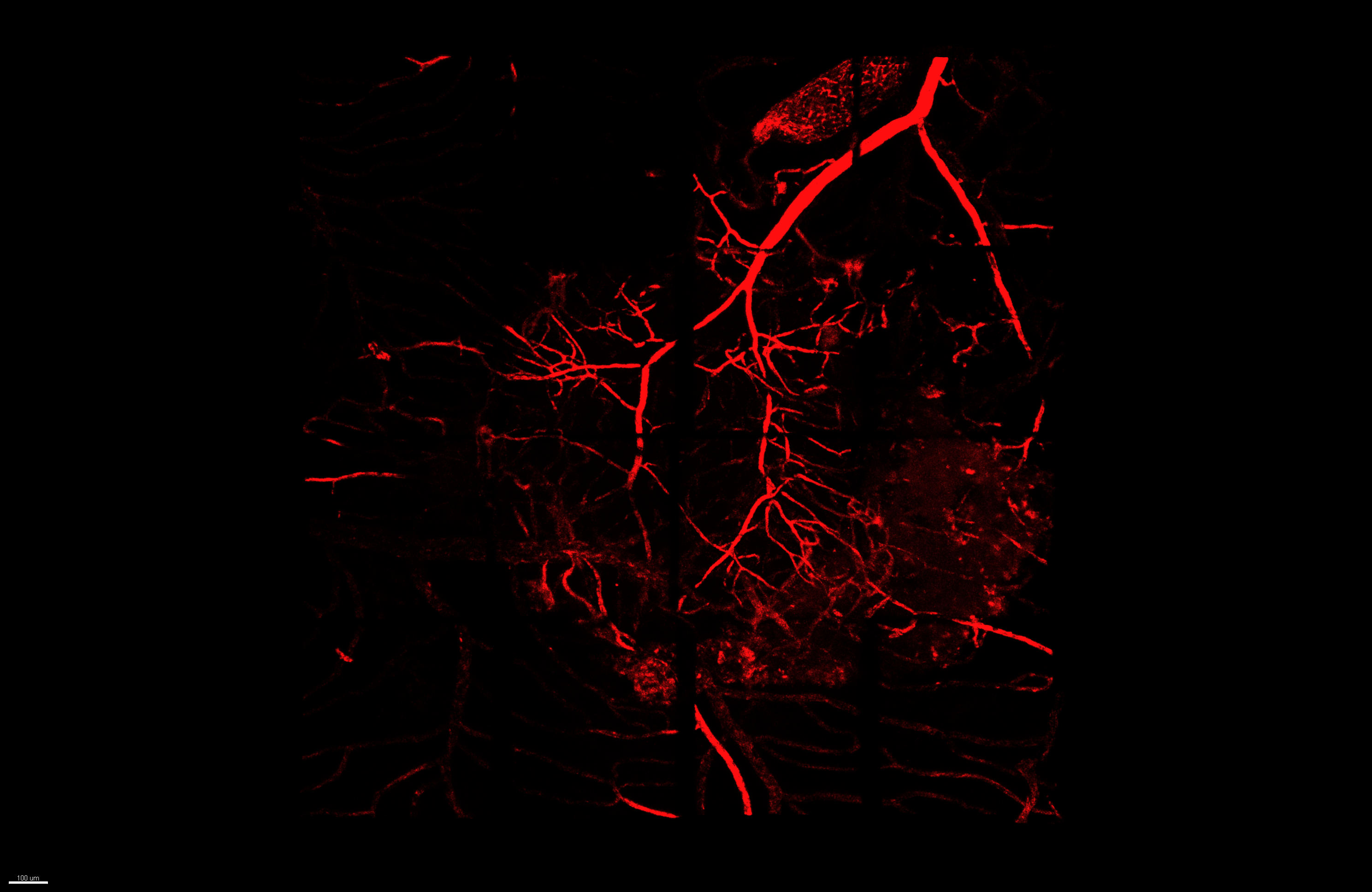

Supplement: Figure 2—source data 1. [file elife-83146-fig2-data1.zip › Figure 2/165 day 21 side 2 flip 40 slices cd31only.tif]

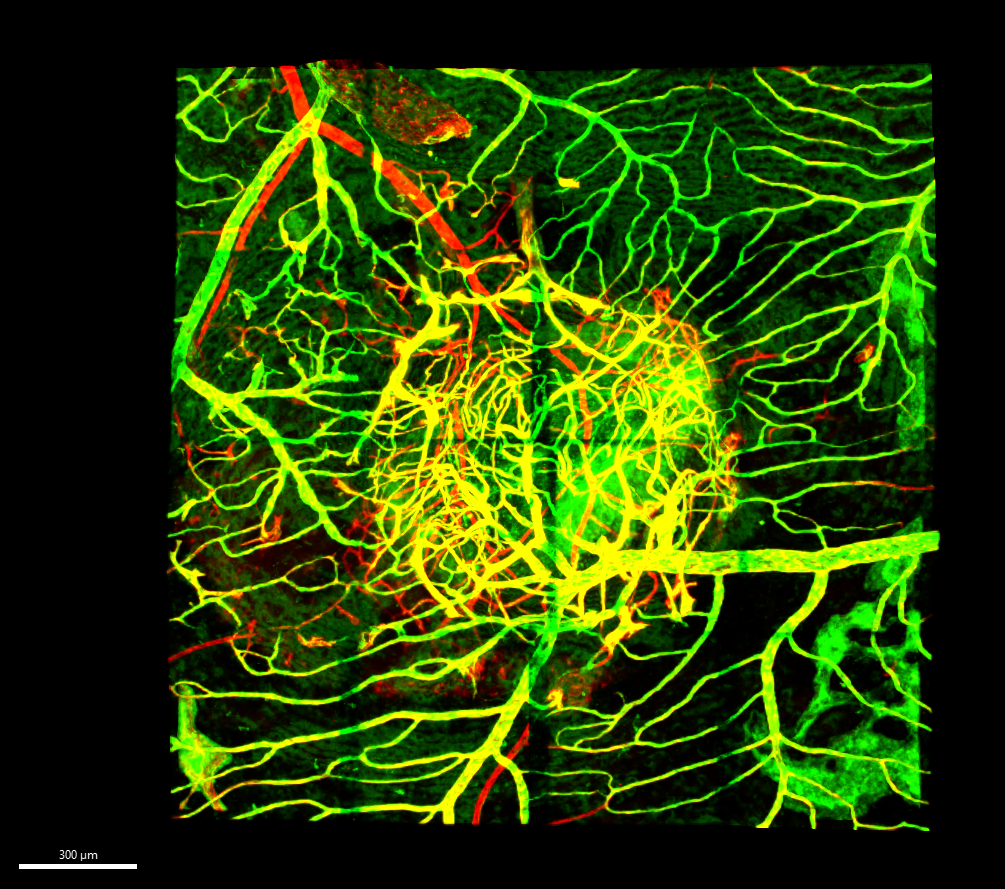

Supplement: Figure 2—source data 1. [file elife-83146-fig2-data1.zip › Figure 2/165 day 21 side 2 flip for quan-40 slices 4x4_ enod cd31.tif]

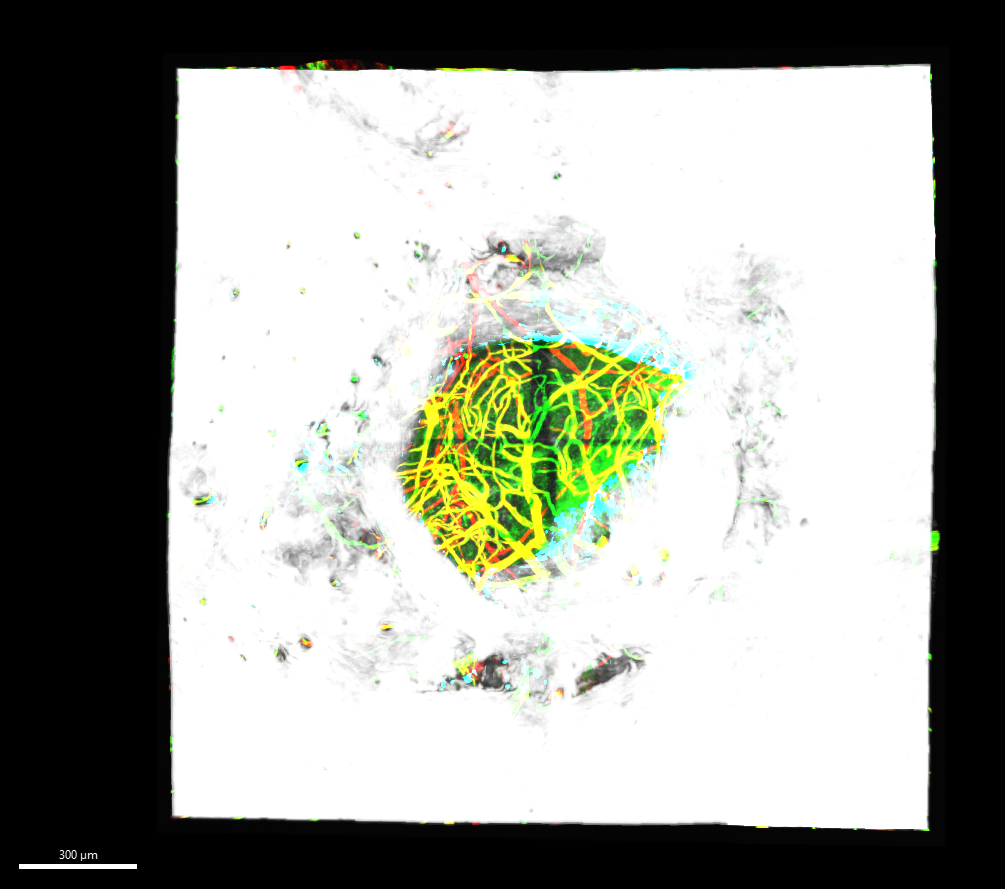

Supplement: Figure 2—source data 1. [file elife-83146-fig2-data1.zip › Figure 2/165 day 21 side 2 flip for quan-40 slices 4x4_ gfp enod cd31 shg.tif]

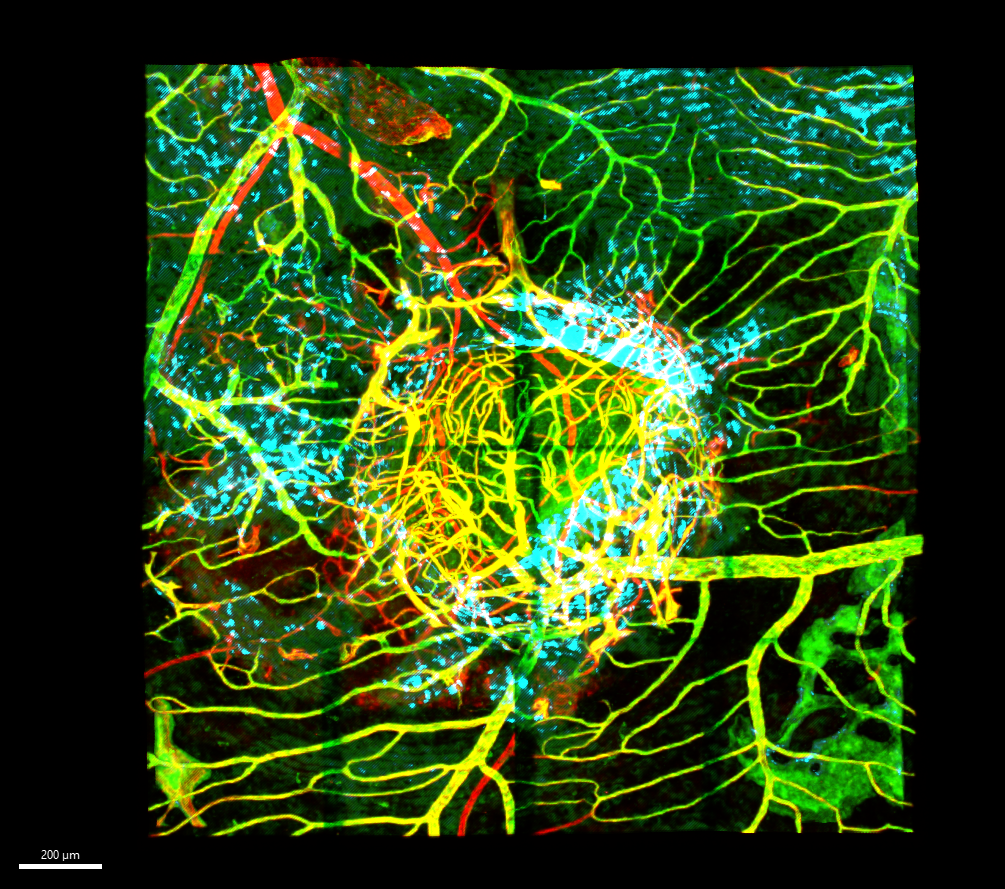

Supplement: Figure 2—source data 1. [file elife-83146-fig2-data1.zip › Figure 2/165 day 21 side 2 flip for quan-40 slices 4x4_ gfp enod cd31.tif]

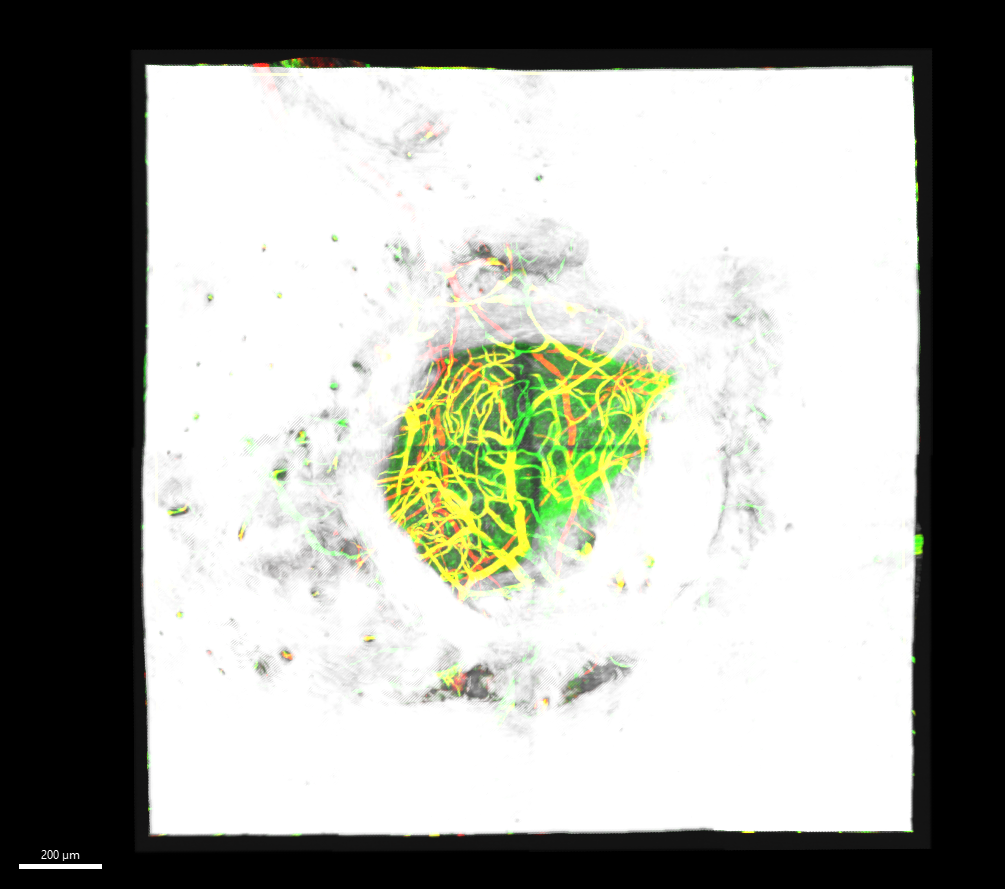

Supplement: Figure 2—source data 1. [file elife-83146-fig2-data1.zip › Figure 2/165 day 21 side 2 flip for quan-40 slices 4x4_.png]

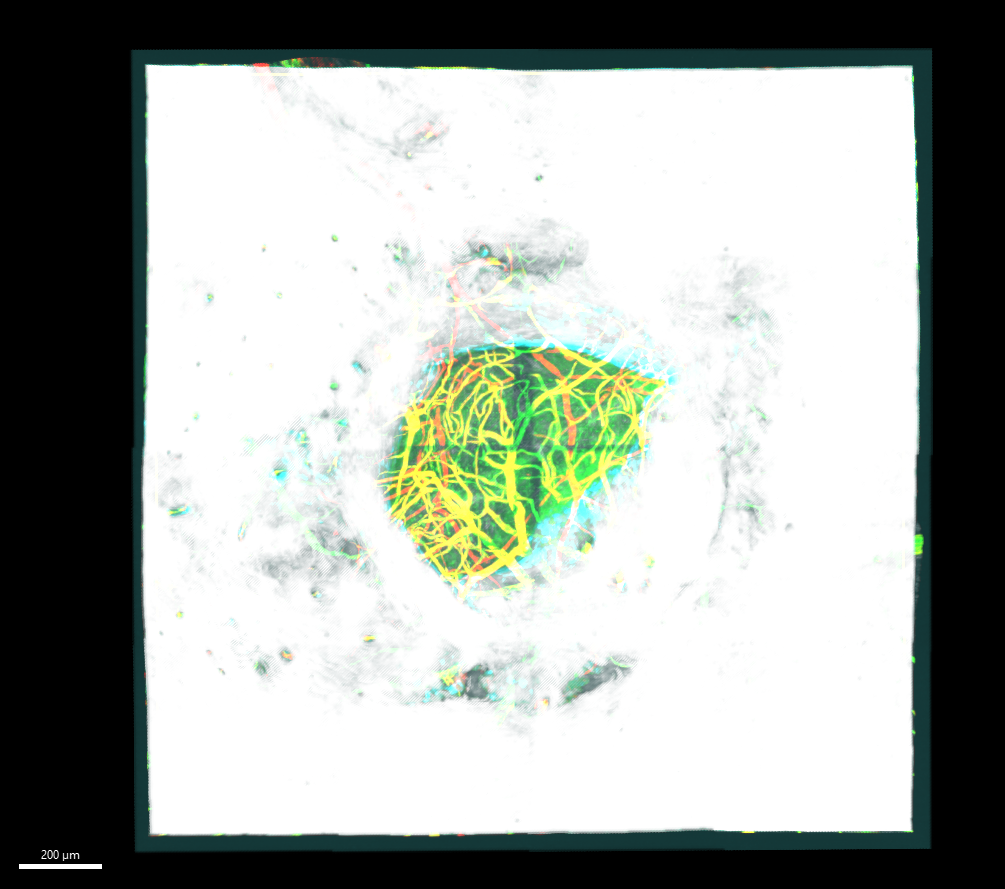

Supplement: Figure 2—source data 1. [file elife-83146-fig2-data1.zip › Figure 2/165 day 21 side 2 flip for quan-40 slices 4x4_shg gfp enod cd31.tif]
