# Supplementary figures and images for: High-resolution imaging of the osteogenic and angiogenic interface at the site of murine cranial bone defect repair via multiphoton microscopy (part 2 of 2)
Source: eLife. 2022 Nov 3;11:e83146. doi: 10.7554/eLife.83146 (PMC9678361; doi:10.7554/eLife.83146)

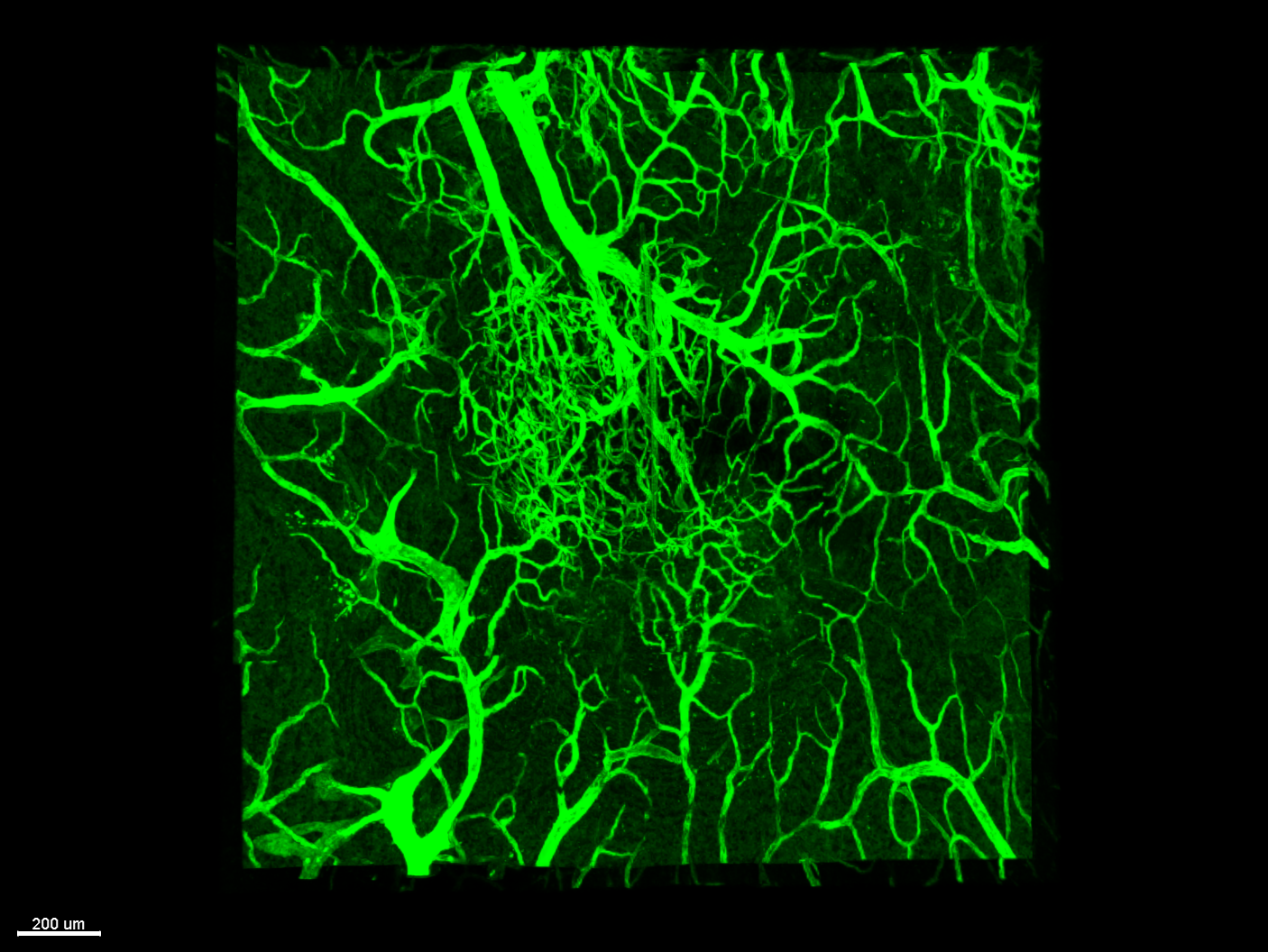

Supplement: Figure 2—source data 1. [file elife-83146-fig2-data1.zip › Figure 2/RE013 day 14 side 1 flip endo.tif]

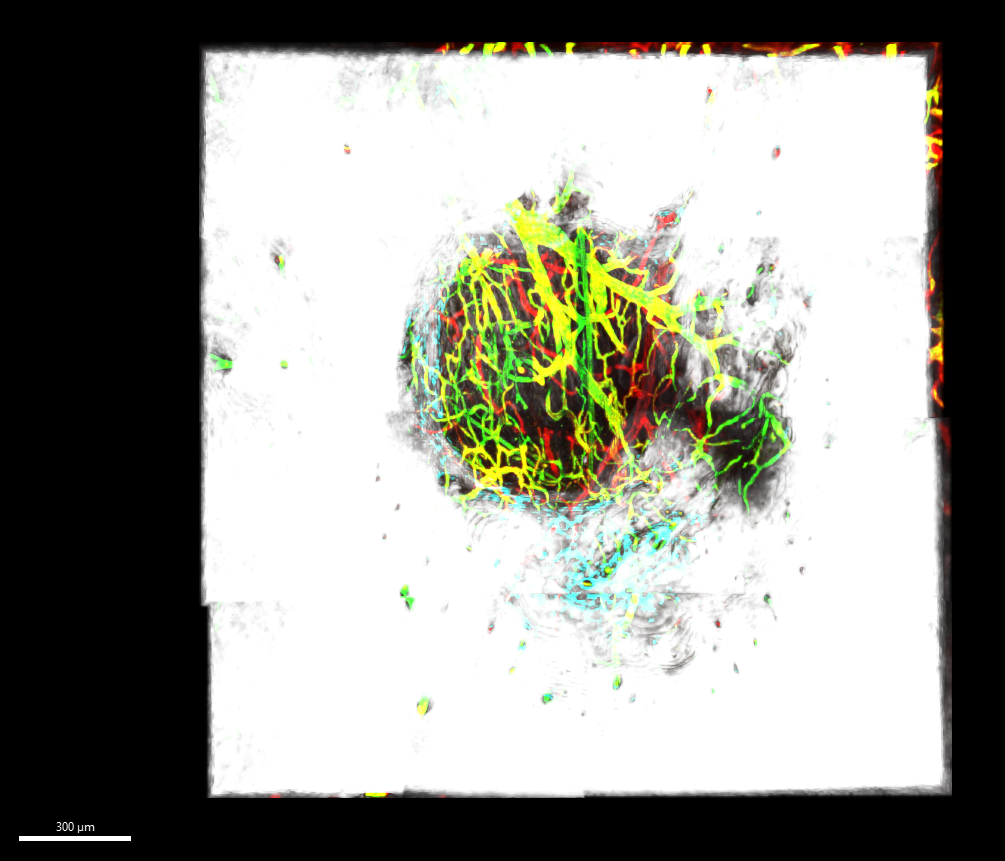

Supplement: Figure 2—source data 1. [file elife-83146-fig2-data1.zip › Figure 2/RE013 day 14 side 1 flip for quan-40 slices 4x4 endo cd31 gfp.tif]

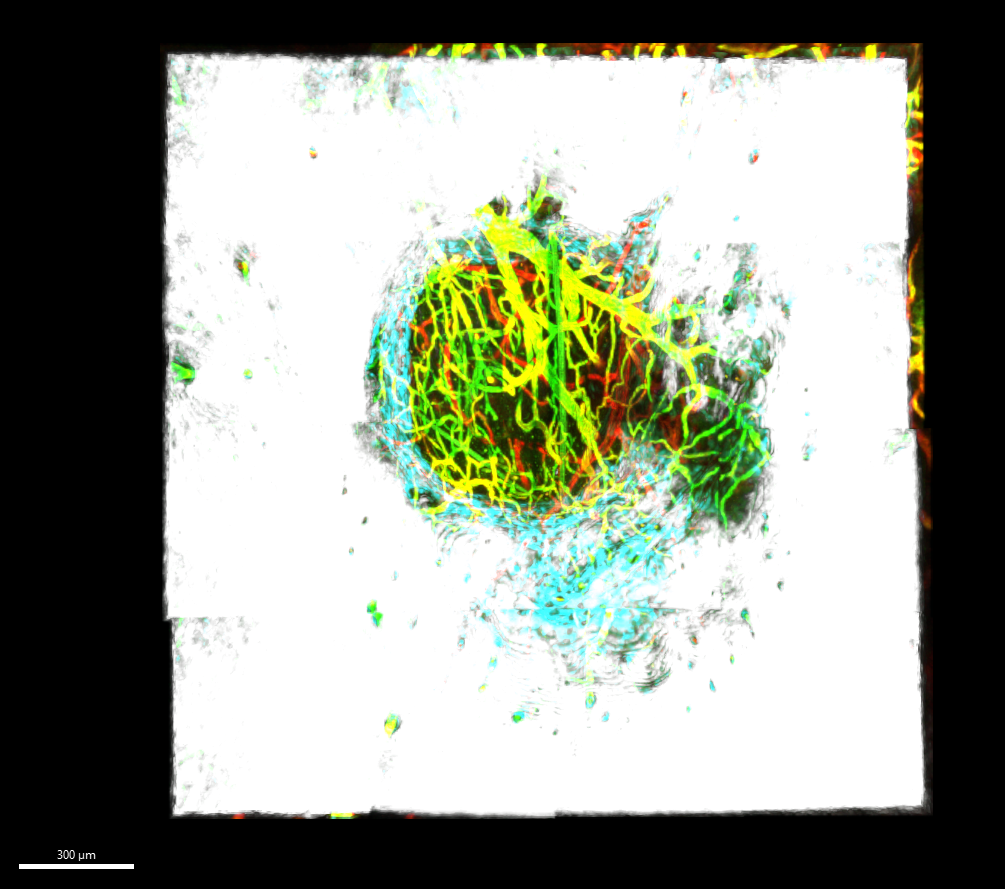

Supplement: Figure 2—source data 1. [file elife-83146-fig2-data1.zip › Figure 2/RE013 day 14 side 1 flip for quan-40 slices 4x4 endo gfp cd31 shg.tif]

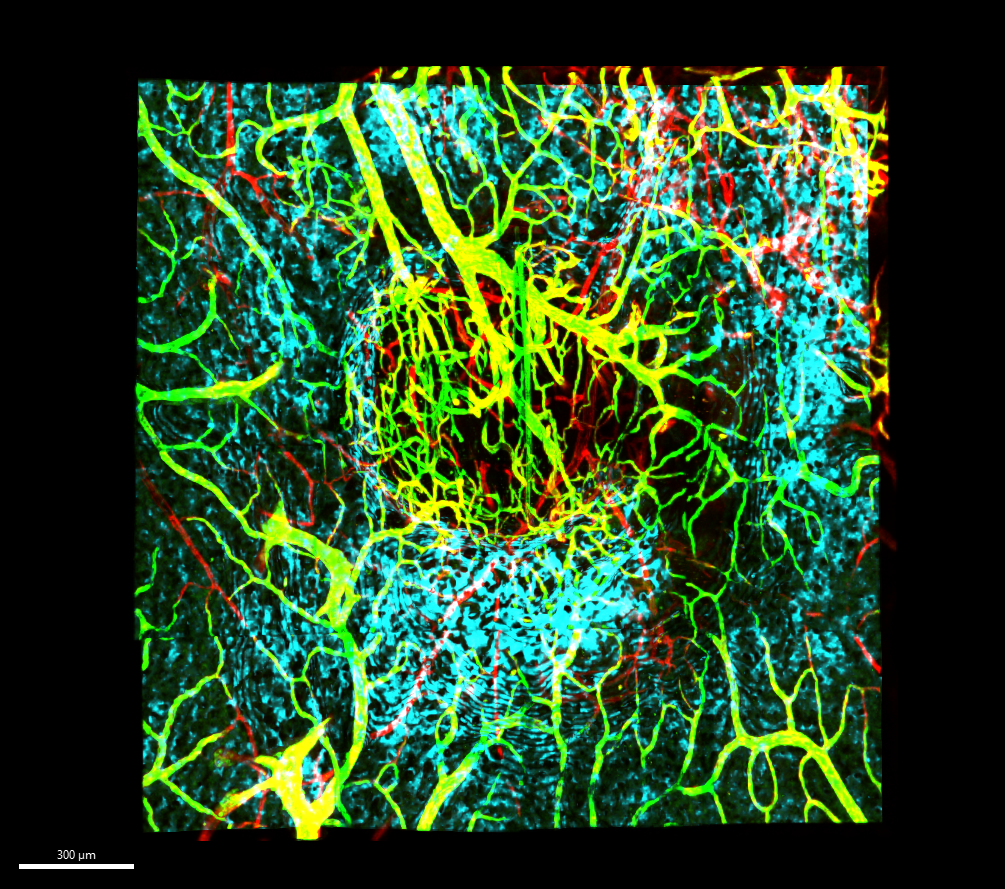

Supplement: Figure 2—source data 1. [file elife-83146-fig2-data1.zip › Figure 2/RE013 day 14 side 1 flip for quan-40 slices 4x4 endo gfp cd31.png]

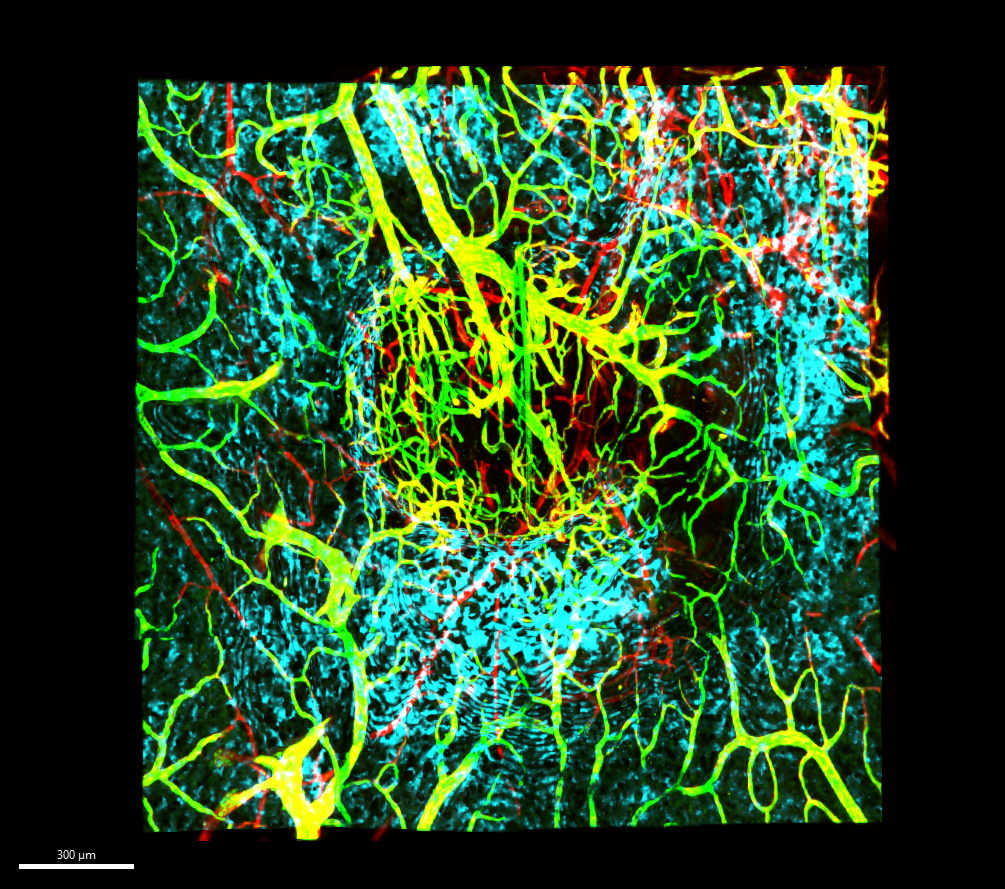

Supplement: Figure 2—source data 1. [file elife-83146-fig2-data1.zip › Figure 2/RE013 day 14 side 1 flip for quan-40 slices 4x4 endo gfp cd31.tif]

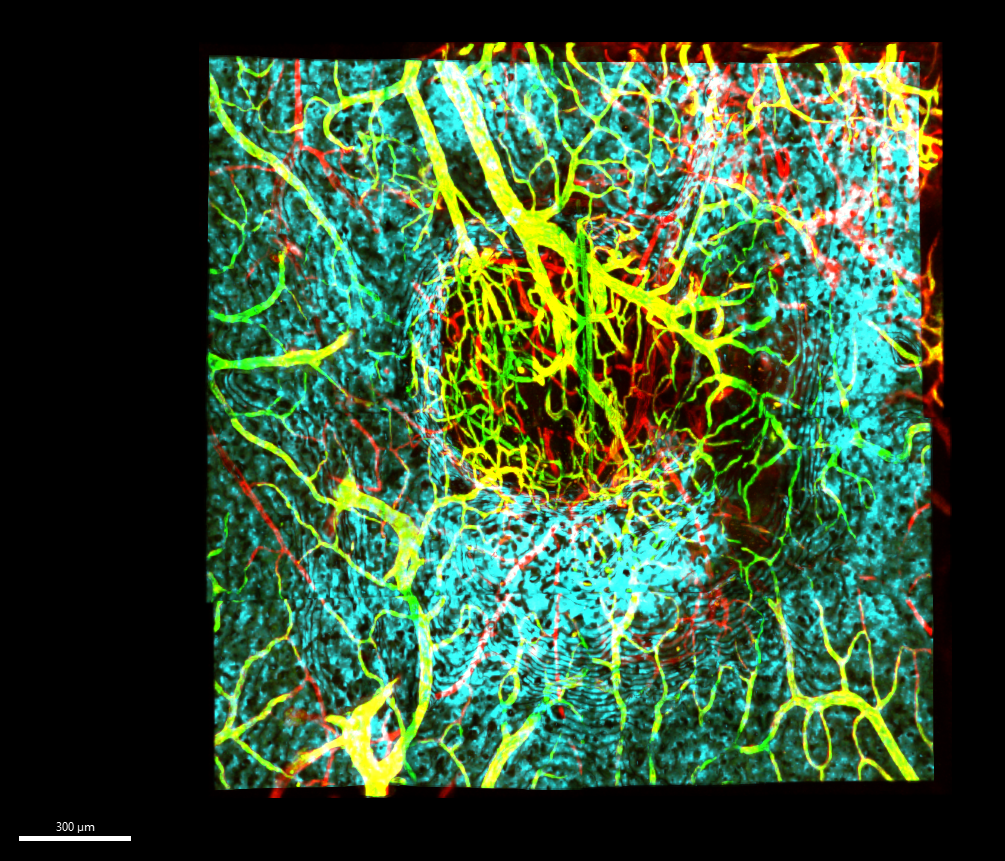

Supplement: Figure 2—source data 1. [file elife-83146-fig2-data1.zip › Figure 2/RE013 day 14 side 1 flip for quan-40 slices 4x4 gfp endo cd31-3.tif]

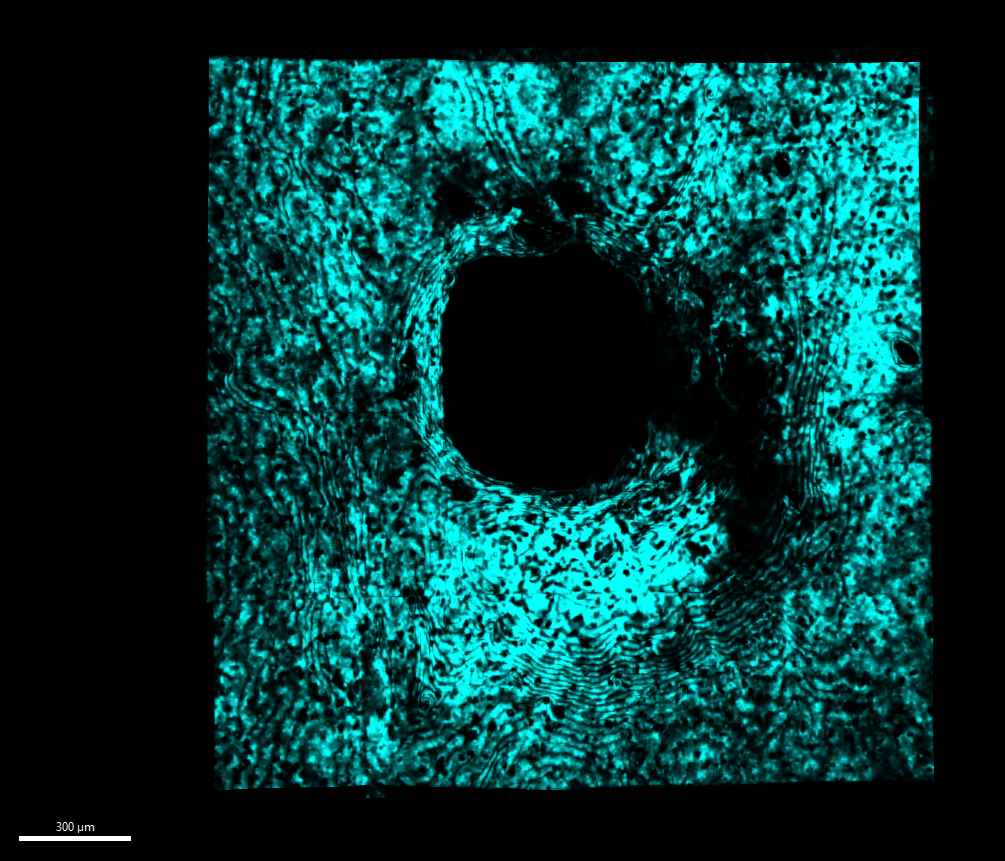

Supplement: Figure 2—source data 1. [file elife-83146-fig2-data1.zip › Figure 2/RE013 day 14 side 1 flip for quan-40 slices 4x4 gfp-3.tif]

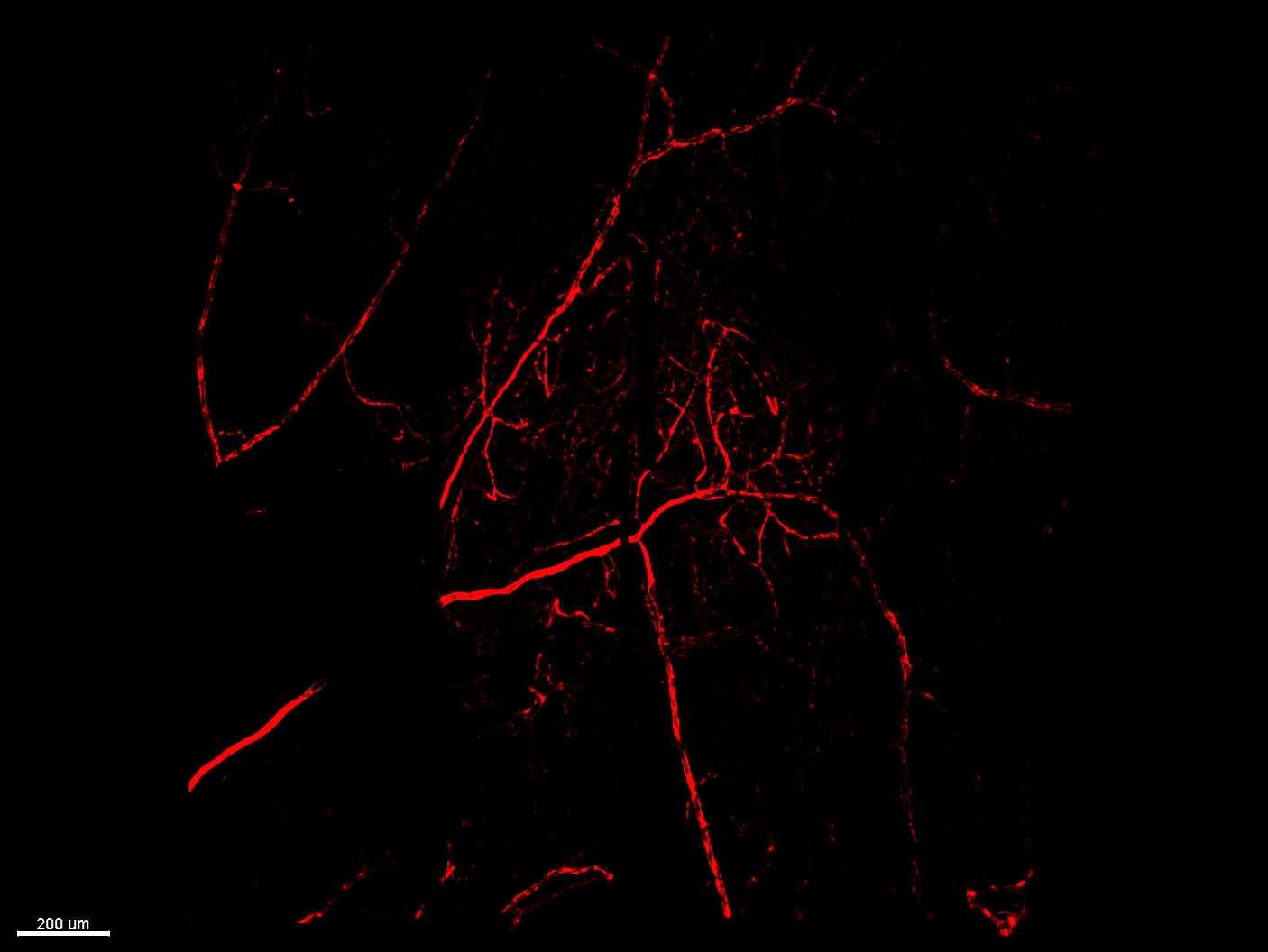

Supplement: Figure 2—source data 1. [file elife-83146-fig2-data1.zip › Figure 2/RE013 day 14 side 2 flip cd31 only.tif]

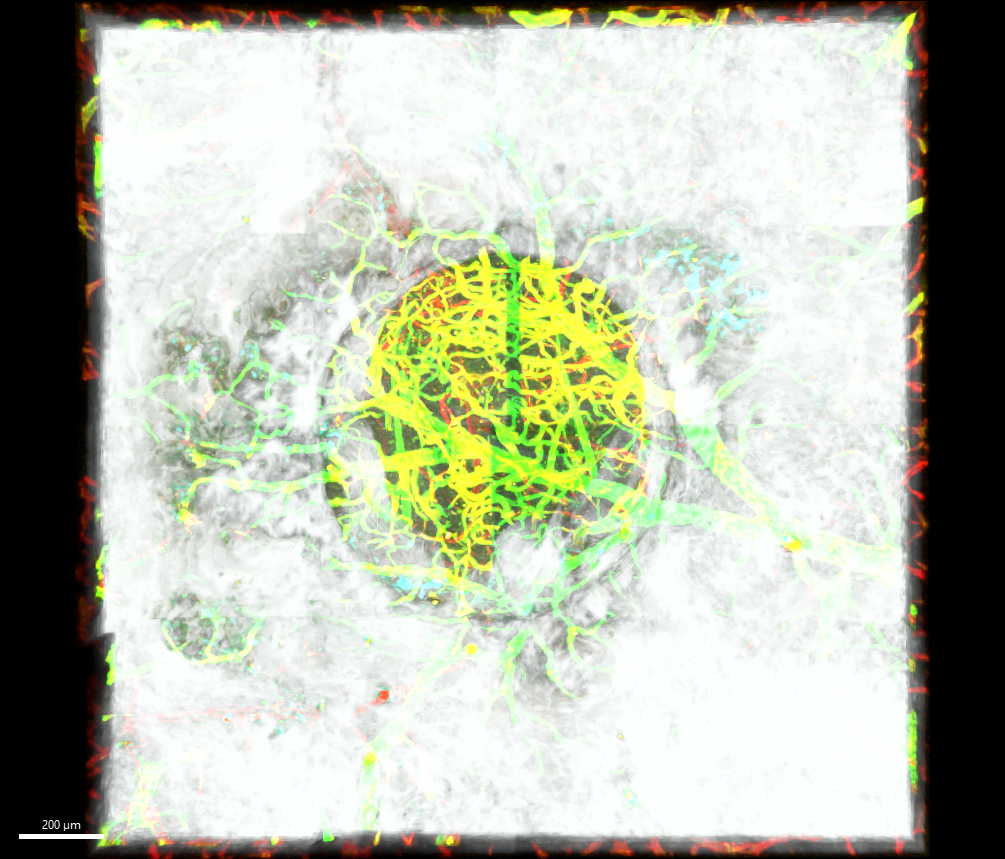

Supplement: Figure 2—source data 1. [file elife-83146-fig2-data1.zip › Figure 2/RE013-day 14 side 2 flip Composite CD31 and Endo shg gfp.tif]

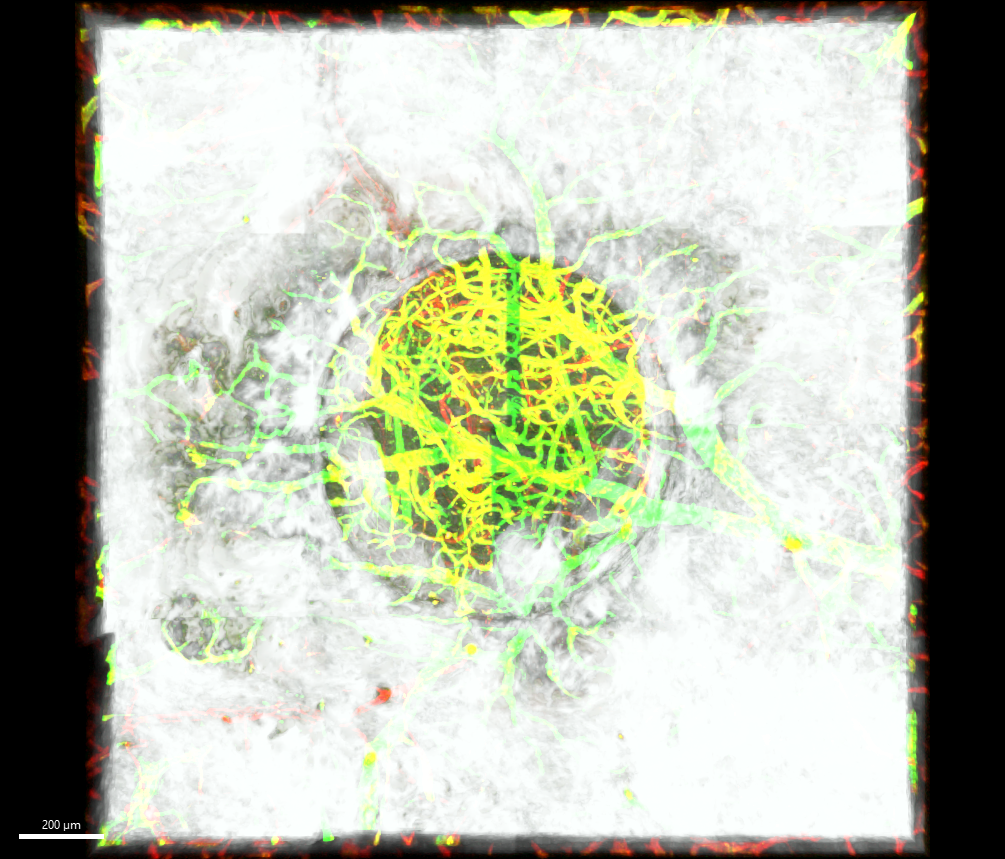

Supplement: Figure 2—source data 1. [file elife-83146-fig2-data1.zip › Figure 2/RE013-day 14 side 2 flip Composite CD31 and Endo shg.tif]

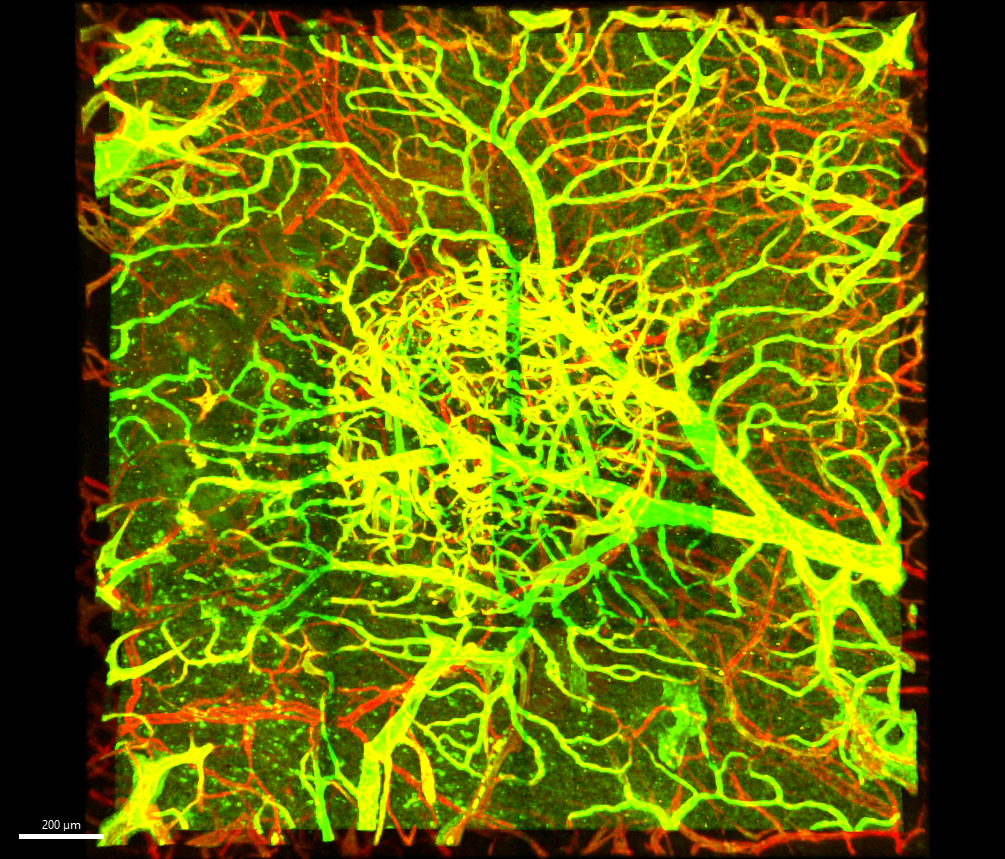

Supplement: Figure 2—source data 1. [file elife-83146-fig2-data1.zip › Figure 2/RE013-day 14 side 2 flip Composite CD31 and Endo.tif]

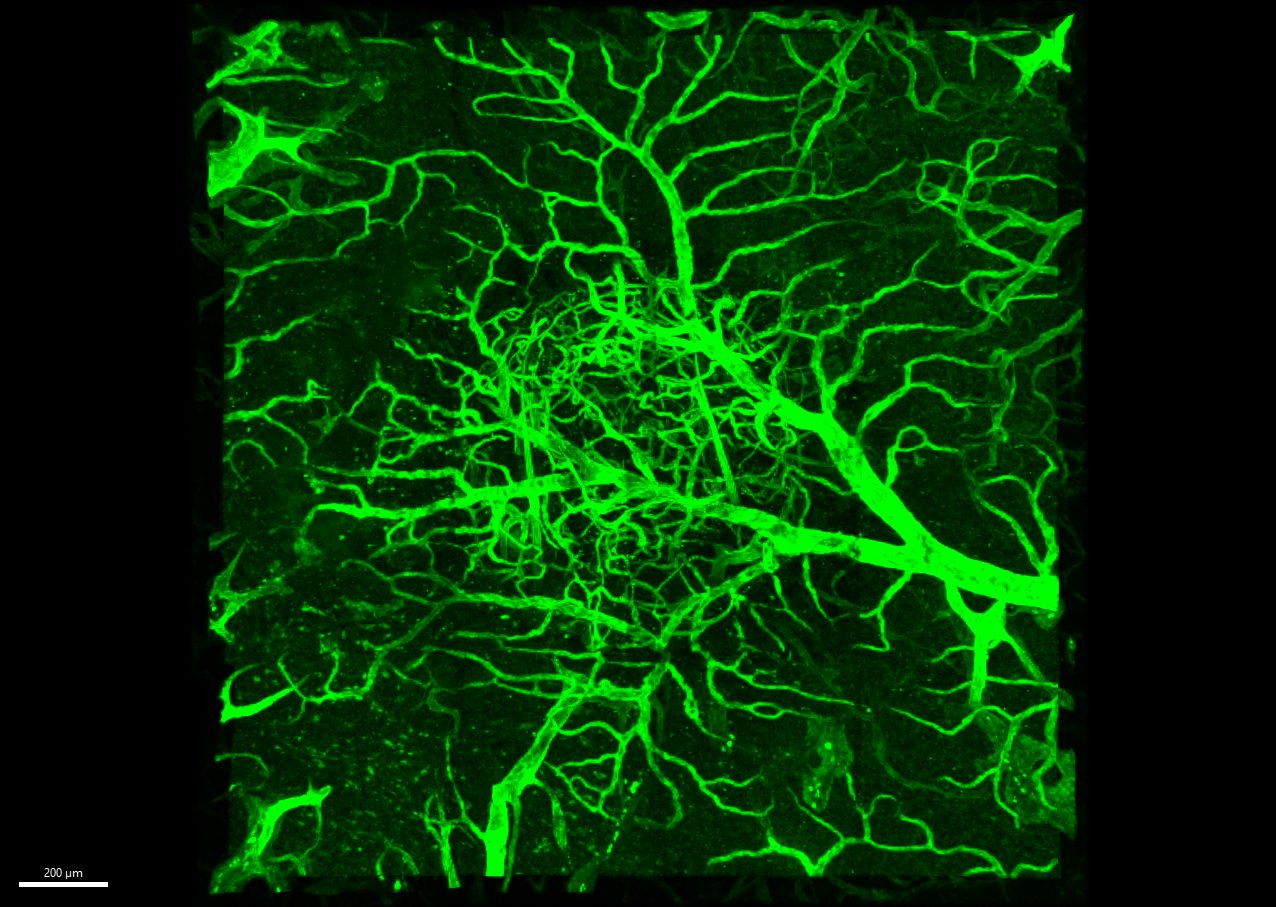

Supplement: Figure 2—source data 1. [file elife-83146-fig2-data1.zip › Figure 2/RE013-day 14 side 2 flip Composite Endo.tif]

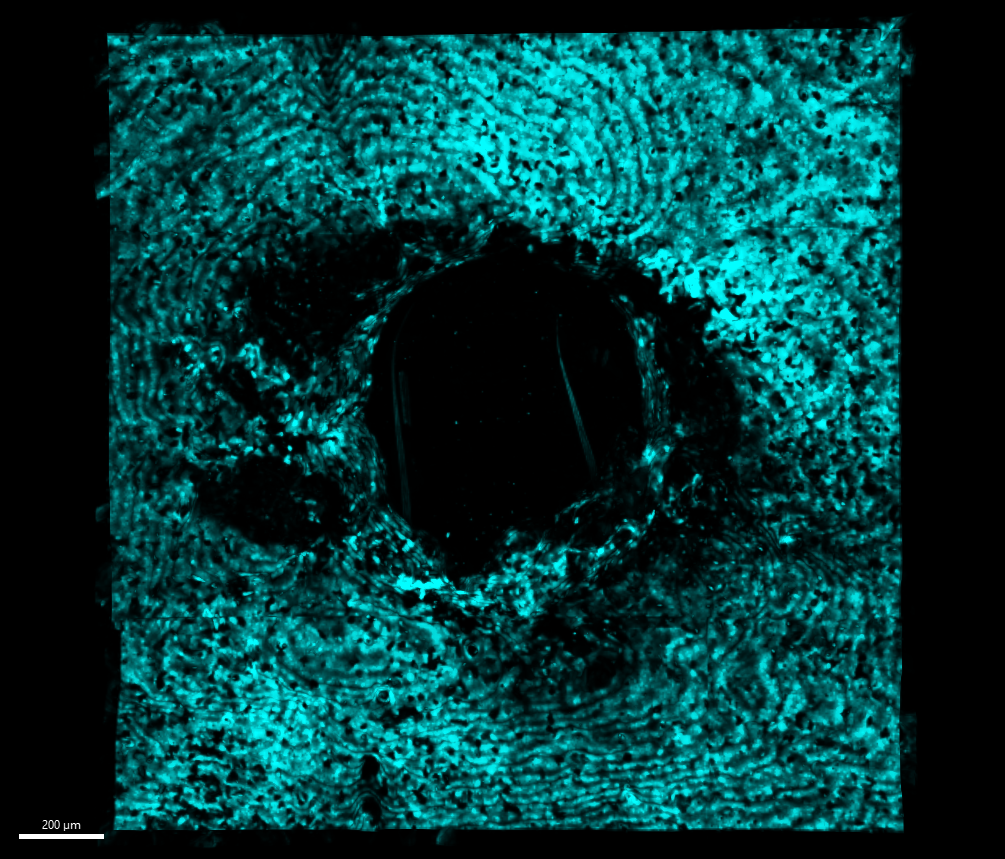

Supplement: Figure 2—source data 1. [file elife-83146-fig2-data1.zip › Figure 2/RE013-day 14 side 2 flip Composite GFP.tif]

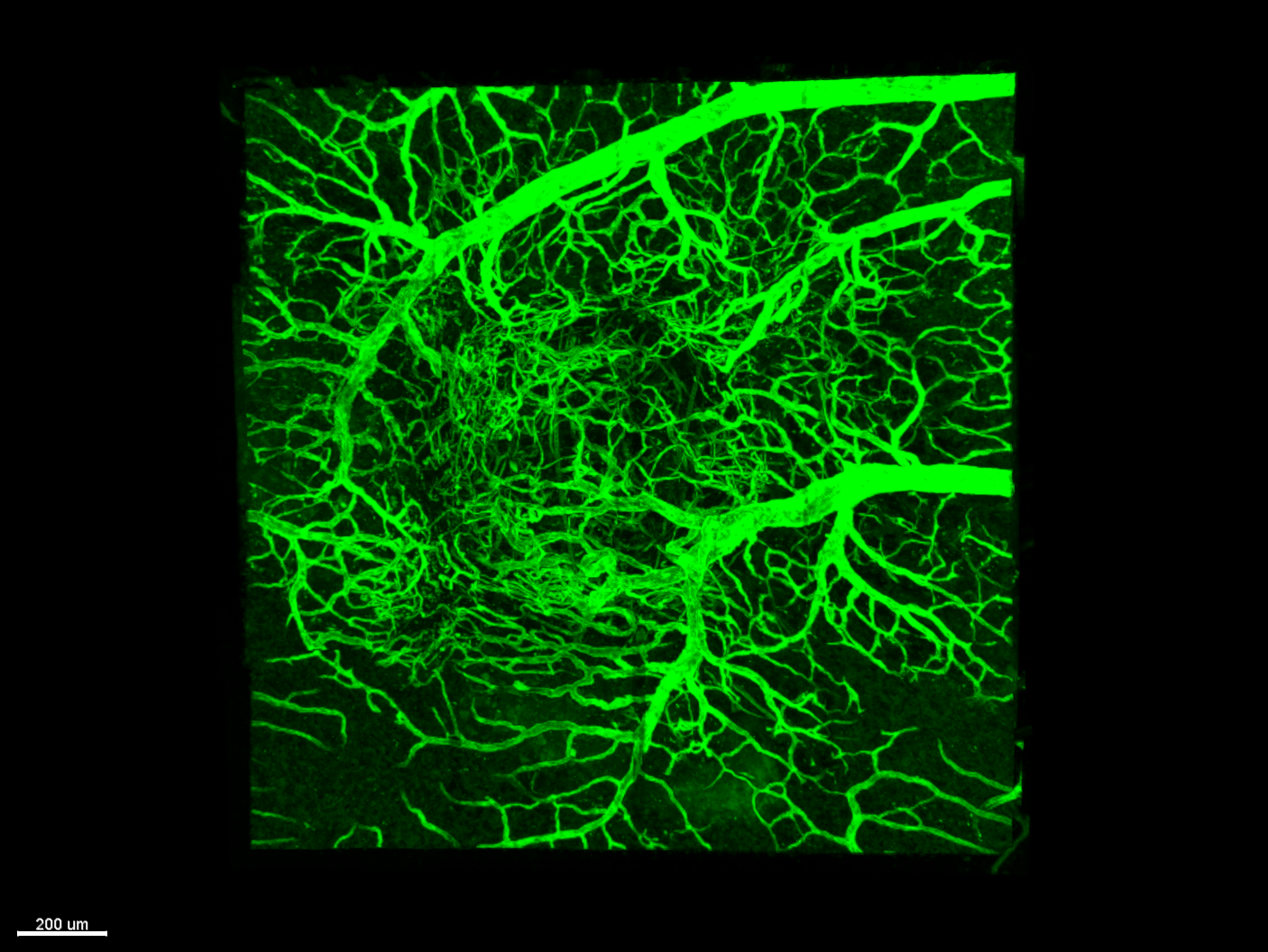

Supplement: Figure 2—source data 1. [file elife-83146-fig2-data1.zip › Figure 2/RE016 day 10 flip side 1 endo.tif]

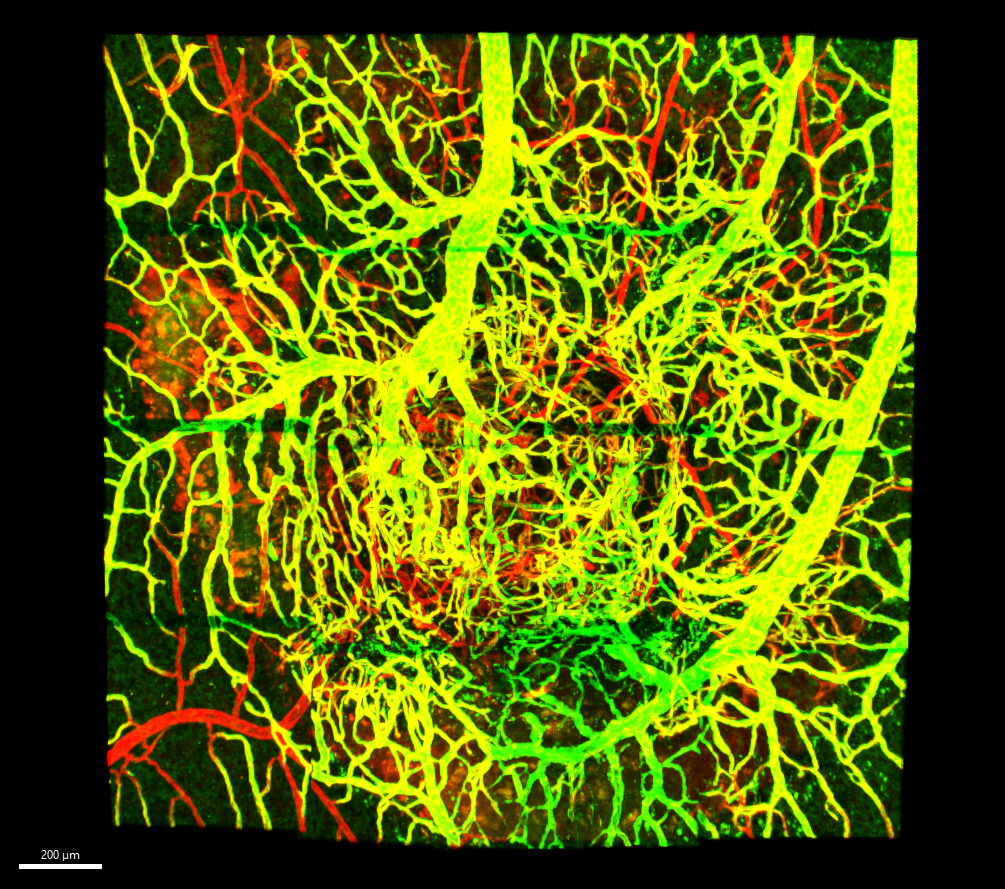

Supplement: Figure 2—source data 1. [file elife-83146-fig2-data1.zip › Figure 2/RE016 day 10 flip side 1 for quan cd31 endo.tif]

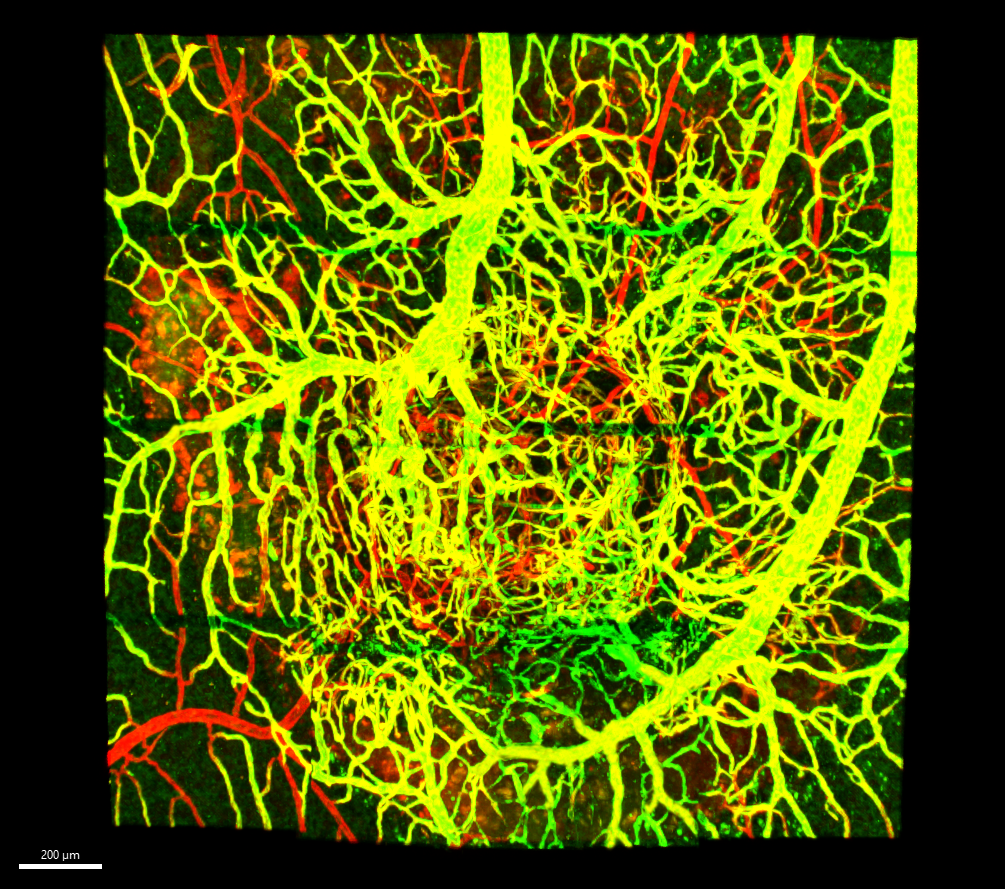

Supplement: Figure 2—source data 1. [file elife-83146-fig2-data1.zip › Figure 2/RE016 day 10 flip side 1 for quan cd31 endo-cleaned.tif]

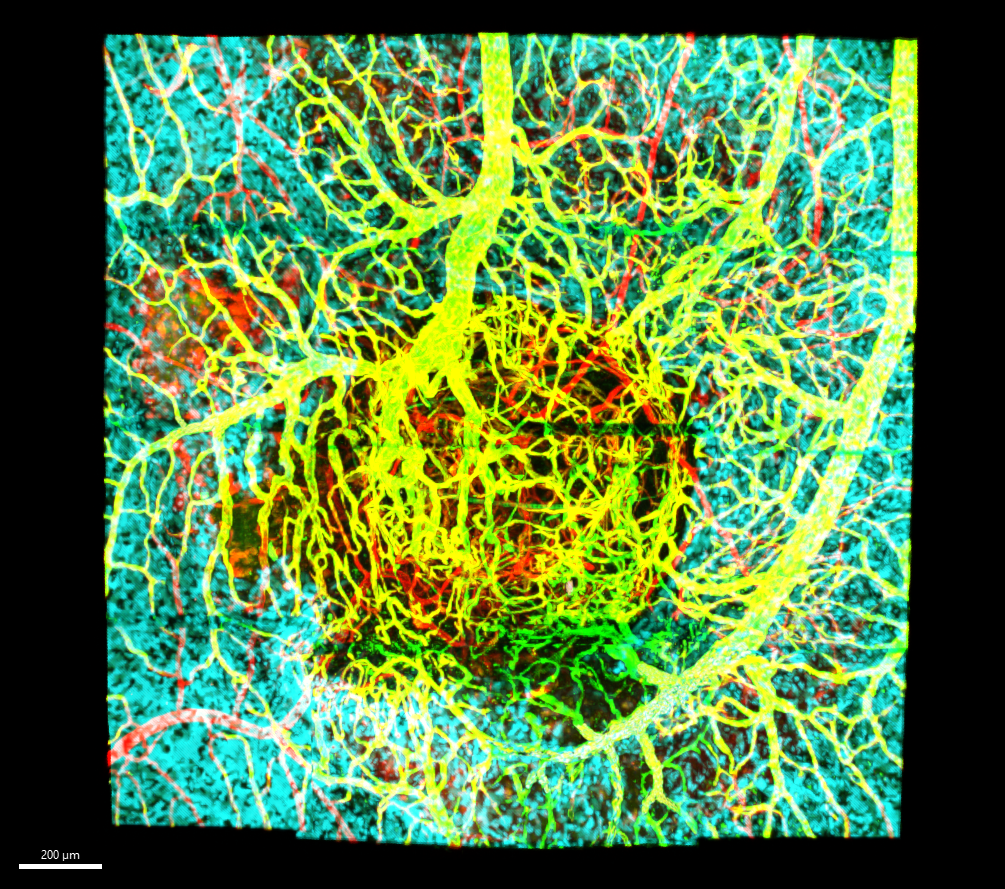

Supplement: Figure 2—source data 1. [file elife-83146-fig2-data1.zip › Figure 2/RE016 day 10 flip side 1 for quan gfp cd31 endo enhanced.tif]

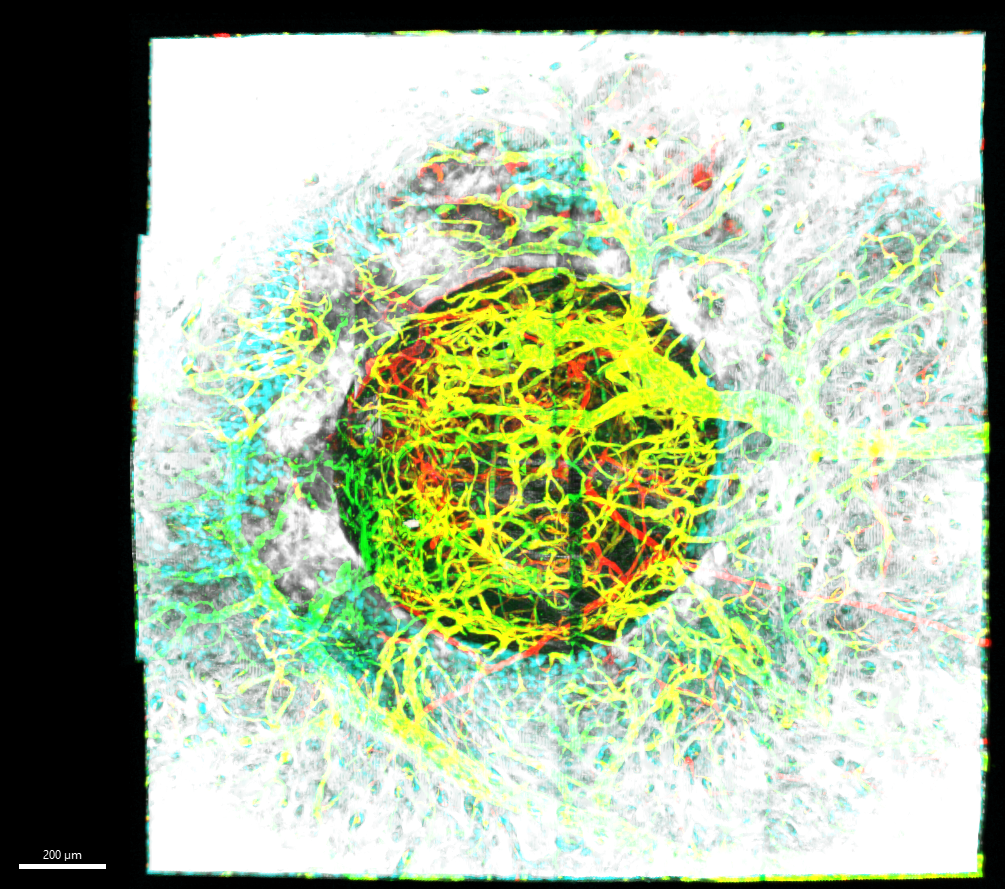

Supplement: Figure 2—source data 1. [file elife-83146-fig2-data1.zip › Figure 2/RE016 day 10 flip side 1 for quan gfp cd31 endo shg.tif]

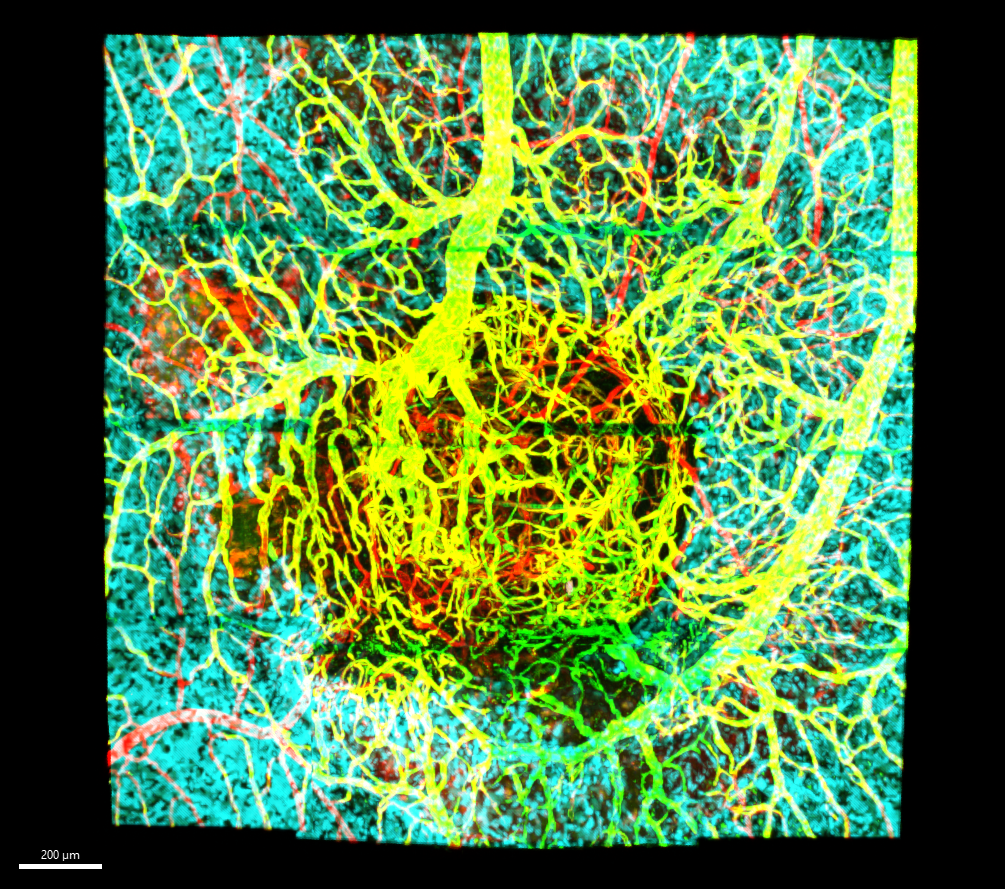

Supplement: Figure 2—source data 1. [file elife-83146-fig2-data1.zip › Figure 2/RE016 day 10 flip side 1 for quan gfp cd31 endo.png]

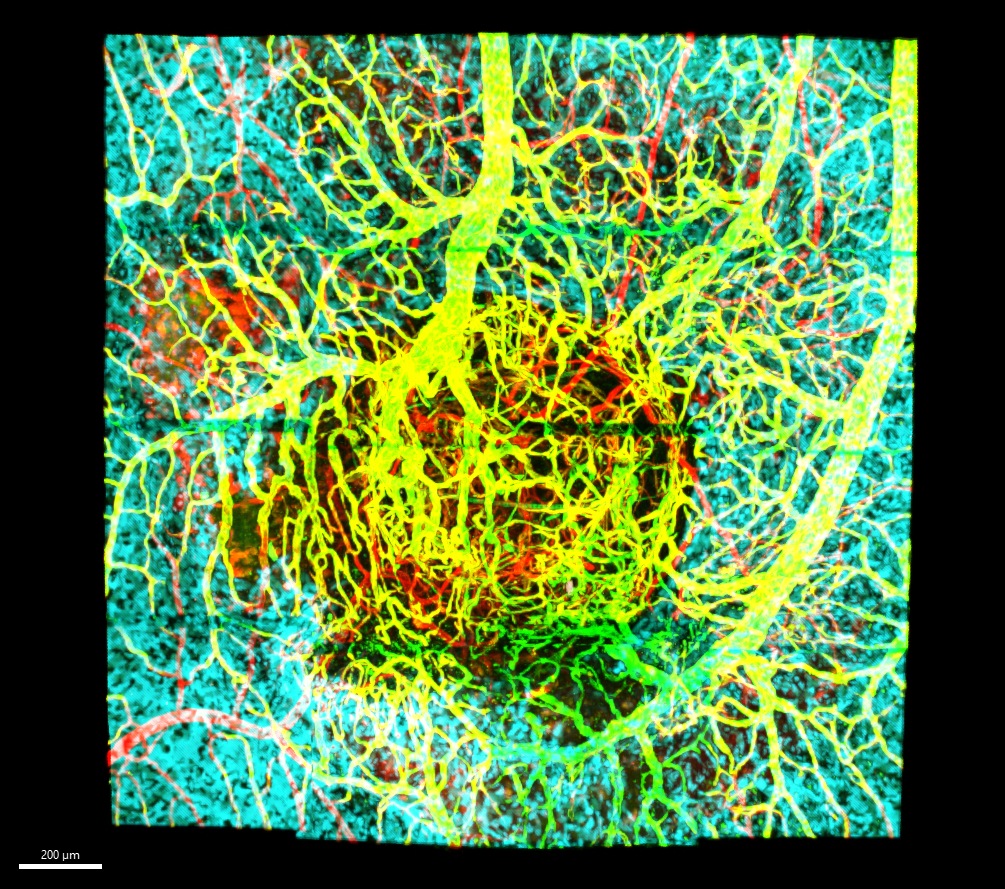

Supplement: Figure 2—source data 1. [file elife-83146-fig2-data1.zip › Figure 2/RE016 day 10 flip side 1 for quan gfp cd31 endo.tif]

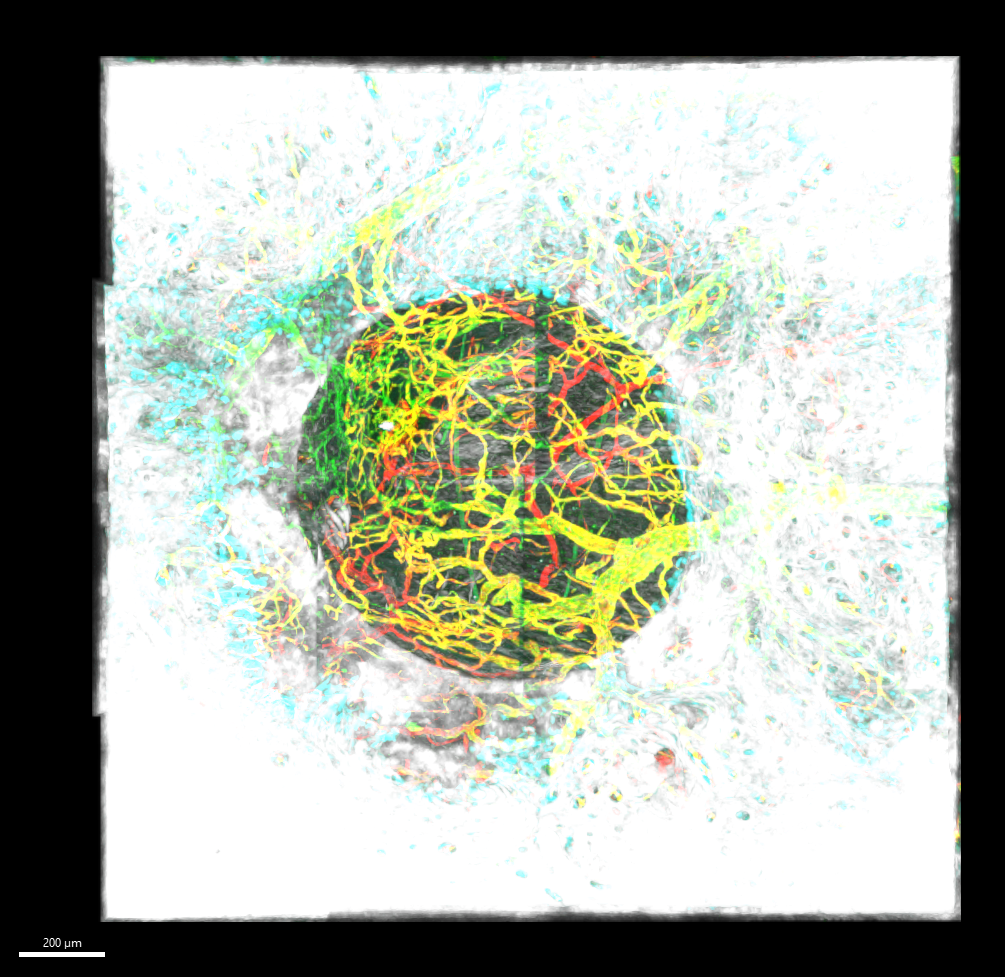

Supplement: Figure 2—source data 1. [file elife-83146-fig2-data1.zip › Figure 2/RE016 day 10 flip side 1 for quan gfp endo cd31 shg.tif]

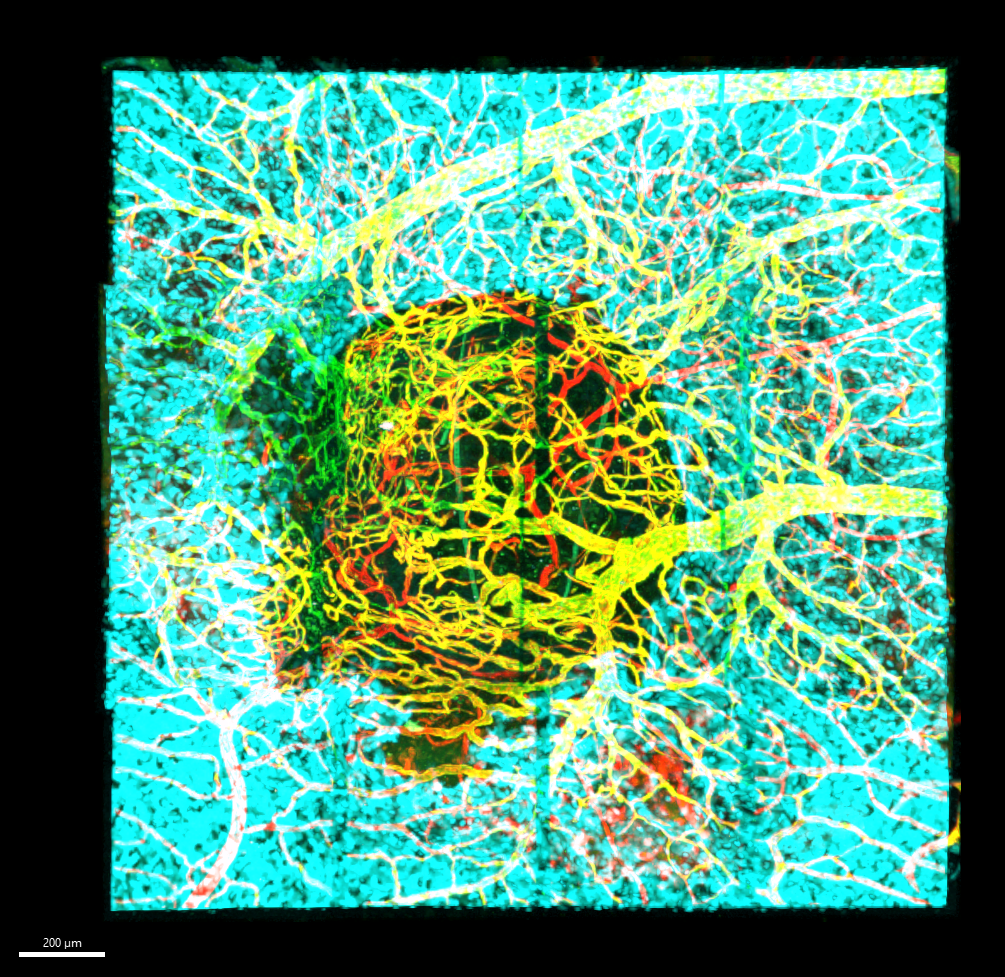

Supplement: Figure 2—source data 1. [file elife-83146-fig2-data1.zip › Figure 2/RE016 day 10 flip side 1 for quan gfp endo cd31.tif]

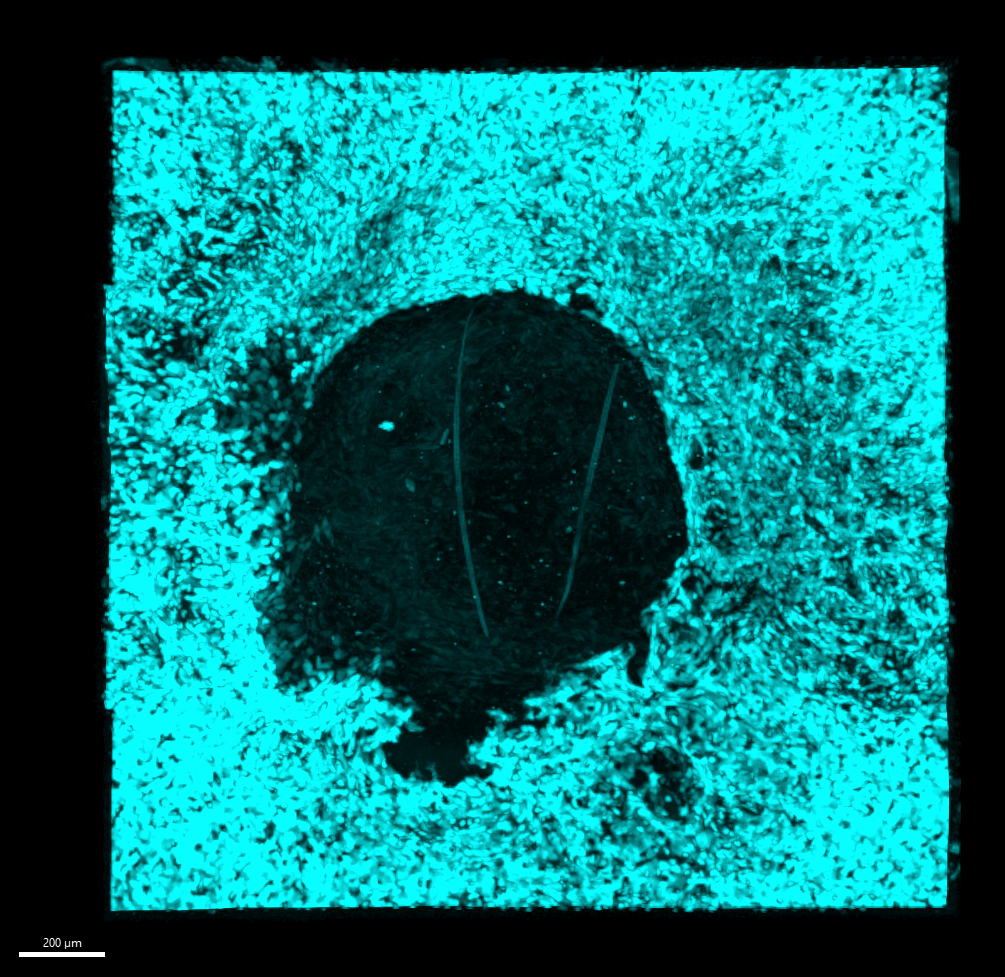

Supplement: Figure 2—source data 1. [file elife-83146-fig2-data1.zip › Figure 2/RE016 day 10 flip side 1 for quan gfp.tif]

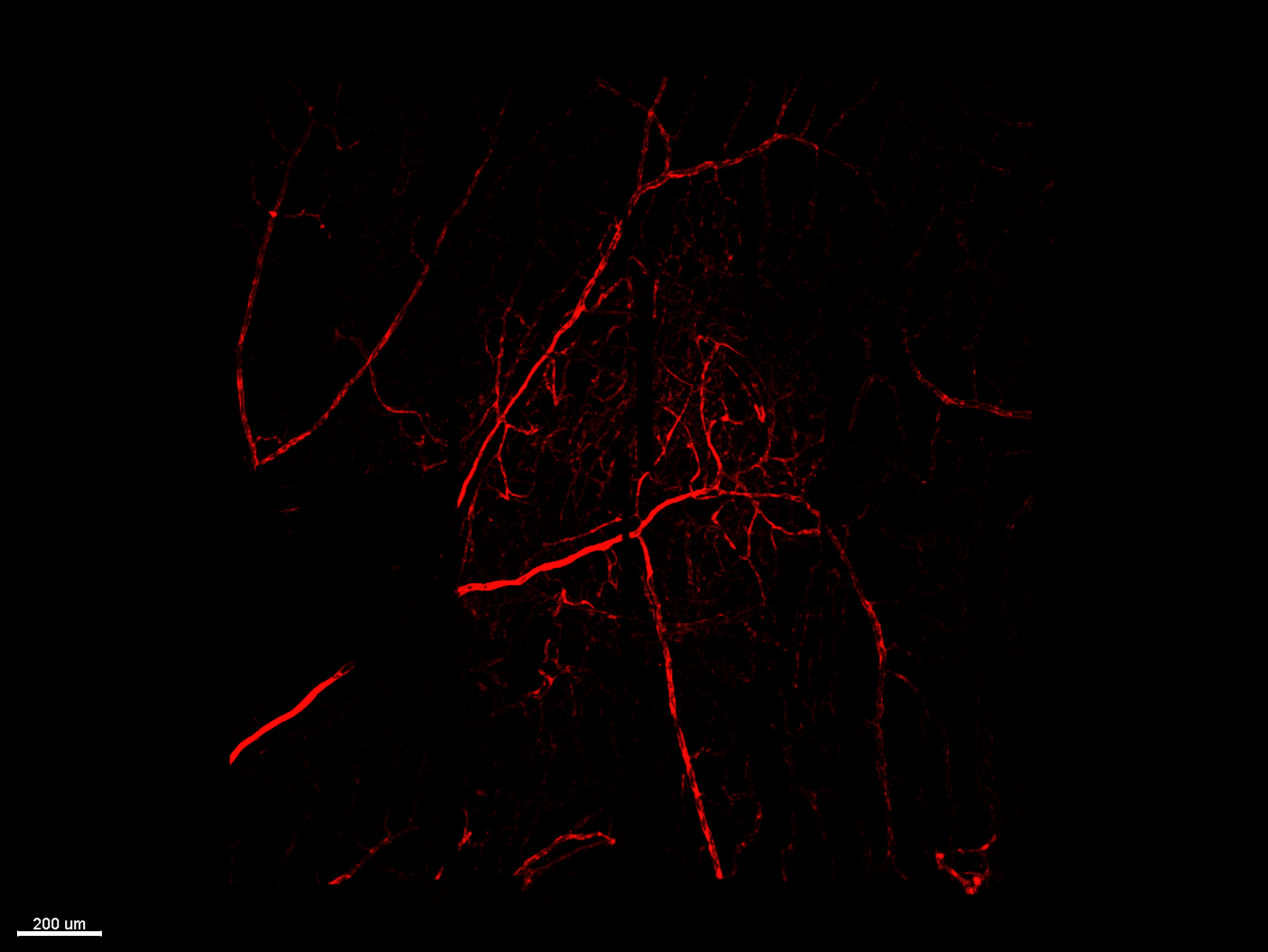

Supplement: Figure 2—source data 1. [file elife-83146-fig2-data1.zip › Figure 2/RE016 day 10 flip side 2 cd31 only.tif]

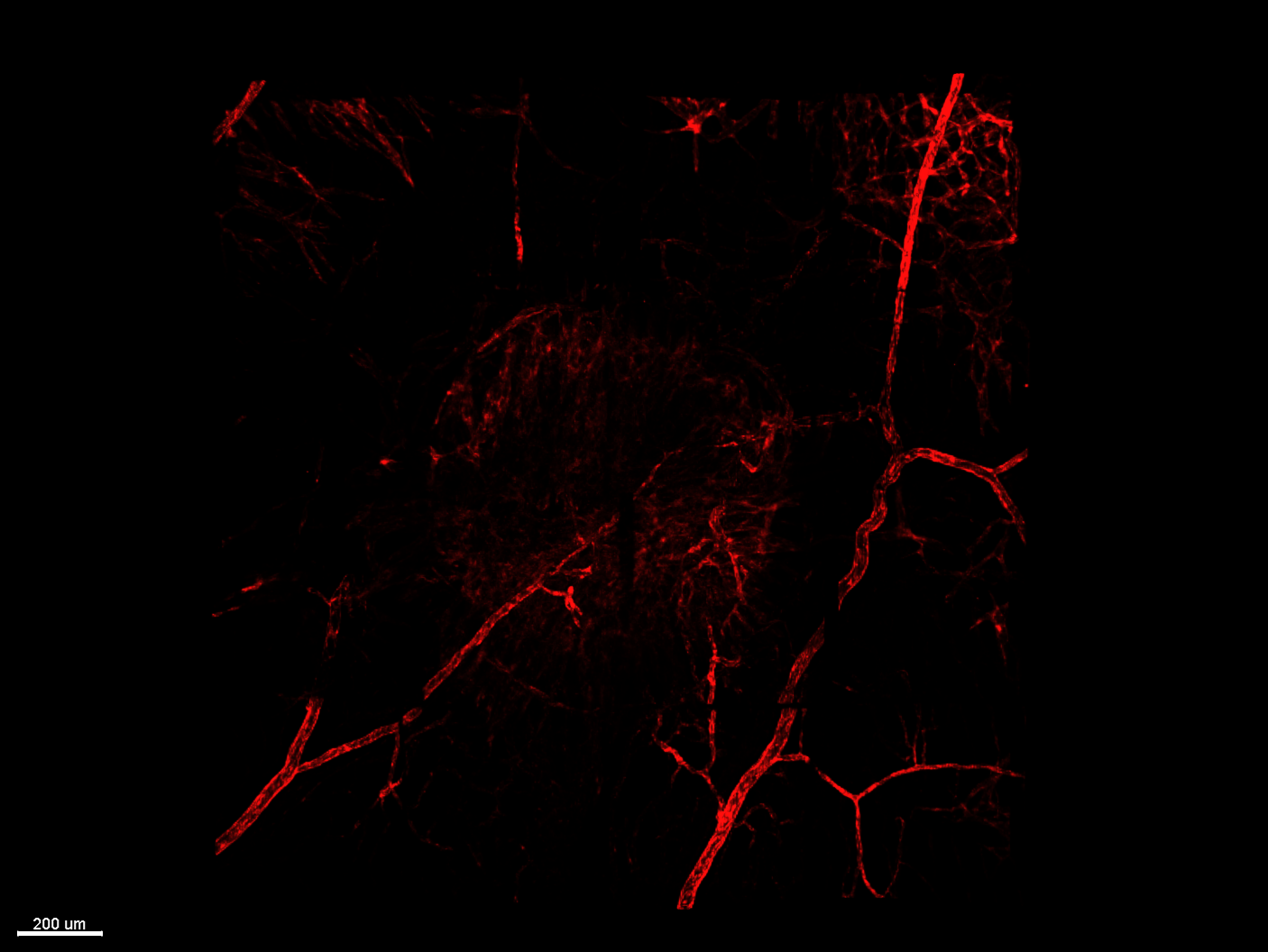

Supplement: Figure 2—source data 1. [file elife-83146-fig2-data1.zip › Figure 2/RE085 day 5 flip side 2 cd31 only.tif]

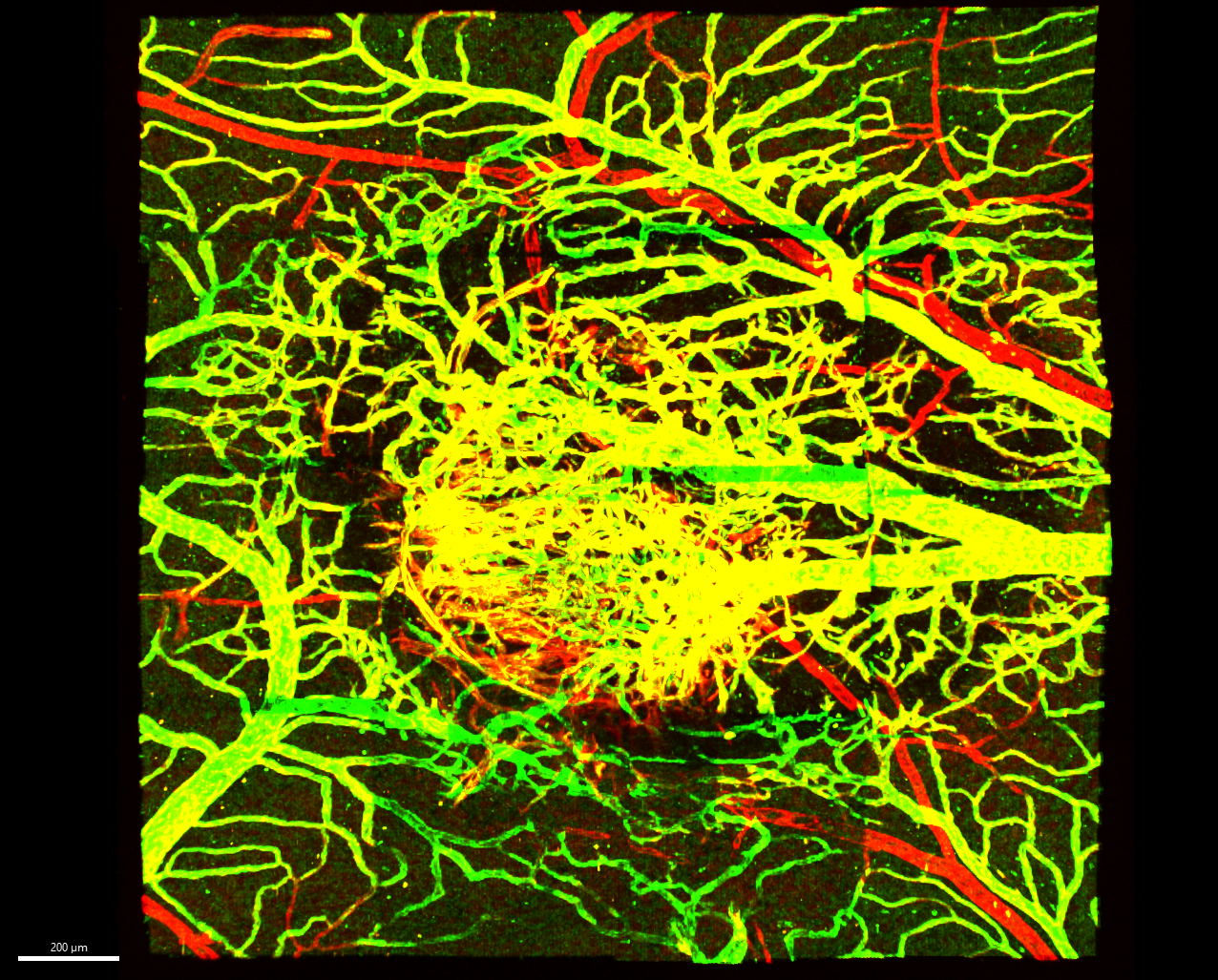

Supplement: Figure 2—source data 1. [file elife-83146-fig2-data1.zip › Figure 2/RE085 day 5 flip side 2 for quan endo cd31 good.tif]

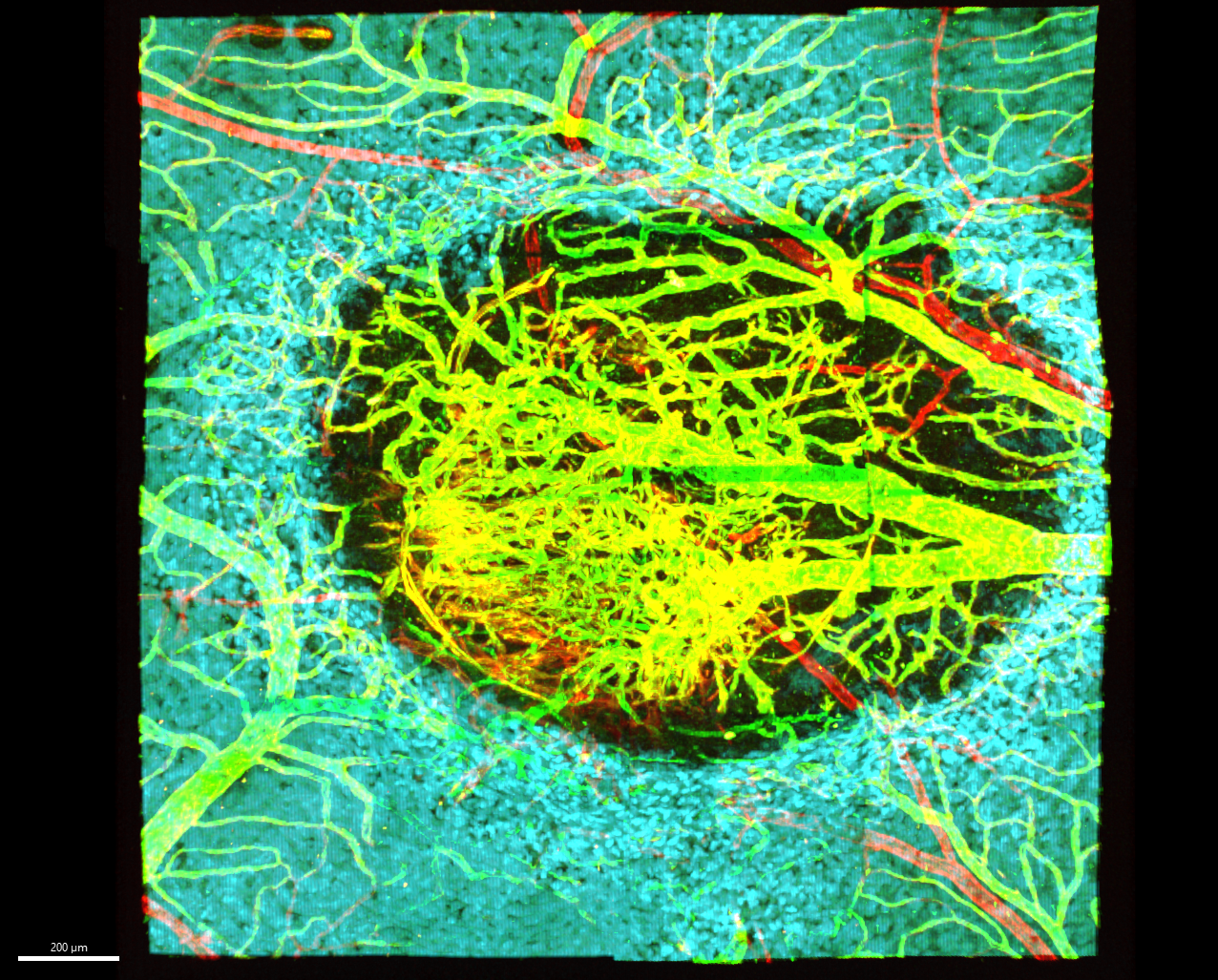

Supplement: Figure 2—source data 1. [file elife-83146-fig2-data1.zip › Figure 2/RE085 day 5 flip side 2 for quan endo cd31 gfp good.tif]

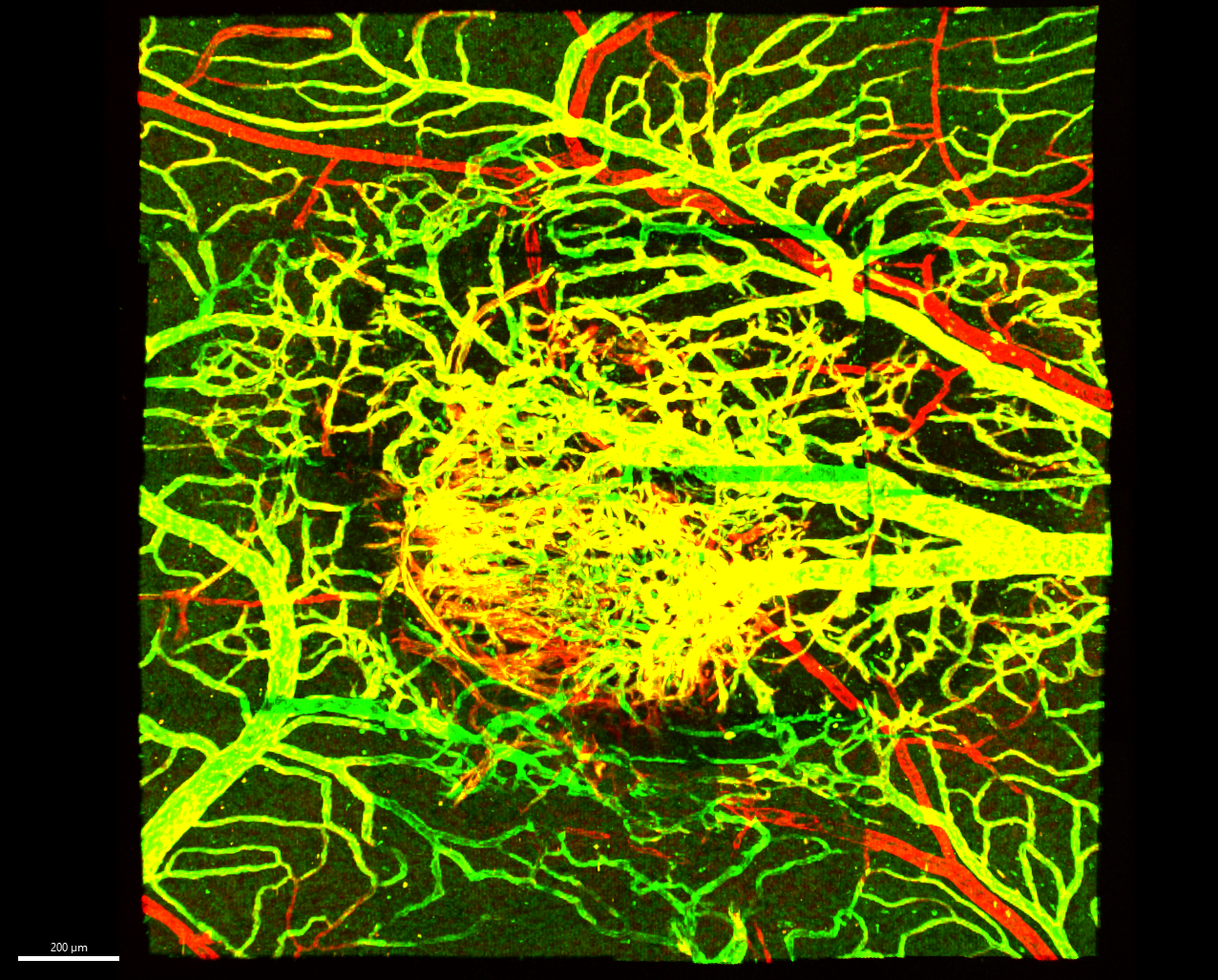

Supplement: Figure 2—source data 1. [file elife-83146-fig2-data1.zip › Figure 2/RE085 day 5 flip side 2 for quan endo cd31 gfp good-3.tif]

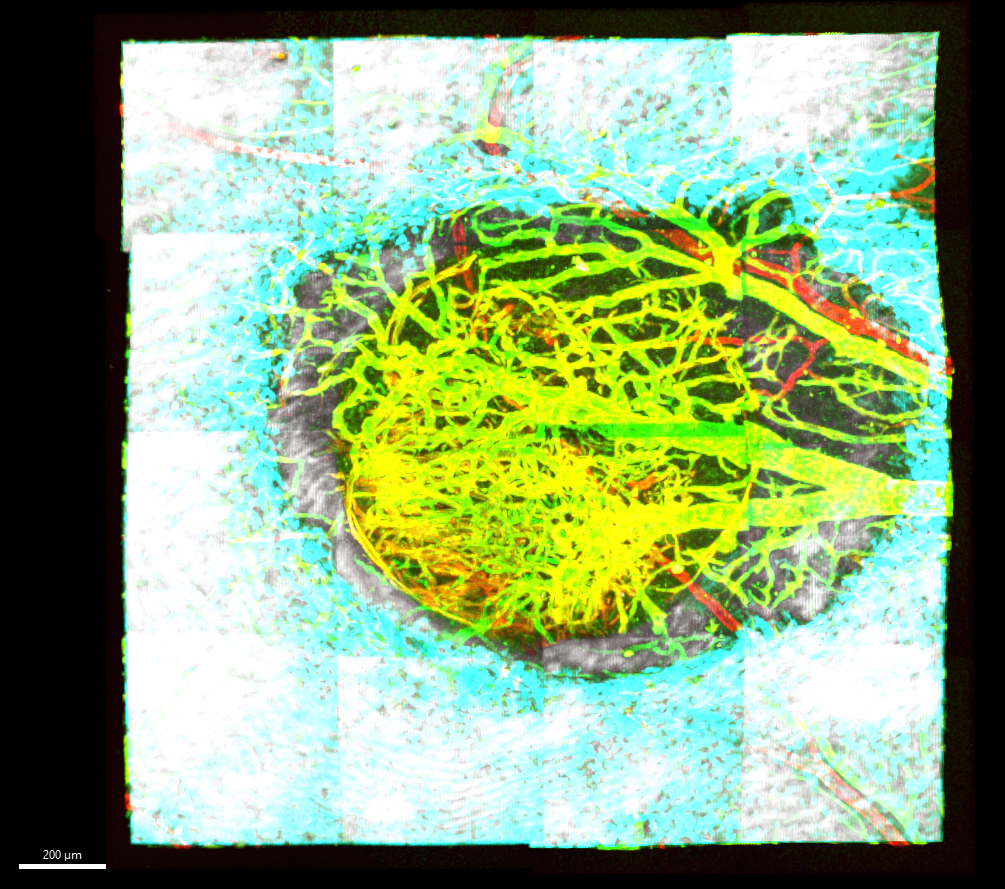

Supplement: Figure 2—source data 1. [file elife-83146-fig2-data1.zip › Figure 2/RE085 day 5 flip side 2 for quan endo cd31 gfp shg.tif]

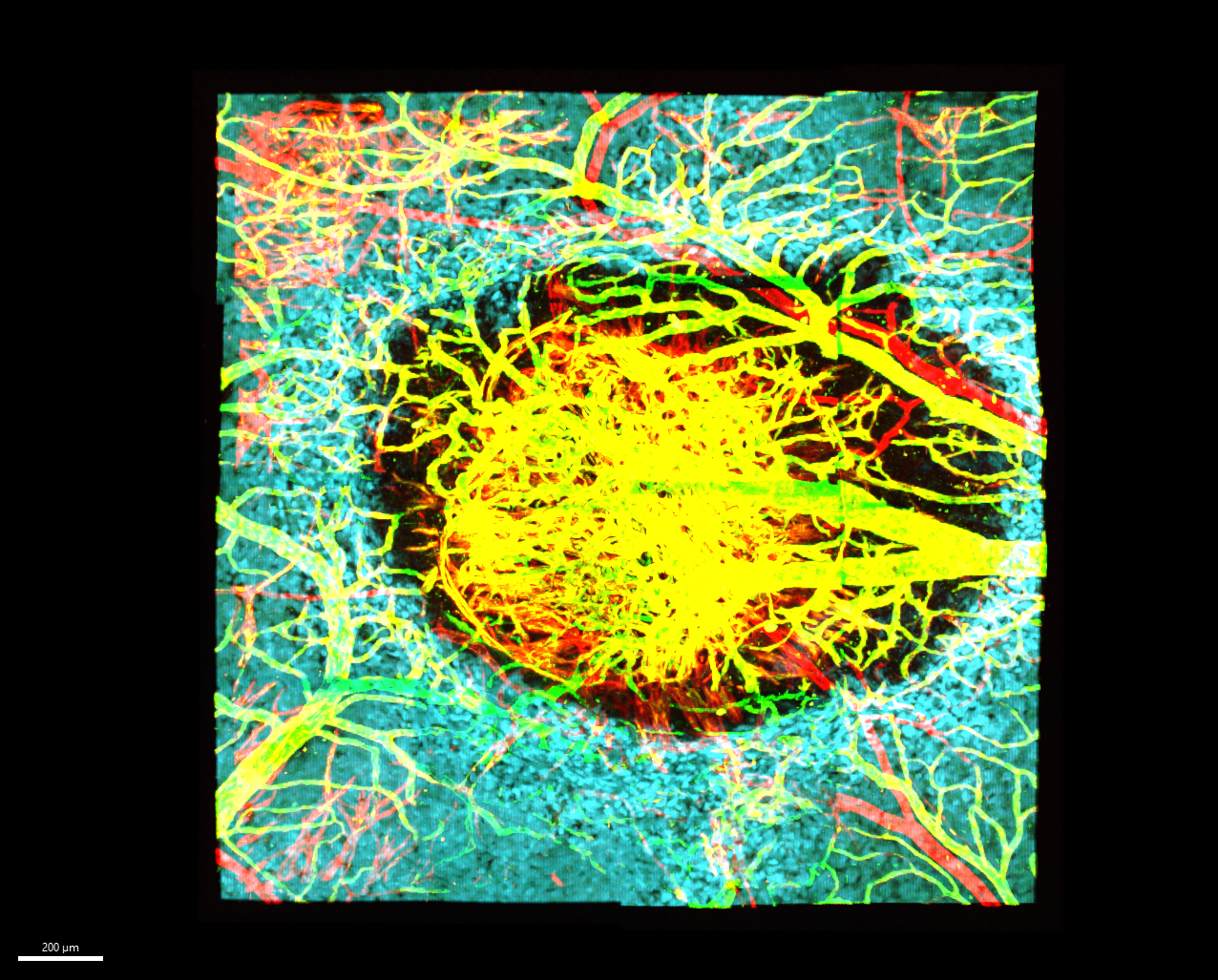

Supplement: Figure 2—source data 1. [file elife-83146-fig2-data1.zip › Figure 2/RE085 day 5 flip side 2 for quan endo cd31 gfp.tif]

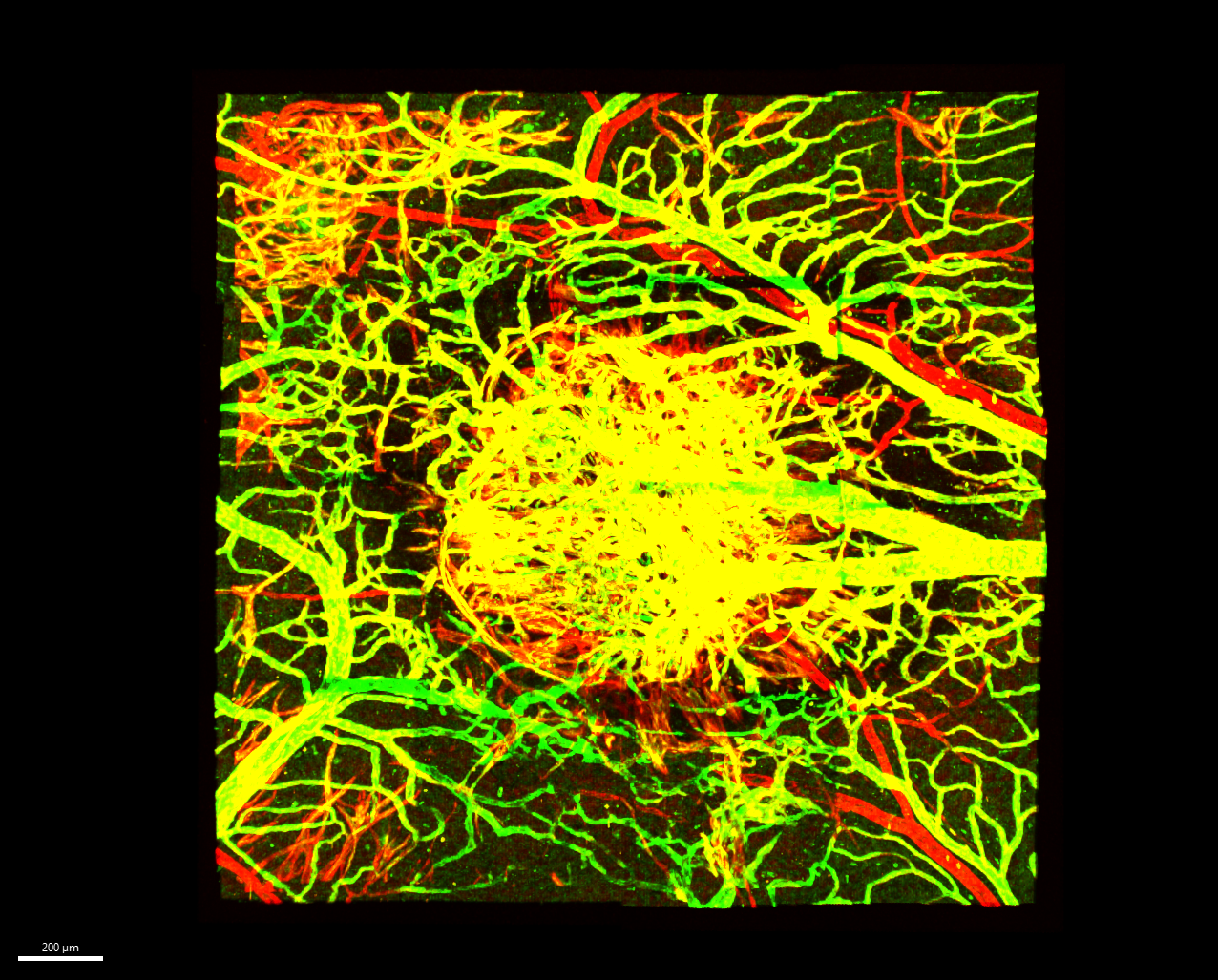

Supplement: Figure 2—source data 1. [file elife-83146-fig2-data1.zip › Figure 2/RE085 day 5 flip side 2 for quan endo cd31.tif]

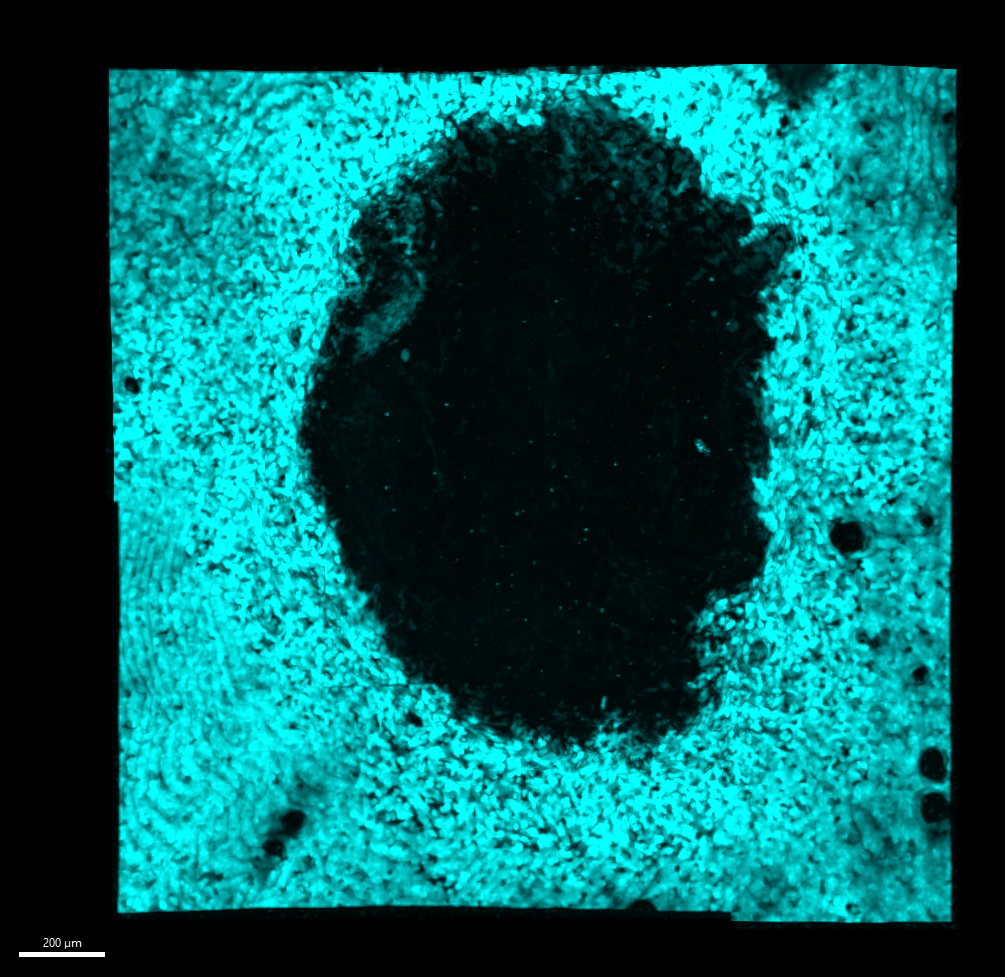

Supplement: Figure 2—source data 1. [file elife-83146-fig2-data1.zip › Figure 2/RE085 day 5 flip side 2 for quan gfp.tif]

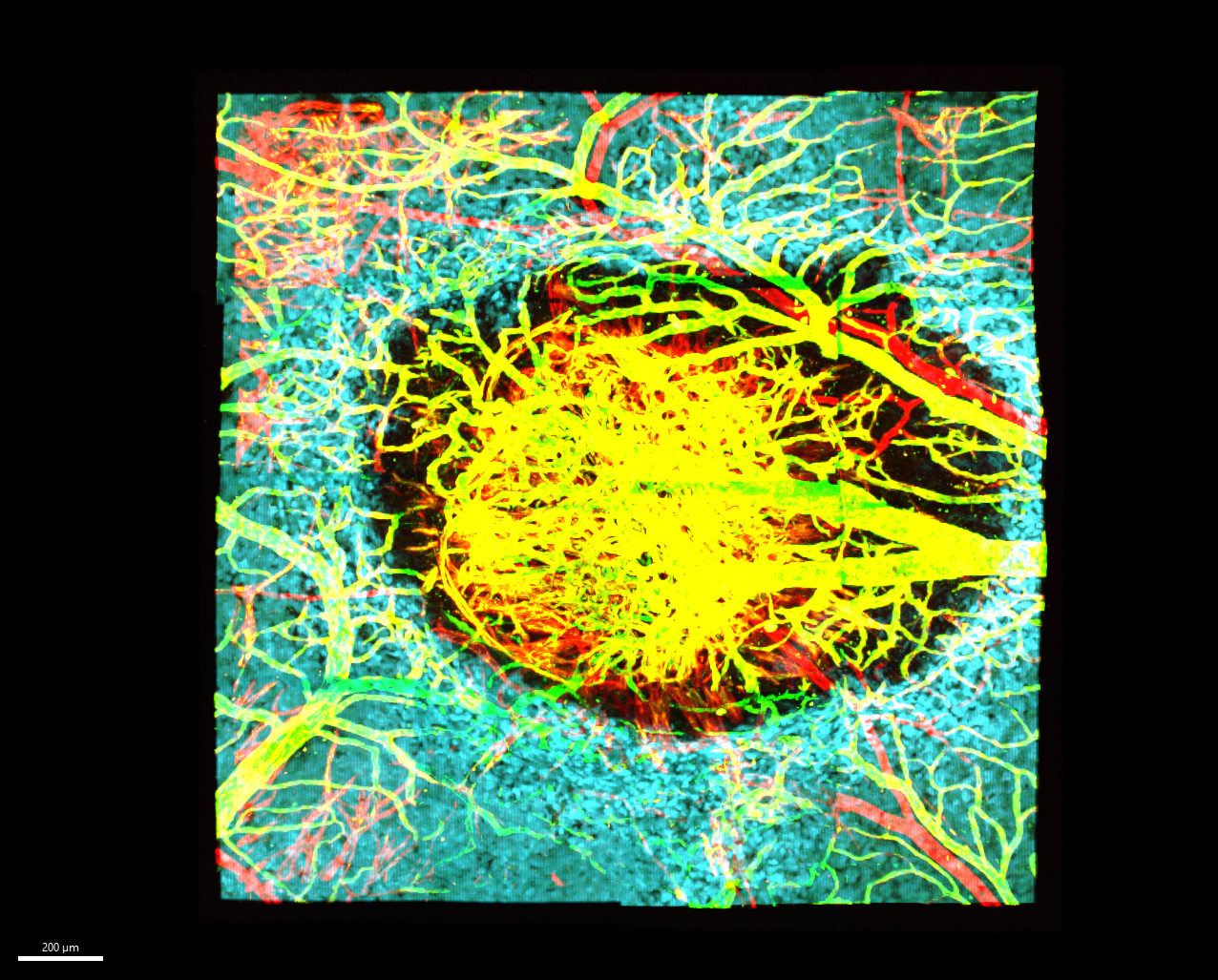

Supplement: Figure 2—source data 1. [file elife-83146-fig2-data1.zip › Figure 2/RE085 day 5 flip side 2 for quan_gfp endo cd31.png]

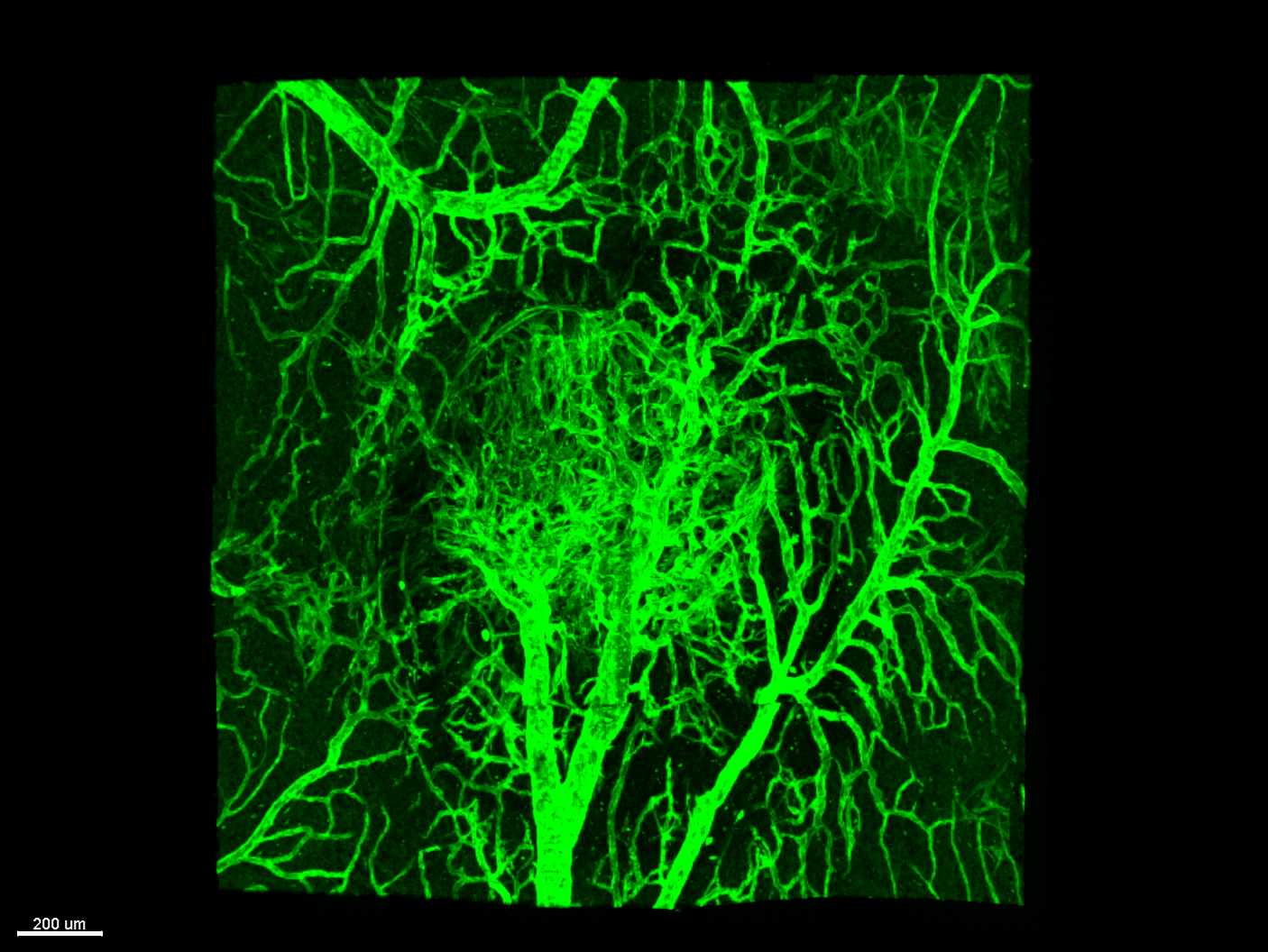

Supplement: Figure 2—source data 1. [file elife-83146-fig2-data1.zip › Figure 2/RE085 day 5 flip side 2endo only.tif]

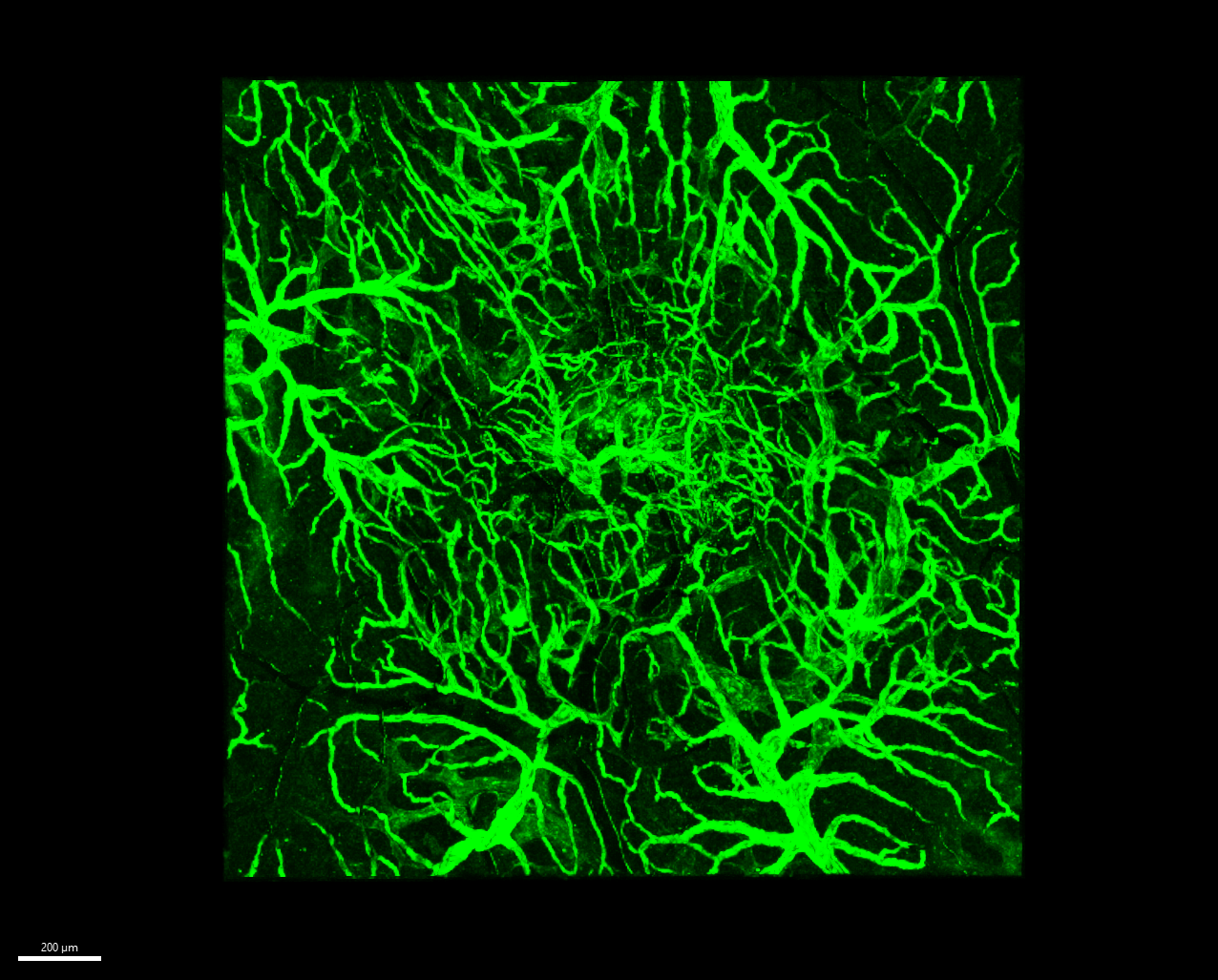

Supplement: Figure 3—source data 1. [file elife-83146-fig3-data1.zip › Figure 3/304-BMX1 side1 flip endo only.tif]

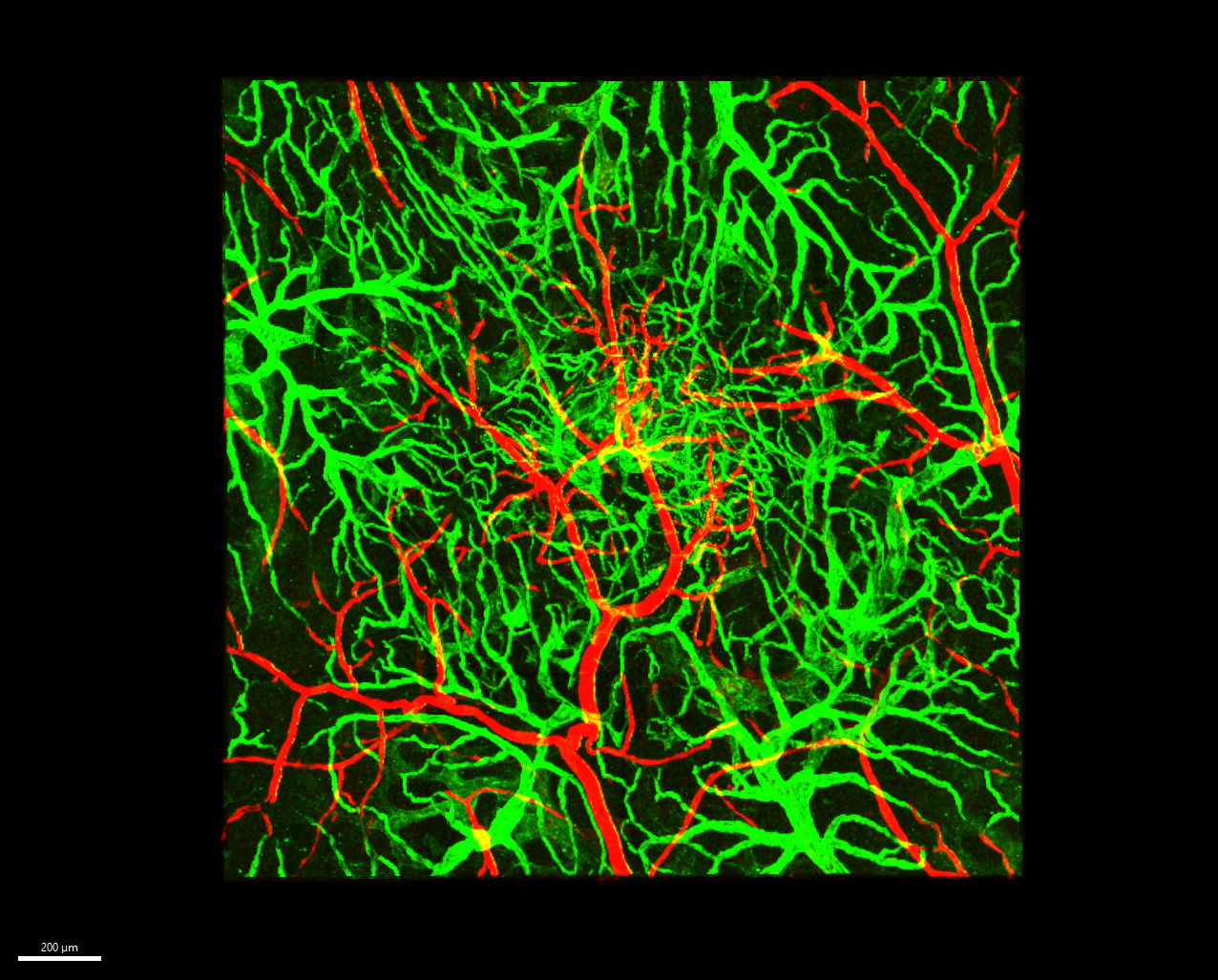

Supplement: Figure 3—source data 1. [file elife-83146-fig3-data1.zip › Figure 3/304-BMX1 side1 flip bmx1 endo.tif]

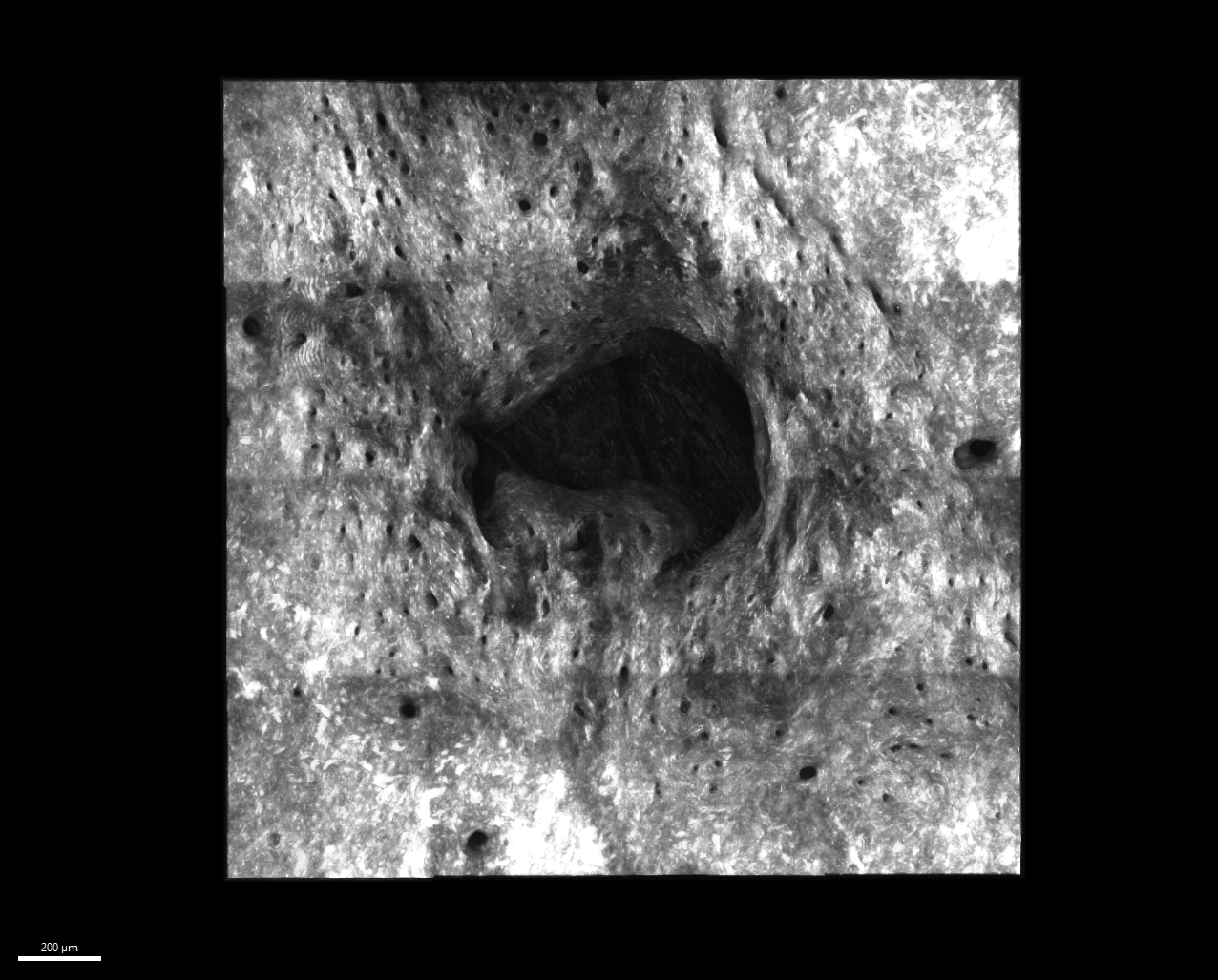

Supplement: Figure 3—source data 1. [file elife-83146-fig3-data1.zip › Figure 3/304-BMX1 side1 flip bmx1 shg.tif]

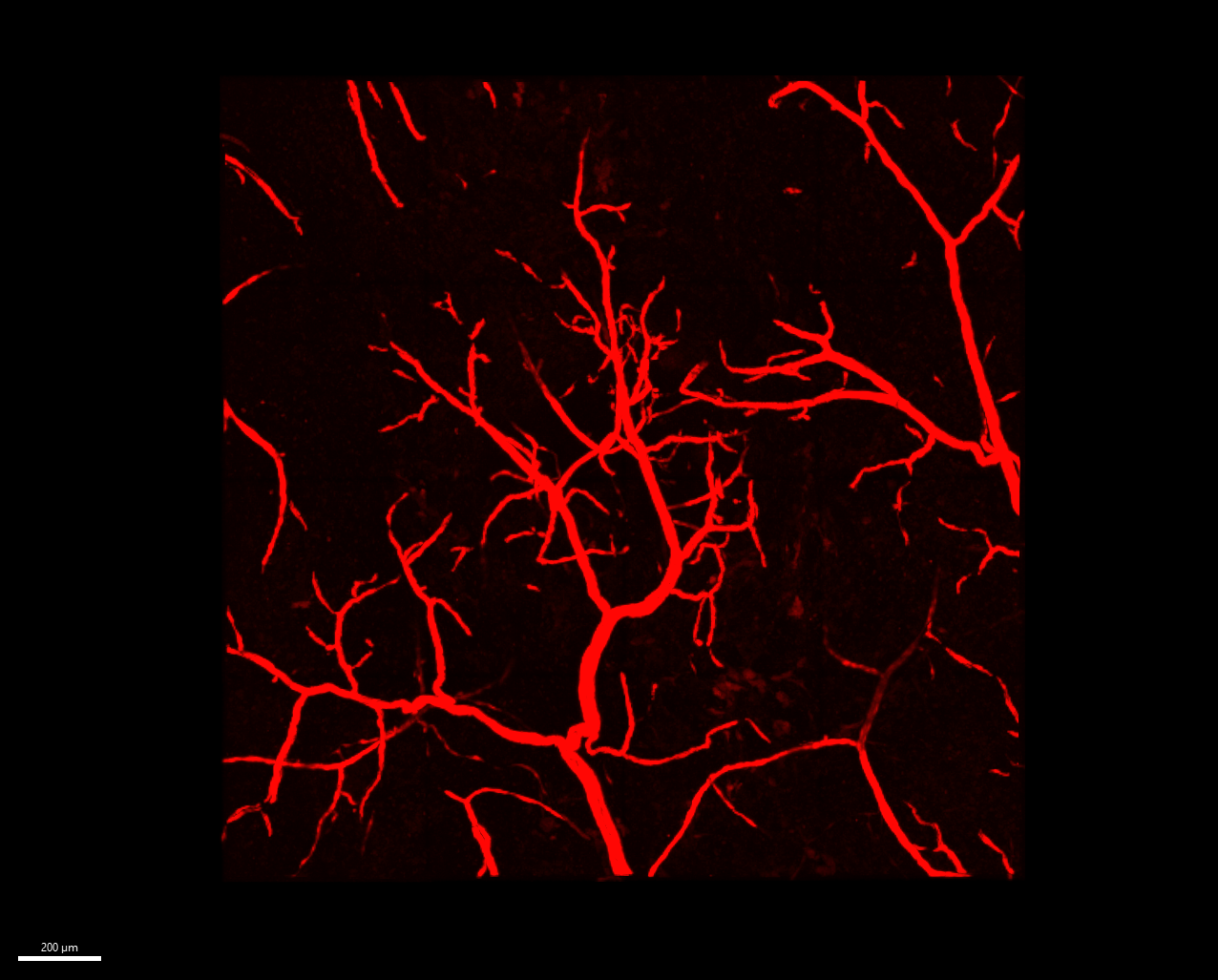

Supplement: Figure 3—source data 1. [file elife-83146-fig3-data1.zip › Figure 3/304-BMX1 side1 flip bmx1.tif]

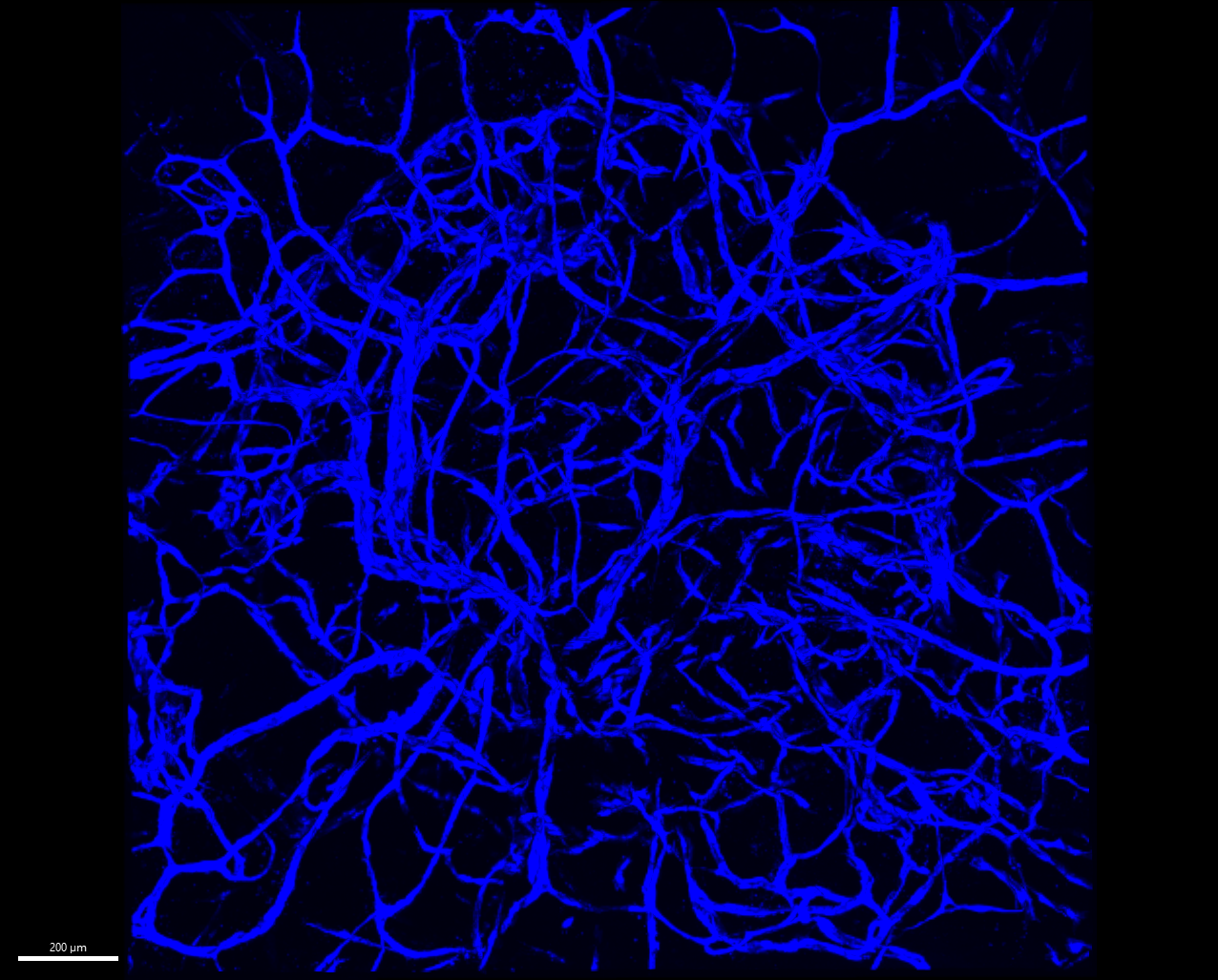

Supplement: Figure 3—source data 1. [file elife-83146-fig3-data1.zip › Figure 3/420+ apln side 2 Apln.tif]

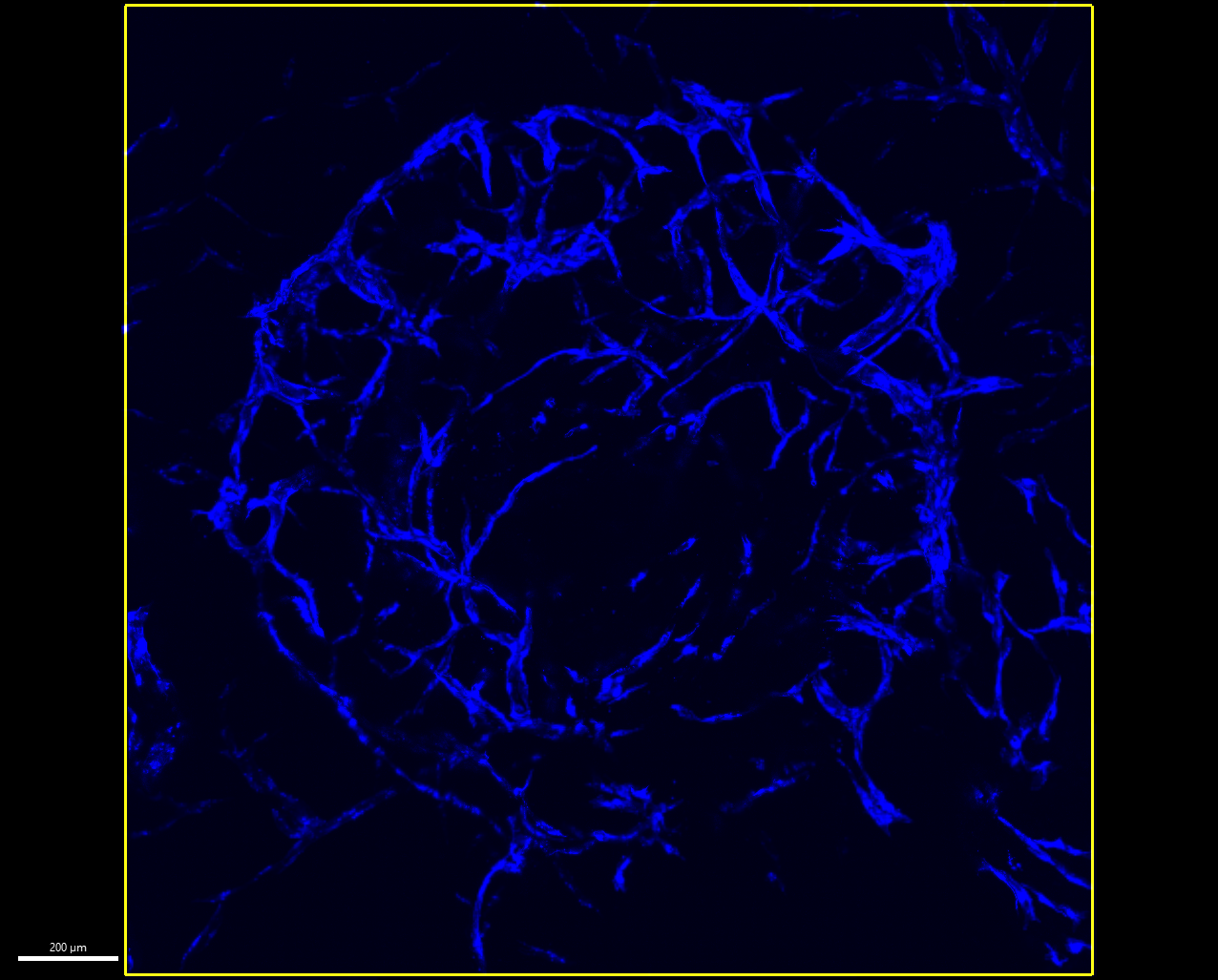

Supplement: Figure 3—source data 1. [file elife-83146-fig3-data1.zip › Figure 3/420+ apln side 2 Apln-2.tif]

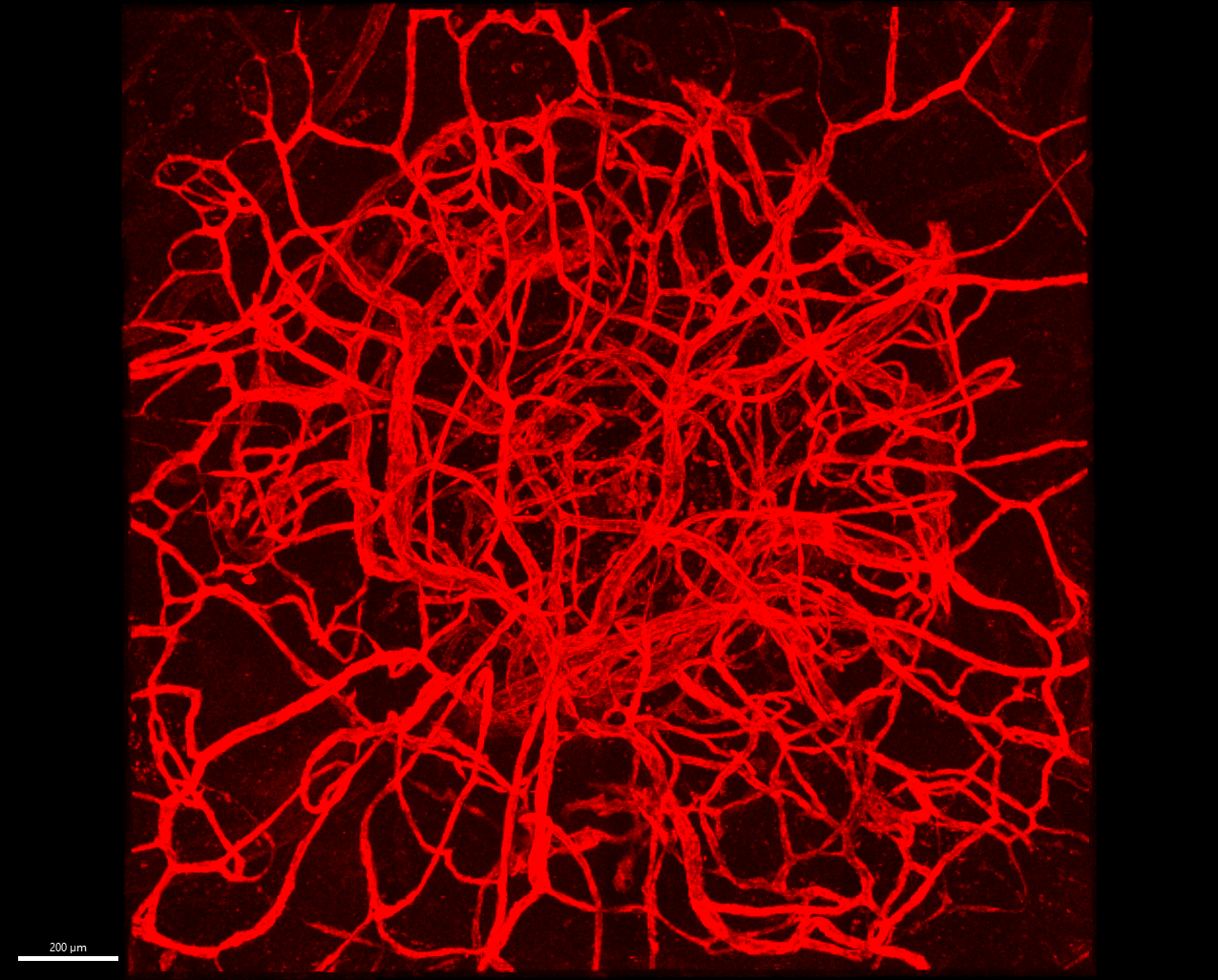

Supplement: Figure 3—source data 1. [file elife-83146-fig3-data1.zip › Figure 3/420+ apln side 2 CD31.tif]

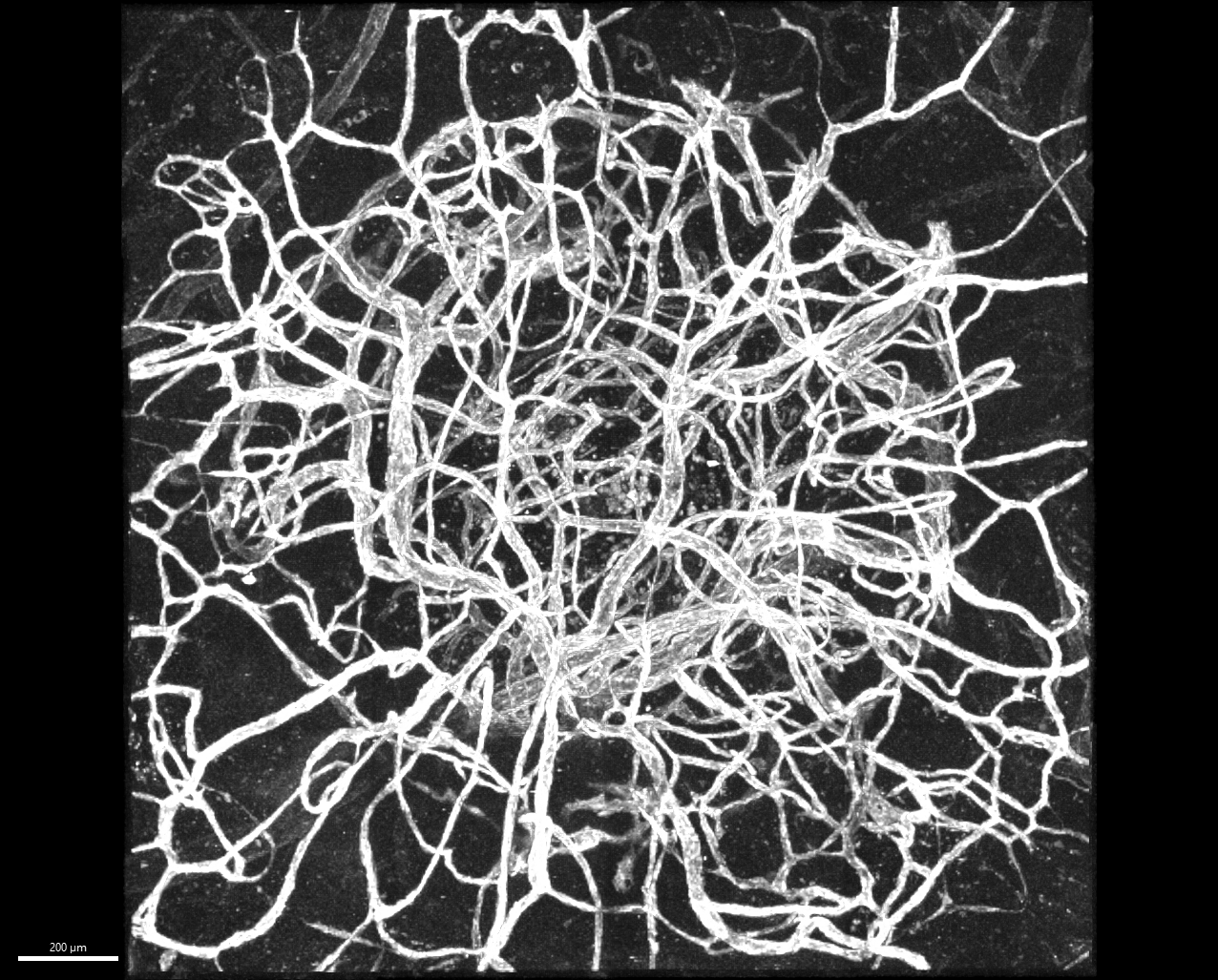

Supplement: Figure 3—source data 1. [file elife-83146-fig3-data1.zip › Figure 3/420+ apln side 2 composite.tif]

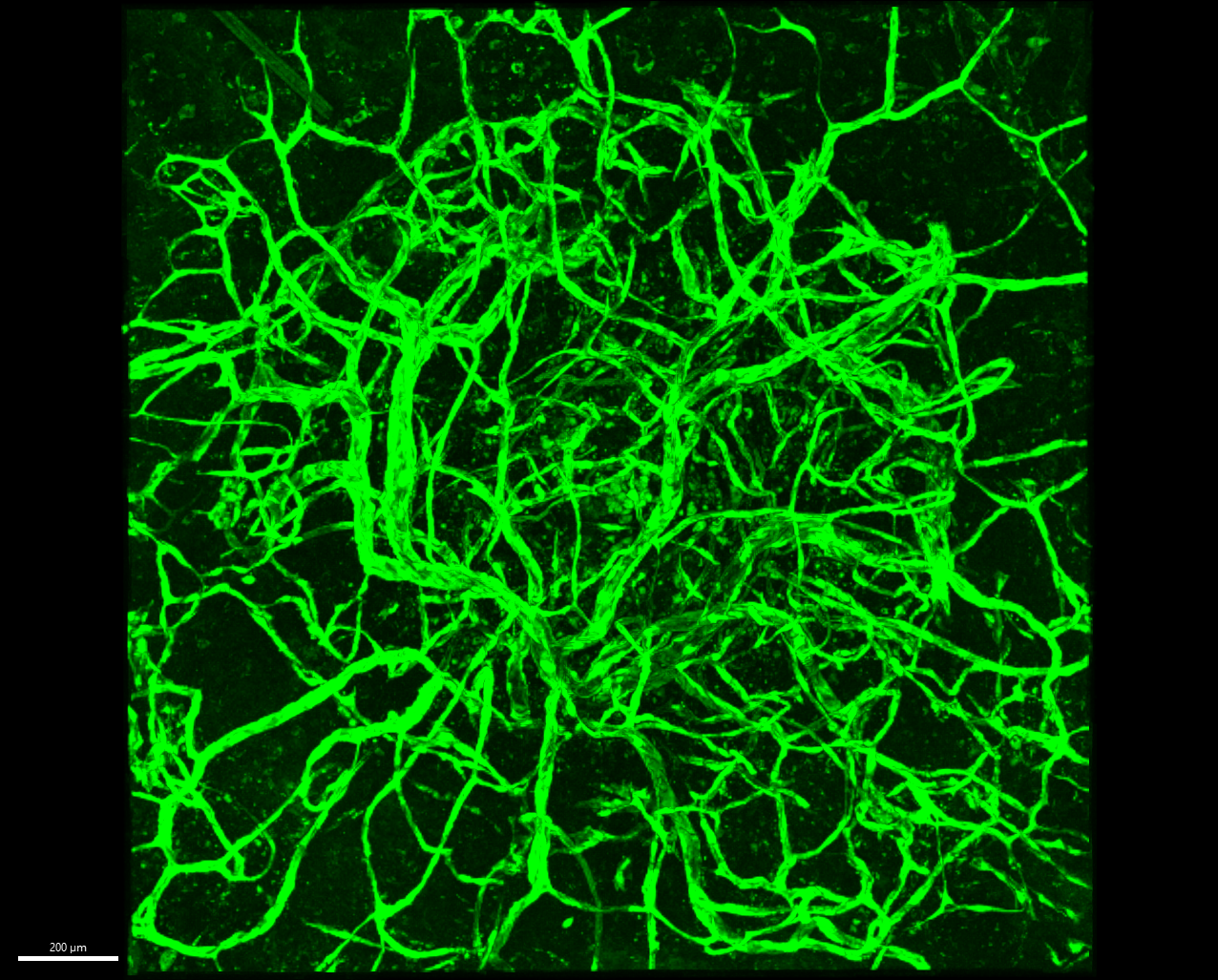

Supplement: Figure 3—source data 1. [file elife-83146-fig3-data1.zip › Figure 3/420+ apln side 2 Endo.tif]

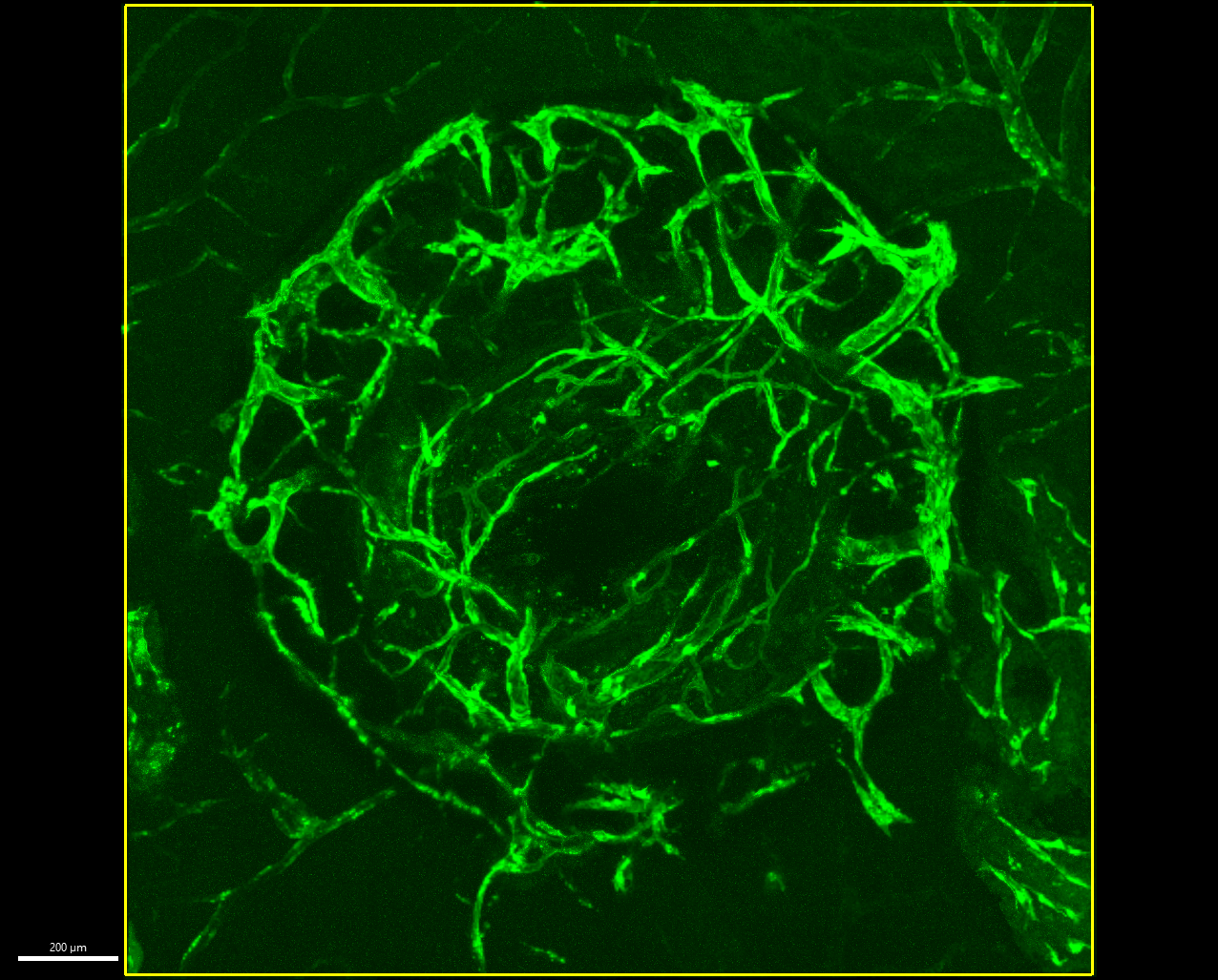

Supplement: Figure 3—source data 1. [file elife-83146-fig3-data1.zip › Figure 3/420+ apln side 2 endo-2.tif]

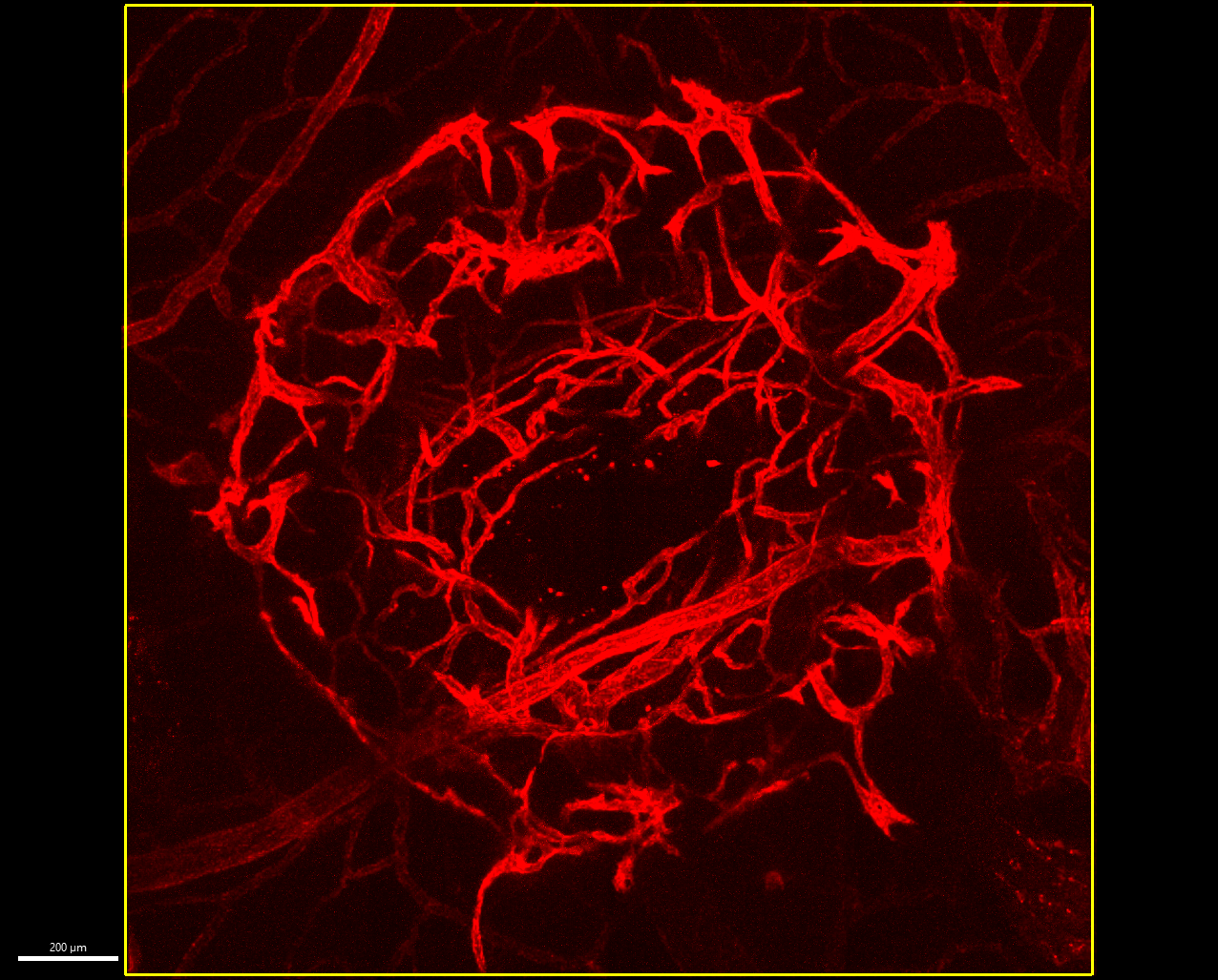

Supplement: Figure 3—source data 1. [file elife-83146-fig3-data1.zip › Figure 3/420+ apln side 2 SHG CD31-2.tif]

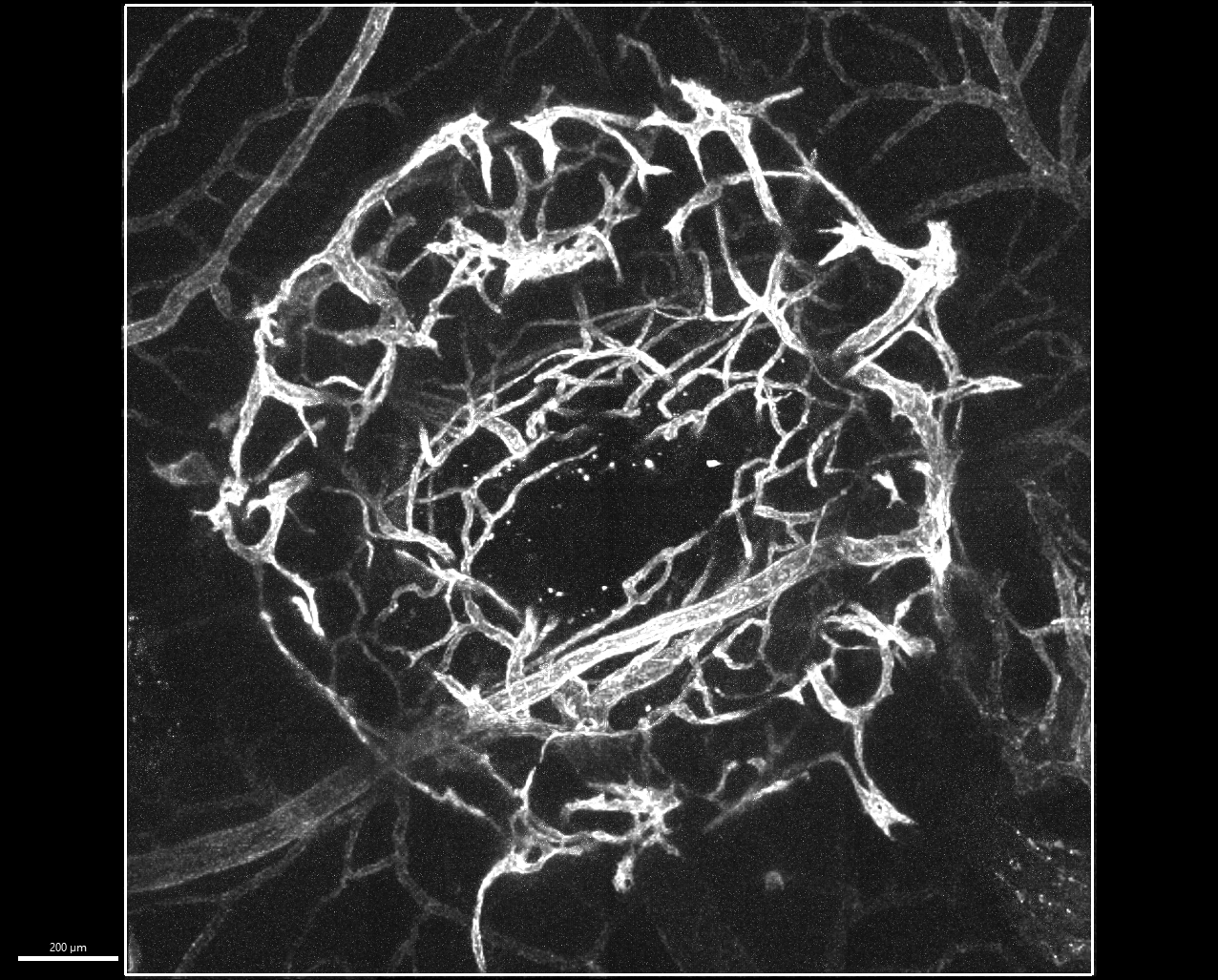

Supplement: Figure 3—source data 1. [file elife-83146-fig3-data1.zip › Figure 3/420+ apln side 2 SHG composite-2.tif]

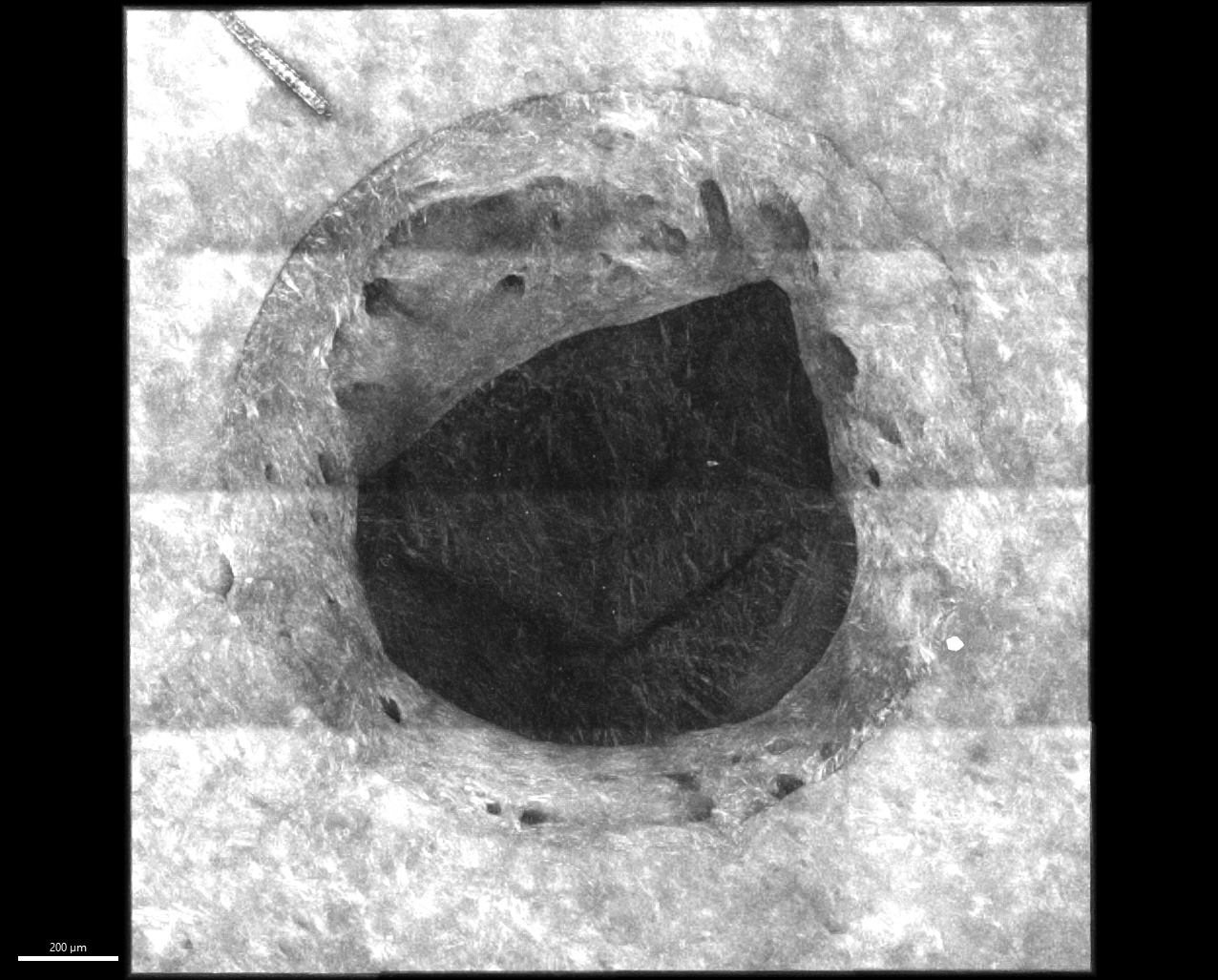

Supplement: Figure 3—source data 1. [file elife-83146-fig3-data1.zip › Figure 3/420+ apln side 2 SHG.tif]

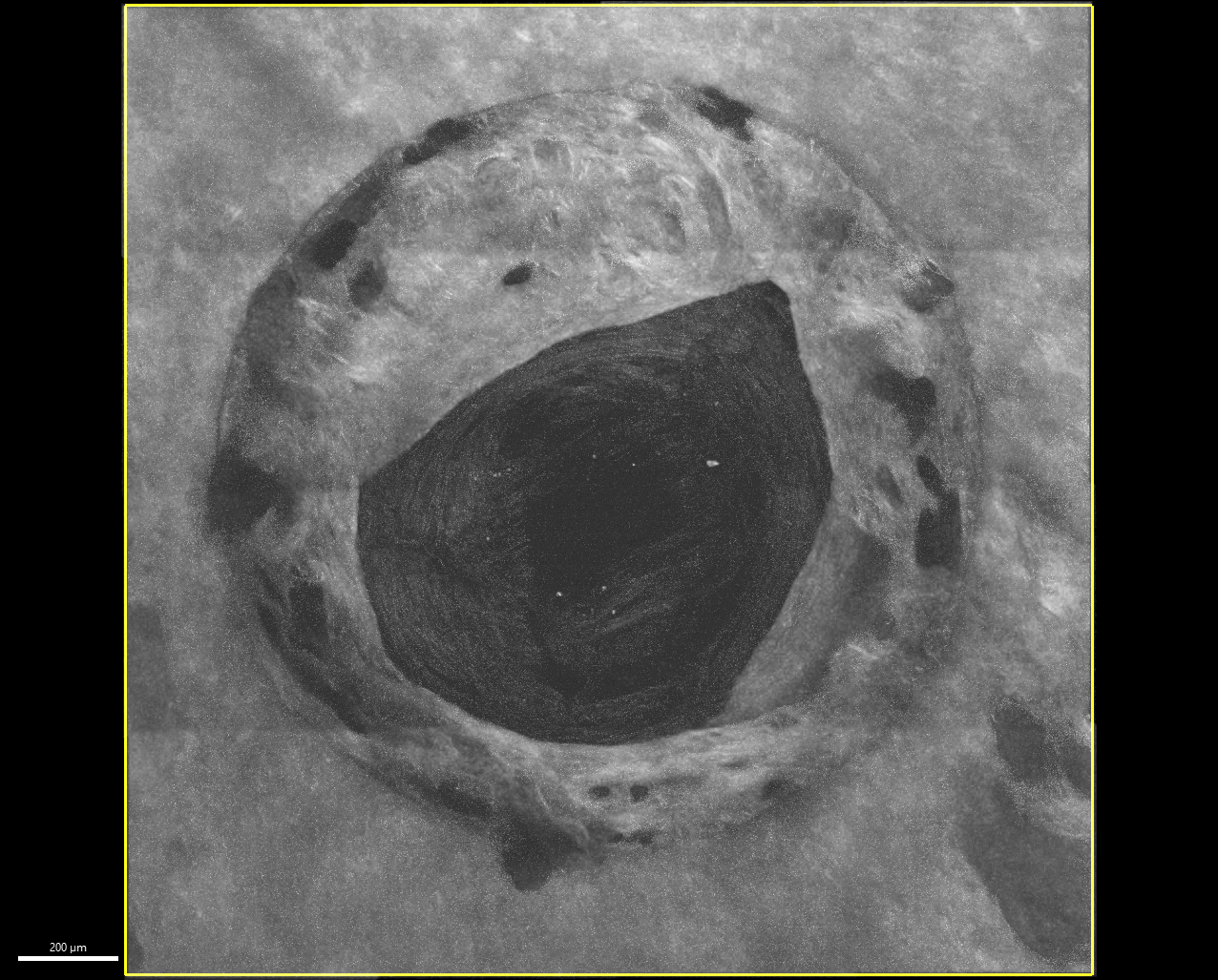

Supplement: Figure 3—source data 1. [file elife-83146-fig3-data1.zip › Figure 3/420+ apln side 2 SHG-2.tif]

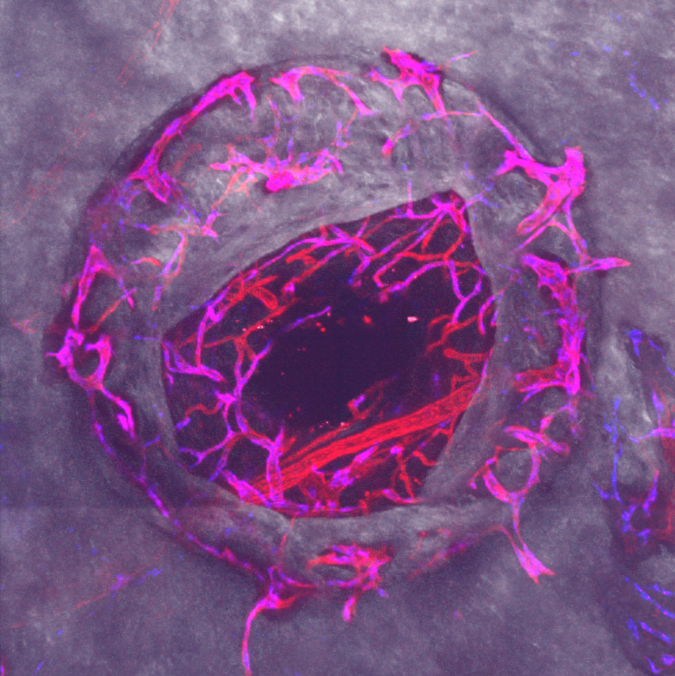

Supplement: Figure 3—source data 1. [file elife-83146-fig3-data1.zip › Figure 3/420+ apln with PTH side 2_fused_110um below cd31 and apln shg.tif]

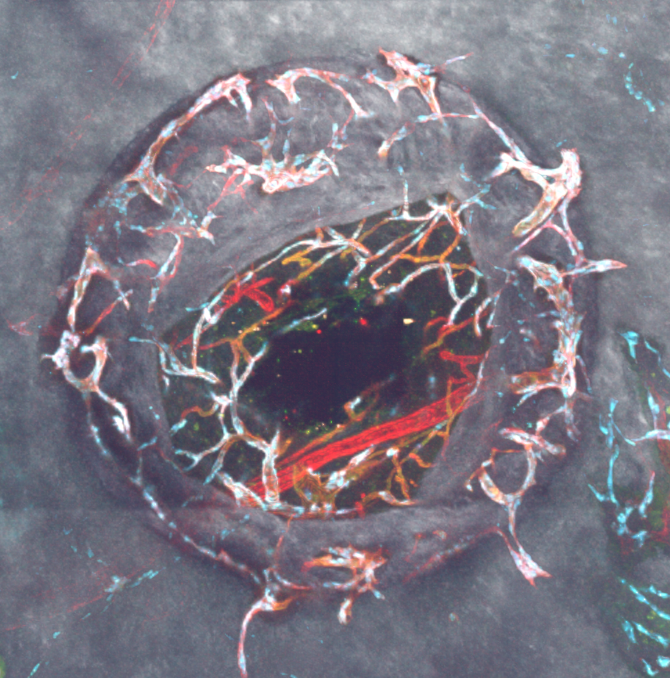

Supplement: Figure 3—source data 1. [file elife-83146-fig3-data1.zip › Figure 3/420+ apln with PTH side 2_fused_110um below cd31 and endo and apln shg.tif]

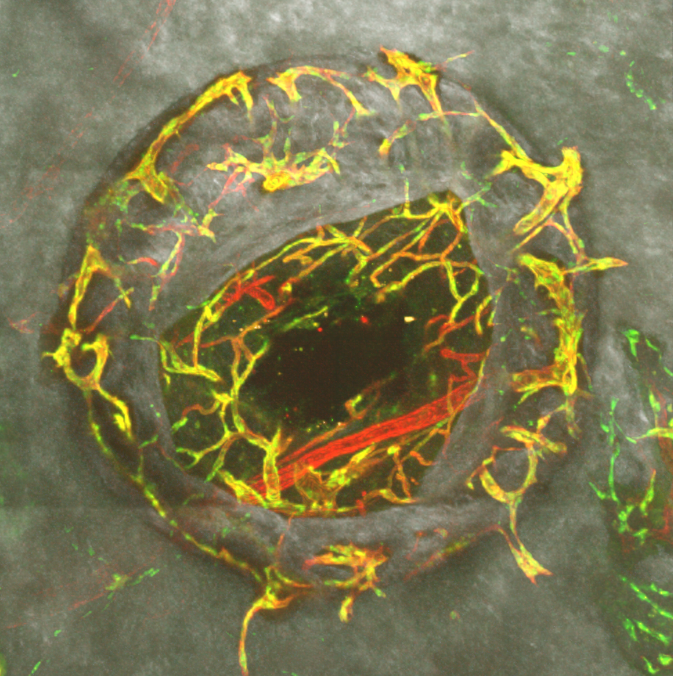

Supplement: Figure 3—source data 1. [file elife-83146-fig3-data1.zip › Figure 3/420+ apln with PTH side 2_fused_110um below cd31 and endo shg.tif]

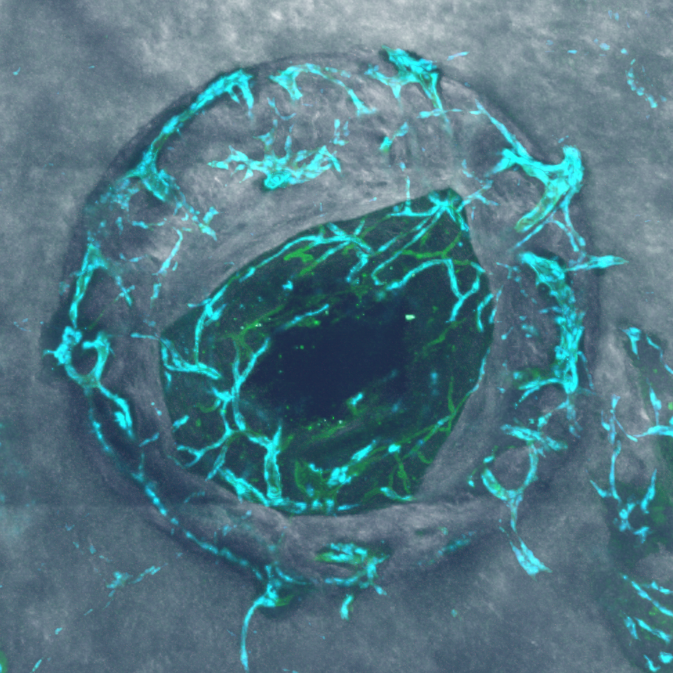

Supplement: Figure 3—source data 1. [file elife-83146-fig3-data1.zip › Figure 3/420+ apln with PTH side 2_fused_110um below endo and apln shg.tif]

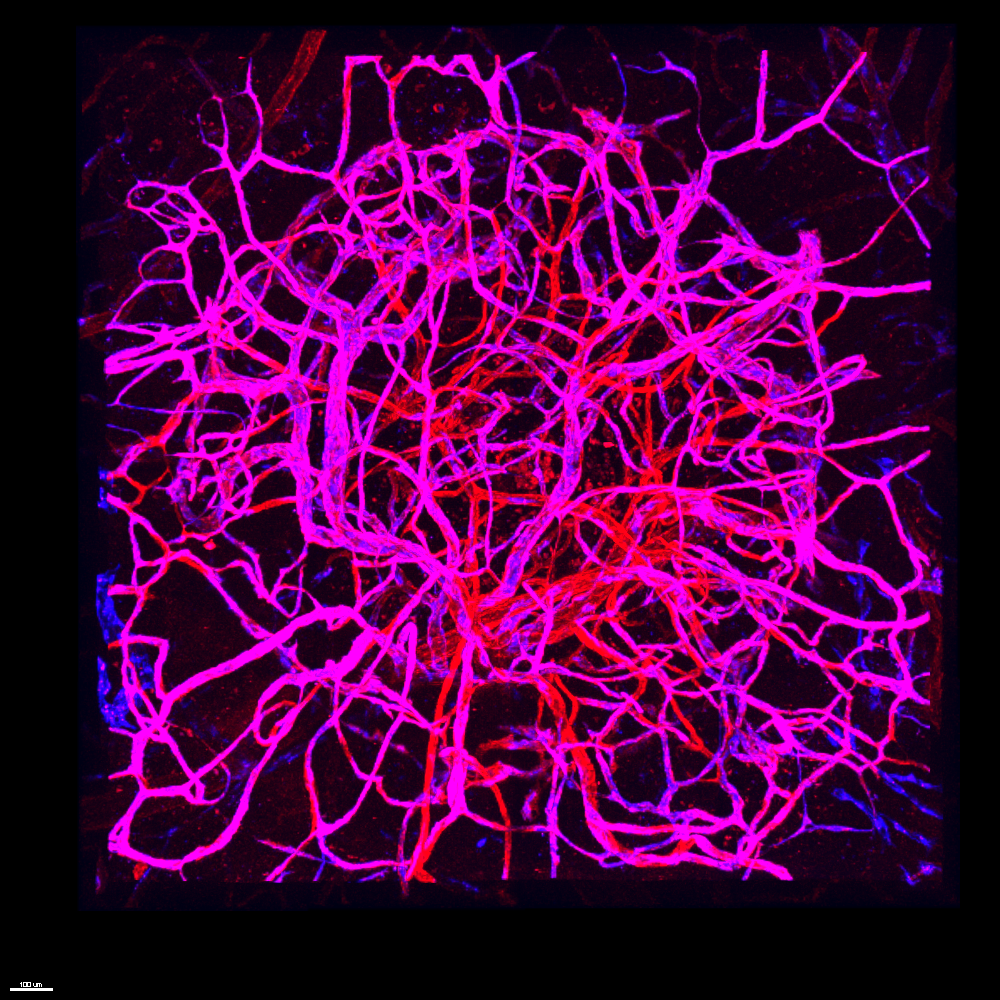

Supplement: Figure 3—source data 1. [file elife-83146-fig3-data1.zip › Figure 3/420+ apln with PTH side 2_fused_cd31 apln.tif]

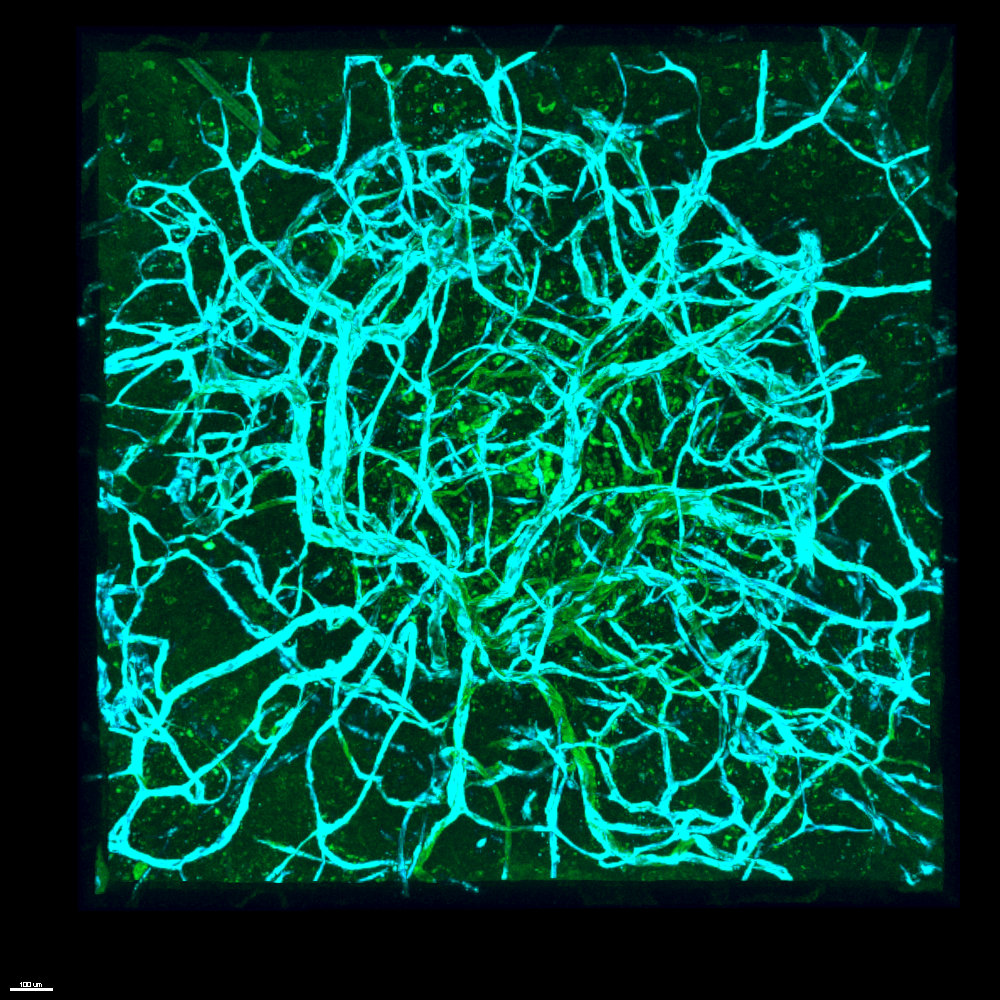

Supplement: Figure 3—source data 1. [file elife-83146-fig3-data1.zip › Figure 3/420+ apln with PTH side 2_fused_endo apln.tif]

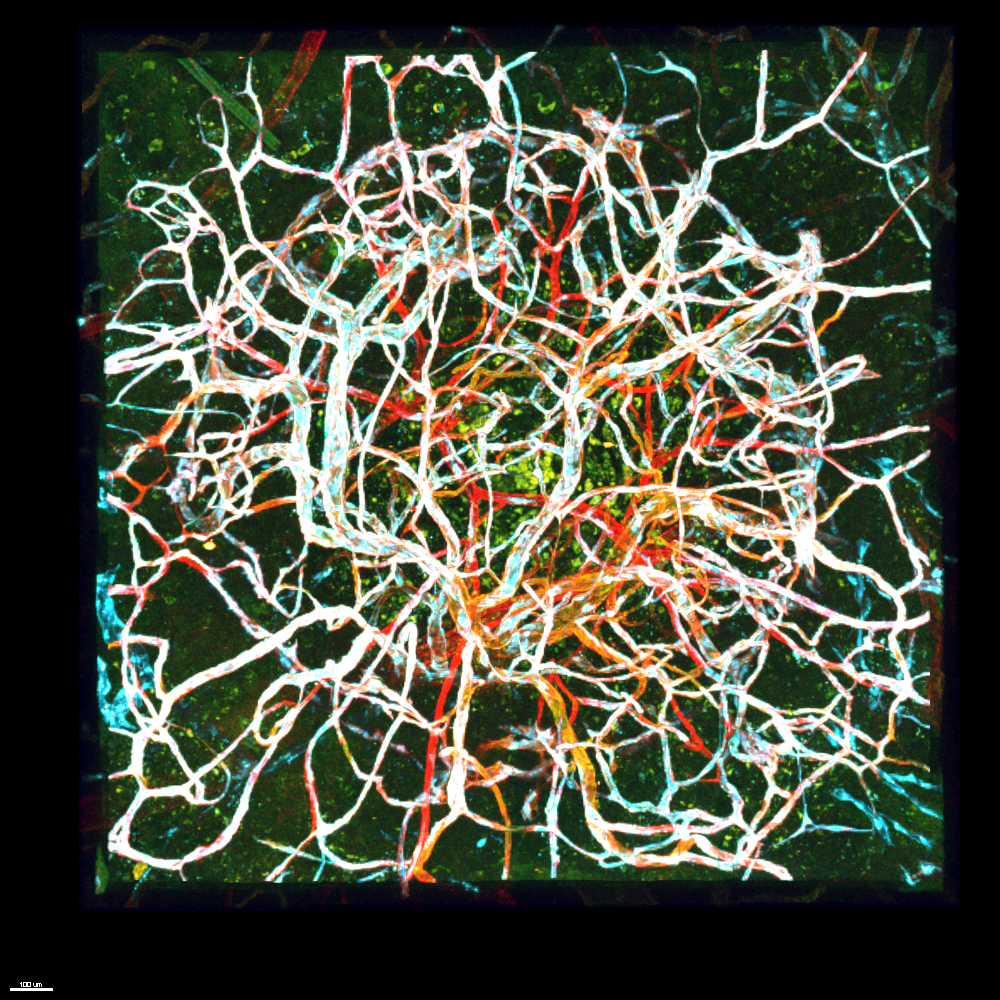

Supplement: Figure 3—source data 1. [file elife-83146-fig3-data1.zip › Figure 3/420+ apln with PTH side 2_fused_endo cd31 apln.tif]

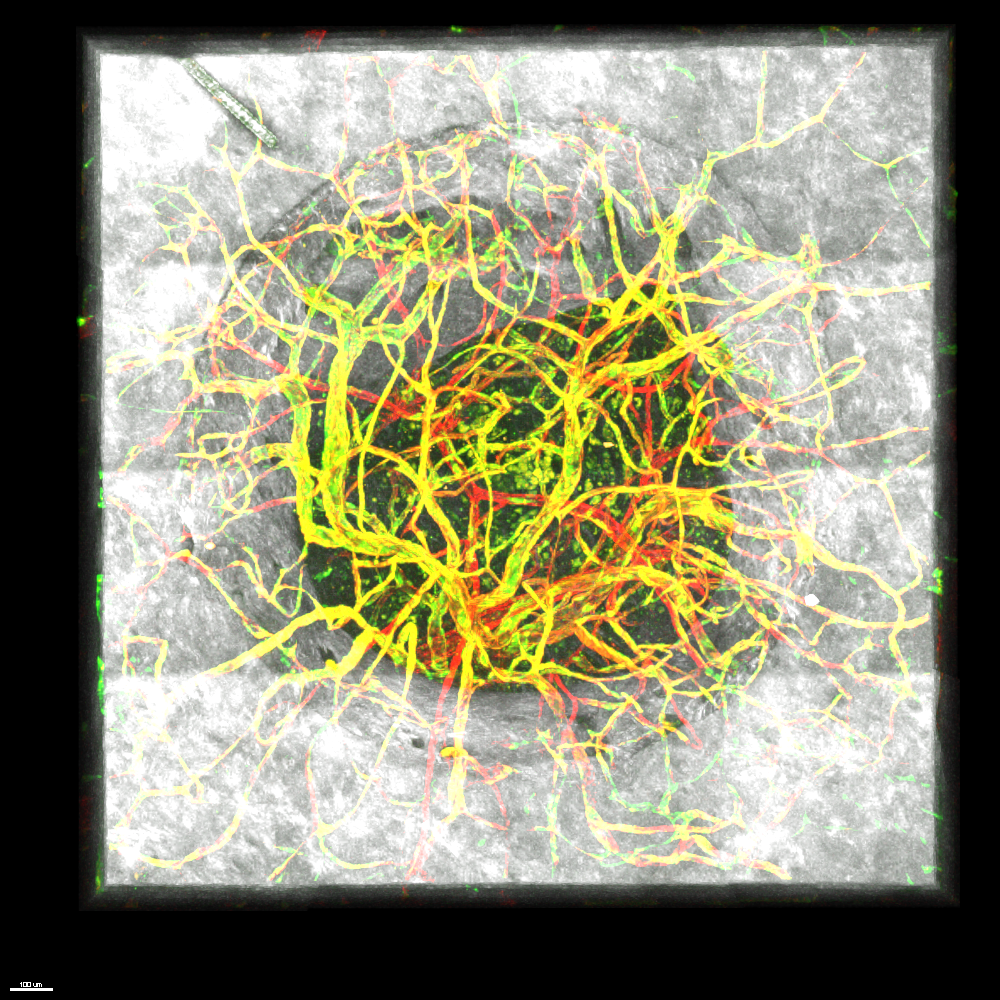

Supplement: Figure 3—source data 1. [file elife-83146-fig3-data1.zip › Figure 3/420+ apln with PTH side 2_fused_endo cd31 shg.tif]

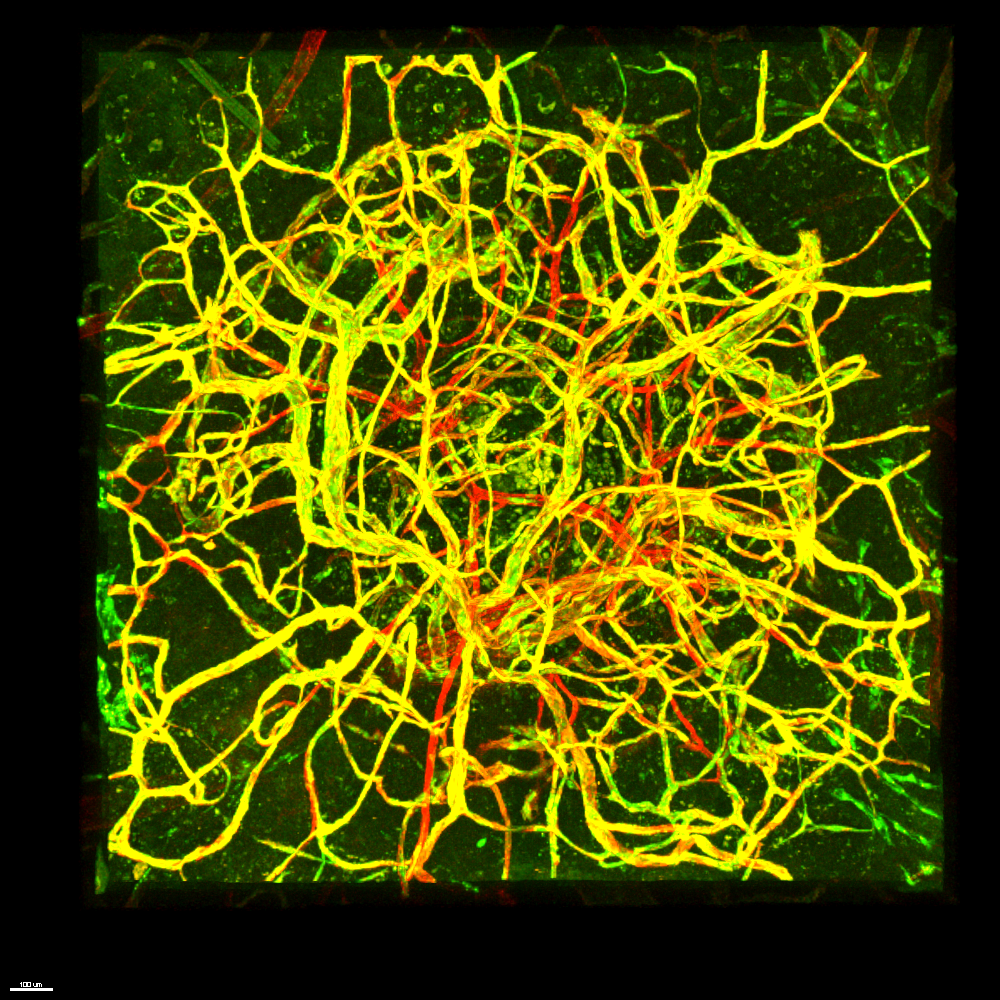

Supplement: Figure 3—source data 1. [file elife-83146-fig3-data1.zip › Figure 3/420+ apln with PTH side 2_fused_endo cd31.tif]

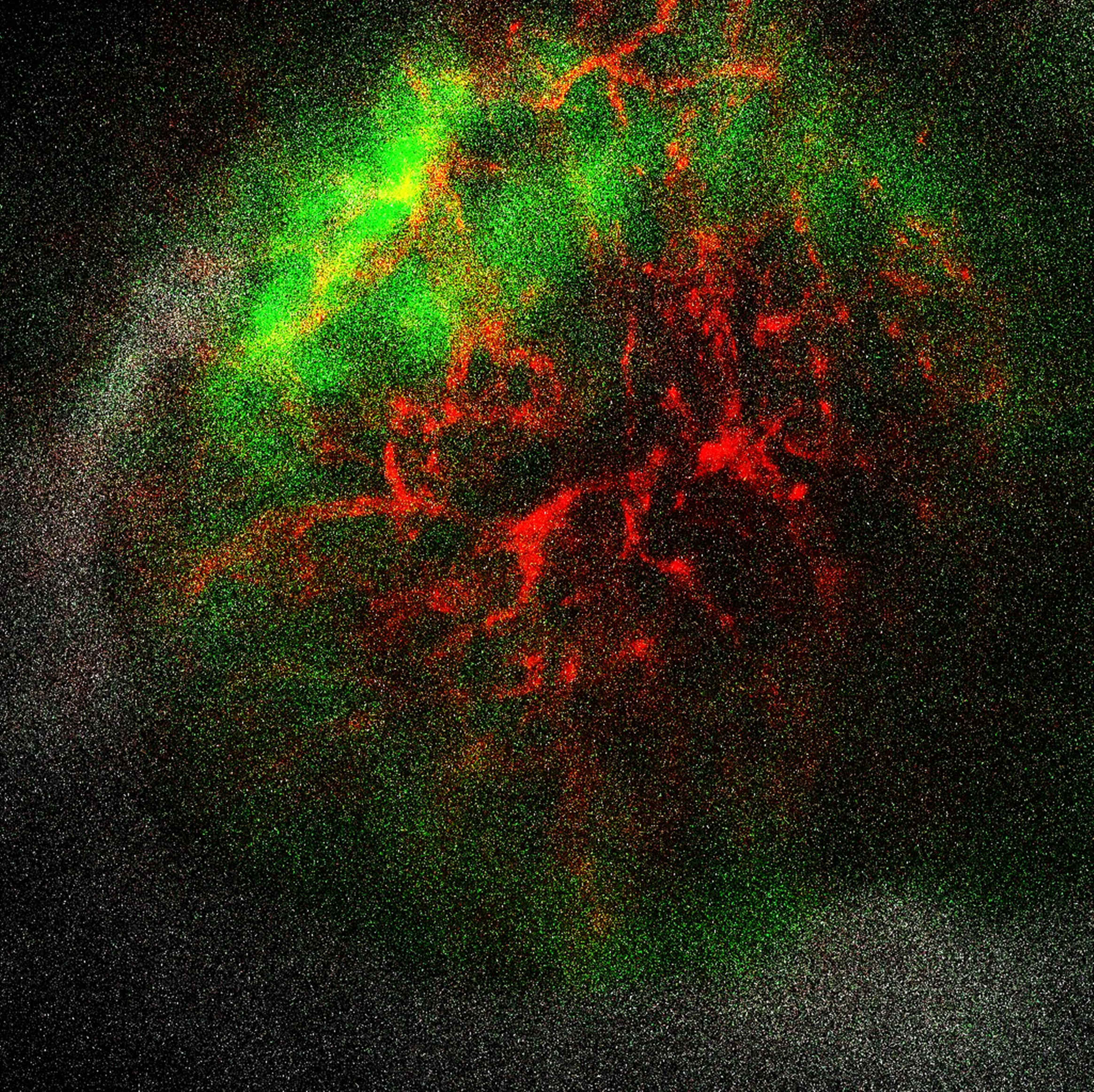

Supplement: Figure 4—source data 2. [file elife-83146-fig4-data2.zip › Figure 4/4A1.tif]

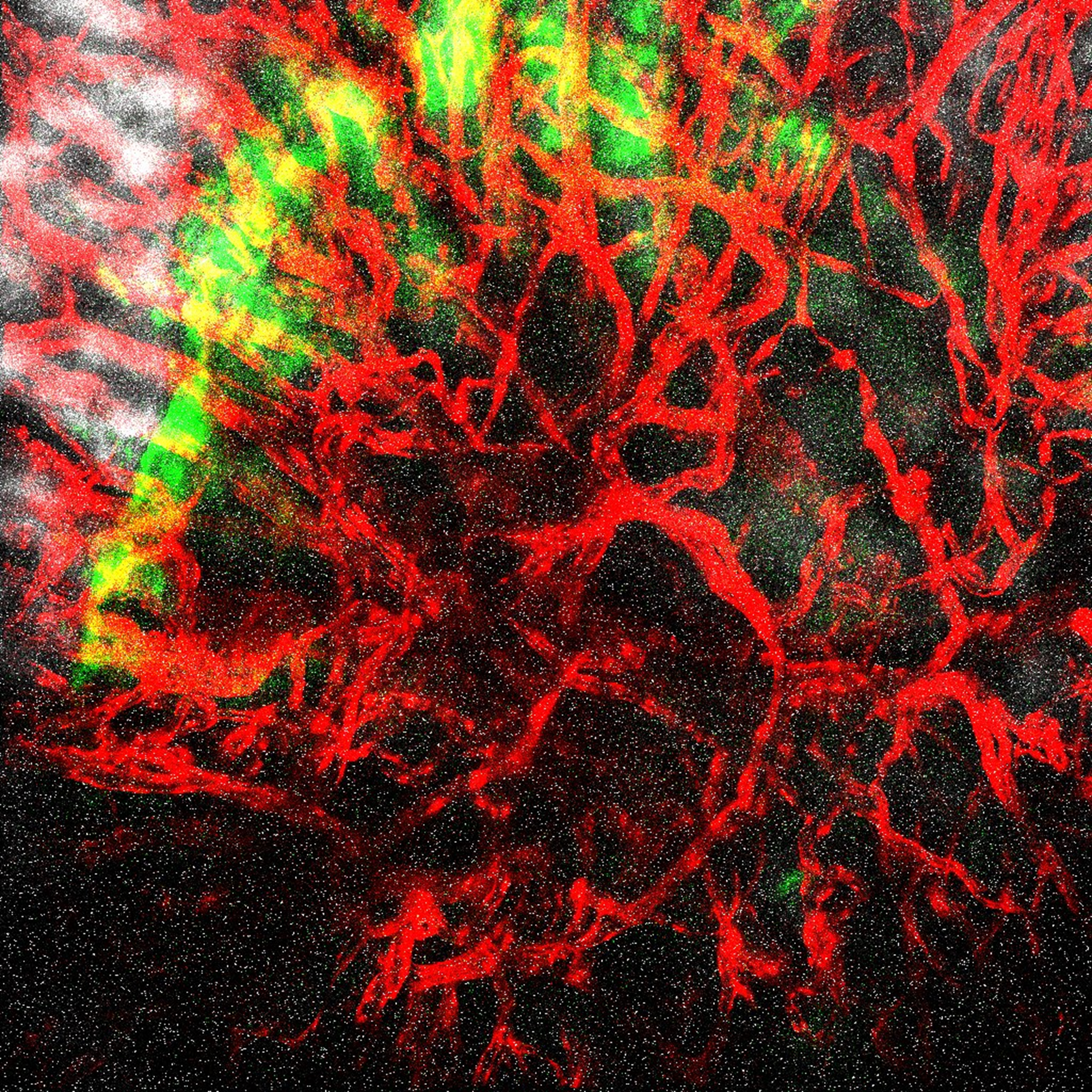

Supplement: Figure 4—source data 2. [file elife-83146-fig4-data2.zip › Figure 4/4A2.tif]

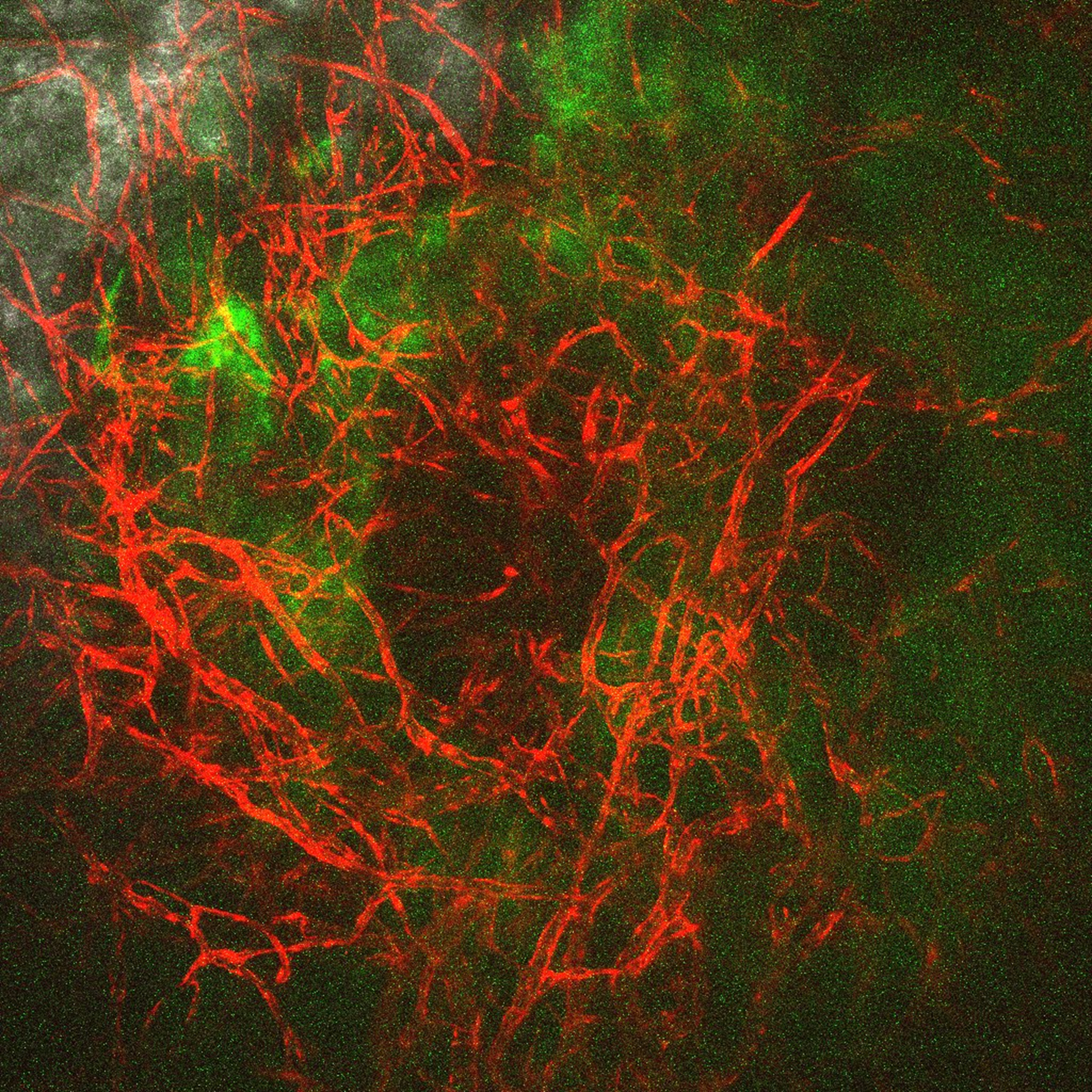

Supplement: Figure 4—source data 2. [file elife-83146-fig4-data2.zip › Figure 4/4A3.tif]

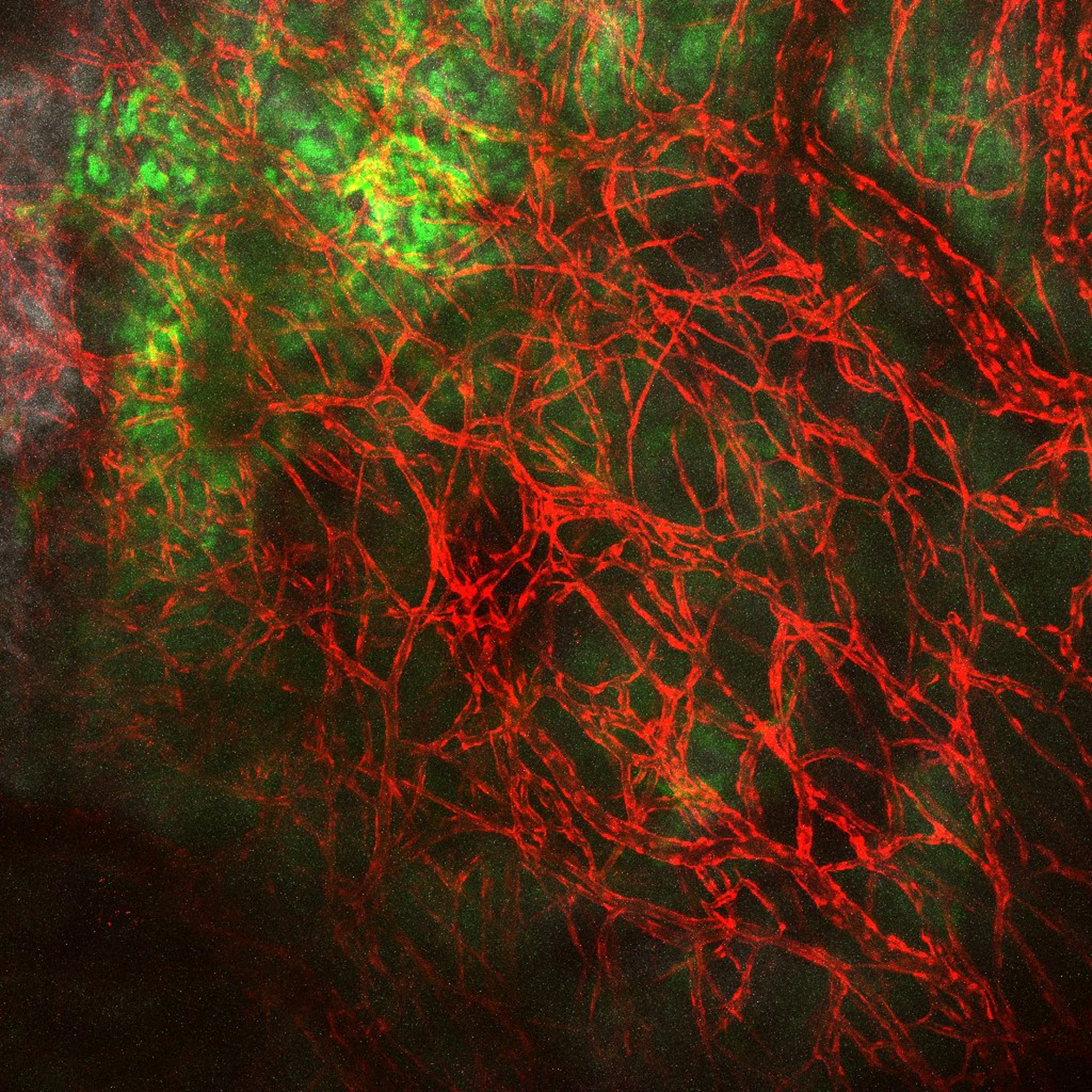

Supplement: Figure 4—source data 2. [file elife-83146-fig4-data2.zip › Figure 4/4A4.tif]

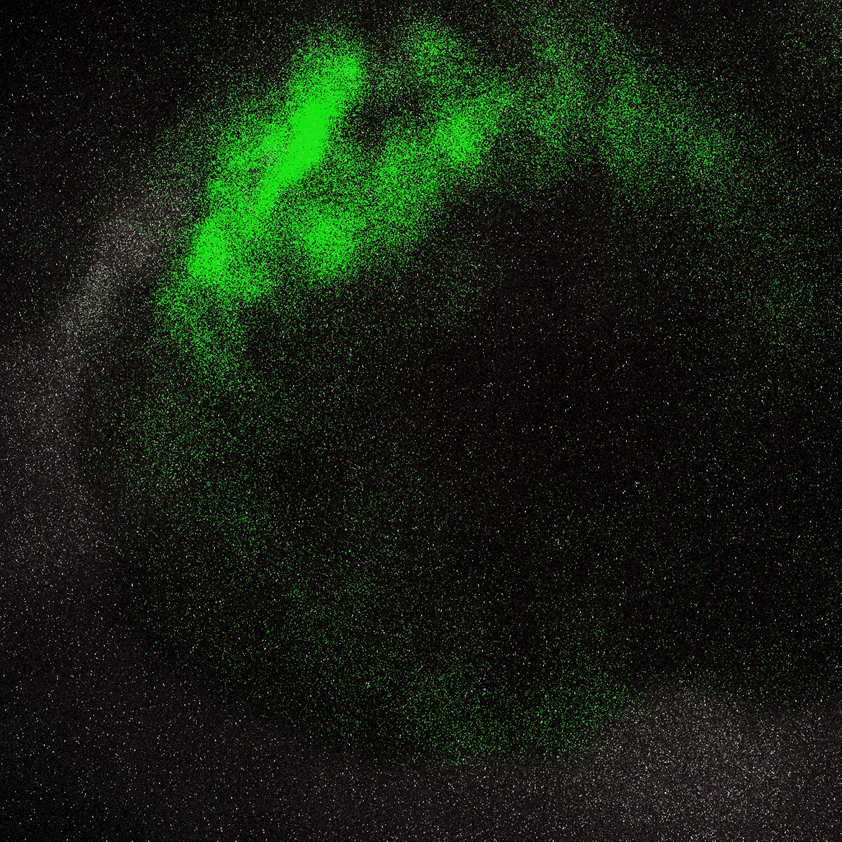

Supplement: Figure 4—source data 2. [file elife-83146-fig4-data2.zip › Figure 4/4B1.tif]

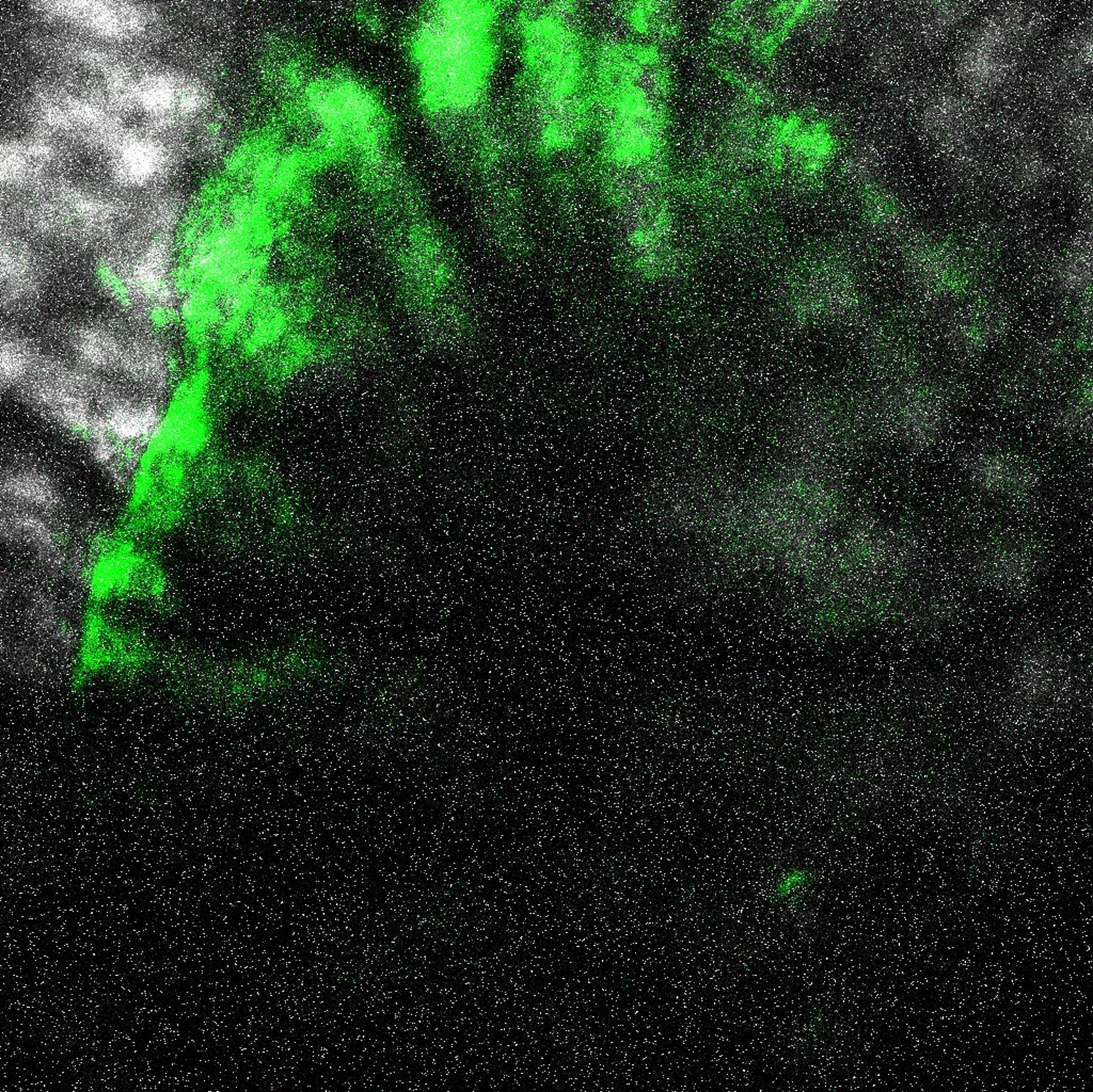

Supplement: Figure 4—source data 2. [file elife-83146-fig4-data2.zip › Figure 4/4B2.tif]

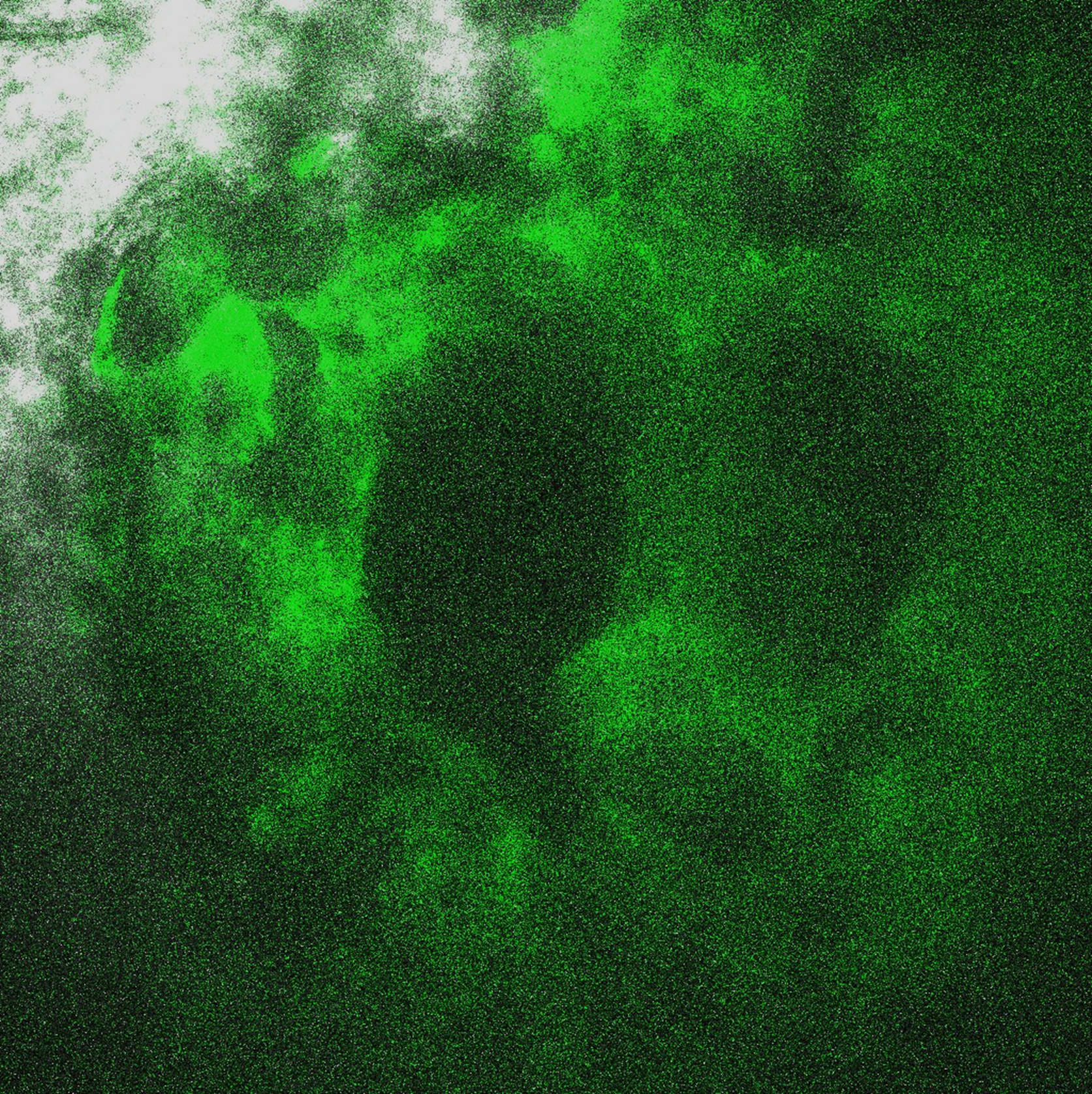

Supplement: Figure 4—source data 2. [file elife-83146-fig4-data2.zip › Figure 4/4B3.tif]

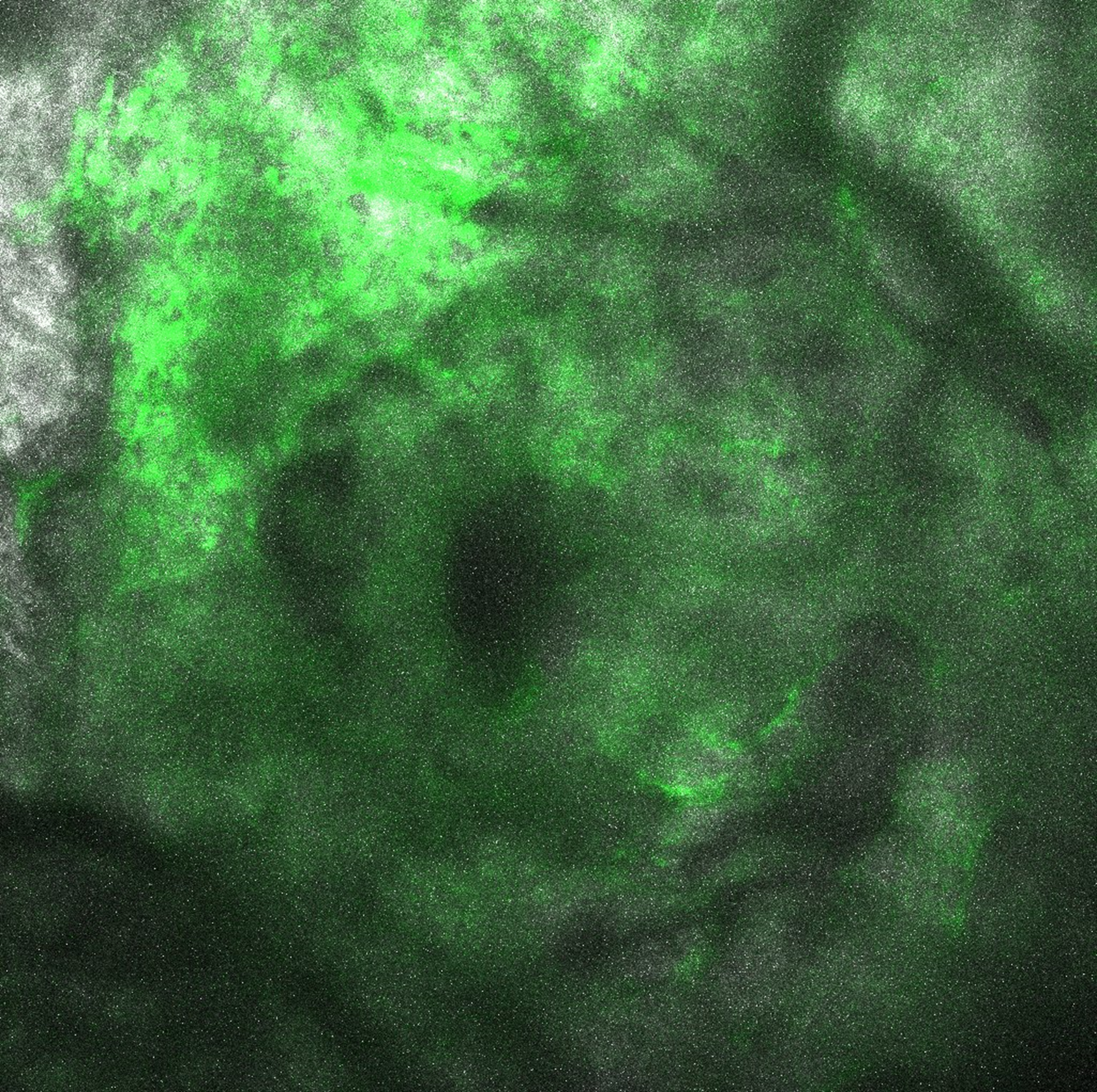

Supplement: Figure 4—source data 2. [file elife-83146-fig4-data2.zip › Figure 4/4B4.tif]

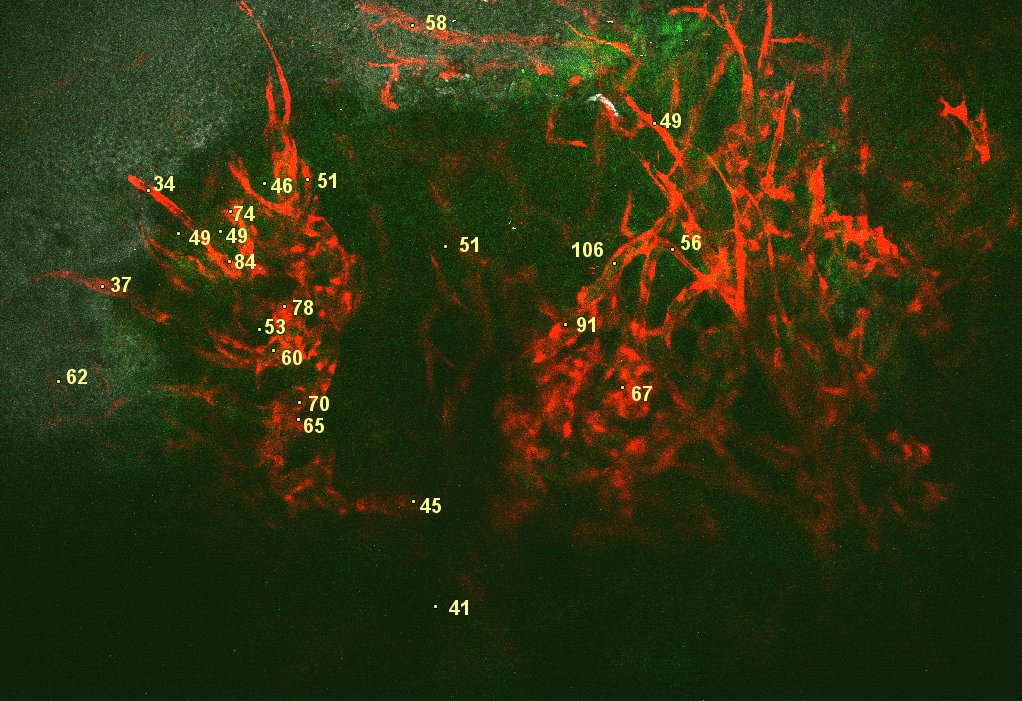

Supplement: Figure 4—source data 2. [file elife-83146-fig4-data2.zip › Figure 4/805_Reg1_Comp_10x-croped labeled new1 slice28.jpg]

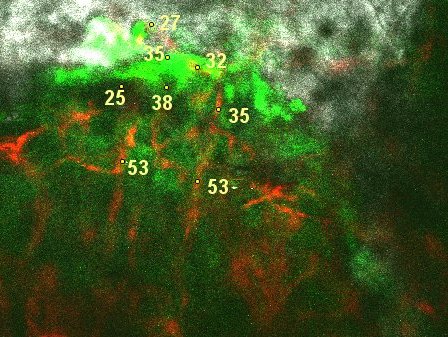

Supplement: Figure 4—source data 2. [file elife-83146-fig4-data2.zip › Figure 4/805_Reg1_Comp_10x-croped labeled new1 slice35 crop.jpg]

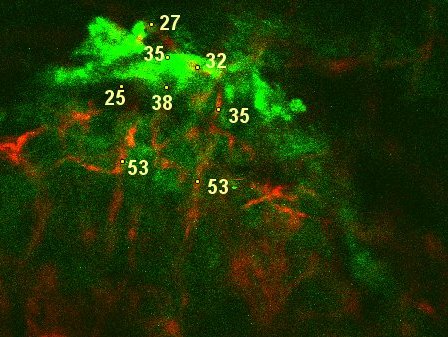

Supplement: Figure 4—source data 2. [file elife-83146-fig4-data2.zip › Figure 4/805_Reg1_Comp_10x-croped labeled new1 slice35 no shg crop.jpg]

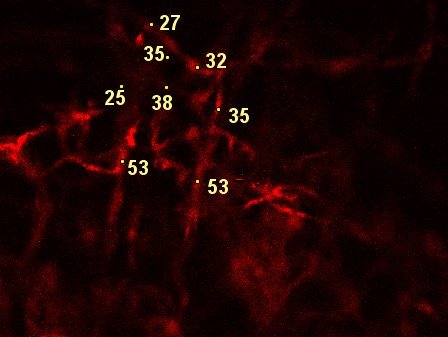

Supplement: Figure 4—source data 2. [file elife-83146-fig4-data2.zip › Figure 4/805_Reg1_Comp_10x-croped labeled new1 slice35 vessel only-crop.jpg]

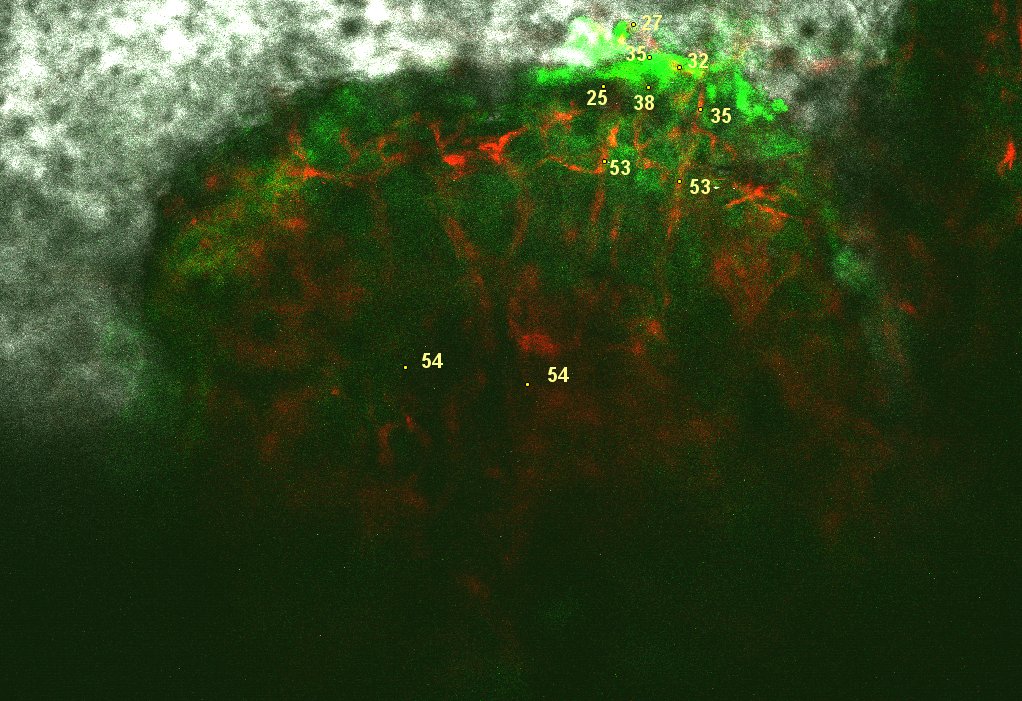

Supplement: Figure 4—source data 2. [file elife-83146-fig4-data2.zip › Figure 4/805_Reg1_Comp_10x-croped labeled new1 slice35.jpg]

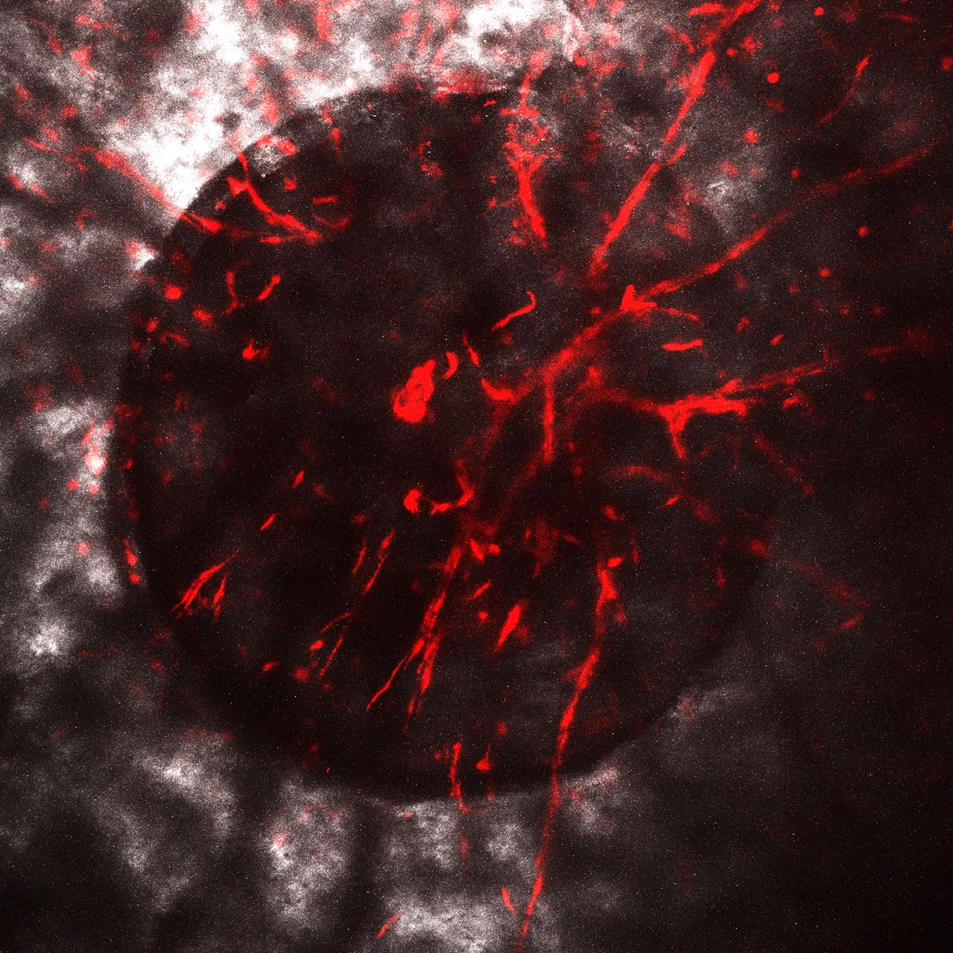

Supplement: Figure 5—source data 1. [file elife-83146-fig5-data1.zip › Figure 5/5A1.tif]

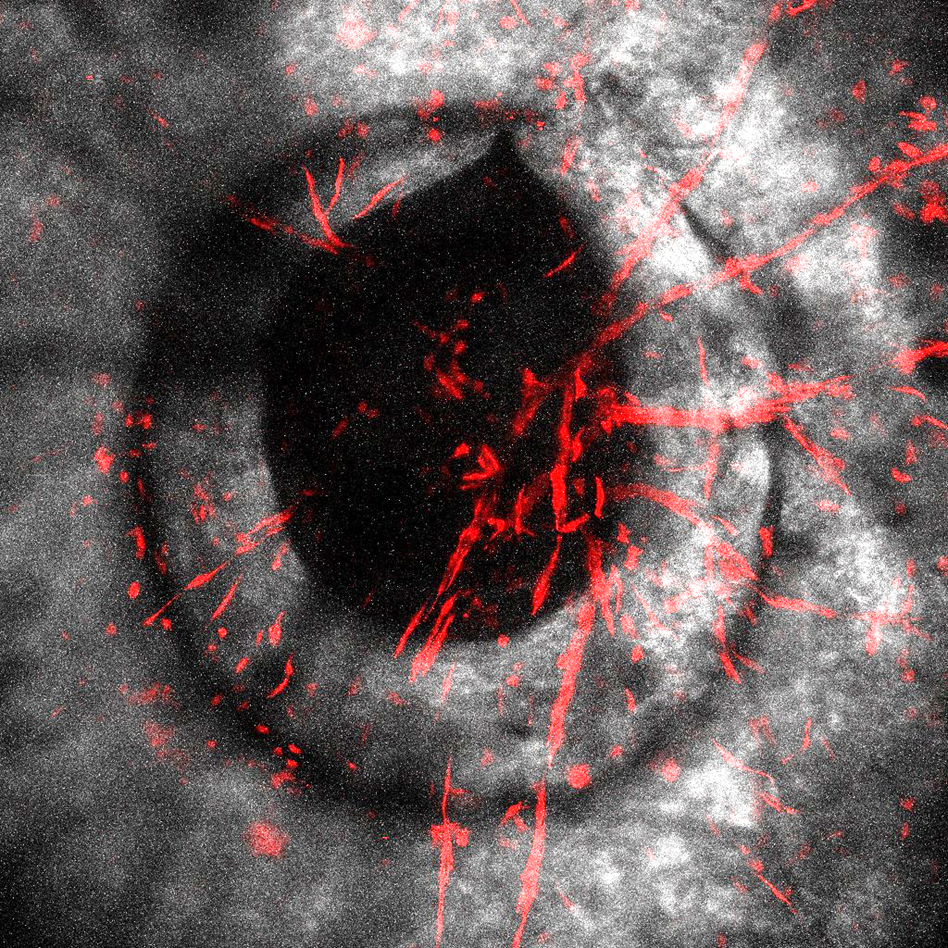

Supplement: Figure 5—source data 1. [file elife-83146-fig5-data1.zip › Figure 5/5A2.tif]

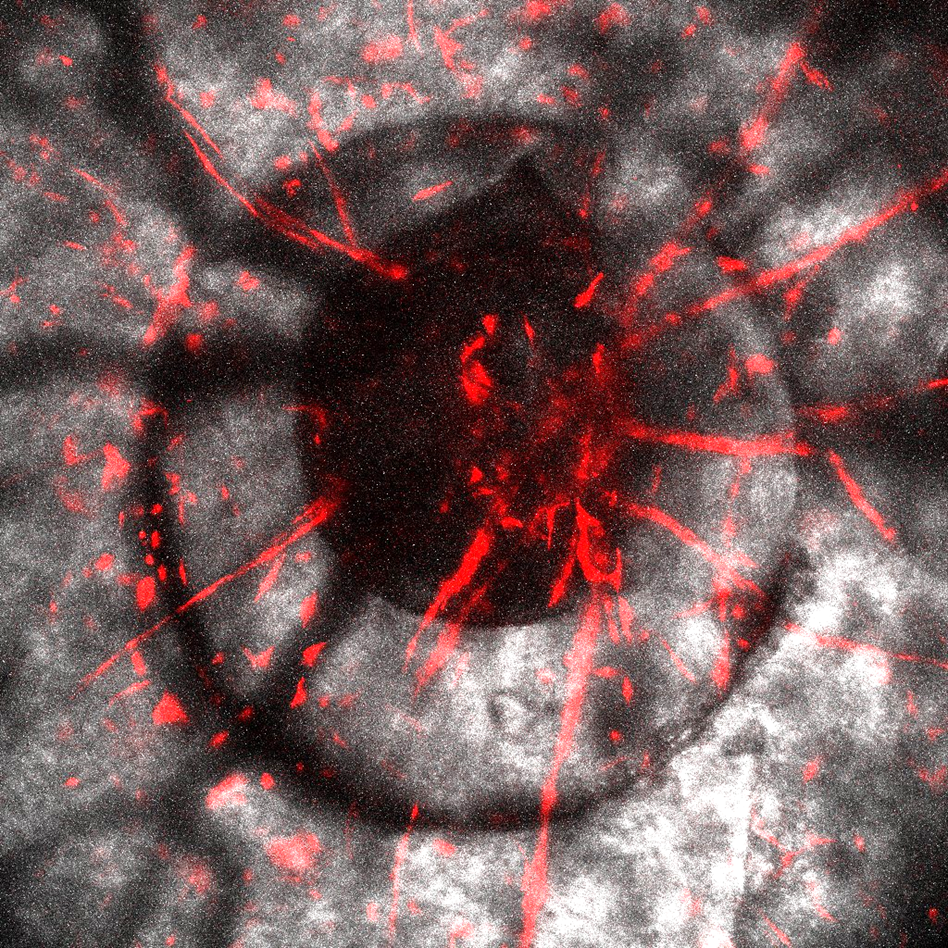

Supplement: Figure 5—source data 1. [file elife-83146-fig5-data1.zip › Figure 5/5A3.tif]

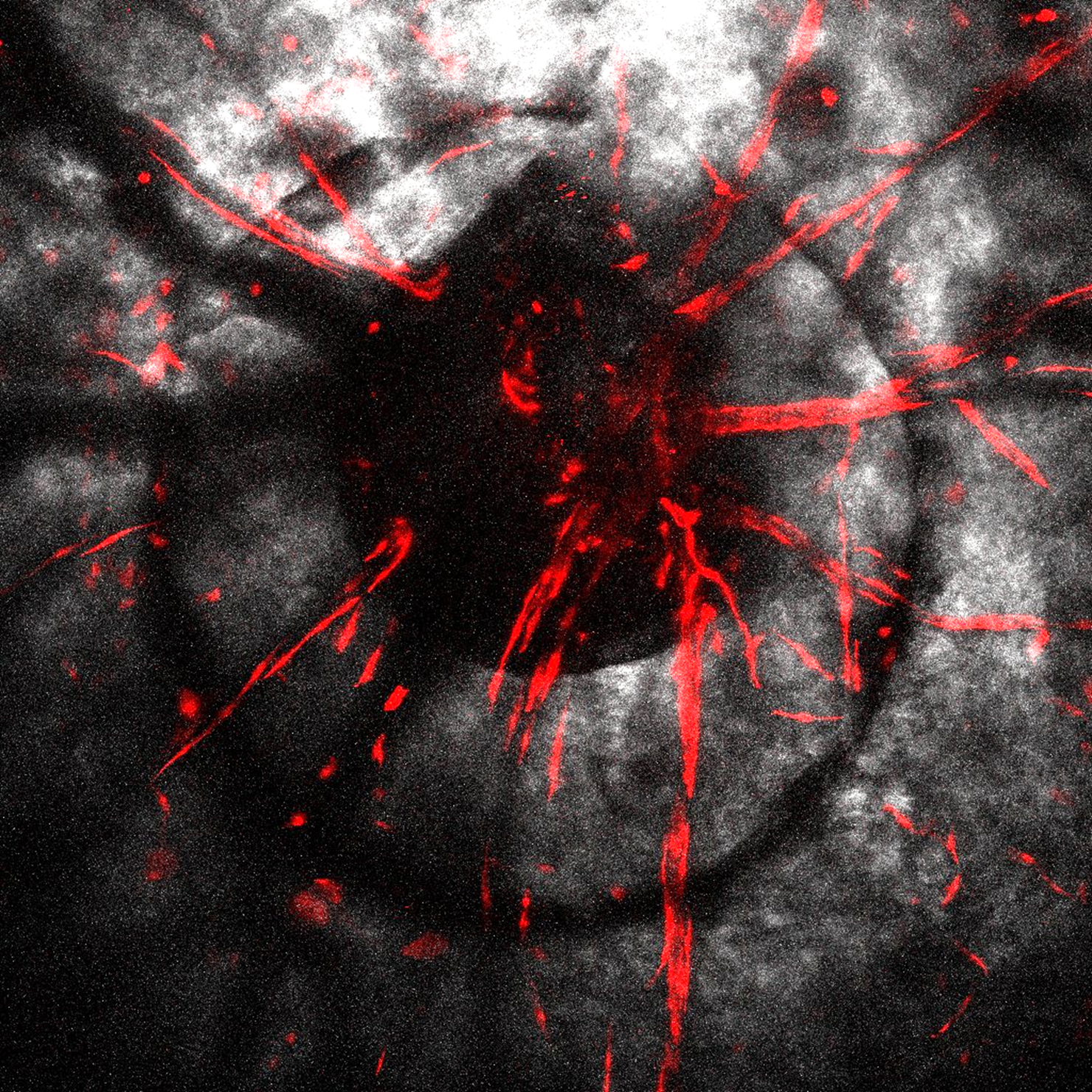

Supplement: Figure 5—source data 1. [file elife-83146-fig5-data1.zip › Figure 5/5A4.tif]
